# Integrated transcriptomic profiling reveals oncogenic pathways and chimeric transcripts in equine sarcoid lesions with predominant BPV1 detection

**DOI:** 10.3389/fmolb.2026.1818241

**Published:** 2026-06-19

**Authors:** Samanta Mecocci, Stefano Capomaccio, Ilaria Porcellato, Filippo Dell’Anno, Roberta Ratto, Luca Mechelli, Livia De Paolis, Floriana Fruscione, Benedetta Passeri, Rodolfo Gialletti, Marco Pepe, Alessandro Ghelardi, Elisabetta Razzuoli, Katia Cappelli

**Affiliations:** 1 Department of Veterinary Medicine, University of Perugia, Perugia, Italy; 2 Sports Horse Research Center (CRCS), University of Perugia, Perugia, Italy; 3 National Reference Center of Veterinary and Comparative Oncology (CEROVEC), Genoa, Italy; 4 Department of Veterinary Medicine, University of Parma, Parma, Italy; 5 UOC Ostetricia e Ginecologia, Azienda Usl Toscana Nord-Ovest, Massa, Italy

**Keywords:** bovine papillomavirus, chimeric transcripts, host-pathogen interaction, hyppo signaling pathway, RNA sequecing, sarcoids

## Abstract

Sarcoids are the most common cutaneous tumors in horses, representing up to 90% (35%–90%) of skin neoplasms. Mostly caused by Bovine Papillomavirus (BPVs) infections, sarcoids are highly resistant to therapy and prone to recurring, posing a significant threat to equine health. The aim of this study is to explore molecular pathogenetic mechanisms underlying the development of equine sarcoids, by applying transcriptomic approach. After testing samples for viral DNA, both mRNA and small RNA expression was analyzed via high-throughput Illumina sequencing comparing 12 sarcoids and 12 healthy skin samples as controls. Differentially expressed genes (DEGs), DE miRNAs (sarcoids vs. controls) and miRNA-DEG couples with opposite expression trends, were retrieved and subjected to a functional analysis. Over 6K DEGs emerged, 3620 down-regulated and 2415 up-regulated along with 145 DE miRNAs, 56 downregulated and 89 upregulated. Among the enriched biological processes for DEGs, some were related to growth factors production and collagen binding, cell migration and proliferation, tissue morphogenesis and inflammatory response. Interestingly, “Pathways in cancer” and “Hippo signaling pathway” were enriched KEGG pathways for the miRNA-DEG couples. Our data identified a great transcription discrepancy between sarcoid lesions and healthy skin with an overall enrichment for processes related to cellular transformation. RNA-seq sequencing depth allowed the search for candidate chimeric transcripts associated with viral integration events. Chimeric RNAs can influence gene regulation and may contribute to tumor growth and immune modulation. Via computational analysis we identified six fusion loci in tumor samples and in two sarcoid margins, with the most frequent event involving WNT10B and FKBP11. This fusion, detected in 6/10 sarcoids, is of particular interest since WNT10B activates the WNT/β-catenin cascade, while FKBP11 has been implicated in osteosarcoma progression. Although functional validation is ongoing, this represents the first report of chimeric transcripts in equine sarcoids, opening new perspectives on BPV-driven oncogenesis.

## 1 Introduction

Equine sarcoids are locally aggressive, non-metastatic skin tumors, affecting up to 12% of horses worldwide. They are the most common neoplastic disease in horses and other equids such as donkeys, mules, or zebras, representing up to 90% of all equine cutaneous tumors (Ogłuszka et al., 2021). Bovine papillomavirus (BPV) type 1, 2, and 13 (BPV1, BPV2, BPV13) play a central role in the etiology of equine sarcoid and the disease is considered the result of a non-productive infection (Trewby et al., 2014). The viral type and the epidemiological proportion between types involved depends on the country in which the horse live (Goodrich et al., 1998; Hainisch et al., 2022; Munday et al., 2021; Gysens et al., 2022). The main way of transmission is by direct contact, contaminated fomites, and shared living environments, but also vertical transmission has been suggested in equids by evidence of BPV gene expression in the blood and semen of healthy horses, as well as in the placenta. Moreover, it has been shown that co-stabling of sarcoid-affected and healthy donkeys can result in the transmission of BPV1, with insects suspected as possible transmission vectors (Trewby et al., 2014). Papillomaviruses (PVs) belong to the large family of animal and human Papillomaviridae that normally infect epithelial cells, mostly causing benign proliferative lesions known as warts. On the other hand, some types of PVs can induce benign and malignant tumors in both humans and animals, including BPV types 1 and 2. These viral types can also infect fibroblasts and induce fibroepithelial tumours, like benign fibropapillomas in cattle (Brandt et al., 2008; Nasir et al., 2007). Among the most common and specie-specific equine PVs is EcPV2, which is related to equine genital squamous cell carcinomas (Mecocci et al., 2021; Porcellato et al., 2020; Porcellato et al., 2021; Armando et al., 2021). Although PVs are normally strictly species-specific and are characterized by a pronounced tropism for cutaneous and mucosal keratinocytes, equine and feline sarcoids are a well-known case of natural cross-species PV infection (Munday et al., 2015; Nasir et al., 2007). Although biology, morphology, and epidemiology of equine sarcoids are known, the pathogenic events leading to the development of tumors and the mechanisms used by BPV to induce the lesions are poorly understood. In general, tumorigenesis is a complex process that involves numerous molecules and pathways; in equine sarcoids BPV1 and BPV2 may be responsible for the abnormal fibroblast proliferation and the alterations in the metabolism of extracellular matrix (ECM) and its main components (e.g., collagen) (Martano et al., 2016). One underexplored mechanism concerns the formation of chimeric transcripts. These fusion events, recently investigated in HPV virus-associated cancers (Jayachandran et al., 2023) may alter gene expression and cellular function, contributing to tumorigenesis. The application of RNA sequencing for chimeric transcript discovery in equine sarcoids represents a new approach, to our knowledge, in the equine species, to unravel important hot-spots potentially leading to new diagnostic, prognostic, and therapeutic opportunities.

Sarcoids are typically diagnosed through a combination of clinical presentation and histopathological evaluation. Polymerase chain reaction (PCR) from superficial swabs, skin scrapings, or the tumour mass can be helpful for the identification of BPV DNA that is thought to be diagnostic for equine sarcoid (Ogihara et al., 2021; Tura et al., 2022). Recently, hypotheses have emerged suggesting also a potential role of ovine papillomavirus in the development of equine sarcoids (De Falco et al., 2024).

The equine sarcoid remains a clinical challenge since the high risk of treatment failure and local invasion is a major constraint to therapy. The likelihood of recurrence (in the same origin site or growth of a new sarcoid) is highest wheb surgical therapeutic methods are applied and it seems to be correlated with the presence of BPV DNA on the surgical margins (Goodrich et al., 1998; Mosseri et al., 2014). Natural immunity against BPV1 and two in equids appears to be poor and sarcoid-affected horses show no measurable anti-BPV1 L1 antibodies. This circumstance may help to explain why sarcoids are usually present as persistent lesions; BPV may escape from immune surveillance because of its paramount localization in cutaneous cells, yet also because of its capacity to inhibit MHC class I-mediated antigen presentation *via* its major oncoprotein, E5 (Hainisch et al., 2012). Given the current challenges in establishing effective therapies, the identification of the key cellular drivers of sarcoid growth paves the way for the future development of brand-new targeted therapeutic approaches (Martano et al., 2016).

In this study, deep RNA and miRNA sequencing were applied to better understand host-pathogen interactions and the tumor microenvironment in equine sarcoid, taking advantage of Next-Generation Sequencing (NGS) technologies. RNA-seq indeed is a gold standard technology for investigating transcriptomic patterns detecting and large number of genes and regulatory regions in specific physiological and pathological conditions quantifying the expression profile (Satam et al., 2023).

## 2 Methods

### 2.1 Sample collection

Horses and donkeys enrolled for this study (Table 1) were examined at the Didactic Veterinary Hospital (OVUD) of the Veterinary Medicine’s Department of the University of Perugia. Written informed consent was obtained from the animal owners prior to their inclusion in the study and sample collection.

**TABLE 1:** Recruited horses and donkeys. F: female; M: male; G: gelding.

Cases	Lab ID	Spieces/ Race	Sex	Age	Lesions’ site
1	T1	*Equus Asinus*	F	Adult	Periocular
2	T2	*Equus Caballus, Thoroughbred*	F	12	Abdomen udder
3	T3	*Equus Caballus, Italian saddle*	G	7	Periocular
4	T4	*Equus Caballus, Italian saddle*	M	5	Periocular
5	T5	*Equus Caballus, Arab*	F	14	Paramammary
6	T6	*Equus Caballus, Akal teeke*	G	13	Abdomen
7	T7	*Equus Caballus, Belgian horse*	M	5	Scrotum and ear
8	T9	*Equus Caballus, Pony*	F	9	Right rear hock
9	T10	*Equus Asinus*	G	5	Periocular
10	T12	*Equus Caballus, Pony*	F	21	Abdomen
11	T14	*Equus Caballus, Italian saddle*	G	7	Abdomen inner thigh
12	T15	*Equus Caballus, Italian saddle*	F	6	Inner thigh and chest

Animals with concomitant diseases or poor clinical conditions were excluded. The lesions were surgically removed with a 3 cm lateral margin. The excised lesion and margin were subjected to histopathological evaluation and polymerase chain reaction (PCR) to assess the presence of viral DNA. After sampling for histology and margin assessment, part of the residual tissues of tumor samples and tumor-free margin samples (potential paired controls from the same animal) were stored at −80°.

The inclusion parameters were:

- clinical and confirmed histopathological diagnosis of sarcoid,
- lesions >2 cm in diameter.

After histological confirmation of diagnosis and assessment of margin status, samples meeting the selection criteria were used for NGS, together with ten cutaneous tissues collected from heathy donors at slaughterhouse that tested negative for viral DNA (control group).

### 2.2 Histopathological characterization

Surgical samples were submitted to routine processing for histopathology, with evaluation of surgical margins. Margins were sampled using a combined evaluation using cross-sectioning for smaller samples (<4 cm as major diameter) and a combination of cross and “bread loaf” sectioning for larger samples (Kamstock et al., 2011).

The histopathological diagnosis of equine sarcoid, with variable degree of diagnostic certainty, was based on the presence of one or more of the following features:

- spindle cell neoplastic proliferation.
- presence of epidermal hyperplasia with deep rete ridges (rete pegs) interdigitating with the dermal proliferation.
- “picket fence” arrangement of neoplastic cells in the subepithelial area (rows of fibroblasts with a perpendicular orientation of the epidermal basement membrane).

### 2.3 DNA extraction and PCR assay for BPV detection

DNA was extracted starting from frozen and grinded samples using RecoverAll Total Nucleic Acid Isolation Kit (Termo Ficher Scientific, Waltham, Massachusetts, United States) and following manufacturer’s instructions. The presence/absence of BPV DNA was assessed for all the samples (sarcoid lesions, margins and controls) by applying a Taqman assay on the L1 genomic region of the 3 BPVs (BPV1, BPV2 and BPV13) using primers and probes (Table 2) designed through Primer3 web-tool (https://primer3.ut.ee).

**TABLE 2:** Primer sequences and probes used for the detection of BPV1, -2, -13 DNA in Real Time PCR (BPV1, BPV2, BPV13). Beta-2-Microglobulin (B2M) was used as the reference gene. Table also reports primers for gene expression of early viral oncogenes BPV1-E5, E6 and E7.

Target	Primer pairs	Probe	Amplicon size	Accession number
BPV1-L1	*For*-5′-CAGGACTGTTCACAACCCAAG-3′	*FAM*-TGCAGGTGTCCAGAGGGCAG-*TAMRA*	97	JX678969
	*Rev*-5′-CCCAGTTACAGTACCTCCAAGA-3′			
BPV2-L1	*For*-5′-ACAGCCCGTCCATGTGTTA-3′	*FAM*-AGAAAATGGTGCGTGTCCTCCT-*TAMRA*	116	M20219
	*Rev*-5′-TCAGCAGCACCAAACCCTAT-3′			
BPV13-L1	*For*-5′-GCACCCCACTTTTAATGCCT-3′	*FAM*-AGGAAAGTGACCAGCCAAACAACA-*TAMRA*	88	NC_030795
	*Rev*-5′-TCCTGTTTGCTTCCTGTCATC-3′			
B2M (DNA)	*For*-5′-CTGATGTTCTCCAGGTGTTCC-3′	*FAM*-ACTCACGTCACCCAGCAGAGA-*TAMRA*	136	NM_001082502.3
	*Rev*-5′-TCAATCTCAGGCGGATGGAA-3′			
BPV1-E5	*For: 5′-TGCTTCAATGCAACTGCTGCT-3′*	*-*	77	JX678969
	*Rev: 5′-AGGAGCACTCAAAATGATCCCAG-3*			
BPV1-E6	*For: 5′-TGCTACTGTGGGGGCAAACT-3′*	*-*	110	JX678969
	*Rev: 5′-CAGTCGTAGCAGCGTCCTCT-3′*			
BPV1-E7	*For: 5′-GCTGTGGAAACTGCGGAAAA-3′*	*-*	122	JX678969
	*Rev: 5′-CAGTTTGGGATTGAAAGGTTTG-3*			
B2M (RNA)	*For: 5′-GGCTACTCTCCCTGACTGG-3′*	*-*	135	JX678969
	*Rev: 5′-TCAATCTCAGGCGGATGGAA-3′*			

For Real Time qPCR detection, 5 μL of template were added to the reaction including 1X CustomProbe, 2x qPCR Master Mix (Canvax Reagents SL, Valladolid, Spain), 200 nM of probe and100 nM of each primer combination. The thermal protocol used for amplification in a CFX96 TM Real- Time (Bio-Rad, California, USA) was 95 °C for 10 min, followed by 40 cycles of 95 °C for 15 s, 60 °C for 60 s.

### 2.4 RNA extraction, sequencing of small RNA and mRNA

The 12 samples of tumour tissue, two histologically healthy portions of the T1 and T5 sarcoid margin and the 10 samples of healthy skin were subjected to total RNA extraction with a commercial kit (miRNeasy Kit, Qiagen, Hilden, Germania), following the manufacturer’s instructions. The extracted RNA was qualitatively and quantitatively evaluated by spectrophotometric measurement with NanoDrop 2000 (Thermo Fisher Scientific, Waltham, MA, USA) and by microfluidic electrophoresis (Bioanalyzer 2,100 Agilent Technologies). RNA samples were used to produce two different sequencing libraries, one for mRNA using the TruSeq RNA Library Prep Kit and the other for small RNA through the TruSeq® Small RNA Library Prep kit, following the manufacturer’s instructions. For small RNA sequencing, unique molecular identifiers (UMIs) were introduced during the library preparation allowing error correction and increased accuracy during sequencing. Libraries were sequenced on a NextSeq500 instrument producing 150 bp pair-end fragments.

### 2.5 RNA extraction and RT-qPCR of early viral oncogene

RNA was extracted as described in Section 2.4. To exclude genomic DNA contamination, samples were treated with the RNase-free DNase I kit (Qiagen, Milan, Italy) according to the manufacturer’s instructions. Subsequently, 250 ng of purified RNA were reverse-transcribed into cDNA using the iScript cDNA Synthesis Kit (Bio-Rad, Hercules, CA, USA). RT-qPCR was then performed to assess the expression levels of the BPV-1 early oncogenes E5, E6, and E7 using the primer sets listed in Table 2. Amplification reactions were carried out on a CFX96™ Real-Time System using the SsoFast™ EvaGreen® Supermix 2x (Bio-Rad, Milan, Italy). Beta-2-Microglobulin (B2M) was used as a reference gene.

### 2.6 Bioinformatic analysis

Raw sequences were first checked for quality with FastQC (http://www.bioinformatics.babraham.ac.uk/projects/fastqc/) and trimmed from low-quality/adapter sequences using Trim Galore 0.6.6 software (https://www.bioinformatics.babraham.ac.uk/projects/trim_galore/). Trimmed reads were used for the alignment procedure and downstream analysis, which differed for mRNAs and small RNAs.

#### 2.6.1 From reads to genes

For smallRNAs libraries were prepared with UMIs, to accurately account for PCR duplicates and enhance quantification precision. The *umitools* package was used to process these data (Smith et al., 2017). Following UMI extraction the reads were used to perform a two-step alignment: first, on miRBase 22 hairpin (horse) (Kozomara et al., 2019), to recover micro RNA (miRNA) information; second, the unmapped reads from the previous step, on EquCab3.0 (Kalbfleisch et al., 2018) genome, to recover additional information on miRNAs and on the other small RNA typologies. The Bowtie2 algorithm with very sensitive local flag (Langmead and Salzberg, 2012) was used as aligner (Ziemann et al., 2016). After alignment steps, reads were deduplicated based on UMIs. Uniquely mapped reads were used for downstream analysis: for miRNAs from miRBase, a homemade script was applied for counting, while reads aligned to the genome were counted through FeatureCounts (Liao et al., 2014) using the horse annotation (Equus_caballus - Ensembl genes 109) and obtaining four count matrices (miRNAs, protein-coding RNAs, lncRNAs and all the other as miscellaneous RNAs). Last, the two miRNA matrices (miRBase and genome) were merged to build a unique count matrix for miRNAs.

For mRNAs, the STAR algorithm (Dobin et al., 2013) was used to align reads to the reference genome EquCab3.0. The data generated from the alignment were then used to identify the genes expressed in our samples using the horse annotation (Equus_caballus - Ensembl genes 109), while their expression level was assessed by counting reads uniquely aligned with the FeatureCounts software.

#### 2.6.2 Retriving gene expression differences between sarcoids and controls

The count matrices were imported into R environment and a preliminary exploratory analysis was carried out applying the hierarchical clustering *hclust* function of the “stats” package (https://rdocumentation.org/packages/stats/versions/3.6.2) and performing a principal component analysis (PCA) through the *plotPCA* function in DESeq2 package (Love et al., 2014). The latter was also used to identify differentially expressed miRNAs and genes (DEGs) between samples derived from sarcoids (T) and those derived from margin(M)/healthy(X) tissues (considered as control group). The R package ggplot2 (Wickham, 2016) was used to produce volcano plots. Genes/miRNAs were considered differentially expressed (T vs. M/X) if they had a |log2FoldChange| (|log2FC|) > 1 and an adjusted p-value (FDR) < 0.05 and were then subjected to the functional analysis.

#### 2.6.3 Functional analysis

For DEGs, the Cytoscape (Shannon et al., 2003) suite was used to construct a protein-protein interaction network (PPI) using the STRING application (Doncheva et al., 2019), which also allows for enrichment analysis by Gene Ontology (GO) categories (Biological Processes and Molecular Functions) and KEGG pathways.

For miRNAs, the top upregulated and downregulated (|log2FC| > 2) were divided into two lists and used separately for target retrieval. First, the most represented form, in terms of the sequences of each miRNA, was used as a reference to search for the corresponding human miRNA on the mirWalk3.0 database (Sticht et al., 2018). Then, predicted and validated target genes were identified, also specifying the site of action of the miRNA: 3′UTR, 5′UTR or CDS (coding region). To limit the extent of the analysis while keeping it informative, target genes common to the majority (at least 20%) of the input miRNAs were selected (keeping divided the targets of upregulated and downregulated miRNAs) and crossed with one-to-one human orthologues of DEGs (retrieved through the BioMart tool of Ensembl database, https://www.ensembl.org/biomart/martview/305776dd82c7aeb43d22c2e342dbfee5). In this way, we were able to identify DEGs that were also targets of DE miRNAs, selecting the miRNA-DEGs couples with opposite expression differences (upregulated miRNAs–downregulated target gene; downregulated miRNAs–upregulated target gene). This criterion was used to identify interactions consistent with the canonical miRNA-mediated post-transcriptional repression mechanism, in which miRNAs generally reduce target gene expression through mRNA destabilization and/or translational repression. However, these opposite trends were not interpreted as proof of direct causality, but rather as a filtering strategy to select biologically plausible candidate regulatory pairs for downstream KEGG pathway enrichment analysis. At last, DEGs from these selected couples were used for a KEGG pathway enrichment analysis.

The two mainly interesting enriched KEGG pathways are highlighted based on KEGG graph.

### 2.7 Chimeric transcript analysis and validation

Detection of chimeric transcripts was performed using Arriba 2.4.0 after alignment with STAR 2.7.10b, guided by Ensembl 109 annotation. False-positive fusion genes were filtered with a custom python script constructed following Arriba best practices (Uhrig et al., 2021; https://github.com/suhrig/arriba/tree/master/documentation, accessed 09/2025). Fusion events were investigated through qRT-PCR designing primers aimed at verifying the existance of the chimeric sequence (Table 3). RNA from all the samples was retrotranscribed to cDNA using iScript cDNA Synthesis Kit (Bio-Rad, Hercules, CA, USA) according to the manufacturer’s instructions. Quantitative PCR was performed using the SsoFast EvaGreen Supermix (Bio-Rad) on a CFX96 Real-Time PCR Detection System (Bio-Rad). Each 20 µL reaction contained 10 µL of SsoFast EvaGreen Supermix, 0.8 µL of forward primer (10 µM), 0.8 µL of reverse primer (10 µM), 2 µL of cDNA template, and nuclease-free water to a final volume of 20 µL. Thermal cycling conditions were as follows: initial denaturation at 95 °C for 30 s, followed by 40 cycles of 95 °C for 5 s and 60 °C for 20 s. Relative gene expression was calculated using the 2^-ΔΔCt method, normalizing target gene expression to the reference gene (B2M).

**TABLE 3:** Primer sequences used for the detection of chimeric transcripts. Beta-2-Microglobulin (B2M) was used as the reference gene.

Chimeric transcript	Forward primer (5’ → 3′)	Reverse primer (5’ → 3′)
WNT10B – FKB11	GGAGTCGGGAGCAGGAA	CAGGATGGTGGTCTTTCC
PFDN1 – ENSECAG00000056784	AGAATTGGAACAGAAAAAATCC	AGAATTGGAACAGAAAAAATCC
REV3L – FYN	CGGAGACGGCTTTTTACAAG	CGGAGACGGCTTTTTACAAG
ENSECAG00000015509 – LSP1	GAAGAGCTGCTGGTGGAAG	GAAGAGCTGCTGGTGGAAG
TMEM135 – ENSECAG00000050932	CTTCCAGCCTCCAGAACT	CTTCCAGCCTCCAGAACT
ENSECAG00000055570 – SLC16A1	AGCCGCGCATAACGATA	AGCCGCGCATAACGATA

## 3 Results

### 3.1 Histopathological characterization

In all 12 cases, histopathological diagnosis confirmed a spindle cell neoplasm, characterized by mild to moderate anysocytosis and anysokariosis within the cellular neoplastic population.

Nine out of 12 cases (75%) where characterized by the presence of rete pegs, whereas only in 3/12 cases (25%), a “picket fence” arrangement of neoplastic fibroblasts was highlighted in the subepithelial areas (Supplementary Figure S1). Three cases did not show picket fence arrangement nor rete pegs (one of these three cases was diffusely ulcerated, not allowing the evaluation of this histological feature). One case was characterized by the presence of “crown cells”, which are frequently observed in perivascular tumors.

### 3.2 DNA and RNA extraction and PCR assay for BPV and early oncogenes detection

Eighteen (Tura et al., 2022) out of 19 samples were positive for BPV1 DNA, while no positivity was found for BPV2 and BPV13 DNA. Of the enrolled animals, only T1 and T5 had peritumor margins that were not infiltrated by neoplastic tissue; both were positive for BPV1. Of the ten cases used as controls, 9/10 (90%) were negative for BPV1, BPV2 and BPV13; one sample was positive for BPV1 (Table 4). RT-qPCR analysis of BPV1 early oncogenes demonstrated significantly increased E5 expression in tumour tissues compared with non-tumour samples (Mann–Whitney test, p < 0.05). In contrast, E6 and E7 transcripts were detected exclusively in tumour tissues and showed comparatively limited variability among cases (Supplementary Table 1).

**TABLE 4:** Real Time qPCR results: data are expressed as Mean ± Standard Deviation (SD).

Sample	B2M (mean ± SD)	BPV1 (mean ± SD)	BPV2 (mean ± SD)	BPV13 (mean ± SD)
T1	20.95 ± 0.02	19.17 ± 0.79	40.00	40.00
T2	23.67 ± 0.80	19.20 ± 0.80	40.00	40.00
T3	24.37 ± 0.21	18.88 ± 0.18	40.00	40.00
T4	22.86 ± 1.05	19.99 ± 0.14	40.00	40.00
T5	22.89 ± 0.91	18.30 ± 0.80	40.00	40.00
T6	24.48 ± 0.48	40.00	40.00	40.00
T7	27.58 ± 0.37	24.25 ± 0.04	40.00	40.00
T8	22.04 ± 0.85	15.51 ± 0.38	40.00	40.00
T9	25.40 ± 0.01	19.08 ± 0.20	40.00	40.00
T10	25.62 ± 0.41	18.27 ± 0.27	40.00	40.00
T11	24.27 ± 0.63	22.78 ± 0.33	40.00	40.00
T12	25.84 ± 0.52	18.18 ± 0.03	40.00	40.00
T13	25.68 ± 0.25	24.00 ± 0.47	40.00	40.00
T14	23.70 ± 0.13	17.30 ± 0.31	40.00	40.00
T15	25.41 ± 0.22	20.00 ± 0.42	40.00	40.00
M1	24.49 ± 0.04	28.09 ± 0.02	40.00	40.00
M5	24.05 ± 0.06	26.44 ± 0.08	40.00	40.00
X1	24.26 ± 0.37	27.97 ± 0.20	40.00	40.00
X2	23.53 ± 0.11	40.00	40.00	40.00
X3	24.55 ± 0.09	40.00	40.00	40.00
X4	24.95 ± 0.14	40.00	40.00	40.00
X5	25.06 ± 0.08	40.00	40.00	40.00
X6	24.23 ± 0.09	40.00	40.00	40.00
X7	23.93 ± 0.26	40.00	40.00	40.00
X8	24.15 ± 0.10	40.00	40.00	40.00
X9	24.89 ± 0.10	40.00	40.00	40.00
X10	24.72 ± 0.05	40.00	40.00	40.00

Notably, sample T6 was negative for BPV1, BPV2, and BPV13 DNA using the qPCR assay applied in this study, whereas one healthy skin sample was BPV1-positive. However, the subsequent unsupervised analyses of both mRNA and small RNA data showed that T6 clustered with the sarcoid group, while the BPV1-positive healthy skin clustered with the other control tissues. This observation supported the retention of T6 in the tumour group as a histologically and transcriptomically sarcoid-like sample, while its BPV-negative status was explicitly considered as a diagnostic limitation.

### 3.3 Sequencing results

All samples were subjected to RNA and small RNA sequencing regardless of their viral DNA positivity/negativity. The experimental group assignment was then verified during the bioinformatics analysis of the sequencing data based on clustering resulting from the principal component analysis (PCA). Except for sample T12, which was excluded from the RNA-seq data due to library preparation failure, both data types revealed clear clustering of edge samples with the other control group samples, which were therefore considered as such. Moreover, the healthy tissue positive for BPV1 (X1) still clustered with the other healthy samples and was classified as a control, while the sarcoid negative for viral DNA (T6) exhibited a similar behavior to the other sarcoids and was thus retained in the tumor group. The sequencing of small RNAs produced an average of over 20 million reads per sample, which were first filtered out by eliminating those of poor quality and then aligned to the microRNA database (miRbase-22) to obtain the most in-depth information for this type of small RNA (Supplementary Table S2). 33% of cleaned reads were uniquely aligned to miRBase, and another 45% kept from the genome mapping. For mRNA sequencing, many sequences were generated for each sample, averaging over 63 million, of which the vast majority (over 89%) were suitable for downstream analysis (uniquely mapped), following clean-up of poor-quality sequences (Supplementary Table S3). The T12 sarcoid sample was subjected to two sequencing runs due to the poor quality of reads generated from the first run (hereafter referred to as T12_old). Unfortunately, the second sequencing (T12_new) also showed issues: although the total number of reads was comparable to that of the other samples, a high number of sequences (over four million) failed to align to the reference genome. Exploratory analysis of the count distribution (Supplementary Figure S2A) further confirmed the poor quality of the T12 sample in both sequencing attempts, leading to its exclusion from downstream analyses. Moreover, a discrepancy between the sarcoid sample T3 and the other samples belonging to the same experimental group was revealed (only in RNA-Seq). Despite samples belonging to the same experimental group (sarcoid group (T) and control group (X) and margins (M)) clustered within each other, the dendrogram (Supplementary Figure S2B) shows T3 closer to the control group samples. From both the heatmap analysis of correlations (Supplementary Figure S2C) and the principal components analysis (PCA) carried out on the first 500 features (Supplementary Figure S2D), intra-group similarities are highlighted, confirming greater homogeneity between the control samples with respect to the sarcoids and the sample T3 as an outlier, differing markedly from all the other samples. We also excluded this sample from the downstream analysis only in RNA-seq.

**FIGURE 1:**
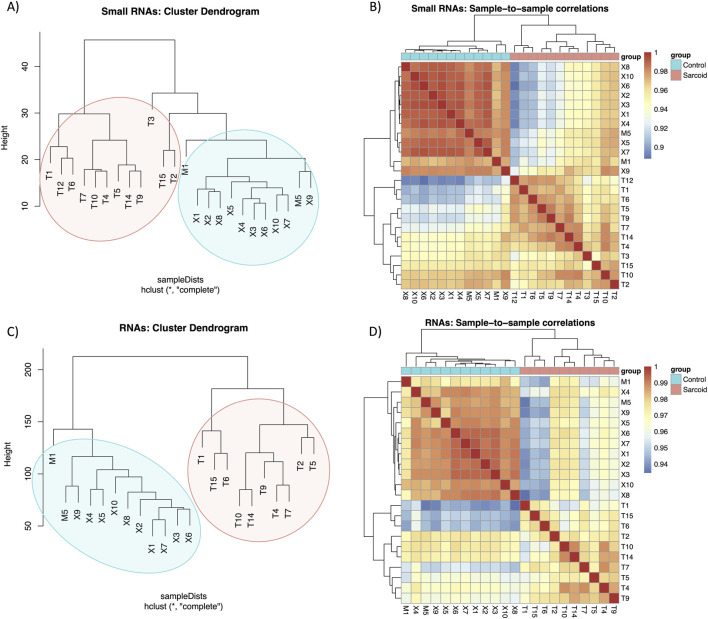
Cluster analysis: dendrograms of sample clusters for small RNA **(A)** and mRNA **(C)**, showing the clustering of margins (M1 and M5) with controls (X); heatmap of the correlations between the samples for small RNA **(B)** and mRNA **(D)**, indicating a correlation coefficient close to one between controls and differences within sarcoids.

By repeating the exploratory analysis after the exclusion of the above-mentioned samples, it is possible to observe a clear division between the two experimental groups, confirming the excellent quality of the selected dataset (Figures 1A,B). The same result was obtained for small RNA data (Figures 1C,D). In general, both dendrograms highlight the proximity of the two margins (M1 and M5) with control healthy skins (X), while the heatmaps show a high intra-group correlation, which was lower for sarcoid samples, highlighting inhomogeneity with the formation of several clusters.

**FIGURE 2:**
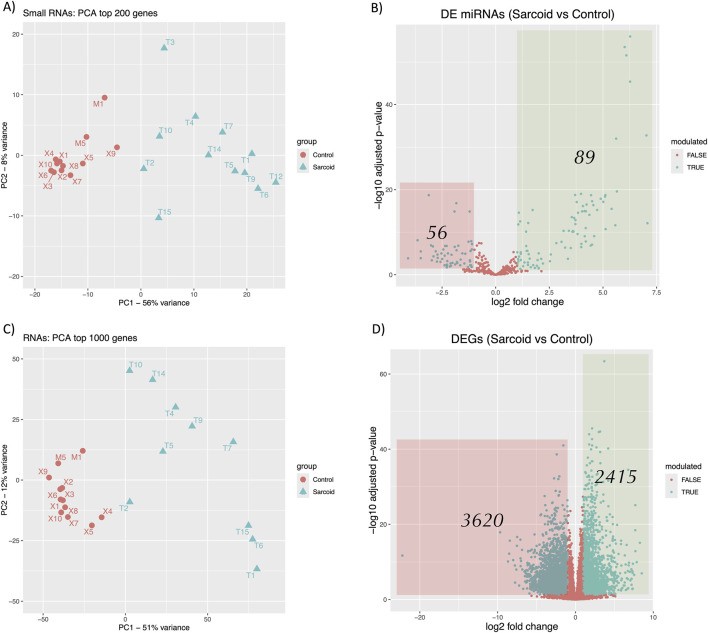
Assessing the difference between sarcoids and controls. Principal component analysis performed on the top 200 and 1,000 genes for small RNA **(A)** and mRNA **(C)**, respectively. Volcano plots representing statistically significant (log2FoldChange > |1| and an adjusted p-value <0.05) DE miRNA **(B)** and DEGs **(D)** (turquoise dots); red boxes enclose downregulated features, while green boxes the upregulated ones.

These results were confirmed by the principal component analysis carried out on the first 200 and 1,000 features (Figures 2A,C) for small RNA and mRNA, respectively. The two groups are perfectly divided with respect to the first component explaining over 50% of the variance, with control samples being closer.

### 3.4 Differential expression analysis

The differential expression analysis revealed 145 differentially expressed miRNAs (log2FoldChange > |1| and an adjusted p-value <0.05), with 56 downregulated and 89 upregulated miRNAs in sarcoids compared to controls (Figure 2B). For mRNA, over 6K DEGs emerged, 3,620 downregulated and 2,415 upregulated (Figure 2D) in sarcoids vs. controls.

Supplementary Tables S4, S5 report the complete lists of DE miRNAs and DEGs, while in Table 5 the top 20 upregulated and downregulated genes are reported in sarcoids compared to controls.

**TABLE 5:** Top 20 upregulated and downregulated genes in sarcoids compared to controls.

	Gene name	log2FoldChange	p-value	FDR
Upregulated genes in sarcoids	*ETV1*	3.70	1.78E-68	3.97E-64
	*HSP90B1*	2.14	2.63E-50	2.94E-46
	*RCN1*	3.16	2.74E-49	2.04E-45
	*GPX8*	2.99	5.06E-49	2.83E-45
	*PPIB*	1.76	2.43E-48	1.08E-44
	*PDIA6*	2.13	5.70E-47	2.12E-43
	*PRDX4*	1.77	1.73E-45	5.54E-42
	*TUSC3*	2.14	4.10E-45	1.02E-41
	*OLFML3*	2.70	1.52E-44	3.39E-41
	*LUM*	4.20	3.16E-44	6.43E-41
	*B3GNT9*	2.01	6.02E-44	1.12E-40
	*ZMAT3*	3.18	3.42E-43	5.87E-40
	*ERP29*	1.59	1.24E-41	1.85E-38
	*OSTC*	2.10	1.20E-40	1.67E-37
	*GPR141*	2.17	1.30E-40	1.71E-37
	*PDIA4*	1.85	4.46E-40	5.54E-37
	*GPX7*	3.20	4.80E-40	5.64E-37
	*SEC61A1*	1.46	1.79E-39	2.00E-36
	*TXNDC5*	2.04	4.97E-38	5.05E-35
	*KDELR2*	1.26	1.34E-37	1.30E-34
Downregulated genes in sarcoids	*ITPRID2*	−1.56	4.00E-45	1.02E-41
	*SGSM1*	−2.40	1.56E-42	2.50E-39
	*PPP1R12B*	−2.07	2.24E-36	1.79E-33
	*FBXL22*	−2.74	8.40E-36	5.87E-33
	*PIEZO2*	−2.20	1.37E-35	9.31E-33
	*FOXC1*	−2.54	2.92E-32	1.28E-29
	*PELI2*	−2.01	4.92E-32	2.11E-29
	*HID1*	−2.17	6.13E-31	2.36E-28
	*ISM1*	−3.96	8.10E-31	3.07E-28
	*TANC1*	−1.03	1.36E-29	4.05E-27
	*CLDN5*	−2.94	5.90E-29	1.65E-26
	*ID4*	−2.98	7.97E-29	2.20E-26
	*RYR2*	−3.87	3.14E-28	8.25E-26
	*TMEM164*	−1.65	3.25E-28	8.45E-26
	*PPT2*	−1.03	6.43E-28	1.63E-25
	*PRR36*	−2.52	1.68E-27	3.96E-25
	*HES4*	−2.25	1.98E-27	4.60E-25
	*EFHD1*	−2.67	4.68E-27	1.08E-24
	*AGO4*	−1.43	5.22E-27	1.19E-24
	*RORC*	−3.70	8.88E-27	2.00E-24

### 3.5 Functional analysis for differentially expressed miRNA

**FIGURE 3:**
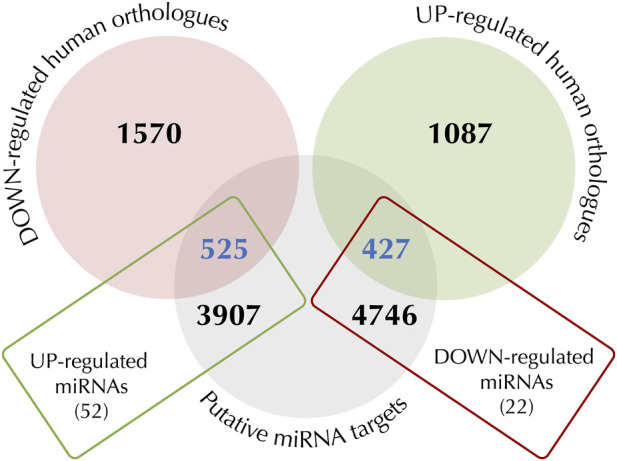
Schematic representation of the strategy used to identify differentially expressed genes (DEGs) that are also targets of differentially expressed (DE) miRNAs, and that were selected for functional analysis. DEGs shown in blue represent miRNA–mRNA pairs with opposite expression trends (i.e., upregulated miRNAs paired with downregulated target genes, and *vice versa*) consistent with canonical miRNA-mediated repression, and were prioritized for KEGG pathway enrichment analysis. The red circle includes human orthologues of genes found to be downregulated in sarcoids compared to controls, while the green circle includes upregulated orthologues. The grey circles enclose the putative targets (retrieved from miRWalk) of at least 20% of DE miRNAs. Rectangles indicate DE miRNAs: green for upregulated and red for downregulated miRNAs.

To retrieve up and downregulated miRNAs targets for the downstream functional analysis, a selection was made according to the criteria described in Figure 3. In brief, top up and downregulated miRNAs (log2FC > |2|, Table 6) were chosen as input for miRWalk software to retrieve the putative (validated and not validated) targets which were then selected for the number of miRNA hits.

**TABLE 6:** Top DE miRNAs used to retrieve human orthologues to be submitted on miRWalk for target identification.

	miRNA	log2FoldChange	p-value	FDR
Upregulated miRNAs in sarcoids	eca-mir-337	7.07	7.96E-14	7.71E-13
	eca-mir-503	7.03	2.37E-35	1.75E-33
	eca-mir-424	6.27	2.66E-59	9.77E-57
	eca-mir-542	6.09	2.20E-54	2.70E-52
	eca-mir-450a	6.01	1.62E-56	2.98E-54
	eca-mir-431	5.65	4.94E-22	2.60E-20
	eca-mir-450b	5.61	1.64E-34	1.01E-32
	eca-mir-541	5.57	2.66E-13	2.39E-12
	eca-mir-134	5.40	1.97E-17	3.29E-16
	eca-mir-487a	5.12	2.23E-12	1.91E-11
	eca-mir-376b	5.11	6.12E-21	1.96E-19
	eca-mir-1185	5.09	1.52E-20	4.30E-19
	eca-mir-376a	5.01	3.18E-19	7.31E-18
	eca-mir-493b	4.89	5.28E-20	1.39E-18
	eca-mir-381	4.73	3.79E-17	6.06E-16
	eca-mir-485	4.59	1.16E-10	8.41E-10
	eca-mir-432	4.55	2.32E-18	4.28E-17
	eca-mir-376c	4.49	2.15E-19	5.27E-18
	eca-mir-655	4.49	1.44E-08	8.41E-08
	eca-mir-409	4.44	6.39E-21	1.96E-19
	eca-mir-1193	4.43	2.46E-08	1.39E-07
	eca-mir-127	4.37	7.40E-16	8.51E-15
	eca-mir-299	4.23	1.60E-18	3.10E-17
	eca-mir-487b	4.20	8.78E-17	1.24E-15
	eca-mir-410	4.20	2.28E-09	1.52E-08
	eca-mir-370	4.19	1.21E-12	1.06E-11
	eca-mir-329a	4.15	1.26E-10	8.92E-10
	eca-mir-379	4.14	7.43E-17	1.09E-15
	eca-mir-369	4.07	4.12E-18	7.22E-17
	eca-mir-411	4.02	9.63E-17	1.31E-15
	eca-mir-495	3.99	9.33E-09	5.63E-08
	eca-mir-377	3.99	6.35E-08	3.20E-07
	eca-mir-889	3.98	2.15E-21	9.91E-20
	eca-mir-494	3.84	2.31E-16	2.74E-15
	eca-mir-154a	3.81	3.74E-11	2.75E-10
	eca-mir-323	3.78	3.77E-19	8.16E-18
	eca-mir-382	3.71	1.31E-13	1.23E-12
	eca-mir-136	3.65	4.10E-15	4.44E-14
	eca-mir-758	3.62	9.65E-12	7.40E-11
	eca-mir-539	3.60	6.81E-12	5.45E-11
	eca-mir-543	3.57	1.03E-11	7.71E-11
	eca-mir-544-2	3.50	1.06E-07	5.21E-07
	eca-mir-433	3.41	5.66E-12	4.73E-11
	eca-mir-380	3.36	5.51E-05	1.81E-04
	eca-mir-412	3.16	5.93E-12	4.85E-11
	eca-mir-656	3.07	2.85E-09	1.84E-08
	eca-mir-496	2.83	5.89E-09	3.67E-08
	eca-mir-92b	2.57	4.50E-06	1.72E-05
	eca-mir-1197	2.53	2.34E-04	6.74E-04
	eca-mir-615	2.21	9.59E-04	2.34E-03
	eca-mir-218-1	2.12	5.78E-05	1.87E-04
	eca-mir-216b	2.00	2.35E-06	9.61E-06
Downregulated miRNAs in sarcoids	eca-mir-486	−2.11	9.02E-04	2.24E-03
	eca-mir-488	−2.13	5.33E-03	1.10E-02
	eca-mir-95	−2.22	2.43E-06	9.81E-06
	eca-mir-205	−2.27	3.86E-03	8.35E-03
	eca-mir-216a	−2.27	1.87E-03	4.31E-03
	eca-mir-1248	−2.28	3.23E-08	1.72E-07
	eca-mir-451	−2.38	2.63E-03	5.86E-03
	eca-mir-184	−2.38	9.18E-06	3.38E-05
	eca-mir-708	−2.44	2.46E-07	1.17E-06
	eca-mir-141	−2.49	6.32E-05	2.00E-04
	eca-mir-135b	−2.53	2.47E-06	9.87E-06
	eca-mir-96	−2.71	4.90E-06	1.86E-05
	eca-mir-200c	−2.71	3.21E-05	1.10E-04
	eca-mir-200b	−2.74	3.10E-04	8.58E-04
	eca-mir-182	−2.80	6.93E-07	3.07E-06
	eca-mir-135a-2	−2.93	4.27E-08	2.21E-07
	eca-mir-1291a	−2.98	1.40E-04	4.14E-04
	eca-mir-183	−3.01	2.22E-08	1.27E-07
	eca-mir-204b-2	−3.12	6.35E-21	1.96E-19
	eca-mir-375	−3.49	3.85E-05	1.30E-04
	eca-mir-489	−3.49	7.62E-07	3.34E-06
	eca-mir-653	−4.09	4.13E-05	1.38E-04

### 3.6 Functional analysis for *DEGs*

A functional analysis for enriched vocabularies of Gene Ontology (GO), “biological processes” and “molecular function”, was performed for DEGs using STRINGdb software in the Cytoscape suite. Enriched biological processes found for upregulated genes in the sarcoid group compared to the control group are reported in Table 7, while Table 8 shows those for downregulated genes. Results for molecular function are reported in Supplementary Tables S6, S7.

**TABLE 7:** Enriched “Biological Processes” for genes found to be upregulated in the sarcoid group compared to the control group.

Go term category	FDR	Background genes	UP-regulated genes
Collagen fibril organization	2.40E-14	38	14
Regulation of multicellular organismal process	8.22E-11	2566	58
Anatomical structure morphogenesis	1.30E-10	1829	47
Response to organic substance	1.90E-08	2152	47
Regulation of response to stimulus	1.81E-06	3258	57
Tube development	2.97E-06	700	25
Locomotion	3.00E-06	1001	28
Collagen metabolic process	1.25E-05	49	9
Regulation of protein metabolic process	2.02E-05	2442	46
Regulation of cell migration	2.80E-05	710	22
Cell adhesion	6.37E-05	771	24
Skeletal system development	1.02E-04	382	17
Vasculature development	1.02E-04	434	18
Reg. of transmembrane receptor protein serine/threonine kinase signaling path	1.02E-04	198	13
Regulation of cell population proliferation	3.79E-04	1232	29
Chondrocyte development	4.21E-04	36	7
Inflammatory response	5.75E-04	386	16
Negative regulation of developmental process	7.93E-04	768	22
Response to wounding	8.10E-04	265	12
Collagen biosynthetic process	1.20E-03	8	4
Regulation of response to external stimulus	1.74E-03	672	20
Reproductive structure development	1.74E-03	316	14
Ossification	1.80E-03	191	10
Enzyme linked receptor protein signaling pathway	1.84E-03	486	17
Regulation of angiogenesis	1.91E-03	223	12
Cellular component organization	2.20E-03	4673	59
Extracellular matrix assembly	2.60E-03	27	5
Negative regulation of wound healing	3.00E-03	52	6
Cellular response to endogenous stimulus	3.30E-03	854	20
Regulation of cell adhesion	3.70E-03	570	16
Response to external stimulus	3.84E-03	1843	34
Tissue morphogenesis	4.03E-03	461	16
Negative regulation of signal transduction	4.70E-03	960	21
Positive regulation of cell differentiation	5.00E-03	809	19
Protein maturation	5.00E-03	175	9
Positive regulation of nervous system development	5.20E-03	458	14
Positive regulation of protein phosphorylation	5.53E-03	809	21
Response to stress	5.53E-03	2744	43
Regulation of ERK1 and ERK2 cascade	5.60E-03	228	10
Regulation of intracellular signal transduction	5.60E-03	1310	25
Transmembrane receptor protein serine/threonine kinase signaling pathway	6.30E-03	137	8
Epithelial cell proliferation	6.92E-03	62	7
Regulation of body fluid levels	8.10E-03	240	10
Negative regulation of reproductive process	9.20E-03	39	5
Nervous system development	9.90E-03	1921	31

**TABLE 8:** Enriched “Biological Processes” for genes found to be downregulated in the sarcoid group compared to the control group.

Go term category	FDR	Background genes	Down regulated genes
Multicellular organism development	3.87E-14	4228	449
Regulation of biological quality	7.77E-12	3271	357
Ion transport	5.04E-09	1244	166
Cell adhesion	1.65E-07	771	114
Regulation of ion transport	2.05E-07	528	88
Neurogenesis	2.08E-07	1428	176
Cell-cell signaling	7.30E-07	753	106
Anatomical structure morphogenesis	7.90E-07	1829	204
Modulation of chemical synaptic transmission	7.90E-07	355	64
Regulation of membrane potential	1.96E-06	349	65
Cell development	9.65E-06	1392	165
Skin development	1.62E-05	220	47
Regulation of multicellular organismal process	1.72E-05	2566	264
Tissue development	5.23E-05	1375	160
Chemical homeostasis	1.27E-04	880	113
Regulation of system process	1.36E-04	390	64
Lipid metabolic process	1.63E-04	982	122
Behavior	3.62E-04	440	68
Multicellular organismal water homeostasis	1.10E-03	37	15
Blood circulation	1.30E-03	246	42
Trans-synaptic signaling	1.50E-03	341	52
Regulation of cell development	1.60E-03	811	97
Regulation of transmembrane transporter activity	2.20E-03	190	35
Regulation of synaptic plasticity	2.40E-03	157	31
Localization	2.40E-03	4640	403
Cell junction organization	2.52E-03	399	61
Positive regulation of synaptic transmission	2.70E-03	110	25
Animal organ morphogenesis	2.83E-03	780	98
Cellular component morphogenesis	2.83E-03	500	71
Regulation of cell communication	2.90E-03	2755	257
Multicellular organismal homeostasis	2.90E-03	247	41
Regulation of hormone levels	3.10E-03	343	51
Axon ensheathment	3.90E-03	98	23
Cell-cell adhesion via plasma-membrane adhesion molecules	4.46E-03	189	37
Regulation of nervous system process	4.57E-03	103	26
Regulation of localization	4.70E-03	2250	215
Positive regulation of developmental process	4.70E-03	1126	122
Olefinic compound metabolic process	4.70E-03	57	17
Divalent inorganic cation homeostasis	4.90E-03	372	53
Adenylate cyclase-modulating G protein-coupled receptor signaling pathway	5.40E-03	193	34
Glial cell differentiation	5.68E-03	135	30
Central nervous system development	5.97E-03	733	92
Cell surface receptor signaling pathway	6.45E-03	1682	175
Positive regulation of ion transport	6.60E-03	178	32
Multicellular organismal signaling	6.70E-03	88	21
Tissue morphogenesis	7.10E-03	461	61
Epithelial cell differentiation	7.98E-03	458	65
Unsaturated fatty acid metabolic process	7.98E-03	72	21
Regulation of body fluid levels	8.04E-03	240	42

### 3.7 Functional analysis for differentially expressed (miRNA-*DEG* couples) according to the expression levels

The putative targets of at least the 20% of input miRNAs were crossed with human orthologues corresponding to our DEGs, identifying miRNA-DEG couples (with opposite expression trends in sarcoids vs. controls) used for the functional analysis (Supplementary Tables S8, S9).

The strategy used to identify differentially expressed genes (DEGs) that are also targets of differentially expressed (DE) miRNAs was to select miRNA–mRNA pairs with opposite expression trends (i.e., upregulated miRNAs paired with downregulated target genes, and *vice versa*). These were used for a KEGG pathway enrichment analysis. The statistically significant enriched KEGG pathways for the selected DEGs are reported in Table 9.

**TABLE 9:** Enriched KEGG pathways for selected DEGs from the miRNA-DEG couples according to expression changes in sarcoids.

Functional grup	Term	p-value	FDR	Associated genes found
One carbon pool by folate	One carbon pool by folate	3.25E-03	1.92E-02	*ALDH1L1, ALDH1L2, MTHFD1L, MTHFD2, SHMT2*
ABC transporters	ABC transporters	2.22E-03	1.39E-02	*ABCA4, ABCA5, ABCA9, ABCC11, ABCC6, ABCC8, ABCG1, CFTR*
Protein digestion and absorption	Protein digestion and absorption	6.41E-06	1.04E-04	*ATP1A1, ATP1A4, COL11A1, COL12A1, COL14A1, COL16A1, COL19A1, COL22A1, COL4A1, COL4A3, COL4A5, COL4A6, COL5A2, COL6A3, COL6A6, COL8A2, PRCP, SLC8A3*
Mineral absorption	Mineral absorption	4.01E-03	2.17E-02	*ATP1A1, ATP1A4, ATP2B2, ATP7B, CYBRD1, SLC5A1, SLC8A3, STEAP2, TF*
Cholesterol metabolism	Cholesterol metabolism	4.96E-03	2.55E-02	*ANGPTL4, CYP27A1, LRP1, LRP2, LRPAP1, PLTP, SCARB1, SORT1*
Pathways in cancer	Pathways in cancer	1.05E-07	2.94E-06	*ADCY5, ADCY6, ADCY7, ALK, APC2, AR, AXIN2, CALML4, CAMK2A, CAMK2B, CDH1, CDK6, COL4A1, COL4A3, COL4A5, COL4A6, CSF3R, CTNNA3, DAPK2, DCC, E2F3, EGF, ERBB2, FGF1, FGFR4, FLT4, FN1, FZD3, FZD6, GLI1, IFNAR1, IFNAR2, IGF2, IL23R, ITGA2, ITGB1, JAG1, KIT, LAMA2, LAMB1, LAMB3, LAMC1, MECOM, MET, NOTCH1, PAX8, PLCB4, PTCH1, RASGRP1, RASGRP2, RUNX1T1, TGFB3, TGFBR1, TRAF5, WNT5A, WNT9B, ZBTB16*
Proteoglycans in cancer	Proteoglycans in cancer	9.00E-05	9.75E-04	*ANK2, ANK3, CAMK2A, CAMK2B, CD63, CTSL, ERBB2, ERBB3, ERBB4, FN1, FZD3, FZD6, GPC3, HCLS1, IGF2, ITGA2, ITGB1, KDR, MET, PLCE1, PTCH1, TIAM1, VAV1, WNT5A, WNT9B*
Rap1 signaling pathway	Rap1 signaling pathway	8.37E-04	6.80E-03	*ADCY5, ADCY6, ADCY7, CALML4, CDH1, EGF, FGF1, FGFR4, FLT4, GRIN2B, ITGB1, KDR, KIT, MAGI1, MAGI2, MET, PDGFC, PLCB4, PLCE1, RAPGEF4, RASGRP2, TIAM1, VAV1*
Calcium signaling pathway	Calcium signaling pathway	4.41E-08	1.72E-06	*ADCY7, ADRA1A, ASPH, ATP2B2, CACNA1D, CACNA1E, CACNA1H, CALML4, CAMK2A, CAMK2B, CAMK4, EGF, ERBB2, ERBB3, ERBB4, FGF1, FGFR4, FLT4, GNAL, GRIN2B, ITPKB, KDR, MET, MST1R, NOS1, NTRK2, NTRK3, ORAI2, P2RX5, PDGFC, PLCB4, PLCE1, RYR2, RYR3, SLC8A3, TACR1*
Axon guidance	Axon guidance	7.44E-11	7.25E-09	*ABLIM1, ABLIM2, CAMK2A, CAMK2B, DCC, EPHA1, EPHA3, EPHA8, EPHB1, FES, FZD3, GDF7, ITGB1, L1CAM, MET, NTN4, NTNG1, PAK5, PLXNA4, PLXNB1, PLXNB3, PLXNC1, PTCH1, ROBO1, ROBO2, SEMA4A, SEMA4G, SEMA5A, SLIT2, SLIT3, SRGAP1, SRGAP3, TRPC4, WNT5A*
Hippo signaling pathway	Hippo signaling pathway	1.76E-03	1.27E-02	*AJUBA, APC2, AXIN2, CDH1, CTNNA3, DLG4, FGF1, FRMD1, FZD3, FZD6, GDF7, LLGL2, NKD1, RASSF6, TGFB3, TGFBR1, WNT5A, WNT9B*
Cell adhesion molecules	Cell adhesion molecules	8.67E-06	1.21E-04	*CADM3, CD86, CD99, CD99L2, CDH1, CDH3, CDH4, ITGB1, L1CAM, MPZL1, NECTIN1, NEGR1, NFASC, NLGN1, NRCAM, NRXN1, NRXN3, NTNG1, PTPRD, PTPRF, PTPRS, SIGLEC1, VCAN*
Adherens junction	Adherens junction	3.52E-03	2.02E-02	*BAIAP2, CDH1, CTNNA3, ERBB2, LMO7, MET, NECTIN1, NECTIN4, PTPRB, PTPRF, SORBS1, TGFBR1*
Regulation of actin cytoskeleton	Regulation of actin cytoskeleton	3.83E-03	2.14E-02	*ABI2, APC2, BAIAP2, C6, DIAPH3, EGF, FGF1, FGFR4, FN1, ITGA11, ITGA2, ITGA7, ITGB1, ITGB4, MYH10, MYH11, PAK5, PDGFC, PIP5K1B, SCIN, SPATA13, TIAM1, VAV1*
PPAR signaling pathway	PPAR signaling pathway	1.84E-03	1.28E-02	*ACOX1, ACSL1, ACSL5, ANGPTL4, CPT1B, CYP27A1, HMGCS1, PLTP, PPARA, SCD, SORBS1*
MAPK signaling pathway	MAPK signaling pathway	1.80E-04	1.76E-03	*CACNA1D, CACNA1E, CACNA1H, CACNA2D4, CACNB2, CACNB4, CACNG5, DUSP6, EGF, ERBB2, ERBB3, ERBB4, FGF1, FGFR4, FLT4, IGF2, KDR, KIT, MAP3K9, MAP4K2, MAP4K4, MAPT, MECOM, MET, NTRK2, PDGFC, PLA2G4F, PTPRR, RASGRP1, RASGRP2, TGFB3, TGFBR1*
Dilated cardiomyopathy	Cardiac muscle contraction	5.96E-03	2.83E-02	*ASPH, ATP1A1, ATP1A4, CACNA1D, CACNA2D4, CACNB2, CACNB4, CACNG5, MYH6, RYR2, SLC8A3*
	Adrenergic signaling in cardiomyocytes	1.62E-06	3.15E-05	*ADCY5, ADCY6, ADCY7, ADRA1A, ATP1A1, ATP1A4, ATP2B2, CACNA1D, CACNA2D4, CACNB2, CACNB4, CACNG5, CALML4, CAMK2A, CAMK2B, CREB3L2, MYH6, PLCB4, RAPGEF4, RYR2, SCN4B, SCN5A, SCN7A, SLC8A3*
	Oxytocin signaling pathway	5.23E-03	2.61E-02	*ADCY5, ADCY6, ADCY7, CACNA1D, CACNA2D4, CACNB2, CACNB4, CACNG5, CALML4, CAMK2A, CAMK2B, CAMK4, GUCY1A2, PLA2G4F, PLCB4, RYR2, RYR3*
	Hypertrophic cardiomyopathy	8.39E-07	1.82E-05	*CACNA1D, CACNA2D4, CACNB2, CACNB4, CACNG5, DMD, ITGA11, ITGA2, ITGA7, ITGB1, ITGB4, LAMA2, MYH6, RYR2, SGCD, SLC8A3, TGFB3, TTN*
	Arrhythmogenic right ventricular cardiomyopathy	2.01E-06	3.57E-05	*CACNA1D, CACNA2D4, CACNB2, CACNB4, CACNG5, CTNNA3, DMD, ITGA11, ITGA2, ITGA7, ITGB1, ITGB4, LAMA2, RYR2, SGCD, SLC8A3*
	Dilated cardiomyopathy	1.94E-08	1.26E-06	*ADCY5, ADCY6, ADCY7, CACNA1D, CACNA2D4, CACNB2, CACNB4, CACNG5, DMD, ITGA11, ITGA2, ITGA7, ITGB1, ITGB4, LAMA2, MYH6, RYR2, SGCD, SLC8A3, TGFB3, TTN*
ECM-receptor interaction	PI3K-Akt signaling pathway	3.82E-07	9.32E-06	*CDK6, COL4A1, COL4A3, COL4A5, COL4A6, COL6A3, COL6A6, CREB3L2, CSF3R, EGF, ERBB2, ERBB3, ERBB4, FGF1, FGFR4, FLT4, FN1, IFNAR1, IFNAR2, IGF2, ITGA11, ITGA2, ITGA7, ITGB1, ITGB4, KDR, KIT, LAMA2, LAMB1, LAMB3, LAMB4, LAMC1, MAGI1, MAGI2, MET, NTRK2, PDGFC, PRLR, RELN, THBS4, TNR, TNXB, VWF*
	Focal adhesion	2.18E-08	1.06E-06	*COL4A1, COL4A3, COL4A5, COL4A6, COL6A3, COL6A6, EGF, EMP2, ERBB2, FLT4, FN1, ITGA11, ITGA2, ITGA7, ITGB1, ITGB4, KDR, LAMA2, LAMB1, LAMB3, LAMB4, LAMC1, MET, PAK5, PDGFC, PIP5K1B, RELN, THBS4, TNR, TNXB, VAV1, VWF*
	ECM-receptor interaction	2.33E-12	4.55E-10	*AGRN, COL4A1, COL4A3, COL4A5, COL4A6, COL6A3, COL6A6, FN1, FRAS1, FREM2, ITGA11, ITGA2, ITGA7, ITGB1, ITGB4, LAMA2, LAMB1, LAMB3, LAMB4, LAMC1, RELN, THBS4, TNR, TNXB, VWF*
	AGE-RAGE signaling pathway in diabetic complications	6.35E-03	2.95E-02	*COL4A1, COL4A3, COL4A5, COL4A6, CYBB, FN1, NOX1, NOX4, PLCB4, PLCE1, TGFB3, TGFBR1*
	Amoebiasis	9.12E-04	7.11E-03	*COL4A1, COL4A3, COL4A5, COL4A6, FN1, GNAL, IL1R2, LAMA2, LAMB1, LAMB3, LAMB4, LAMC1, PLCB4, TGFB3*
	Human papillomavirus infection	6.85E-06	1.03E-04	*APC2, ATP6V0A4, AXIN2, CDK6, COL4A1, COL4A3, COL4A5, COL4A6, COL6A3, COL6A6, CREB3L2, EGF, FN1, FZD3, FZD6, IFNAR1, IFNAR2, ITGA11, ITGA2, ITGA7, ITGB1, ITGB4, JAG1, LAMA2, LAMB1, LAMB3, LAMB4, LAMC1, LLGL2, MAGI1, NOTCH1, RELN, THBS4, TNR, TNXB, VWF, WNT5A, WNT9B*
	Small cell lung cancer	8.67E-05	9.95E-04	*CDK6, COL4A1, COL4A3, COL4A5, COL4A6, E2F3, FN1, ITGA2, ITGB1, LAMA2, LAMB1, LAMB3, LAMB4, LAMC1, TRAF5*
Insulin secretion	cGMP-PKG signaling pathway	1.40E-03	1.05E-02	*ADCY5, ADCY6, ADCY7, ADRA1A, ATP1A1, ATP1A4, ATP2B2, CACNA1D, CALML4, CREB3L2, GTF2IRD1, GUCY1A2, IRS4, KCNMA1, KCNU1, MYH6, OPRD1, PLCB4, SLC8A3*
	cAMP signaling pathway	2.40E-05	3.12E-04	*ACOX1, ADCY5, ADCY6, ADCY7, ATP1A1, ATP1A4, ATP2B2, CACNA1D, CALML4, CAMK2A, CAMK2B, CAMK4, CFTR, CREB3L2, GLI1, GLP1R, GRIA1, GRIN2B, PDE10A, PDE4C, PDE4D, PLCE1, PPARA, PTCH1, RAPGEF4, RYR2, TIAM1, VAV1*
	Cardiac muscle contraction	5.96E-03	2.83E-02	*ASPH, ATP1A1, ATP1A4, CACNA1D, CACNA2D4, CACNB2, CACNB4, CACNG5, MYH6, RYR2, SLC8A3*
	Adrenergic signaling in cardiomyocytes	1.62E-06	3.15E-05	*ADCY5, ADCY6, ADCY7, ADRA1A, ATP1A1, ATP1A4, ATP2B2, CACNA1D, CACNA2D4, CACNB2, CACNB4, CACNG5, CALML4, CAMK2A, CAMK2B, CREB3L2, MYH6, PLCB4, RAPGEF4, RYR2, SCN4B, SCN5A, SCN7A, SLC8A3*
	Circadian entrainment	1.61E-04	1.65E-03	*ADCY5, ADCY6, ADCY7, CACNA1D, CACNA1H, CALML4, CAMK2A, CAMK2B, GRIA1, GRIN2B, GUCY1A2, NOS1, PLCB4, RYR2, RYR3*
	Glutamatergic synapse	4.75E-03	2.50E-02	*ADCY5, ADCY6, ADCY7, CACNA1D, DLG4, GRIA1, GRIK3, GRIN2B, PLA2G4F, PLCB4, SHANK1, SHANK2, SLC1A2, SLC1A3*
	Insulin secretion	7.85E-08	2.55E-06	*ABCC8, ADCY5, ADCY6, ADCY7, ATP1A1, ATP1A4, CACNA1D, CAMK2A, CAMK2B, CREB3L2, GLP1R, KCNMA1, KCNN3, KCNU1, PCLO, PLCB4, RAPGEF4, RIMS2, RYR2*
	Melanogenesis	8.25E-04	7.00E-03	*ADCY5, ADCY6, ADCY7, CALML4, CAMK2A, CAMK2B, CREB3L2, DCT, FZD3, FZD6, KIT, PLCB4, WNT5A, WNT9B*
	Thyroid hormone synthesis	5.89E-03	2.87E-02	*ADCY5, ADCY6, ADCY7, ATP1A1, ATP1A4, CREB3L2, DUOX2, LRP2, PAX8, PLCB4*
	Oxytocin signaling pathway	5.23E-03	2.61E-02	*ADCY5, ADCY6, ADCY7, CACNA1D, CACNA2D4, CACNB2, CACNB4, CACNG5, CALML4, CAMK2A, CAMK2B, CAMK4, GUCY1A2, PLA2G4F, PLCB4, RYR2, RYR3*
	Aldosterone synthesis and secretion	4.98E-05	6.07E-04	*ADCY5, ADCY6, ADCY7, ATP1A1, ATP1A4, ATP2B2, CACNA1D, CACNA1H, CALML4, CAMK2A, CAMK2B, CAMK4, CREB3L2, KCNK3, PLCB4, SCARB1*
	Cortisol synthesis and secretion	2.04E-03	1.37E-02	*ADCY5, ADCY6, ADCY7, CACNA1D, CACNA1H, CREB3L2, KCNA4, KCNK3, PLCB4, SCARB1*
	Cushing syndrome	1.86E-04	1.73E-03	*ADCY5, ADCY6, ADCY7, APC2, AXIN2, CACNA1D, CACNA1H, CAMK2A, CAMK2B, CDK6, CREB3L2, E2F3, FZD3, FZD6, KCNA4, KCNK3, PLCB4, SCARB1, WNT5A, WNT9B*
	Salivary secretion	3.51E-04	3.12E-03	*ADCY5, ADCY6, ADCY7, ADRA1A, ATP1A1, ATP1A4, ATP2B2, CALML4, GUCY1A2, KCNMA1, LPO, NOS1, PLCB4, RYR3*
	Gastric acid secretion	2.05E-03	1.33E-02	*ADCY5, ADCY6, ADCY7, ATP1A1, ATP1A4, CALML4, CAMK2A, CAMK2B, CFTR, KCNK10, PLCB4*
	Bile secretion	7.07E-03	3.21E-02	*ADCY5, ADCY6, ADCY7, AQP4, ATP1A1, ATP1A4, CFTR, SCARB1, SLC4A5, SLC5A1, SLCO1A2*
	Amphetamine addiction	3.20E-03	1.95E-02	*ADCY5, CACNA1D, CALML4, CAMK2A, CAMK2B, CAMK4, CREB3L2, DDC, GRIA1, GRIN2B*
	Dilated cardiomyopathy	1.94E-08	1.26E-06	*ADCY5, ADCY6, ADCY7, CACNA1D, CACNA2D4, CACNB2, CACNB4, CACNG5, DMD, ITGA11, ITGA2, ITGA7, ITGB1, ITGB4, LAMA2, MYH6, RYR2, SGCD, SLC8A3, TGFB3, TTN*

**FIGURE 4:**
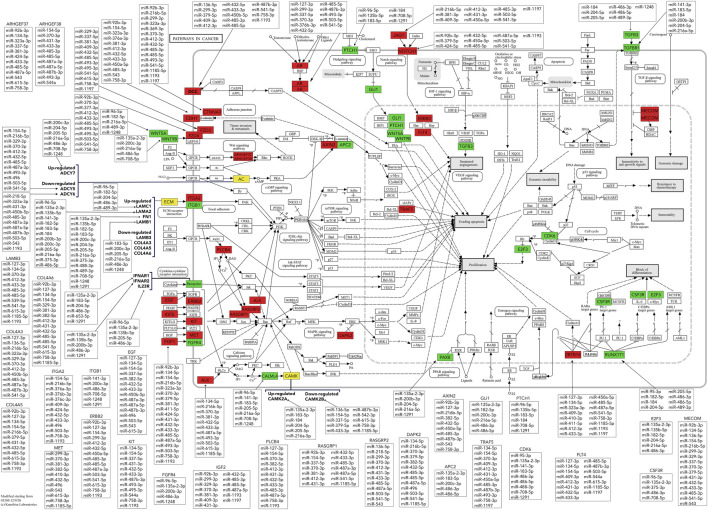
“Pathways in cancer” illustration highlighting DEGs (red for downregulated, green for upregulated and yellow when isoforms with an opposite trend of expression are present) and reporting related DE miRNAs (with an opposite expression modulation with respect to the mRNA). Figure modified starting from Kanehisa Laboratories KEGG map. Regular map notation is available at https://www.kegg.jp/kegg/document/help_pathway.html.

**FIGURE 5:**
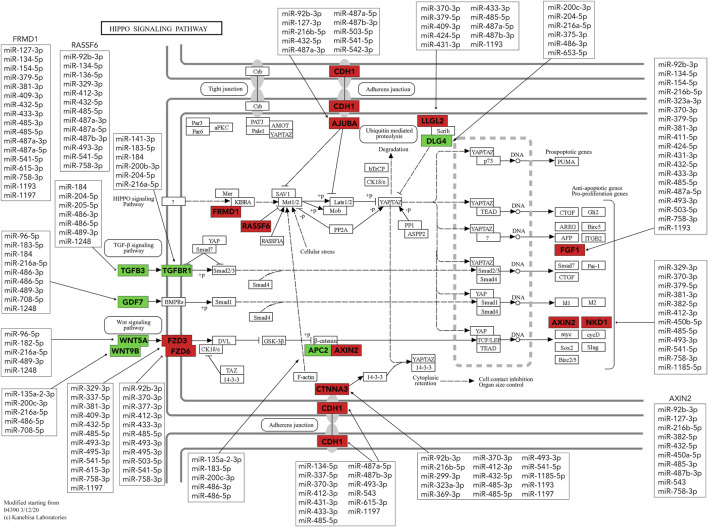
“Hippo signaling pathway” illustration highlighting DEGs (red for downregulated and green for upregulated) and reporting related DE miRNAs (with an opposite expression modulation with respect to the mRNA). Figure modified starting from Kanehisa Laboratories KEGG map. Regular map notation is available at https://www.kegg.jp/kegg/document/help_pathway.html.

Figures 4, 5 illustrate two of the mainly interesting enriched KEGG pathways based on KEGG graphs.

### 3.8 Chimeric transcript detection

**FIGURE 6:**
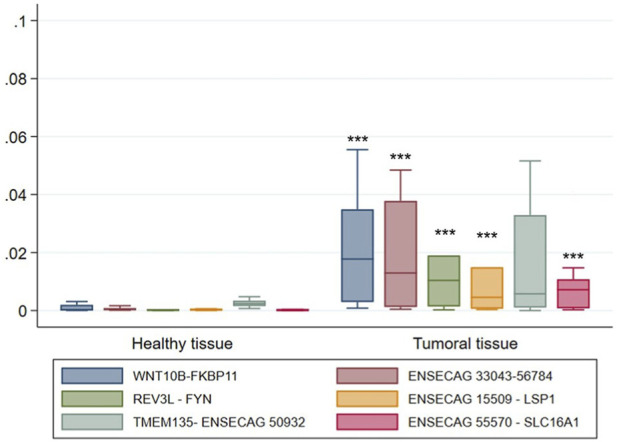
Relative expression levels of target genes in equine sarcoid and healty samples normalized to B2M. Data are presented as 2^–ΔΔCq values.

Computational analysis identified 237 potential fusion events classified in Supplementary Table S10. However, since horse has no annotation pointing out common fusion tanscipts, the filtering reduced this number to six candidates. Validation through real-time PCR confirmed *in silico* analysis showing that five out of the six fusion transcripts were overexpressed (overall mean increase of approximately 19.5-fold) in tumor tissues (p < 0.001) (Figure 6).

## 4 Discussions

In this study, a deep RNA and miRNA seq analysis was conducted on 12 sarcoid tissue samples, two paired skin samples from histologically healthy margins, and 10 healthy skin samples harvested from healthy horses. We set up a Real-Time PCR protocol for the detection of BPV 1, 2, and 13 with a high degree of sensitivity and specificity which allowed us to evaluate the positivity of samples to the most common type of bovine papillomavirus (BPV1).

As expected, BPV infection was demonstrated in most of the examined cases. In particular, the only viral type present was BPV1, with invariable negativity for BPV2 and BPV13, which have been reported in Brazil, Austria, New Zealand and also other areas of Italy (Munday et al., 2021).

The retention of sample T6 deserves specific consideration. This sample was negative for BPV1, BPV2, and BPV13 DNA by qPCR, but showed histopathological features consistent with equine sarcoid and clustered with the other sarcoid samples in both mRNA and small RNA transcriptomic analyses. Although BPV detection represents an important diagnostic support for equine sarcoid, the available literature indicates that BPV PCR results should be interpreted in an integrated clinicopathological context (Epperson and Castleman, 2017; Martano et al., 2025; Munday et al., 2021). Munday et al. reported that no causative papillomavirus DNA was detectable in a subset of equine sarcoids, raising the possibility that some cases may involve papillomavirus types not covered by the assays used, low viral copy number, or other technical/biological factors affecting detection. Therefore, we retained T6 in the sarcoid group because its global molecular phenotype was consistent with the sarcoid samples and clearly distinct from healthy skin and tumour-free margin tissues. Nevertheless, we acknowledge that the absence of detectable BPV DNA in this sample represents a limitation, and T6 should be regarded as a diagnostically challenging BPV-negative sarcoid-like lesion within the present cohort.

Interestingly, RT-qPCR analysis demonstrated significantly higher expression of the BPV early gene E5 in tumour tissues compared with non-tumour samples, whereas E6 and E7 transcripts were not detected in non-tumour tissues and showed comparatively limited variability among tumour samples. Moreover, non-tumour margins clustered together with healthy tissues according to the DEG profile, suggesting that the transcriptional alterations identified in this study are specifically associated with the tumoural state rather than with viral presence alone.

Although direct mechanistic interactions between BPV early genes and specific host DEGs or miRNAs were not investigated here, these findings support the hypothesis that persistent viral oncogene activity may contribute to shaping the transcriptional landscape of equine sarcoids.

Regarding the RNA sequencing results, we aimed to explore the deep characterization of the sarcoid cell transcriptome by integrating data from both small-RNA and mRNA sequencing. To this end, we performed a differential analysis of both transcriptomes, as detailed in the Materials and Methods section. Subsequently, we performed an analysis incorporating differentially expressed miRNAs, their predicted target genes, and differentially expressed mRNAs, creating miRNA-DEG pairs (Supplementary Tables S8, S9). This approach enabled a functional analysis of the modulated pathways across the entire expressed transcriptome.

The identification of upregulated and downregulated genes, along with their regulatory non-coding elements, highlighted pathways closely associated with tumor progression, as summarized in our enriched KEGG pathways for selected DEGs from the miRNA-DEG couples analysis (Table 9).

The differential expression analysis of miRNAs in tumor tissues compared to healthy skin revealed many well-known oncomiRs, both up- and downregulated. Nine out of the 20 most upregulated miRNA and seven out of the 20 most downregulated miRNA were also identified by Pawlina and collaborators (Pawlina et al., 2017) in the only study, to our knowledge, on miRNA dysregulation in equine sarcoids (Pawlina et al., 2017).

For instance, miR-450a, which results upregulated in both studies, is similarly upregulated in human oral squamous cell carcinoma, where it is likely involved in promoting cell motility, a process essential for cancer invasion (Hsing et al., 2019). Hence, also in sarcoids, despite their embryological differences with squamous cell carcinomas, miR-450a might act as an onco-miRNA, with its upregulation contributing to local invasiveness (Hsing et al., 2015).

Another shared result is a significant downregulation of miR-200b and miR-141, both part of the miR-200 miRNA family. A similar result has been reported in some human virus-related cancers, such as Epstein-Barr virus-induced gastric carcinoma and HPV-induced cervical cancer (Shinozaki et al., 2010; Hu et al., 2010). Generally, the downregulation of miR-200 family members is associated with increased cell invasiveness and, overall, reduced survival.

We also identified uniquely modulated miRNAs not previously reported in sarcoids. Some of these miRNAs can have dual roles in tumor progression, invasion, and malignancy—functions that may vary depending on the cellular and neoplastic context. MiR-337, miR-503, miR-542, and miR-431-5p have all been shown to act both as tumor suppressors or promoters in different neoplasms.

More in detail, miR-337-3p - the most highly expressed miRNA in our study-has been previously reported as overexpressed in human liposarcomas, a tumor that shares a mesenchymal origin, similarly to equine sarcoids (Peter et al., 2016). On the other hand, miR-337-3p was downregulated both in tumor and serum of patients with osteosarcoma, with levels reverting to normal after excisional surgery (Dey et al., 2025). It is thought to inhibit migration and invasion in breast cancer while promoting apoptosis (Pan et al., 2021). Additionally, miR-337 targets *PIK3CA* and *PIK3CB*, thereby reducing PI3K/AKT signaling activation (Zhang et al., 2020).

The second and third most upregulated miRNAs in our study, respectively miR-503 and miR-427, have also been identified as tumor suppressors in several malignancies, but they have also been reported to exhibit oncogenic functions in specific cancer types, suggesting a tissue- or disease-specific role (Wu et al., 2020). Notably, miR-424 and miR-503, both members of the miR-16 family, are clustered on the same chromosome in humans and horses. These miRNAs are often dysregulated in cancers and play crucial yet paradoxical roles in tumor initiation and progression by targeting different genes and molecular pathways. Furthermore, these miRNAs are often co-expressed in cancer cells, indicating a potential coordinated function as a cluster (Wu et al., 2020).

Another interesting observation was the strong upregulation of miR-542. A similar result has been observed in human osteosarcoma, both in cell cultures and neoplastic tissue, where it is recognized as a cellular proliferation promoter, and as a circulating biomarker. A higher miR-542-3p concentration has been indeed associated with advanced tumor stage and short overall survival (Li et al., 2018; Cheng et al., 2015). MiR-542 regulates multiple cancer-related behaviors like cell apoptosis, metastasis, proliferation, cell cycle, and glycolysis, through the targeting of at least 18 genes and key signaling pathways such as Wnt/β-catenin, ERK1/2, JAK2, and PI3K/AKT (Alshahrani et al., 2023). Additional future studies may help to evaluate the prognostic relevance of this circulating miRNA, especially in relation to recurrence in equine sarcoids.

Similarly, miR-431-5p, which was also upregulated in our study, has been implicated in various cancers, regulating processes such as proliferation, apoptosis, autophagy, migration, invasion, and angiogenesis (Kong et al., 2018).

Taken together, these results might reflect the unique nature of sarcoids, which are locally aggressive but non-metastatic and support the hypothesis that a tumor-specific profile of mi-RNA expression, with different roles in tumorigenesis, tumor biology and phenotyping should be better described.

The analyses of differential mRNA expression between sarcoid and healthy skin also revealed substantial differences in both gene expression and active pathways. Genes involved in phosphorylation (*PPP1R12B, PELI2*), cell adhesion (*CLDN5*), virus-host interactions (*HSP90B1*, *PDIA6, PPIB, AGO4*), cell differentiation (*FOXC1, ID4, ETV1, RORC*), actin-cytoskeleton organization with lamellipodium assembly (*PPP1R12B, PIEZO2, TANC1*), and cellular movement (*HSP90B1, PPP1R12B, PIEZO2, TANC1, RYR2*) were significantly altered (Table 4). HSP90B1 acts as a molecular chaperone, facilitating the folding of viral proteins and modulating host immune responses, *PDIA6* is involved in protein folding interacting with viral proteins, *PPIB* has been implicated in viral replication processes, *AGO4* contribute to antiviral defense. Of extreme interest, since we are dealing with a mesenchymal neoplasm, is also the modulation of *SMAD2* and *HIF1AN*, suggesting a complex regulatory system in the tumor microenvironment.

Notably, enriched KEGG pathways for selected DEGs from the miRNA-DEG couples analysis included critical processes such as “Pathways in Cancer” (Figure 4) and the “Hippo signaling pathway” (Figure 5). While this selection strategy does not exclude positively correlated miRNA–mRNA pairs or indirect/non-canonical regulatory mechanisms, it allowed us to prioritize candidate interactions consistent with canonical miRNA-mediated repression, which should be interpreted as putative regulatory relationships requiring experimental validation. In this context, a plethora of key regulators of tissue invasion and metastasis modulated in our sarcoids samples are outlined in cancer-related pathways (Figure 4). Of particular interest in papillomavirus-related cancers in horses is the modulation of the alternative Wnt signaling pathway (Mecocci et al., 2021), which seems involved also in sarcoids. Indeed, WNT5a and WNT9b genes were likely upregulated due to the activation of the Wnt/β-catenin-independent pathway. A crucial protein in this alternative Wnt signaling pathway is YAP/TAZ, recently identified as part of the Wnt-YAP/TAZ signal transduction mechanism (Yang et al., 2021). This complex can promote the expression of Notch receptors and ligands, further integrating multiple signaling pathways. Interestingly, Wnt5a/b ligands serve as both upstream activators and downstream targets of the YAP/TAZ-TEAD complex, suggesting a potential positive feedback loop that stimulates the other particularly modulated and interesting pathway in our sarcoids, the Hippo signaling pathway (Li and Guan, 2022).

The Hippo signaling pathway, detailed in Figure 5, is an evolutionarily conserved signaling cascade initially identified in *Drosophila melanogaster* for its role in restricting tissue overgrowth (Ma et al., 2019). In mammals, it regulates cell proliferation, differentiation, apoptosis, organ size control, and tissue homeostasis (Yang et al., 2021). Hippo signaling pathway dysregulation is linked to several diseases, including cancer, as it interferes with upstream regulatory mechanisms that are normally controlled by a complex interplay of intrinsic and extrinsic signals—such as mechanical stress, cell–cell contact, polarity, energy levels, cellular stress, and various diffusible hormonal factors, many of which signal through G protein–coupled receptors (Xiao and Dong, 2021).

The Hippo signaling pathway exerts its effects through its core effectors, YAP/TAZ. When the pathway is inactive, unphosphorylated YAP/TAZ accumulates in the nucleus, where they interact with TEAD transcription factors to drive gene expression. Conversely, when the Hippo signaling pathway is active, YAP/TAZ are phosphorylated by LATS1/2 leading to their sequestration in the cytoplasm. In our study, several downregulated miRNAs (miR-200c-3p, miR-204-5p, miR-216a-5p, miR-375-3p, miR-486-3p, miR-653-5p) target and modulate *DLG4* gene, that encodes signaling protein known to suppress Hippo signaling pathway activation by inhibiting YAP/TAZ phosphorylation (Yang et al., 2021).

Moreover, our results indicate that LATS1/2 activity may have been enhanced through miRNA-mediated repression of *AJUBA* gene, a negative regulator of Hippo signaling pathway, and its upstream inhibitor, *RASSF6* (Figure 5). Given that Hippo signaling pathway components interact with other key signaling pathways such as Wnt, AMPK, TGF-β, and Notch, its role in cancer progression is highly complex (Yang et al., 2021).

Furthermore, our study revealed that *FZD3* and *FZD6* genes were downregulated due to the upregulation of several miRNAs on this pathway (Figure 5). Frizzled receptors (FZDs) regulate both the canonical β-catenin pathway and various non-canonical β-catenin-independent pathways. Aberrant FZD signaling is implicated in numerous diseases, including cancer, although the role of non-canonical Wnt pathways can play either tumor-promoting or tumor-suppressing roles.

Interestingly, we observed both Hippo activation and repression signals, suggesting a dynamic balance between tumor proliferation, infiltration, and malignancy—reflecting the unique non-metastatic behavior of sarcoid tumors.

The cGAS-STING pathway, which triggers the innate immune system in presence of pathogens or of damaged cells through NF-κB and IRF3, leading to the production of type I interferons (IFN-I) and pro-inflammatory cytokines (Zhou et al., 2023), can deeply influence the tumor microenvironment (TME). In our study, we observed a significant modulation of the Hippo pathway, which aligns with literature findings that link cGAS-STING signaling to PV infection. A functional connection between cGAS-STING and the Hippo pathway has been previously observed in PV-induced tumors, where PV exploits the E6/E7 oncoproteins to deactivate Hippo signaling (Pa et al., 2024). This promotes YAP/TAZ activity, which, in turn, suppresses cGAS-STING, reducing interferon production and supporting immune evasion, thereby blocking the innate immune response and promoting tumor transformation (Tolbert et al., 2024).

Although the Hippo signaling pathway has been extensively studied *in vitro* as a potential therapeutic target, its *in vivo* roles remain less understood. In our study we demonstrated for the first time in horses, the downregulation of cGAS/STING signaling in sarcoids; this is an important finding that showed as equine BPV1-induced sarcoids have an immune evasion mechanism similar to high risk human PV (Bortnik et al., 2021; Zhou et al., 2019).

The Hippo signaling pathway plays a vital role in fibroblast activation, stem cell maintenance, ECM remodeling, immune infiltration, and angiogenesis. Through these processes, it modulates the secretion of molecules that impact tumor development *via* interactions with TME components. Increasing evidence suggests that the Hippo signaling pathway can exert both pro- and anti-tumor effects, depending on the tumor type and context (Yang et al., 2021).

In this type of cancer, the pathway appears to be uniquely activated, as it is linked to immune evasion, fibroblast activation, and increased cell mobility, while still being associated with low metastatic potential. Notably, in this study, we observed (for the first time to our knowledge) a marked overexpression of chimeric transcripts in sarcoid-affected tissues compared to healthy tissues, with an average increase of approximately 19.5-fold. After a thorough filtering procedure, a total of six fusion loci were identified, among which the most common was a transcript derived from the fusion of WNT10B and FKBP11. This event was found in six out of 10 tumor samples. WNT10B is a member of the WNT family activating the WNT/β-catenin signaling cascade and often associated with different forms of cancer (e.g., T-cell acute lymphoblastic leukemia) (Cassaro et al., 2021), while FKBP11 has been implicated in enhancing malignant properties of osteosarcoma and acting as a prognostic marker (Zeng et al., 2023). Although the precise functional role of this transcript in sarcoids remains to be elucidated, these data suggest a potential involvement in tumor progression. To our knowledge, this is the first report on the expression of chimeric transcripts in equine sarcoids, suggesting these events may represent a previously overlooked aspect of the biology of this neoplasm. Indeed, the available literature on sarcoids has focused primarily on the role of bovine papillomavirus (BPV) and the dysregulation of cellular genes it induces, while there are no specific data regarding gene fusion events. However, in other tumor contexts, both human and animal, chimeric transcripts have been widely documented as potential oncogenic factors or as markers of genomic instability (Liu et al., 2023; Nguyen et al., 2018). In human carcinomas associated with high-risk papillomavirus (HPV), for example, transcriptional alterations and splicing abnormalities contribute to tumor progression, suggesting that similar mechanisms may also operate in sarcoids. The high expression of chimeric transcripts in pathological samples suggests that these are not isolated or marginal events, but phenomena actively supported by the transcriptional program of the neoplastic cell. These findings, although preliminary in nature, open interesting prospects from both research and diagnostic perspectives, potentially allowing the identification of new biomarkers and a better understanding of the complex biology of tumor progression.

## 5 Conclusion

In this study, for the first time to the authors’ knowledge, both coding and non-coding transcript-regulated metabolic pathways were simultaneously investigated in equine sarcoids and overall, underscores the complex regulatory interplay between miRNAs, mRNAs, and key oncogenic pathways in sarcoid tumors, providing novel insights into their molecular pathogenesis. This comprehensive and effective global analysis enabled the identification of highly regulated pathways, providing deeper insight into the pathogenesis of a tumor with a peculiar behavior in terms of invasiveness and low metastatic potential, and paving the way for the identification of novel therapeutic targets.

Furthermore, a marked overexpression of chimeric transcripts in sarcoid tissues has also been observed, representing a previously overlooked aspect of sarcoid biology. These fusion transcripts may serve as tumor-specific biomarkers and potential therapeutic targets, warranting further investigation.

Overall, these findings broaden our understanding of the molecular mechanisms underlying equine sarcoids and introduce chimeric transcripts as a promising avenue for future research in comparative oncology.

## Data Availability

The datasets presented in this study can be found in online repositories. The names of the repository/repositories and accession number(s) can be found below: https://www.ncbi.nlm.nih.gov/, PRJNA1273441.
