# Supplementary material for: Integrated transcriptomic profiling reveals oncogenic pathways and chimeric transcripts in equine sarcoid lesions with predominant BPV1 detection
Source: Front Mol Biosci. 2026 Jun 19;13:1818241. doi: 10.3389/fmolb.2026.1818241 (PMC13328017; doi:10.3389/fmolb.2026.1818241)
Supplement: Supplementary file 1 [file DataSheet1.docx]

Supplementary Material

##
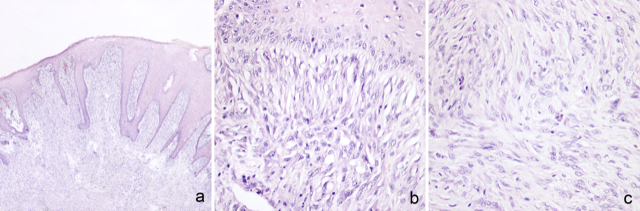


Supplementary Figure S1: a) The epidermis, when present, was moderately to severely hyperplastic, forming some projections into the underlying dermis, called “rete pegs” (H&E, 1.5x); b) under the epidermis, the neoplastic cells were often forming palisades, defined as “picket fence” aspect ” (H&E, 40x); c) the tumors where invariably represented by a proliferation of spindle cells, arranged in variably long bundles, usually supported by a mild to moderate amount of fibrovascular stroma (H&E, 40x).

Supplementary Figure S2. Exploratory analysis for mRNA-seq data: A) Count distribution's histogram for each sample showing T12 data high variability compared to the other samples; B) Dendrogram of sample clusters, sample T3 is closer to the samples belonging to the control group (X) and the margins (M); C) Correlations’ heatmap between samples; D) Principal components analysis.

Supplementary Table S1. BPV1 oncogene expression. Data are expressed as Cq mean value ± standard deviation.

| **Sample** | **BPV1-E5 (Cq ± SD)** | **BPV1-E6 (Cq ± SD)** | **BPV1-E7 (Cq ± SD)** | **B2M (Cq ± SD)** |
| --- | --- | --- | --- | --- |
| X1 | 31.4 ± 0.34 | – | – | 26.3 ± 0.31 |
| M1 | 33.3 ± 0.49 | – | – | 24.4 ± 0.41 |
| M5 | 29.6 ± 0.01 | – | – | 25.0 ± 0.01 |
| T1 | 21.6 ± 0.08 | 28.5 ± 0.08 | 28.1 ± 0.51 | 26.0 ± 0.01 |
| T2 | 20.3 ± 0.04 | 26.6 ± 0.05 | 27.0 ± 0.23 | 23.7 ± 0.01 |
| T3 | 20.7 ± 0.14 | 27.6 ± 0.02 | 27.7 ± 0.43 | 25.0 ± 0.12 |
| T4 | 20.3 ± 0.05 | 28.9 ± 0.17 | 27.7 ± 0.38 | 25.2 ± 0.02 |
| T5 | 19.4 ± 0.41 | 28.3 ± 0.04 | 27.3 ± 0.06 | 25.5 ± 0.42 |
| T6 | 19.9 ± 0.08 | 27.2 ± 0.13 | 27.8 ± 0.42 | 24.6 ± 0.02 |
| T9 | 20.7 ± 0.06 | 28.5 ± 0.07 | 29.2 ± 0.26 | 26.0 ± 0.02 |
| T10 | 21.6 ± 0.18 | 27.3 ± 0.27 | 28.0 ± 0.69 | 24.3 ± 0.12 |
| T11 | 24.6 ± 0.15 | 29.7 ± 0.31 | 29.7 ± 0.24 | 28.5 ± 0.12 |
| T14 | 21.2 ± 0.10 | 30.5 ± 0.15 | 29.4 ± 0.06 | 24.5 ± 0.12 |
| T15 | 21.5 ± 0.06 | 29.2 ± 0.01 | 27.9 ± 0.31 | 24.3 ± 0.02 |

Supplementary Table S2. Sequencing statistics for the small RNA library

| Sample | raw reads | reads after trimming | uniquely mapped reads on miRBase | % uniquely mapped reads on miRBase | uniquely mapped reads on genome | % uniquely mapped reads on genome |
| --- | --- | --- | --- | --- | --- | --- |
| X1 | 22,946,838 | 13,992,161 | 4,056,794 | 28.99 | 1,858,903 | 24.85 |
| X2 | 28,361,614 | 19,238,747 | 5,386,617 | 28.00 | 2,637,376 | 24.96 |
| X3 | 24,464,425 | 16,008,968 | 4,937,742 | 30.84 | 1,993,307 | 24.97 |
| X4 | 27,065,074 | 17,020,728 | 5,434,249 | 31.93 | 2,191,507 | 28.29 |
| X5 | 19,553,653 | 15,219,690 | 5,838,894 | 38.36 | 2,108,486 | 35.72 |
| X6 | 28,621,472 | 20,730,965 | 7,513,440 | 36.24 | 2,794,350 | 33.64 |
| X7 | 25,718,809 | 20,510,398 | 8,565,840 | 41.76 | 2,676,182 | 39.35 |
| X8 | 28,566,145 | 14,222,037 | 3,558,806 | 25.02 | 1,866,919 | 21.51 |
| X9 | 20,636,061 | 10,731,968 | 1,823,845 | 16.99 | 1,346,325 | 17.10 |
| X10 | 27,238,511 | 21,998,717 | 8,256,423 | 37.53 | 3,198,760 | 42.65 |
| M1 | 20,022,594 | 10,647,400 | 2,773,481 | 26.05 | 1,438,404 | 25.61 |
| M5 | 25,865,479 | 17,039,492 | 6,623,757 | 38.87 | 2,335,915 | 36.76 |
| T1 | 18,114,873 | 15,719,714 | 4,903,817 | 31.20 | 3,531,853 | 71.18 |
| T2 | 19,407,085 | 16,170,253 | 6,148,970 | 38.03 | 3,117,468 | 64.40 |
| T3 | 27,666,641 | 20,449,783 | 5,930,284 | 29.00 | 2,935,375 | 38.48 |
| T4 | 21,937,703 | 15,814,869 | 5,538,278 | 35.02 | 2,753,081 | 41.40 |
| T5 | 20,154,454 | 18,223,896 | 6,482,835 | 35.57 | 3,789,791 | 67.36 |
| T6 | 29,420,797 | 26,614,812 | 9,147,262 | 34.37 | 5,729,745 | 70.41 |
| T7 | 28,295,014 | 24,312,705 | 8,902,413 | 36.62 | 4,885,971 | 55.81 |
| T9 | 22,513,938 | 20,007,772 | 7,323,385 | 36.60 | 4,239,920 | 67.48 |
| T10 | 25,371,726 | 21,087,407 | 7,679,602 | 36.42 | 4,059,574 | 55.97 |
| T12 | 26,663,851 | 22,306,860 | 6,462,039 | 28.97 | 5,256,534 | 53.91 |
| T14 | 18,137,985 | 15,736,001 | 6,939,717 | 44.10 | 3,011,615 | 65.70 |
| T15 | 21,223,295 | 19,541,421 | 7,170,445 | 36.69 | 3,659,432 | 76.07 |
| Mean | 24,082,001.54 | 18,056,115.17 | 6,141,622.29 | 33.47 | 3,059,033.04 | 45.15 |

Supplementary Table S3. Sequencing statistics for the mRNA library

| **Sample** | **raw reads** | **reads after cleaning** | **uniquely mapped reads** | **% uniquely mapped reads** |
| --- | --- | --- | --- | --- |
| **X1** | 56,215,397 | 55,237,106 | 50,474,334 | 91.38 |
| **X2** | 64,604,127 | 63,206,606 | 57,376,405 | 90.78 |
| **X3** | 49,214,999 | 48,365,058 | 44,693,629 | 92.41 |
| **X4** | 56,222,786 | 55,496,936 | 51,501,411 | 92.80 |
| **X5** | 48,457,581 | 47,106,232 | 41,723,124 | 88.57 |
| **X6** | 69,219,598 | 68,089,349 | 62,452,562 | 91.72 |
| **X7** | 74,308,834 | 73,611,554 | 69,447,053 | 94.34 |
| **X8** | 51,342,135 | 50,156,655 | 44,884,497 | 89.49 |
| **X9** | 103,515,977 | 102,239,313 | 94,417,979 | 92.35 |
| **X10** | 83,526,757 | 82,691,835 | 75,508,660 | 91.31 |
| **M1** | 63,464,106 | 62,703,908 | 58,269,163 | 92.93 |
| **M5** | 57,680,830 | 56,989,352 | 52,137,681 | 91.49 |
| **T1** | 78,816,397 | 77,990,872 | 73,579,716 | 94.34 |
| **T2** | 80,024,619 | 79,292,775 | 75,637,640 | 95.39 |
| **T3** | 68,371,123 | 67,594,426 | 63,800,055 | 94.39 |
| **T4** | 62,064,697 | 61,441,043 | 58,746,056 | 95.61 |
| **T5** | 49,361,244 | 48,655,305 | 46,022,631 | 94.59 |
| **T6** | 55,877,759 | 55,150,853 | 52,457,143 | 95.12 |
| **T7** | 49,888,069 | 49,292,547 | 47,058,519 | 95.47 |
| **T9** | 67,624,599 | 66,950,983 | 63,755,723 | 95.23 |
| **T10** | 60,487,486 | 59,738,515 | 56,355,790 | 94.34 |
| **T12_old** | 40,478,281 | 37,547,515 | 18,306,000 | 48.75 |
| **T12_new** | 67,426,918 | 57,960,404 | 30,971,678 | 53.44 |
| **T14** | 50,124,767 | 49,147,420 | 46,011,376 | 93.62 |
| **T15** | 67,340,492 | 66,134,223 | 62279,156 | 94.17 |
| **Mean** | 63,026,383 | 61,711,631 | 55,914,719 | 89.76 |

Supplementary Table S4. Differentially expressed miRNAs in sarcoid vs control groups.

| miRNA | log2FoldChange | p-value | FDR |
| --- | --- | --- | --- |
| eca-mir-337 | 7.071777295 | 7.96E-14 | 7.71E-13 |
| eca-mir-503 | 7.02522599 | 2.37E-35 | 1.75E-33 |
| eca-mir-424 | 6.267462271 | 2.66E-59 | 9.77E-57 |
| eca-mir-450c | 6.265053572 | 4.56E-48 | 4.20E-46 |
| eca-mir-542 | 6.08942605 | 2.20E-54 | 2.70E-52 |
| eca-mir-450a | 6.010005388 | 1.62E-56 | 2.98E-54 |
| eca-mir-431 | 5.64709917 | 4.94E-22 | 2.60E-20 |
| eca-mir-450b | 5.61207343 | 1.64E-34 | 1.01E-32 |
| eca-mir-541 | 5.574062873 | 2.66E-13 | 2.39E-12 |
| eca-mir-134 | 5.401625707 | 1.97E-17 | 3.29E-16 |
| eca-mir-487a | 5.1244619 | 2.23E-12 | 1.91E-11 |
| eca-mir-376b | 5.111979955 | 6.12E-21 | 1.96E-19 |
| eca-mir-1185 | 5.09330345 | 1.52E-20 | 4.30E-19 |
| eca-mir-376a | 5.014099691 | 3.18E-19 | 7.31E-18 |
| eca-mir-493b | 4.894663121 | 5.28E-20 | 1.39E-18 |
| eca-mir-381 | 4.727298208 | 3.79E-17 | 6.06E-16 |
| eca-mir-485 | 4.590257726 | 1.16E-10 | 8.41E-10 |
| eca-mir-432 | 4.549476116 | 2.32E-18 | 4.28E-17 |
| eca-mir-376c | 4.494125722 | 2.15E-19 | 5.27E-18 |
| eca-mir-655 | 4.490523971 | 1.44E-08 | 8.41E-08 |
| eca-mir-409 | 4.444323215 | 6.39E-21 | 1.96E-19 |
| eca-mir-1193 | 4.427956132 | 2.46E-08 | 1.39E-07 |
| eca-mir-127 | 4.366854467 | 7.40E-16 | 8.51E-15 |
| eca-mir-299 | 4.233973052 | 1.60E-18 | 3.10E-17 |
| eca-mir-487b | 4.200348817 | 8.78E-17 | 1.24E-15 |
| eca-mir-410 | 4.197738471 | 2.28E-09 | 1.52E-08 |
| eca-mir-370 | 4.194469227 | 1.21E-12 | 1.06E-11 |
| eca-mir-329a | 4.148829988 | 1.26E-10 | 8.92E-10 |
| eca-mir-379 | 4.140313478 | 7.43E-17 | 1.09E-15 |
| eca-mir-369 | 4.065778882 | 4.12E-18 | 7.22E-17 |
| eca-mir-411 | 4.022969011 | 9.63E-17 | 1.31E-15 |
| eca-mir-495 | 3.991082968 | 9.33E-09 | 5.63E-08 |
| eca-mir-377 | 3.985604457 | 6.35E-08 | 3.20E-07 |
| eca-mir-889 | 3.975396464 | 2.15E-21 | 9.91E-20 |
| eca-mir-494 | 3.835620723 | 2.31E-16 | 2.74E-15 |
| eca-mir-154a | 3.813978165 | 3.74E-11 | 2.75E-10 |
| eca-mir-323 | 3.776810042 | 3.77E-19 | 8.16E-18 |
| eca-mir-154b | 3.715277242 | 1.32E-14 | 1.39E-13 |
| eca-mir-382 | 3.710913817 | 1.31E-13 | 1.23E-12 |
| eca-mir-3958 | 3.699631732 | 4.35E-21 | 1.78E-19 |
| eca-mir-136 | 3.652717377 | 4.10E-15 | 4.44E-14 |
| eca-mir-758 | 3.623406426 | 9.65E-12 | 7.40E-11 |
| eca-mir-539 | 3.600108833 | 6.81E-12 | 5.45E-11 |
| eca-mir-543 | 3.574945834 | 1.03E-11 | 7.71E-11 |
| eca-mir-3959 | 3.541273423 | 2.59E-15 | 2.88E-14 |
| eca-mir-544-2 | 3.496820497 | 1.06E-07 | 5.21E-07 |
| eca-mir-433 | 3.407745523 | 5.66E-12 | 4.73E-11 |
| eca-mir-380 | 3.363854494 | 5.51E-05 | 0.00018113 |
| eca-mir-329b | 3.233849878 | 8.62E-08 | 4.29E-07 |
| eca-mir-412 | 3.161701887 | 5.93E-12 | 4.85E-11 |
| eca-mir-656 | 3.065642046 | 2.85E-09 | 1.84E-08 |
| eca-mir-496 | 2.828231645 | 5.89E-09 | 3.67E-08 |
| eca-mir-92b | 2.572295298 | 4.50E-06 | 1.72E-05 |
| eca-mir-1197 | 2.531637332 | 0.00023437 | 0.0006738 |
| eca-mir-615 | 2.206059431 | 0.00095893 | 0.00233701 |
| eca-mir-218-1 | 2.123884487 | 5.78E-05 | 0.00018657 |
| eca-mir-763 | 2.006585152 | 0.0127568 | 0.02407438 |
| eca-mir-216b | 2.004181613 | 2.35E-06 | 9.61E-06 |
| eca-mir-9097 | 2.000642862 | 0.01865555 | 0.03316541 |
| eca-mir-7177b | 1.953633522 | 0.00012136 | 0.00036606 |
| eca-mir-301b | 1.921694984 | 0.00354599 | 0.00776741 |
| eca-mir-1597 | 1.82851355 | 0.01376168 | 0.02557726 |
| eca-mir-218-2 | 1.751020013 | 0.00246921 | 0.00557467 |
| eca-mir-671 | 1.721922162 | 3.95E-17 | 6.06E-16 |
| eca-mir-2114 | 1.636855769 | 0.00875493 | 0.01704379 |
| eca-mir-675 | 1.585329881 | 5.09E-05 | 0.00016861 |
| eca-mir-190a | 1.538466212 | 3.87E-06 | 1.50E-05 |
| eca-mir-214 | 1.515890287 | 7.07E-14 | 7.03E-13 |
| eca-mir-224 | 1.506320107 | 0.0022558 | 0.00515611 |
| eca-mir-128-1 | 1.503305273 | 0.00051171 | 0.00133553 |
| eca-mir-100 | 1.477966097 | 5.28E-07 | 2.43E-06 |
| eca-mir-181b | 1.447817969 | 5.47E-07 | 2.48E-06 |
| eca-mir-345 | 1.424758469 | 3.39E-08 | 1.78E-07 |
| eca-mir-125b | 1.41889486 | 9.38E-12 | 7.34E-11 |
| eca-mir-2483 | 1.34359714 | 0.00049098 | 0.00129986 |
| eca-mir-8958 | 1.280330207 | 0.00476682 | 0.00991068 |
| eca-mir-1301 | 1.172403921 | 3.12E-06 | 1.22E-05 |
| eca-mir-9185 | 1.17195442 | 0.00010779 | 0.00033055 |
| eca-mir-221 | 1.167519504 | 1.26E-06 | 5.36E-06 |
| eca-mir-146a | 1.165721109 | 0.00011732 | 0.0003568 |
| eca-mir-8977 | 1.148397387 | 0.02381237 | 0.041928 |
| eca-mir-199b | 1.142208116 | 2.81E-14 | 2.88E-13 |
| eca-mir-454 | 1.093740246 | 1.50E-05 | 5.34E-05 |
| ENSECAG00000038227 | 1.080736785 | 2.16E-16 | 2.65E-15 |
| eca-mir-199a | 1.056255777 | 1.60E-13 | 1.47E-12 |
| eca-mir-3613 | 1.055112956 | 0.00028141 | 0.00079052 |
| eca-mir-330 | 1.04299213 | 6.60E-05 | 0.00020752 |
| eca-mir-130b | 1.01207733 | 0.00117695 | 0.00279432 |
| ENSECAG00000050098 | 1.007975668 | 1.27E-06 | 5.36E-06 |
| eca-mir-126 | -1.101654262 | 1.01E-08 | 6.00E-08 |
| eca-mir-139 | -1.113339705 | 2.31E-07 | 1.12E-06 |
| eca-mir-3065 | -1.119259613 | 0.00769506 | 0.01529709 |
| eca-let-7f-2 | -1.135002328 | 1.91E-09 | 1.30E-08 |
| ENSECAG00000037174 | -1.141398595 | 0.00292466 | 0.00644477 |
| eca-mir-92a | -1.172959525 | 6.02E-07 | 2.70E-06 |
| eca-mir-122 | -1.185759387 | 0.00250568 | 0.0056225 |
| eca-mir-483 | -1.197130815 | 0.0002763 | 0.00078213 |
| eca-mir-449b | -1.20420671 | 0.00013523 | 0.00040133 |
| eca-mir-92a-2 | -1.213643623 | 1.03E-16 | 1.36E-15 |
| eca-let-7a | -1.266000335 | 2.67E-09 | 1.75E-08 |
| eca-mir-8978 | -1.293207185 | 0.00773168 | 0.01529709 |
| ENSECAG00000025939 | -1.415952384 | 2.93E-06 | 1.16E-05 |
| eca-mir-9000 | -1.459094035 | 0.00091614 | 0.00226268 |
| eca-mir-551a | -1.55349084 | 2.32E-06 | 9.60E-06 |
| eca-mir-8928 | -1.63334705 | 9.01E-05 | 0.00028095 |
| eca-mir-873 | -1.651634274 | 0.00270969 | 0.00600703 |
| eca-mir-8986a | -1.741452928 | 2.77E-08 | 1.52E-07 |
| eca-mir-9105 | -1.788973188 | 0.00071678 | 0.00181914 |
| eca-mir-885 | -1.80418391 | 0.00038239 | 0.00104237 |
| eca-mir-592 | -1.82452832 | 3.83E-07 | 1.79E-06 |
| eca-mir-143 | -1.833940936 | 7.49E-19 | 1.53E-17 |
| eca-mir-8915 | -1.8865301 | 0.00163456 | 0.00378313 |
| eca-mir-8986b | -1.90964272 | 5.45E-08 | 2.79E-07 |
| eca-mir-145 | -1.919406019 | 1.08E-16 | 1.37E-15 |
| eca-mir-9023 | -2.012244605 | 0.00063568 | 0.00163588 |
| eca-mir-9147 | -2.058671714 | 5.40E-06 | 2.03E-05 |
| eca-mir-9152 | -2.085703315 | 0.0039056 | 0.00840504 |
| eca-mir-486 | -2.106716213 | 0.00090212 | 0.00224311 |
| eca-mir-488 | -2.134075471 | 0.00533474 | 0.0109675 |
| eca-mir-95 | -2.224760818 | 2.43E-06 | 9.81E-06 |
| eca-mir-9184 | -2.254662137 | 0.00021488 | 0.00062263 |
| eca-mir-216a | -2.271098644 | 0.00187333 | 0.00430866 |
| eca-mir-205 | -2.27211667 | 0.00385682 | 0.00834888 |
| eca-mir-9180 | -2.279378903 | 0.01033883 | 0.01981609 |
| eca-mir-1248 | -2.282691131 | 3.23E-08 | 1.72E-07 |
| eca-mir-9034 | -2.354819362 | 3.20E-08 | 1.72E-07 |
| eca-mir-184 | -2.377660833 | 9.18E-06 | 3.38E-05 |
| eca-mir-451 | -2.381223338 | 0.00262859 | 0.00586255 |
| eca-mir-708 | -2.443812719 | 2.46E-07 | 1.17E-06 |
| eca-mir-141 | -2.493132269 | 6.32E-05 | 0.00020046 |
| eca-mir-135b | -2.530932896 | 2.47E-06 | 9.87E-06 |
| eca-mir-429 | -2.571580776 | 0.00035705 | 0.00098056 |
| eca-mir-200c | -2.706684628 | 3.21E-05 | 0.00011041 |
| eca-mir-96 | -2.712723502 | 4.90E-06 | 1.86E-05 |
| eca-mir-200b | -2.738282554 | 0.00031 | 0.00085775 |
| eca-mir-182 | -2.795596884 | 6.93E-07 | 3.07E-06 |
| eca-mir-9013 | -2.899341018 | 1.32E-05 | 4.76E-05 |
| eca-mir-135a-2 | -2.925555953 | 4.27E-08 | 2.21E-07 |
| eca-mir-1291a | -2.977285471 | 0.00014047 | 0.00041354 |
| eca-mir-183 | -3.012305409 | 2.22E-08 | 1.27E-07 |
| eca-mir-204b-2 | -3.11897216 | 6.35E-21 | 1.96E-19 |
| eca-mir-489 | -3.492502908 | 7.62E-07 | 3.34E-06 |
| eca-mir-375 | -3.493821726 | 3.85E-05 | 0.0001301 |
| eca-mir-8939 | -3.643236704 | 1.14E-09 | 7.89E-09 |
| eca-mir-653 | -4.085318444 | 4.13E-05 | 0.00013829 |

Supplementary table S5. Differentially expressed genes in sarcoid vs control groups.

| Ensembl gene ID | log2FoldChange | pvalue | FDR |
| --- | --- | --- | --- |
| ENSECAG00000000092 | 8.51749074 | 9.97E-09 | 1.13E-07 |
| ENSECAG00000005948 | 8.27881924 | 1.55E-17 | 8.66E-16 |
| ENSECAG00000037274 | 7.87640042 | 5.31E-12 | 1.15E-10 |
| ENSECAG00000032944 | 7.70592044 | 3.78E-15 | 1.43E-13 |
| ENSECAG00000035391 | 7.69454691 | 3.07E-28 | 8.17E-26 |
| ENSECAG00000044439 | 7.67657033 | 4.19E-21 | 3.84E-19 |
| ENSECAG00000048241 | 7.5460684 | 2.77E-08 | 2.81E-07 |
| ENSECAG00000012690 | 7.38263215 | 1.51E-11 | 3.05E-10 |
| ENSECAG00000022590 | 7.35268759 | 0.00035873 | 0.0012867 |
| ENSECAG00000020355 | 7.33405638 | 6.97E-09 | 8.24E-08 |
| ENSECAG00000016567 | 7.31295011 | 2.17E-09 | 2.90E-08 |
| ENSECAG00000031731 | 7.30596247 | 0.00049014 | 0.00168512 |
| ENSECAG00000052623 | 7.21526567 | 3.96E-07 | 3.04E-06 |
| ENSECAG00000052066 | 7.20583044 | 2.51E-05 | 0.00012279 |
| ENSECAG00000051645 | 7.18811836 | 6.04E-10 | 9.00E-09 |
| ENSECAG00000051458 | 7.05831726 | 1.23E-06 | 8.40E-06 |
| ENSECAG00000006879 | 7.00118672 | 3.31E-09 | 4.20E-08 |
| ENSECAG00000044210 | 6.9644999 | 3.32E-09 | 4.21E-08 |
| ENSECAG00000008946 | 6.87889888 | 7.48E-12 | 1.58E-10 |
| ENSECAG00000034891 | 6.84217894 | 4.55E-11 | 8.45E-10 |
| ENSECAG00000019048 | 6.7836897 | 0.00025046 | 0.00093988 |
| ENSECAG00000046053 | 6.7802074 | 2.93E-38 | 3.12E-35 |
| ENSECAG00000046821 | 6.73410753 | 1.94E-09 | 2.62E-08 |
| ENSECAG00000020826 | 6.73056976 | 4.91E-07 | 3.70E-06 |
| ENSECAG00000012702 | 6.68032548 | 0.00042357 | 0.00148729 |
| ENSECAG00000019922 | 6.6003161 | 2.76E-07 | 2.20E-06 |
| ENSECAG00000059991 | 6.57602062 | 6.33E-17 | 3.14E-15 |
| ENSECAG00000058637 | 6.47509491 | 0.00090897 | 0.00290626 |
| ENSECAG00000059098 | 6.44973129 | 2.46E-11 | 4.76E-10 |
| ENSECAG00000011260 | 6.4024074 | 6.02E-09 | 7.21E-08 |
| ENSECAG00000013717 | 6.33737396 | 6.22E-14 | 1.91E-12 |
| ENSECAG00000022230 | 6.32873564 | 5.09E-06 | 2.95E-05 |
| ENSECAG00000016482 | 6.3080576 | 4.10E-05 | 0.00019079 |
| ENSECAG00000037997 | 6.19974129 | 3.96E-05 | 0.00018486 |
| ENSECAG00000016325 | 6.02051423 | 0.00022527 | 0.00085587 |
| ENSECAG00000039631 | 5.95032133 | 3.63E-07 | 2.82E-06 |
| ENSECAG00000049329 | 5.91274626 | 0.00042519 | 0.00149227 |
| ENSECAG00000049449 | 5.90160899 | 4.98E-11 | 9.18E-10 |
| ENSECAG00000049514 | 5.89236311 | 1.10E-06 | 7.61E-06 |
| ENSECAG00000029110 | 5.86782188 | 3.75E-18 | 2.30E-16 |
| ENSECAG00000013199 | 5.86020582 | 0.0012699 | 0.00389582 |
| ENSECAG00000054424 | 5.85045427 | 3.56E-17 | 1.85E-15 |
| ENSECAG00000048157 | 5.73080952 | 9.16E-09 | 1.05E-07 |
| ENSECAG00000050649 | 5.55443995 | 6.54E-06 | 3.70E-05 |
| ENSECAG00000055363 | 5.53487699 | 2.48E-14 | 8.07E-13 |
| ENSECAG00000048888 | 5.52516327 | 0.00091862 | 0.0029332 |
| ENSECAG00000056392 | 5.50664939 | 8.18E-08 | 7.40E-07 |
| ENSECAG00000056249 | 5.47646603 | 6.13E-06 | 3.49E-05 |
| ENSECAG00000010869 | 5.37367048 | 1.91E-11 | 3.76E-10 |
| ENSECAG00000058246 | 5.35325029 | 5.29E-08 | 5.01E-07 |
| ENSECAG00000047759 | 5.34908743 | 1.33E-22 | 1.50E-20 |
| ENSECAG00000020373 | 5.34375972 | 2.03E-06 | 1.31E-05 |
| ENSECAG00000060279 | 5.31419637 | 2.28E-07 | 1.86E-06 |
| ENSECAG00000046503 | 5.29400056 | 4.07E-05 | 0.00018947 |
| ENSECAG00000047014 | 5.25946406 | 2.20E-06 | 1.41E-05 |
| ENSECAG00000037831 | 5.24184867 | 1.30E-10 | 2.21E-09 |
| ENSECAG00000049849 | 5.19103535 | 2.66E-17 | 1.42E-15 |
| ENSECAG00000059461 | 5.12569505 | 0.01642294 | 0.03574085 |
| ENSECAG00000012742 | 5.12419276 | 3.63E-09 | 4.57E-08 |
| ENSECAG00000052712 | 5.10258756 | 4.69E-07 | 3.54E-06 |
| ENSECAG00000057128 | 5.10075873 | 5.87E-09 | 7.07E-08 |
| ENSECAG00000046014 | 5.09791358 | 5.54E-12 | 1.19E-10 |
| ENSECAG00000001034 | 5.07393074 | 1.08E-08 | 1.21E-07 |
| ENSECAG00000036059 | 5.06728207 | 2.36E-18 | 1.48E-16 |
| ENSECAG00000011404 | 5.06647494 | 3.78E-08 | 3.71E-07 |
| ENSECAG00000045572 | 5.06064554 | 0.01085894 | 0.02507792 |
| ENSECAG00000056945 | 5.05995739 | 0.00026246 | 0.00098074 |
| ENSECAG00000048679 | 5.05123534 | 9.12E-05 | 0.00038894 |
| ENSECAG00000034502 | 5.02186142 | 3.79E-07 | 2.93E-06 |
| ENSECAG00000011334 | 5.01720069 | 1.94E-06 | 1.26E-05 |
| ENSECAG00000055512 | 5.01483455 | 2.57E-08 | 2.64E-07 |
| ENSECAG00000043839 | 4.97970565 | 2.99E-13 | 8.14E-12 |
| ENSECAG00000022850 | 4.97065079 | 8.95E-08 | 8.03E-07 |
| ENSECAG00000000065 | 4.93909063 | 0.00152466 | 0.004575 |
| ENSECAG00000045458 | 4.93434153 | 9.53E-15 | 3.36E-13 |
| ENSECAG00000058764 | 4.92865407 | 9.00E-06 | 4.92E-05 |
| ENSECAG00000054390 | 4.92350649 | 1.80E-08 | 1.92E-07 |
| ENSECAG00000008597 | 4.91024492 | 0.00014311 | 0.00057494 |
| ENSECAG00000054655 | 4.9038609 | 0.01049496 | 0.02437082 |
| ENSECAG00000049787 | 4.89565552 | 4.06E-06 | 2.42E-05 |
| ENSECAG00000047076 | 4.86175716 | 0.00159374 | 0.00475167 |
| ENSECAG00000021446 | 4.85428722 | 2.98E-12 | 6.68E-11 |
| ENSECAG00000059511 | 4.85019969 | 8.87E-05 | 0.00037926 |
| ENSECAG00000059589 | 4.84412476 | 5.97E-09 | 7.17E-08 |
| ENSECAG00000011274 | 4.83748024 | 0.00141565 | 0.00428988 |
| ENSECAG00000014395 | 4.83572719 | 3.63E-12 | 8.04E-11 |
| ENSECAG00000023425 | 4.8354173 | 2.40E-19 | 1.80E-17 |
| ENSECAG00000016649 | 4.82938506 | 2.26E-15 | 8.78E-14 |
| ENSECAG00000046252 | 4.82414668 | 4.03E-15 | 1.51E-13 |
| ENSECAG00000002935 | 4.80179354 | 7.73E-23 | 9.05E-21 |
| ENSECAG00000054997 | 4.78995601 | 5.96E-09 | 7.17E-08 |
| ENSECAG00000037347 | 4.78034567 | 1.04E-18 | 6.91E-17 |
| ENSECAG00000047293 | 4.77360916 | 4.12E-18 | 2.51E-16 |
| ENSECAG00000030364 | 4.76531199 | 1.58E-06 | 1.05E-05 |
| ENSECAG00000012602 | 4.76304671 | 7.14E-25 | 1.17E-22 |
| ENSECAG00000021568 | 4.74301806 | 5.52E-07 | 4.11E-06 |
| ENSECAG00000032607 | 4.71178081 | 0.00074486 | 0.00243664 |
| ENSECAG00000020070 | 4.70164809 | 5.75E-05 | 0.00025734 |
| ENSECAG00000013924 | 4.68644105 | 2.31E-13 | 6.40E-12 |
| ENSECAG00000021728 | 4.67431184 | 3.61E-08 | 3.57E-07 |
| ENSECAG00000014295 | 4.66031515 | 6.22E-05 | 0.00027699 |
| ENSECAG00000024627 | 4.65080401 | 2.76E-12 | 6.24E-11 |
| ENSECAG00000032622 | 4.62381813 | 5.95E-07 | 4.38E-06 |
| ENSECAG00000010025 | 4.61573257 | 8.00E-14 | 2.38E-12 |
| ENSECAG00000056331 | 4.61303937 | 6.08E-13 | 1.57E-11 |
| ENSECAG00000032414 | 4.61060725 | 0.00017233 | 0.00067608 |
| ENSECAG00000029056 | 4.59726238 | 0.00012437 | 0.00050845 |
| ENSECAG00000014510 | 4.58399834 | 1.26E-08 | 1.39E-07 |
| ENSECAG00000054526 | 4.57669929 | 1.24E-10 | 2.12E-09 |
| ENSECAG00000020526 | 4.56586105 | 0.00744112 | 0.01814088 |
| ENSECAG00000049353 | 4.54533651 | 1.97E-09 | 2.66E-08 |
| ENSECAG00000040200 | 4.53142631 | 3.29E-09 | 4.18E-08 |
| ENSECAG00000055019 | 4.52855378 | 0.00017842 | 0.00069688 |
| ENSECAG00000046675 | 4.49797035 | 0.00614568 | 0.01539245 |
| ENSECAG00000013106 | 4.49217811 | 4.80E-10 | 7.26E-09 |
| ENSECAG00000020494 | 4.48975025 | 1.24E-20 | 1.08E-18 |
| ENSECAG00000038266 | 4.45896672 | 0.00056053 | 0.00189839 |
| ENSECAG00000041814 | 4.45171192 | 7.00E-07 | 5.05E-06 |
| ENSECAG00000056423 | 4.44607731 | 0.0077379 | 0.01876003 |
| ENSECAG00000012011 | 4.41891341 | 1.20E-25 | 2.28E-23 |
| ENSECAG00000021886 | 4.41191775 | 8.86E-17 | 4.27E-15 |
| ENSECAG00000013453 | 4.39907095 | 0.00036213 | 0.0012964 |
| ENSECAG00000057078 | 4.38140341 | 5.52E-11 | 1.01E-09 |
| ENSECAG00000014725 | 4.3780557 | 0.00084749 | 0.00273474 |
| ENSECAG00000036646 | 4.37736618 | 1.04E-13 | 3.01E-12 |
| ENSECAG00000044204 | 4.34935379 | 1.47E-09 | 2.03E-08 |
| ENSECAG00000016169 | 4.34656109 | 9.05E-10 | 1.30E-08 |
| ENSECAG00000045275 | 4.34540458 | 0.00010195 | 0.00042845 |
| ENSECAG00000048350 | 4.33430177 | 0.00106256 | 0.00332963 |
| ENSECAG00000030884 | 4.33342799 | 0.0058584 | 0.01478061 |
| ENSECAG00000027765 | 4.33157531 | 1.24E-11 | 2.54E-10 |
| ENSECAG00000051525 | 4.30968518 | 0.01850101 | 0.03959996 |
| ENSECAG00000031150 | 4.30855891 | 0.00078437 | 0.00255279 |
| ENSECAG00000020940 | 4.27292235 | 2.33E-11 | 4.53E-10 |
| ENSECAG00000000203 | 4.26090551 | 5.58E-07 | 4.14E-06 |
| ENSECAG00000013243 | 4.23205674 | 6.55E-28 | 1.64E-25 |
| ENSECAG00000006917 | 4.21627534 | 3.93E-35 | 2.44E-32 |
| ENSECAG00000030430 | 4.2146631 | 5.80E-07 | 4.28E-06 |
| ENSECAG00000018248 | 4.19697218 | 3.16E-44 | 6.43E-41 |
| ENSECAG00000056552 | 4.17035753 | 4.89E-09 | 6.00E-08 |
| ENSECAG00000053458 | 4.15803017 | 8.15E-13 | 2.04E-11 |
| ENSECAG00000002782 | 4.15776369 | 3.25E-09 | 4.14E-08 |
| ENSECAG00000051570 | 4.14937286 | 2.21E-06 | 1.41E-05 |
| ENSECAG00000058560 | 4.1356738 | 7.02E-14 | 2.13E-12 |
| ENSECAG00000021547 | 4.12524167 | 0.00015995 | 0.00063351 |
| ENSECAG00000048778 | 4.11818773 | 0.00016829 | 0.0006623 |
| ENSECAG00000047720 | 4.09245616 | 2.58E-10 | 4.12E-09 |
| ENSECAG00000058459 | 4.08775765 | 2.05E-05 | 0.00010236 |
| ENSECAG00000023888 | 4.08394577 | 8.48E-24 | 1.20E-21 |
| ENSECAG00000047160 | 4.0743179 | 4.26E-07 | 3.24E-06 |
| ENSECAG00000016797 | 4.07174706 | 1.61E-09 | 2.21E-08 |
| ENSECAG00000056976 | 4.07154141 | 1.36E-07 | 1.17E-06 |
| ENSECAG00000049100 | 4.06193758 | 3.39E-05 | 0.00016051 |
| ENSECAG00000036051 | 4.05287484 | 7.43E-07 | 5.33E-06 |
| ENSECAG00000055603 | 4.04806052 | 2.55E-16 | 1.14E-14 |
| ENSECAG00000022263 | 4.04733558 | 7.30E-24 | 1.05E-21 |
| ENSECAG00000035666 | 4.02228361 | 0.00048988 | 0.00168464 |
| ENSECAG00000037840 | 4.01847563 | 1.20E-07 | 1.05E-06 |
| ENSECAG00000004572 | 4.00106839 | 7.81E-25 | 1.27E-22 |
| ENSECAG00000009252 | 3.99481563 | 3.55E-06 | 2.15E-05 |
| ENSECAG00000018008 | 3.97353535 | 2.70E-09 | 3.52E-08 |
| ENSECAG00000050609 | 3.97253115 | 8.08E-10 | 1.16E-08 |
| ENSECAG00000018691 | 3.95859802 | 1.29E-06 | 8.73E-06 |
| ENSECAG00000041160 | 3.94999349 | 3.70E-05 | 0.00017357 |
| ENSECAG00000058229 | 3.94289456 | 6.82E-09 | 8.08E-08 |
| ENSECAG00000029191 | 3.92652387 | 0.00874482 | 0.02085059 |
| ENSECAG00000022062 | 3.92565303 | 5.53E-21 | 4.98E-19 |
| ENSECAG00000010059 | 3.92367345 | 1.24E-19 | 9.76E-18 |
| ENSECAG00000030285 | 3.90714381 | 1.46E-05 | 7.58E-05 |
| ENSECAG00000053220 | 3.90501261 | 3.54E-06 | 2.14E-05 |
| ENSECAG00000053132 | 3.85830257 | 3.01E-06 | 1.86E-05 |
| ENSECAG00000030913 | 3.85577392 | 4.12E-08 | 4.00E-07 |
| ENSECAG00000028320 | 3.85372945 | 7.23E-09 | 8.52E-08 |
| ENSECAG00000021008 | 3.84150647 | 7.51E-09 | 8.80E-08 |
| ENSECAG00000032305 | 3.83153778 | 0.00046275 | 0.00160544 |
| ENSECAG00000033117 | 3.82756568 | 0.00103361 | 0.00325449 |
| ENSECAG00000023485 | 3.82280973 | 0.00556532 | 0.01413855 |
| ENSECAG00000051854 | 3.81567322 | 0.00013501 | 0.00054537 |
| ENSECAG00000013318 | 3.8107251 | 1.68E-05 | 8.57E-05 |
| ENSECAG00000006777 | 3.80436614 | 1.06E-06 | 7.37E-06 |
| ENSECAG00000046004 | 3.78846876 | 0.00354248 | 0.00954775 |
| ENSECAG00000014254 | 3.78813782 | 3.46E-09 | 4.38E-08 |
| ENSECAG00000043977 | 3.78671369 | 1.58E-05 | 8.13E-05 |
| ENSECAG00000045529 | 3.75896432 | 2.45E-06 | 1.55E-05 |
| ENSECAG00000037578 | 3.75181111 | 3.32E-11 | 6.30E-10 |
| ENSECAG00000024042 | 3.74932288 | 2.35E-08 | 2.44E-07 |
| ENSECAG00000000041 | 3.73659602 | 4.49E-09 | 5.58E-08 |
| ENSECAG00000011627 | 3.73600005 | 7.30E-07 | 5.24E-06 |
| ENSECAG00000045952 | 3.73364907 | 3.99E-09 | 4.99E-08 |
| ENSECAG00000057962 | 3.72970943 | 0.0091431 | 0.02164089 |
| ENSECAG00000056980 | 3.72727456 | 0.00305248 | 0.00837147 |
| ENSECAG00000052918 | 3.72454822 | 0.00011048 | 0.00046024 |
| ENSECAG00000056416 | 3.71032395 | 2.10E-07 | 1.73E-06 |
| ENSECAG00000020251 | 3.70225544 | 1.78E-68 | 3.97E-64 |
| ENSECAG00000018134 | 3.69558845 | 5.36E-05 | 0.00024219 |
| ENSECAG00000058995 | 3.68507397 | 0.00029718 | 0.00109187 |
| ENSECAG00000022352 | 3.68391449 | 1.83E-10 | 3.02E-09 |
| ENSECAG00000055762 | 3.68017537 | 3.53E-06 | 2.14E-05 |
| ENSECAG00000043288 | 3.65779235 | 2.12E-09 | 2.83E-08 |
| ENSECAG00000044160 | 3.65767697 | 0.00367292 | 0.00985886 |
| ENSECAG00000035913 | 3.64333745 | 0.00015284 | 0.0006088 |
| ENSECAG00000037583 | 3.64197084 | 0.00813357 | 0.0196129 |
| ENSECAG00000019682 | 3.63562613 | 1.29E-05 | 6.76E-05 |
| ENSECAG00000012744 | 3.62285357 | 4.10E-13 | 1.09E-11 |
| ENSECAG00000054134 | 3.62083491 | 2.30E-05 | 0.00011372 |
| ENSECAG00000021205 | 3.61881577 | 5.32E-08 | 5.03E-07 |
| ENSECAG00000044666 | 3.61619573 | 0.01883836 | 0.0402065 |
| ENSECAG00000009930 | 3.61567674 | 3.18E-13 | 8.59E-12 |
| ENSECAG00000059685 | 3.60743531 | 0.00499065 | 0.01286455 |
| ENSECAG00000005327 | 3.59696758 | 0.00011566 | 0.00047789 |
| ENSECAG00000013526 | 3.59691853 | 1.08E-05 | 5.78E-05 |
| ENSECAG00000057388 | 3.59490132 | 5.05E-05 | 0.00022954 |
| ENSECAG00000055308 | 3.58763417 | 0.00122318 | 0.00377009 |
| ENSECAG00000044559 | 3.58727196 | 5.60E-07 | 4.15E-06 |
| ENSECAG00000036894 | 3.58532412 | 0.00020847 | 0.00079865 |
| ENSECAG00000045808 | 3.58438656 | 1.97E-05 | 9.92E-05 |
| ENSECAG00000035279 | 3.5825048 | 2.97E-31 | 1.21E-28 |
| ENSECAG00000054041 | 3.58245603 | 0.00910612 | 0.02157394 |
| ENSECAG00000014774 | 3.57691049 | 2.27E-29 | 6.68E-27 |
| ENSECAG00000042387 | 3.57048329 | 3.36E-05 | 0.00015942 |
| ENSECAG00000035175 | 3.56724172 | 0.00012037 | 0.0004948 |
| ENSECAG00000050948 | 3.56572649 | 7.92E-14 | 2.37E-12 |
| ENSECAG00000004716 | 3.56507047 | 2.28E-09 | 3.04E-08 |
| ENSECAG00000047377 | 3.56243593 | 8.93E-35 | 5.39E-32 |
| ENSECAG00000036940 | 3.55901999 | 0.01776451 | 0.03825438 |
| ENSECAG00000015770 | 3.55604224 | 1.64E-06 | 1.08E-05 |
| ENSECAG00000025610 | 3.54392692 | 0.00626551 | 0.01566446 |
| ENSECAG00000054016 | 3.54345507 | 0.00237264 | 0.00674612 |
| ENSECAG00000036372 | 3.54138904 | 4.06E-07 | 3.11E-06 |
| ENSECAG00000035701 | 3.5403681 | 0.00014872 | 0.00059495 |
| ENSECAG00000015083 | 3.53709896 | 9.37E-08 | 8.35E-07 |
| ENSECAG00000004217 | 3.53550473 | 0.01390801 | 0.03099824 |
| ENSECAG00000032386 | 3.53255136 | 0.00240123 | 0.00681147 |
| ENSECAG00000033892 | 3.53053144 | 6.02E-19 | 4.24E-17 |
| ENSECAG00000049110 | 3.51454913 | 0.02053518 | 0.04329048 |
| ENSECAG00000026037 | 3.50229302 | 4.53E-08 | 4.36E-07 |
| ENSECAG00000012104 | 3.49029169 | 1.21E-06 | 8.26E-06 |
| ENSECAG00000043937 | 3.48851269 | 3.35E-10 | 5.22E-09 |
| ENSECAG00000014772 | 3.48757146 | 2.43E-09 | 3.20E-08 |
| ENSECAG00000019243 | 3.48745304 | 5.40E-11 | 9.87E-10 |
| ENSECAG00000018168 | 3.46886983 | 3.35E-19 | 2.45E-17 |
| ENSECAG00000013100 | 3.46528177 | 1.60E-17 | 8.92E-16 |
| ENSECAG00000059626 | 3.45470454 | 3.94E-06 | 2.35E-05 |
| ENSECAG00000044773 | 3.44094586 | 0.00895506 | 0.02127018 |
| ENSECAG00000049680 | 3.42737579 | 1.95E-06 | 1.26E-05 |
| ENSECAG00000045914 | 3.42037712 | 6.35E-07 | 4.63E-06 |
| ENSECAG00000054248 | 3.41947329 | 0.00302947 | 0.00831653 |
| ENSECAG00000052101 | 3.41931492 | 0.0022953 | 0.00655808 |
| ENSECAG00000029750 | 3.41066154 | 0.00032571 | 0.00118424 |
| ENSECAG00000040478 | 3.40820585 | 2.62E-09 | 3.43E-08 |
| ENSECAG00000021110 | 3.40769011 | 1.01E-13 | 2.96E-12 |
| ENSECAG00000016927 | 3.39978568 | 2.27E-21 | 2.19E-19 |
| ENSECAG00000012212 | 3.39633818 | 1.93E-07 | 1.61E-06 |
| ENSECAG00000056687 | 3.39041995 | 1.77E-05 | 8.98E-05 |
| ENSECAG00000053341 | 3.39037888 | 1.16E-12 | 2.84E-11 |
| ENSECAG00000024769 | 3.39019219 | 1.26E-09 | 1.75E-08 |
| ENSECAG00000049382 | 3.38920554 | 3.95E-22 | 4.27E-20 |
| ENSECAG00000019514 | 3.37979071 | 9.15E-07 | 6.42E-06 |
| ENSECAG00000045239 | 3.37543419 | 0.00472987 | 0.01228996 |
| ENSECAG00000054486 | 3.37388479 | 2.45E-05 | 0.00012026 |
| ENSECAG00000049507 | 3.37060984 | 0.00030588 | 0.00111998 |
| ENSECAG00000023992 | 3.36971701 | 6.48E-08 | 6.01E-07 |
| ENSECAG00000032667 | 3.36909418 | 2.22E-05 | 0.00010978 |
| ENSECAG00000030646 | 3.36660751 | 7.96E-10 | 1.15E-08 |
| ENSECAG00000016086 | 3.34861303 | 0.00084971 | 0.0027411 |
| ENSECAG00000046756 | 3.34657698 | 0.00025953 | 0.00097113 |
| ENSECAG00000040205 | 3.33977395 | 0.0017057 | 0.00505043 |
| ENSECAG00000035336 | 3.33753696 | 1.27E-05 | 6.69E-05 |
| ENSECAG00000053920 | 3.33232476 | 0.00901827 | 0.02140212 |
| ENSECAG00000032431 | 3.33060054 | 0.00141772 | 0.00429447 |
| ENSECAG00000055101 | 3.32743195 | 1.93E-11 | 3.79E-10 |
| ENSECAG00000057763 | 3.32319294 | 0.00282227 | 0.00781977 |
| ENSECAG00000015851 | 3.32032381 | 0.00068376 | 0.00226192 |
| ENSECAG00000044804 | 3.31870085 | 2.38E-13 | 6.57E-12 |
| ENSECAG00000017662 | 3.30587395 | 2.41E-06 | 1.53E-05 |
| ENSECAG00000034472 | 3.30489276 | 2.08E-08 | 2.19E-07 |
| ENSECAG00000049130 | 3.29862561 | 9.17E-09 | 1.05E-07 |
| ENSECAG00000014346 | 3.29644989 | 4.50E-06 | 2.64E-05 |
| ENSECAG00000021196 | 3.29463699 | 6.45E-07 | 4.70E-06 |
| ENSECAG00000002430 | 3.29031057 | 1.97E-10 | 3.23E-09 |
| ENSECAG00000049873 | 3.28565218 | 3.12E-06 | 1.92E-05 |
| ENSECAG00000046900 | 3.273592 | 0.00029613 | 0.0010889 |
| ENSECAG00000058617 | 3.27190354 | 7.49E-05 | 0.00032677 |
| ENSECAG00000020869 | 3.26117379 | 0.00015196 | 0.00060552 |
| ENSECAG00000006830 | 3.25785914 | 1.88E-05 | 9.51E-05 |
| ENSECAG00000026349 | 3.25367211 | 0.00612541 | 0.01535597 |
| ENSECAG00000008483 | 3.25280017 | 5.68E-29 | 1.61E-26 |
| ENSECAG00000054004 | 3.24481133 | 0.0095442 | 0.02245943 |
| ENSECAG00000042865 | 3.2398905 | 8.03E-05 | 0.00034709 |
| ENSECAG00000024272 | 3.23331066 | 6.27E-05 | 0.00027887 |
| ENSECAG00000056431 | 3.22869668 | 2.01E-06 | 1.30E-05 |
| ENSECAG00000028035 | 3.22763456 | 0.00362158 | 0.00973978 |
| ENSECAG00000003515 | 3.22759423 | 0.00013338 | 0.0005396 |
| ENSECAG00000024740 | 3.21549229 | 1.72E-11 | 3.43E-10 |
| ENSECAG00000016139 | 3.21450612 | 6.64E-08 | 6.14E-07 |
| ENSECAG00000022142 | 3.21269356 | 2.13E-06 | 1.37E-05 |
| ENSECAG00000013771 | 3.2124435 | 2.23E-12 | 5.16E-11 |
| ENSECAG00000008400 | 3.21042287 | 7.63E-07 | 5.45E-06 |
| ENSECAG00000051935 | 3.2087634 | 0.00132749 | 0.0040569 |
| ENSECAG00000022465 | 3.2019088 | 0.00790867 | 0.01913461 |
| ENSECAG00000021022 | 3.20129235 | 4.80E-40 | 5.64E-37 |
| ENSECAG00000013342 | 3.20110899 | 3.30E-05 | 0.0001571 |
| ENSECAG00000032894 | 3.19759056 | 6.54E-05 | 0.00028944 |
| ENSECAG00000033726 | 3.18203619 | 0.00026378 | 0.00098488 |
| ENSECAG00000022074 | 3.17915728 | 9.41E-08 | 8.37E-07 |
| ENSECAG00000031827 | 3.17891301 | 1.18E-12 | 2.88E-11 |
| ENSECAG00000012784 | 3.17851018 | 3.42E-43 | 5.87E-40 |
| ENSECAG00000005343 | 3.17540757 | 2.13E-18 | 1.34E-16 |
| ENSECAG00000013693 | 3.17140071 | 3.99E-09 | 4.99E-08 |
| ENSECAG00000048018 | 3.1705798 | 0.0002565 | 0.00096068 |
| ENSECAG00000022526 | 3.17042418 | 1.08E-05 | 5.76E-05 |
| ENSECAG00000022068 | 3.15957255 | 2.74E-49 | 2.04E-45 |
| ENSECAG00000044939 | 3.14529216 | 0.0095409 | 0.0224564 |
| ENSECAG00000016945 | 3.14518567 | 0.02299203 | 0.04760306 |
| ENSECAG00000058175 | 3.1443949 | 0.01267524 | 0.02858133 |
| ENSECAG00000019781 | 3.12798836 | 6.29E-12 | 1.34E-10 |
| ENSECAG00000057644 | 3.12767722 | 0.00022719 | 0.00086237 |
| ENSECAG00000016101 | 3.12390317 | 1.15E-15 | 4.71E-14 |
| ENSECAG00000016121 | 3.12370368 | 2.39E-23 | 3.05E-21 |
| ENSECAG00000052929 | 3.12123695 | 0.00170613 | 0.00505104 |
| ENSECAG00000033412 | 3.11797032 | 0.00208922 | 0.00603722 |
| ENSECAG00000003452 | 3.11140695 | 7.11E-08 | 6.52E-07 |
| ENSECAG00000044649 | 3.10629062 | 9.49E-21 | 8.39E-19 |
| ENSECAG00000014713 | 3.10626446 | 1.78E-13 | 5.02E-12 |
| ENSECAG00000021029 | 3.10579883 | 2.96E-12 | 6.64E-11 |
| ENSECAG00000021541 | 3.10515046 | 0.00044842 | 0.00156153 |
| ENSECAG00000052646 | 3.10425059 | 0.00021499 | 0.00082123 |
| ENSECAG00000056387 | 3.09789484 | 0.00190942 | 0.00557895 |
| ENSECAG00000059253 | 3.09562872 | 0.00038609 | 0.00136885 |
| ENSECAG00000041951 | 3.09541449 | 0.00066657 | 0.00220997 |
| ENSECAG00000021712 | 3.09119127 | 1.33E-22 | 1.50E-20 |
| ENSECAG00000012370 | 3.08035154 | 0.00015006 | 0.00059922 |
| ENSECAG00000024232 | 3.07500068 | 2.42E-05 | 0.00011888 |
| ENSECAG00000055860 | 3.07195818 | 6.81E-06 | 3.83E-05 |
| ENSECAG00000035315 | 3.07178904 | 0.00204929 | 0.0059364 |
| ENSECAG00000047209 | 3.06572741 | 0.01017402 | 0.02372417 |
| ENSECAG00000035510 | 3.06524955 | 0.00137854 | 0.00419341 |
| ENSECAG00000057712 | 3.06117604 | 9.81E-18 | 5.65E-16 |
| ENSECAG00000055734 | 3.04583313 | 0.02100102 | 0.04412684 |
| ENSECAG00000057636 | 3.04044235 | 0.00158314 | 0.00472321 |
| ENSECAG00000032630 | 3.03735494 | 0.00224554 | 0.00643484 |
| ENSECAG00000045631 | 3.03355395 | 0.00281095 | 0.00779324 |
| ENSECAG00000016307 | 3.03296515 | 0.00234626 | 0.00668316 |
| ENSECAG00000007598 | 3.03229886 | 1.47E-11 | 2.97E-10 |
| ENSECAG00000023889 | 3.03133587 | 0.00155952 | 0.00466161 |
| ENSECAG00000024640 | 3.03100186 | 3.32E-05 | 0.00015786 |
| ENSECAG00000038787 | 3.0308228 | 0.00581393 | 0.01467835 |
| ENSECAG00000021073 | 3.02705077 | 0.00119798 | 0.00370008 |
| ENSECAG00000043461 | 3.01769871 | 3.67E-16 | 1.60E-14 |
| ENSECAG00000017782 | 3.01491949 | 5.94E-13 | 1.54E-11 |
| ENSECAG00000000607 | 2.99815881 | 3.75E-09 | 4.72E-08 |
| ENSECAG00000059292 | 2.99485395 | 0.00025047 | 0.00093988 |
| ENSECAG00000023301 | 2.99402944 | 5.06E-49 | 2.83E-45 |
| ENSECAG00000010953 | 2.99245673 | 3.00E-08 | 3.02E-07 |
| ENSECAG00000008499 | 2.98901262 | 6.39E-25 | 1.06E-22 |
| ENSECAG00000013528 | 2.98672406 | 2.72E-08 | 2.77E-07 |
| ENSECAG00000048459 | 2.98656694 | 0.01344689 | 0.03008453 |
| ENSECAG00000031935 | 2.98301379 | 0.006586 | 0.01633778 |
| ENSECAG00000012388 | 2.9827232 | 8.00E-14 | 2.38E-12 |
| ENSECAG00000043859 | 2.98209339 | 0.00016052 | 0.0006353 |
| ENSECAG00000010967 | 2.98119139 | 1.25E-10 | 2.14E-09 |
| ENSECAG00000030957 | 2.97665865 | 0.01339896 | 0.0299893 |
| ENSECAG00000022141 | 2.97534295 | 3.08E-09 | 3.96E-08 |
| ENSECAG00000009154 | 2.97509049 | 0.01496534 | 0.03299935 |
| ENSECAG00000044665 | 2.96983066 | 0.00633537 | 0.01581967 |
| ENSECAG00000010500 | 2.96964387 | 4.32E-16 | 1.84E-14 |
| ENSECAG00000011187 | 2.96839563 | 1.62E-09 | 2.22E-08 |
| ENSECAG00000056292 | 2.9650922 | 0.00010044 | 0.00042293 |
| ENSECAG00000049916 | 2.96338642 | 4.56E-06 | 2.68E-05 |
| ENSECAG00000020030 | 2.96297043 | 0.00880915 | 0.02097408 |
| ENSECAG00000038005 | 2.9615255 | 5.10E-06 | 2.96E-05 |
| ENSECAG00000046976 | 2.95191551 | 0.00048662 | 0.00167575 |
| ENSECAG00000017885 | 2.95022715 | 3.46E-06 | 2.10E-05 |
| ENSECAG00000048115 | 2.94271211 | 0.00432603 | 0.01135951 |
| ENSECAG00000003551 | 2.94196684 | 5.18E-07 | 3.88E-06 |
| ENSECAG00000024485 | 2.94134679 | 0.00042164 | 0.00148144 |
| ENSECAG00000044127 | 2.93888239 | 1.70E-05 | 8.67E-05 |
| ENSECAG00000004587 | 2.93836093 | 1.42E-09 | 1.96E-08 |
| ENSECAG00000022463 | 2.93317813 | 0.00329265 | 0.00895431 |
| ENSECAG00000012803 | 2.93292819 | 0.00394801 | 0.0105014 |
| ENSECAG00000052839 | 2.93061376 | 0.0041513 | 0.01094701 |
| ENSECAG00000051461 | 2.93028346 | 0.02107731 | 0.04426216 |
| ENSECAG00000059993 | 2.92993294 | 0.01200497 | 0.02729857 |
| ENSECAG00000022686 | 2.92969479 | 6.18E-07 | 4.52E-06 |
| ENSECAG00000028496 | 2.92847578 | 0.00436358 | 0.01144332 |
| ENSECAG00000024687 | 2.92638748 | 9.54E-15 | 3.36E-13 |
| ENSECAG00000057903 | 2.92117557 | 6.22E-05 | 0.00027702 |
| ENSECAG00000030543 | 2.91870892 | 0.01168985 | 0.02672094 |
| ENSECAG00000048793 | 2.9187024 | 0.00030023 | 0.00110144 |
| ENSECAG00000002264 | 2.91561801 | 0.00209411 | 0.00604744 |
| ENSECAG00000055803 | 2.91266153 | 0.02424581 | 0.04981123 |
| ENSECAG00000004085 | 2.91251332 | 1.26E-13 | 3.61E-12 |
| ENSECAG00000034282 | 2.91005908 | 1.44E-08 | 1.57E-07 |
| ENSECAG00000021361 | 2.908263 | 0.00036595 | 0.00130757 |
| ENSECAG00000018964 | 2.9045951 | 0.0054667 | 0.01392601 |
| ENSECAG00000056018 | 2.90391105 | 0.00196722 | 0.00573134 |
| ENSECAG00000021019 | 2.90113696 | 1.21E-20 | 1.06E-18 |
| ENSECAG00000007331 | 2.89826351 | 3.87E-05 | 0.00018118 |
| ENSECAG00000012861 | 2.89689287 | 3.76E-17 | 1.95E-15 |
| ENSECAG00000048110 | 2.89297103 | 2.25E-07 | 1.84E-06 |
| ENSECAG00000051098 | 2.88898033 | 4.40E-05 | 0.00020301 |
| ENSECAG00000015104 | 2.88709472 | 0.00104928 | 0.00329548 |
| ENSECAG00000044851 | 2.88678583 | 1.52E-11 | 3.06E-10 |
| ENSECAG00000049290 | 2.88503771 | 1.05E-14 | 3.66E-13 |
| ENSECAG00000033234 | 2.88406346 | 1.92E-05 | 9.69E-05 |
| ENSECAG00000031605 | 2.88226373 | 3.98E-07 | 3.05E-06 |
| ENSECAG00000013806 | 2.88202635 | 5.19E-06 | 3.00E-05 |
| ENSECAG00000031235 | 2.88095714 | 9.40E-06 | 5.11E-05 |
| ENSECAG00000053152 | 2.8788457 | 0.00308706 | 0.0084466 |
| ENSECAG00000007324 | 2.87802112 | 0.00445652 | 0.01165013 |
| ENSECAG00000007077 | 2.87385695 | 1.50E-11 | 3.02E-10 |
| ENSECAG00000014675 | 2.87293386 | 5.27E-21 | 4.76E-19 |
| ENSECAG00000015711 | 2.86440424 | 0.00070921 | 0.00233232 |
| ENSECAG00000020789 | 2.86386561 | 2.34E-05 | 0.00011564 |
| ENSECAG00000052971 | 2.86165084 | 0.00642667 | 0.01600473 |
| ENSECAG00000022127 | 2.85551315 | 2.63E-08 | 2.70E-07 |
| ENSECAG00000004464 | 2.8553079 | 3.29E-08 | 3.28E-07 |
| ENSECAG00000047602 | 2.85362948 | 0.01752742 | 0.03779117 |
| ENSECAG00000037499 | 2.85318485 | 4.51E-06 | 2.65E-05 |
| ENSECAG00000045488 | 2.85214391 | 0.01935511 | 0.04112478 |
| ENSECAG00000055428 | 2.85156357 | 0.02255942 | 0.04685898 |
| ENSECAG00000023694 | 2.84660303 | 8.75E-05 | 0.00037429 |
| ENSECAG00000024563 | 2.84426958 | 2.15E-05 | 0.00010682 |
| ENSECAG00000059250 | 2.84110875 | 4.15E-37 | 3.71E-34 |
| ENSECAG00000048588 | 2.84109057 | 1.97E-06 | 1.28E-05 |
| ENSECAG00000000296 | 2.84034281 | 4.17E-10 | 6.36E-09 |
| ENSECAG00000056017 | 2.83969651 | 7.63E-07 | 5.45E-06 |
| ENSECAG00000013981 | 2.83403533 | 2.20E-08 | 2.31E-07 |
| ENSECAG00000045595 | 2.82572074 | 6.45E-11 | 1.16E-09 |
| ENSECAG00000033332 | 2.82258188 | 2.82E-10 | 4.47E-09 |
| ENSECAG00000008823 | 2.81992599 | 3.62E-21 | 3.39E-19 |
| ENSECAG00000011790 | 2.8194991 | 2.82E-35 | 1.80E-32 |
| ENSECAG00000058124 | 2.81884227 | 0.01191188 | 0.0271449 |
| ENSECAG00000022231 | 2.81728472 | 1.04E-05 | 5.59E-05 |
| ENSECAG00000006833 | 2.8053277 | 1.05E-17 | 6.02E-16 |
| ENSECAG00000030839 | 2.80480351 | 0.00221817 | 0.00636537 |
| ENSECAG00000020258 | 2.80281814 | 5.86E-13 | 1.52E-11 |
| ENSECAG00000023120 | 2.79700804 | 7.89E-08 | 7.16E-07 |
| ENSECAG00000042522 | 2.79374451 | 0.00335874 | 0.00912184 |
| ENSECAG00000036983 | 2.78995908 | 7.42E-05 | 0.00032436 |
| ENSECAG00000054450 | 2.78989316 | 0.00016904 | 0.00066505 |
| ENSECAG00000014891 | 2.78650351 | 5.76E-08 | 5.39E-07 |
| ENSECAG00000026235 | 2.78003432 | 1.41E-08 | 1.55E-07 |
| ENSECAG00000042491 | 2.78003136 | 0.01223544 | 0.02773797 |
| ENSECAG00000024753 | 2.77780742 | 2.02E-09 | 2.72E-08 |
| ENSECAG00000052245 | 2.77754808 | 7.77E-12 | 1.64E-10 |
| ENSECAG00000016631 | 2.77514725 | 0.02355367 | 0.04857238 |
| ENSECAG00000038342 | 2.77344238 | 0.00011104 | 0.00046194 |
| ENSECAG00000008371 | 2.76994745 | 0.00161576 | 0.00480769 |
| ENSECAG00000044248 | 2.76803409 | 0.01713733 | 0.03705736 |
| ENSECAG00000044632 | 2.7675389 | 0.00111696 | 0.00347578 |
| ENSECAG00000024520 | 2.76588585 | 3.90E-34 | 2.18E-31 |
| ENSECAG00000052405 | 2.76407016 | 8.48E-08 | 7.64E-07 |
| ENSECAG00000022273 | 2.76234214 | 1.11E-06 | 7.63E-06 |
| ENSECAG00000023864 | 2.76098184 | 1.28E-07 | 1.11E-06 |
| ENSECAG00000023729 | 2.75928323 | 0.00034432 | 0.00124244 |
| ENSECAG00000050417 | 2.75883706 | 0.00453627 | 0.01183924 |
| ENSECAG00000056747 | 2.75782656 | 0.00292624 | 0.0080758 |
| ENSECAG00000008092 | 2.75383198 | 6.21E-32 | 2.62E-29 |
| ENSECAG00000028902 | 2.75266519 | 0.00148254 | 0.00446361 |
| ENSECAG00000013951 | 2.75148925 | 1.26E-07 | 1.10E-06 |
| ENSECAG00000055490 | 2.74968504 | 0.01145203 | 0.02626356 |
| ENSECAG00000049678 | 2.74887341 | 0.00040101 | 0.00141495 |
| ENSECAG00000058971 | 2.74880986 | 3.60E-12 | 7.97E-11 |
| ENSECAG00000048839 | 2.74743035 | 0.00743967 | 0.01813931 |
| ENSECAG00000028571 | 2.73820428 | 9.69E-10 | 1.38E-08 |
| ENSECAG00000006998 | 2.737939 | 7.72E-09 | 9.02E-08 |
| ENSECAG00000001949 | 2.73533329 | 0.0013408 | 0.00408974 |
| ENSECAG00000031261 | 2.73243978 | 0.00913751 | 0.02163224 |
| ENSECAG00000048539 | 2.7310275 | 0.00944786 | 0.02226555 |
| ENSECAG00000004871 | 2.73003996 | 5.45E-31 | 2.14E-28 |
| ENSECAG00000017084 | 2.72996289 | 5.35E-12 | 1.16E-10 |
| ENSECAG00000000516 | 2.72821405 | 7.09E-37 | 6.10E-34 |
| ENSECAG00000046399 | 2.72597928 | 0.00026319 | 0.00098301 |
| ENSECAG00000034061 | 2.72537103 | 0.00131144 | 0.0040139 |
| ENSECAG00000053532 | 2.72041869 | 0.00126177 | 0.003873 |
| ENSECAG00000039532 | 2.7173064 | 0.0073481 | 0.01795328 |
| ENSECAG00000052401 | 2.71628131 | 2.24E-17 | 1.22E-15 |
| ENSECAG00000058040 | 2.71576896 | 7.06E-05 | 0.00030976 |
| ENSECAG00000016047 | 2.71509022 | 2.44E-10 | 3.92E-09 |
| ENSECAG00000054075 | 2.71204281 | 0.0014338 | 0.00433614 |
| ENSECAG00000012799 | 2.7106876 | 2.75E-12 | 6.22E-11 |
| ENSECAG00000023430 | 2.70939589 | 6.07E-12 | 1.30E-10 |
| ENSECAG00000024172 | 2.70190711 | 0.00010683 | 0.00044653 |
| ENSECAG00000009417 | 2.69886117 | 0.01870215 | 0.03995316 |
| ENSECAG00000022456 | 2.69728628 | 0.0120776 | 0.02743303 |
| ENSECAG00000009925 | 2.69705993 | 3.41E-11 | 6.45E-10 |
| ENSECAG00000013439 | 2.69557309 | 1.52E-44 | 3.39E-41 |
| ENSECAG00000044882 | 2.69548974 | 0.00295785 | 0.00815297 |
| ENSECAG00000016992 | 2.69523041 | 0.00017705 | 0.00069252 |
| ENSECAG00000017922 | 2.69104164 | 3.42E-17 | 1.79E-15 |
| ENSECAG00000047227 | 2.68910556 | 3.25E-06 | 1.99E-05 |
| ENSECAG00000011750 | 2.68737066 | 7.39E-06 | 4.12E-05 |
| ENSECAG00000044703 | 2.68625071 | 1.12E-09 | 1.57E-08 |
| ENSECAG00000057294 | 2.68389288 | 0.00151732 | 0.00455727 |
| ENSECAG00000040919 | 2.68268709 | 0.00178117 | 0.00524469 |
| ENSECAG00000020433 | 2.6815953 | 4.78E-24 | 7.08E-22 |
| ENSECAG00000035439 | 2.67996689 | 5.68E-18 | 3.40E-16 |
| ENSECAG00000053848 | 2.67990896 | 0.01172932 | 0.02679349 |
| ENSECAG00000011893 | 2.67933703 | 4.45E-08 | 4.30E-07 |
| ENSECAG00000024669 | 2.67743144 | 1.63E-22 | 1.80E-20 |
| ENSECAG00000017181 | 2.67222811 | 0.00011933 | 0.00049116 |
| ENSECAG00000007883 | 2.67080098 | 2.07E-10 | 3.37E-09 |
| ENSECAG00000006195 | 2.66624449 | 1.96E-06 | 1.27E-05 |
| ENSECAG00000015342 | 2.6606154 | 0.0117457 | 0.02682366 |
| ENSECAG00000029465 | 2.65795562 | 0.00108054 | 0.00337707 |
| ENSECAG00000050849 | 2.652983 | 0.00012344 | 0.00050528 |
| ENSECAG00000011449 | 2.65238295 | 1.75E-08 | 1.88E-07 |
| ENSECAG00000028576 | 2.65177566 | 0.00266641 | 0.00746007 |
| ENSECAG00000035115 | 2.64890444 | 0.00607005 | 0.01523425 |
| ENSECAG00000012252 | 2.64772027 | 3.36E-21 | 3.17E-19 |
| ENSECAG00000005381 | 2.64615178 | 1.97E-16 | 9.03E-15 |
| ENSECAG00000059767 | 2.64534567 | 0.00481054 | 0.01247335 |
| ENSECAG00000055189 | 2.64446743 | 7.10E-09 | 8.38E-08 |
| ENSECAG00000007731 | 2.63706024 | 4.76E-05 | 0.0002177 |
| ENSECAG00000041909 | 2.63523281 | 0.01772935 | 0.03818971 |
| ENSECAG00000024835 | 2.63147025 | 0.0023369 | 0.00665821 |
| ENSECAG00000005046 | 2.626069 | 0.0178961 | 0.03847473 |
| ENSECAG00000024312 | 2.6253638 | 1.14E-07 | 9.96E-07 |
| ENSECAG00000036937 | 2.62385628 | 1.58E-07 | 1.34E-06 |
| ENSECAG00000057404 | 2.62222618 | 0.00147199 | 0.00443721 |
| ENSECAG00000006776 | 2.61972569 | 1.94E-12 | 4.54E-11 |
| ENSECAG00000050133 | 2.61920927 | 1.89E-11 | 3.73E-10 |
| ENSECAG00000024746 | 2.61361719 | 1.92E-17 | 1.05E-15 |
| ENSECAG00000058282 | 2.61161554 | 1.21E-07 | 1.06E-06 |
| ENSECAG00000028292 | 2.60829634 | 1.51E-12 | 3.62E-11 |
| ENSECAG00000054009 | 2.60822521 | 1.55E-07 | 1.32E-06 |
| ENSECAG00000042356 | 2.6074725 | 1.18E-15 | 4.83E-14 |
| ENSECAG00000040426 | 2.60652817 | 0.01271259 | 0.02864821 |
| ENSECAG00000018734 | 2.60614956 | 3.48E-05 | 0.0001644 |
| ENSECAG00000054245 | 2.60552498 | 9.32E-07 | 6.53E-06 |
| ENSECAG00000042282 | 2.60469743 | 1.77E-06 | 1.16E-05 |
| ENSECAG00000019099 | 2.60037915 | 2.80E-09 | 3.64E-08 |
| ENSECAG00000019596 | 2.60003037 | 6.61E-10 | 9.76E-09 |
| ENSECAG00000018306 | 2.59905645 | 2.57E-17 | 1.38E-15 |
| ENSECAG00000045659 | 2.59663963 | 0.00193584 | 0.00564729 |
| ENSECAG00000016277 | 2.59388258 | 3.36E-17 | 1.76E-15 |
| ENSECAG00000057783 | 2.59253934 | 1.69E-06 | 1.11E-05 |
| ENSECAG00000048080 | 2.59216566 | 3.42E-30 | 1.11E-27 |
| ENSECAG00000011895 | 2.58920247 | 0.00227268 | 0.00650426 |
| ENSECAG00000007854 | 2.58858582 | 0.00266731 | 0.00746165 |
| ENSECAG00000059379 | 2.58794159 | 0.02013845 | 0.0425545 |
| ENSECAG00000024756 | 2.58440895 | 7.96E-34 | 4.14E-31 |
| ENSECAG00000030025 | 2.58434034 | 2.60E-05 | 0.00012654 |
| ENSECAG00000013089 | 2.58226099 | 8.22E-24 | 1.17E-21 |
| ENSECAG00000022258 | 2.58060835 | 7.47E-06 | 4.16E-05 |
| ENSECAG00000058013 | 2.57832868 | 7.68E-15 | 2.73E-13 |
| ENSECAG00000010003 | 2.57698261 | 1.22E-23 | 1.66E-21 |
| ENSECAG00000034341 | 2.57135437 | 0.01596795 | 0.03492072 |
| ENSECAG00000059680 | 2.57031798 | 5.29E-05 | 0.0002391 |
| ENSECAG00000057103 | 2.56963216 | 3.13E-06 | 1.92E-05 |
| ENSECAG00000044406 | 2.56643295 | 0.018503 | 0.03960042 |
| ENSECAG00000045225 | 2.56486793 | 2.38E-05 | 0.00011725 |
| ENSECAG00000012627 | 2.56281371 | 1.28E-27 | 3.07E-25 |
| ENSECAG00000019011 | 2.56209059 | 1.54E-12 | 3.68E-11 |
| ENSECAG00000053529 | 2.561061 | 0.01621163 | 0.03536019 |
| ENSECAG00000041410 | 2.55441044 | 2.31E-16 | 1.04E-14 |
| ENSECAG00000047836 | 2.55331264 | 0.00048717 | 0.00167688 |
| ENSECAG00000015270 | 2.5512381 | 2.64E-07 | 2.11E-06 |
| ENSECAG00000019417 | 2.55115766 | 0.00553886 | 0.01408183 |
| ENSECAG00000013712 | 2.54920461 | 0.00223743 | 0.00641405 |
| ENSECAG00000057849 | 2.54681788 | 5.12E-26 | 1.04E-23 |
| ENSECAG00000022919 | 2.54675958 | 6.05E-07 | 4.44E-06 |
| ENSECAG00000008606 | 2.54429777 | 9.42E-07 | 6.60E-06 |
| ENSECAG00000058697 | 2.54427117 | 0.0234637 | 0.0484226 |
| ENSECAG00000022699 | 2.54183266 | 0.00063099 | 0.00210763 |
| ENSECAG00000012814 | 2.53888976 | 0.0071173 | 0.01746383 |
| ENSECAG00000022869 | 2.5388304 | 3.06E-08 | 3.08E-07 |
| ENSECAG00000059312 | 2.53395715 | 1.20E-07 | 1.04E-06 |
| ENSECAG00000031528 | 2.53364324 | 1.83E-06 | 1.20E-05 |
| ENSECAG00000029260 | 2.531431 | 0.02244527 | 0.04666127 |
| ENSECAG00000023251 | 2.53057794 | 3.45E-13 | 9.27E-12 |
| ENSECAG00000028258 | 2.53020865 | 1.63E-05 | 8.31E-05 |
| ENSECAG00000009226 | 2.52915078 | 9.86E-05 | 0.0004156 |
| ENSECAG00000030155 | 2.5291312 | 3.13E-07 | 2.46E-06 |
| ENSECAG00000035361 | 2.52912337 | 3.99E-05 | 0.00018611 |
| ENSECAG00000049224 | 2.5218649 | 0.0084773 | 0.0203146 |
| ENSECAG00000052745 | 2.51948942 | 0.00138651 | 0.00421382 |
| ENSECAG00000024588 | 2.51500055 | 1.25E-05 | 6.61E-05 |
| ENSECAG00000035500 | 2.51435574 | 0.00026795 | 0.00099774 |
| ENSECAG00000059484 | 2.51420108 | 0.00015457 | 0.00061501 |
| ENSECAG00000045032 | 2.50462325 | 5.21E-08 | 4.94E-07 |
| ENSECAG00000011930 | 2.49707546 | 0.02055987 | 0.04333435 |
| ENSECAG00000044704 | 2.49537983 | 0.01560492 | 0.03424408 |
| ENSECAG00000021766 | 2.49235292 | 1.79E-17 | 9.92E-16 |
| ENSECAG00000055300 | 2.49231953 | 0.00156972 | 0.00468881 |
| ENSECAG00000045650 | 2.48834355 | 0.01376455 | 0.03071526 |
| ENSECAG00000012887 | 2.48760932 | 0.00171267 | 0.00506838 |
| ENSECAG00000024406 | 2.48582755 | 1.75E-06 | 1.15E-05 |
| ENSECAG00000022398 | 2.48548033 | 4.55E-08 | 4.38E-07 |
| ENSECAG00000037707 | 2.48318753 | 1.57E-09 | 2.15E-08 |
| ENSECAG00000050527 | 2.47425957 | 0.01145955 | 0.02627759 |
| ENSECAG00000059182 | 2.47169658 | 0.01698891 | 0.03677201 |
| ENSECAG00000021617 | 2.47127396 | 9.86E-29 | 2.69E-26 |
| ENSECAG00000021165 | 2.47060595 | 0.00011615 | 0.00047949 |
| ENSECAG00000040740 | 2.46994813 | 7.67E-13 | 1.93E-11 |
| ENSECAG00000020303 | 2.4692826 | 1.09E-29 | 3.39E-27 |
| ENSECAG00000005678 | 2.4642055 | 3.62E-08 | 3.57E-07 |
| ENSECAG00000019830 | 2.4638385 | 5.46E-06 | 3.14E-05 |
| ENSECAG00000022279 | 2.46335584 | 8.04E-20 | 6.49E-18 |
| ENSECAG00000056673 | 2.46308992 | 0.00248236 | 0.00700601 |
| ENSECAG00000045796 | 2.46276599 | 0.00159798 | 0.0047624 |
| ENSECAG00000016961 | 2.46182678 | 2.07E-07 | 1.71E-06 |
| ENSECAG00000021052 | 2.45841101 | 1.19E-05 | 6.28E-05 |
| ENSECAG00000052490 | 2.45752554 | 0.00374337 | 0.01002508 |
| ENSECAG00000047862 | 2.45502043 | 0.00204372 | 0.00592181 |
| ENSECAG00000050469 | 2.45495517 | 4.47E-05 | 0.00020614 |
| ENSECAG00000018120 | 2.45429033 | 0.00034875 | 0.00125635 |
| ENSECAG00000047363 | 2.45000208 | 0.01678721 | 0.03640949 |
| ENSECAG00000056977 | 2.44859725 | 0.0100498 | 0.02346388 |
| ENSECAG00000008152 | 2.43683316 | 8.44E-09 | 9.74E-08 |
| ENSECAG00000018125 | 2.43234396 | 3.25E-07 | 2.55E-06 |
| ENSECAG00000019398 | 2.4313756 | 1.37E-11 | 2.78E-10 |
| ENSECAG00000033245 | 2.43056185 | 3.42E-10 | 5.32E-09 |
| ENSECAG00000008836 | 2.42711741 | 1.57E-12 | 3.73E-11 |
| ENSECAG00000052096 | 2.42665149 | 9.81E-05 | 0.00041387 |
| ENSECAG00000031571 | 2.42021138 | 0.00298954 | 0.00822308 |
| ENSECAG00000036612 | 2.4201904 | 0.00042612 | 0.00149435 |
| ENSECAG00000047372 | 2.41776389 | 0.00263581 | 0.00738557 |
| ENSECAG00000014590 | 2.41598258 | 1.27E-06 | 8.60E-06 |
| ENSECAG00000009742 | 2.41567585 | 0.00432751 | 0.01136121 |
| ENSECAG00000009523 | 2.41371043 | 0.00101006 | 0.00319202 |
| ENSECAG00000022973 | 2.41348706 | 1.17E-05 | 6.21E-05 |
| ENSECAG00000045531 | 2.41206489 | 1.37E-05 | 7.15E-05 |
| ENSECAG00000004502 | 2.41015367 | 2.29E-18 | 1.44E-16 |
| ENSECAG00000009887 | 2.40914659 | 3.24E-16 | 1.43E-14 |
| ENSECAG00000045984 | 2.40884267 | 0.00016229 | 0.00064177 |
| ENSECAG00000049587 | 2.40744299 | 0.0012234 | 0.00377024 |
| ENSECAG00000052638 | 2.4067351 | 6.82E-10 | 1.00E-08 |
| ENSECAG00000015947 | 2.40203832 | 0.00888995 | 0.02114024 |
| ENSECAG00000043319 | 2.40168512 | 1.25E-07 | 1.09E-06 |
| ENSECAG00000013874 | 2.39868852 | 1.83E-11 | 3.63E-10 |
| ENSECAG00000056823 | 2.39821785 | 0.02202043 | 0.04591049 |
| ENSECAG00000017347 | 2.3967744 | 3.22E-12 | 7.20E-11 |
| ENSECAG00000006902 | 2.39265543 | 0.0048686 | 0.01259899 |
| ENSECAG00000008352 | 2.39252189 | 4.86E-24 | 7.15E-22 |
| ENSECAG00000008055 | 2.3910695 | 0.00016283 | 0.00064342 |
| ENSECAG00000029466 | 2.38612453 | 6.44E-07 | 4.69E-06 |
| ENSECAG00000054465 | 2.38588967 | 5.77E-06 | 3.31E-05 |
| ENSECAG00000058191 | 2.38488667 | 4.71E-06 | 2.75E-05 |
| ENSECAG00000014549 | 2.38449173 | 0.0118985 | 0.0271218 |
| ENSECAG00000016624 | 2.38162738 | 3.10E-09 | 3.98E-08 |
| ENSECAG00000020400 | 2.38075014 | 1.40E-06 | 9.43E-06 |
| ENSECAG00000020639 | 2.38048911 | 7.46E-13 | 1.88E-11 |
| ENSECAG00000016721 | 2.37834816 | 7.12E-13 | 1.81E-11 |
| ENSECAG00000032752 | 2.3782603 | 8.25E-05 | 0.00035529 |
| ENSECAG00000019098 | 2.37665045 | 4.04E-06 | 2.40E-05 |
| ENSECAG00000016075 | 2.37484686 | 0.00035114 | 0.00126355 |
| ENSECAG00000046266 | 2.37361709 | 2.18E-07 | 1.79E-06 |
| ENSECAG00000022680 | 2.37061113 | 0.00045897 | 0.00159355 |
| ENSECAG00000020170 | 2.36994308 | 3.02E-08 | 3.05E-07 |
| ENSECAG00000044297 | 2.36866549 | 1.58E-06 | 1.05E-05 |
| ENSECAG00000014259 | 2.36818532 | 1.21E-08 | 1.35E-07 |
| ENSECAG00000010531 | 2.36793656 | 1.23E-26 | 2.70E-24 |
| ENSECAG00000053020 | 2.36747459 | 0.00446278 | 0.01166376 |
| ENSECAG00000006429 | 2.36605854 | 6.02E-19 | 4.24E-17 |
| ENSECAG00000050145 | 2.36163291 | 8.15E-06 | 4.49E-05 |
| ENSECAG00000039591 | 2.36143762 | 9.23E-05 | 0.00039275 |
| ENSECAG00000052645 | 2.3605816 | 1.60E-07 | 1.36E-06 |
| ENSECAG00000059423 | 2.35880823 | 0.00410777 | 0.01085014 |
| ENSECAG00000048020 | 2.35666064 | 0.00241997 | 0.00685468 |
| ENSECAG00000004493 | 2.35654108 | 4.16E-14 | 1.32E-12 |
| ENSECAG00000046296 | 2.35430602 | 4.48E-21 | 4.08E-19 |
| ENSECAG00000056686 | 2.35248056 | 0.0006885 | 0.00227492 |
| ENSECAG00000052280 | 2.3487217 | 0.00765047 | 0.01857829 |
| ENSECAG00000047818 | 2.34837711 | 0.00015265 | 0.00060814 |
| ENSECAG00000022390 | 2.34558598 | 5.03E-07 | 3.78E-06 |
| ENSECAG00000005409 | 2.34260636 | 4.35E-10 | 6.61E-09 |
| ENSECAG00000019739 | 2.3397316 | 1.53E-22 | 1.71E-20 |
| ENSECAG00000004548 | 2.33772831 | 4.99E-08 | 4.75E-07 |
| ENSECAG00000004408 | 2.33752182 | 2.36E-14 | 7.70E-13 |
| ENSECAG00000010705 | 2.3373121 | 0.01538876 | 0.03382954 |
| ENSECAG00000002389 | 2.33427856 | 1.08E-06 | 7.46E-06 |
| ENSECAG00000055277 | 2.33394993 | 0.00032313 | 0.00117562 |
| ENSECAG00000026963 | 2.33276333 | 1.05E-06 | 7.25E-06 |
| ENSECAG00000022731 | 2.33186446 | 3.76E-18 | 2.30E-16 |
| ENSECAG00000036115 | 2.33097657 | 2.83E-06 | 1.76E-05 |
| ENSECAG00000057115 | 2.32680275 | 0.01858632 | 0.03974068 |
| ENSECAG00000055772 | 2.32500827 | 0.01580549 | 0.03461284 |
| ENSECAG00000058599 | 2.32123005 | 3.78E-07 | 2.92E-06 |
| ENSECAG00000043642 | 2.32105527 | 1.98E-14 | 6.55E-13 |
| ENSECAG00000059420 | 2.31752202 | 0.00637066 | 0.01589003 |
| ENSECAG00000021367 | 2.31144467 | 1.81E-11 | 3.60E-10 |
| ENSECAG00000008913 | 2.31112562 | 1.72E-30 | 6.01E-28 |
| ENSECAG00000022890 | 2.31017107 | 0.00293297 | 0.00809038 |
| ENSECAG00000023405 | 2.30874935 | 3.46E-14 | 1.11E-12 |
| ENSECAG00000020937 | 2.30820548 | 6.99E-07 | 5.05E-06 |
| ENSECAG00000023442 | 2.30583004 | 1.65E-07 | 1.39E-06 |
| ENSECAG00000038584 | 2.30326661 | 4.06E-23 | 4.98E-21 |
| ENSECAG00000007663 | 2.30319308 | 2.85E-10 | 4.50E-09 |
| ENSECAG00000051452 | 2.30276549 | 0.00352552 | 0.00950891 |
| ENSECAG00000003447 | 2.29844951 | 1.47E-06 | 9.82E-06 |
| ENSECAG00000055251 | 2.29810595 | 2.21E-11 | 4.30E-10 |
| ENSECAG00000013510 | 2.29755584 | 1.45E-20 | 1.25E-18 |
| ENSECAG00000011901 | 2.29622429 | 0.00060472 | 0.00203026 |
| ENSECAG00000018701 | 2.29580197 | 0.0001831 | 0.00071294 |
| ENSECAG00000019061 | 2.29479325 | 2.14E-05 | 0.00010678 |
| ENSECAG00000037319 | 2.29323038 | 0.00066193 | 0.00219655 |
| ENSECAG00000018841 | 2.29173299 | 4.10E-06 | 2.44E-05 |
| ENSECAG00000020892 | 2.29108609 | 3.59E-10 | 5.57E-09 |
| ENSECAG00000024282 | 2.28693279 | 5.94E-06 | 3.39E-05 |
| ENSECAG00000054591 | 2.28491363 | 4.53E-09 | 5.61E-08 |
| ENSECAG00000025126 | 2.28289351 | 1.04E-17 | 5.94E-16 |
| ENSECAG00000023281 | 2.28240161 | 2.82E-12 | 6.37E-11 |
| ENSECAG00000021047 | 2.28224014 | 1.95E-25 | 3.61E-23 |
| ENSECAG00000015500 | 2.28132552 | 1.22E-06 | 8.32E-06 |
| ENSECAG00000023303 | 2.28061416 | 6.55E-09 | 7.81E-08 |
| ENSECAG00000050868 | 2.28037035 | 0.0002798 | 0.00103517 |
| ENSECAG00000053823 | 2.28024621 | 6.76E-05 | 0.00029773 |
| ENSECAG00000053465 | 2.27780658 | 1.58E-06 | 1.05E-05 |
| ENSECAG00000010356 | 2.27704031 | 7.66E-06 | 4.25E-05 |
| ENSECAG00000055055 | 2.27507123 | 0.01366365 | 0.03050839 |
| ENSECAG00000056991 | 2.27268865 | 0.00022828 | 0.00086591 |
| ENSECAG00000010377 | 2.27210786 | 2.84E-07 | 2.26E-06 |
| ENSECAG00000003201 | 2.27097742 | 1.03E-05 | 5.55E-05 |
| ENSECAG00000056462 | 2.2691923 | 0.0002753 | 0.00102122 |
| ENSECAG00000019036 | 2.26708353 | 6.23E-06 | 3.53E-05 |
| ENSECAG00000046791 | 2.26604584 | 1.71E-05 | 8.69E-05 |
| ENSECAG00000025038 | 2.25701803 | 5.23E-11 | 9.58E-10 |
| ENSECAG00000018176 | 2.25687179 | 8.74E-22 | 9.21E-20 |
| ENSECAG00000053421 | 2.2558353 | 4.37E-07 | 3.32E-06 |
| ENSECAG00000038588 | 2.25573224 | 8.40E-16 | 3.48E-14 |
| ENSECAG00000011240 | 2.2550747 | 9.05E-06 | 4.94E-05 |
| ENSECAG00000046268 | 2.25412107 | 0.00505129 | 0.01300266 |
| ENSECAG00000047309 | 2.25401684 | 4.22E-12 | 9.27E-11 |
| ENSECAG00000019371 | 2.25297742 | 7.28E-06 | 4.07E-05 |
| ENSECAG00000018674 | 2.2507484 | 0.00014148 | 0.00056903 |
| ENSECAG00000010054 | 2.25060107 | 2.90E-06 | 1.80E-05 |
| ENSECAG00000016866 | 2.24999889 | 2.06E-30 | 6.96E-28 |
| ENSECAG00000004055 | 2.24884318 | 2.25E-22 | 2.46E-20 |
| ENSECAG00000009829 | 2.24779672 | 0.00386904 | 0.01032576 |
| ENSECAG00000059199 | 2.24775619 | 0.00965047 | 0.02267131 |
| ENSECAG00000054832 | 2.24739129 | 1.00E-05 | 5.42E-05 |
| ENSECAG00000014126 | 2.24721671 | 0.01244987 | 0.02814984 |
| ENSECAG00000039848 | 2.24437325 | 8.85E-10 | 1.27E-08 |
| ENSECAG00000024325 | 2.24069785 | 4.39E-07 | 3.34E-06 |
| ENSECAG00000017094 | 2.24044766 | 7.99E-28 | 1.98E-25 |
| ENSECAG00000024601 | 2.24002933 | 6.14E-10 | 9.14E-09 |
| ENSECAG00000028822 | 2.2379117 | 7.41E-07 | 5.32E-06 |
| ENSECAG00000040040 | 2.23517376 | 0.00087791 | 0.00282149 |
| ENSECAG00000022737 | 2.23302789 | 1.27E-08 | 1.41E-07 |
| ENSECAG00000005787 | 2.23266248 | 9.37E-08 | 8.35E-07 |
| ENSECAG00000006442 | 2.23163344 | 3.68E-15 | 1.39E-13 |
| ENSECAG00000016696 | 2.23113377 | 5.67E-06 | 3.25E-05 |
| ENSECAG00000000931 | 2.22919118 | 7.99E-14 | 2.38E-12 |
| ENSECAG00000053122 | 2.227934 | 0.00150302 | 0.00451855 |
| ENSECAG00000021094 | 2.22770813 | 0.00702587 | 0.01727744 |
| ENSECAG00000045550 | 2.22758367 | 1.46E-09 | 2.01E-08 |
| ENSECAG00000008932 | 2.22741501 | 1.55E-06 | 1.03E-05 |
| ENSECAG00000038417 | 2.22725033 | 2.46E-07 | 1.99E-06 |
| ENSECAG00000002620 | 2.22710696 | 9.46E-05 | 0.00040068 |
| ENSECAG00000037735 | 2.22699018 | 0.00018168 | 0.00070801 |
| ENSECAG00000040464 | 2.22592376 | 7.96E-25 | 1.29E-22 |
| ENSECAG00000011979 | 2.22501357 | 9.06E-19 | 6.14E-17 |
| ENSECAG00000002144 | 2.2234069 | 0.00457042 | 0.01192002 |
| ENSECAG00000028304 | 2.22257814 | 0.00362957 | 0.00975892 |
| ENSECAG00000019838 | 2.22248087 | 4.77E-17 | 2.43E-15 |
| ENSECAG00000000495 | 2.22233289 | 7.19E-26 | 1.40E-23 |
| ENSECAG00000030142 | 2.21946876 | 2.21E-05 | 0.00010958 |
| ENSECAG00000006175 | 2.21898628 | 0.00016427 | 0.00064879 |
| ENSECAG00000002887 | 2.21235715 | 0.00040811 | 0.00143733 |
| ENSECAG00000059535 | 2.20928081 | 0.00681874 | 0.01682549 |
| ENSECAG00000052567 | 2.20399828 | 0.01858631 | 0.03974068 |
| ENSECAG00000020808 | 2.20195396 | 1.07E-10 | 1.87E-09 |
| ENSECAG00000012493 | 2.20035694 | 1.33E-10 | 2.25E-09 |
| ENSECAG00000005159 | 2.19761403 | 2.78E-06 | 1.73E-05 |
| ENSECAG00000035435 | 2.19348288 | 1.98E-17 | 1.09E-15 |
| ENSECAG00000034729 | 2.19343623 | 0.01314173 | 0.02952595 |
| ENSECAG00000012439 | 2.19250612 | 0.00467019 | 0.01215469 |
| ENSECAG00000049732 | 2.19032277 | 0.02222866 | 0.04628731 |
| ENSECAG00000009285 | 2.18967417 | 0.00866686 | 0.02070448 |
| ENSECAG00000008871 | 2.18909907 | 9.45E-07 | 6.62E-06 |
| ENSECAG00000058409 | 2.188878 | 6.37E-10 | 9.43E-09 |
| ENSECAG00000030620 | 2.18885473 | 5.19E-07 | 3.89E-06 |
| ENSECAG00000028008 | 2.18594844 | 1.42E-10 | 2.38E-09 |
| ENSECAG00000017678 | 2.18572252 | 1.06E-19 | 8.42E-18 |
| ENSECAG00000019322 | 2.18569273 | 4.69E-07 | 3.54E-06 |
| ENSECAG00000040180 | 2.18539595 | 2.96E-09 | 3.82E-08 |
| ENSECAG00000008358 | 2.18518212 | 1.47E-16 | 6.81E-15 |
| ENSECAG00000020721 | 2.18460258 | 8.56E-12 | 1.79E-10 |
| ENSECAG00000024399 | 2.18261849 | 0.00019206 | 0.00074277 |
| ENSECAG00000026033 | 2.18246329 | 0.00060907 | 0.00204328 |
| ENSECAG00000049075 | 2.18169315 | 0.00015876 | 0.00062953 |
| ENSECAG00000008303 | 2.17921269 | 5.34E-17 | 2.69E-15 |
| ENSECAG00000007668 | 2.17697863 | 2.34E-08 | 2.43E-07 |
| ENSECAG00000000835 | 2.17583633 | 2.23E-12 | 5.15E-11 |
| ENSECAG00000051695 | 2.17468317 | 0.0209543 | 0.04404108 |
| ENSECAG00000036551 | 2.17302997 | 0.00414009 | 0.01092132 |
| ENSECAG00000034447 | 2.1727405 | 1.86E-09 | 2.52E-08 |
| ENSECAG00000020505 | 2.17209313 | 1.05E-14 | 3.66E-13 |
| ENSECAG00000020887 | 2.17118161 | 6.25E-15 | 2.26E-13 |
| ENSECAG00000005856 | 2.17082351 | 8.92E-06 | 4.88E-05 |
| ENSECAG00000002370 | 2.17077121 | 1.30E-40 | 1.71E-37 |
| ENSECAG00000052716 | 2.16955257 | 0.01717289 | 0.03712389 |
| ENSECAG00000049780 | 2.16893985 | 0.01017805 | 0.0237284 |
| ENSECAG00000009462 | 2.16736925 | 8.29E-14 | 2.46E-12 |
| ENSECAG00000020778 | 2.16683465 | 1.70E-09 | 2.32E-08 |
| ENSECAG00000059067 | 2.16583436 | 1.38E-06 | 9.31E-06 |
| ENSECAG00000021296 | 2.16374175 | 4.76E-25 | 7.94E-23 |
| ENSECAG00000009420 | 2.16371573 | 3.00E-36 | 2.24E-33 |
| ENSECAG00000029588 | 2.16300552 | 2.53E-32 | 1.13E-29 |
| ENSECAG00000004251 | 2.16234232 | 1.03E-30 | 3.85E-28 |
| ENSECAG00000007300 | 2.1611318 | 1.71E-11 | 3.42E-10 |
| ENSECAG00000054273 | 2.1591078 | 0.00049384 | 0.0016954 |
| ENSECAG00000006881 | 2.15774478 | 4.29E-29 | 1.23E-26 |
| ENSECAG00000036892 | 2.15760591 | 0.01017902 | 0.0237284 |
| ENSECAG00000016805 | 2.15564374 | 7.30E-10 | 1.06E-08 |
| ENSECAG00000057566 | 2.1556211 | 0.0021039 | 0.00607255 |
| ENSECAG00000040326 | 2.15530433 | 1.48E-08 | 1.61E-07 |
| ENSECAG00000019904 | 2.15356602 | 9.49E-10 | 1.35E-08 |
| ENSECAG00000022791 | 2.15306406 | 5.11E-05 | 0.00023177 |
| ENSECAG00000035511 | 2.15144217 | 0.01832137 | 0.03927189 |
| ENSECAG00000024119 | 2.1502542 | 5.49E-18 | 3.30E-16 |
| ENSECAG00000024750 | 2.15021704 | 8.82E-25 | 1.42E-22 |
| ENSECAG00000023396 | 2.14975253 | 6.23E-26 | 1.25E-23 |
| ENSECAG00000051167 | 2.14936671 | 0.00010636 | 0.00044483 |
| ENSECAG00000000953 | 2.14569338 | 6.29E-23 | 7.52E-21 |
| ENSECAG00000019436 | 2.14405136 | 1.46E-19 | 1.14E-17 |
| ENSECAG00000047732 | 2.14336812 | 0.00400089 | 0.01061427 |
| ENSECAG00000000320 | 2.14230031 | 4.10E-45 | 1.02E-41 |
| ENSECAG00000023211 | 2.13949865 | 0.00015101 | 0.00060238 |
| ENSECAG00000049953 | 2.13904421 | 0.01214248 | 0.02755239 |
| ENSECAG00000032553 | 2.13786474 | 0.00757968 | 0.01842038 |
| ENSECAG00000059439 | 2.13602629 | 2.90E-13 | 7.90E-12 |
| ENSECAG00000010610 | 2.1360031 | 2.63E-50 | 2.94E-46 |
| ENSECAG00000024371 | 2.13474689 | 6.03E-30 | 1.92E-27 |
| ENSECAG00000046336 | 2.13376756 | 0.00886342 | 0.02108388 |
| ENSECAG00000059701 | 2.13359696 | 4.51E-11 | 8.38E-10 |
| ENSECAG00000048070 | 2.13331197 | 3.68E-07 | 2.85E-06 |
| ENSECAG00000056117 | 2.1316824 | 0.00223474 | 0.00640799 |
| ENSECAG00000056073 | 2.13032192 | 0.00134852 | 0.00410939 |
| ENSECAG00000051445 | 2.13030455 | 1.19E-05 | 6.32E-05 |
| ENSECAG00000050360 | 2.13024907 | 0.00224064 | 0.00642162 |
| ENSECAG00000030367 | 2.12953233 | 0.00012689 | 0.00051713 |
| ENSECAG00000004725 | 2.12869057 | 0.01077623 | 0.02491264 |
| ENSECAG00000022717 | 2.12854424 | 5.70E-47 | 2.12E-43 |
| ENSECAG00000000460 | 2.12831327 | 3.08E-07 | 2.43E-06 |
| ENSECAG00000026858 | 2.12766963 | 2.23E-34 | 1.31E-31 |
| ENSECAG00000060247 | 2.12711029 | 8.68E-17 | 4.19E-15 |
| ENSECAG00000005384 | 2.12683541 | 9.34E-12 | 1.95E-10 |
| ENSECAG00000024631 | 2.12594964 | 0.00010729 | 0.00044811 |
| ENSECAG00000053839 | 2.12523231 | 0.00468683 | 0.01219372 |
| ENSECAG00000034732 | 2.12492801 | 1.49E-10 | 2.49E-09 |
| ENSECAG00000016708 | 2.12390881 | 2.38E-16 | 1.07E-14 |
| ENSECAG00000016615 | 2.12375663 | 3.78E-13 | 1.01E-11 |
| ENSECAG00000015848 | 2.12343078 | 0.00069748 | 0.00229879 |
| ENSECAG00000008118 | 2.118988 | 4.56E-09 | 5.64E-08 |
| ENSECAG00000013721 | 2.11828067 | 0.00340792 | 0.00923855 |
| ENSECAG00000039891 | 2.11633672 | 5.78E-09 | 6.97E-08 |
| ENSECAG00000022992 | 2.11212335 | 0.0004786 | 0.00165169 |
| ENSECAG00000014728 | 2.11193013 | 5.84E-09 | 7.04E-08 |
| ENSECAG00000015611 | 2.11080042 | 2.39E-08 | 2.48E-07 |
| ENSECAG00000006745 | 2.10991674 | 7.55E-20 | 6.12E-18 |
| ENSECAG00000039479 | 2.10978823 | 3.71E-06 | 2.23E-05 |
| ENSECAG00000014703 | 2.10908839 | 1.93E-08 | 2.05E-07 |
| ENSECAG00000032864 | 2.108713 | 2.16E-25 | 3.96E-23 |
| ENSECAG00000016176 | 2.10632613 | 0.00011831 | 0.0004876 |
| ENSECAG00000015125 | 2.10627769 | 2.10E-09 | 2.82E-08 |
| ENSECAG00000032598 | 2.10532601 | 2.07E-07 | 1.71E-06 |
| ENSECAG00000032595 | 2.10353596 | 6.96E-10 | 1.02E-08 |
| ENSECAG00000008333 | 2.10338272 | 1.20E-11 | 2.45E-10 |
| ENSECAG00000049053 | 2.10283598 | 0.01899324 | 0.04048678 |
| ENSECAG00000019709 | 2.10211797 | 1.20E-40 | 1.67E-37 |
| ENSECAG00000024993 | 2.10144909 | 2.03E-07 | 1.68E-06 |
| ENSECAG00000002855 | 2.09863872 | 3.65E-23 | 4.56E-21 |
| ENSECAG00000018935 | 2.09625609 | 1.16E-09 | 1.62E-08 |
| ENSECAG00000020731 | 2.09529519 | 4.85E-18 | 2.92E-16 |
| ENSECAG00000012716 | 2.09490663 | 4.10E-12 | 9.02E-11 |
| ENSECAG00000011414 | 2.09274961 | 0.00498171 | 0.01284574 |
| ENSECAG00000045119 | 2.089979 | 0.00949912 | 0.02237456 |
| ENSECAG00000049949 | 2.08959082 | 0.00183879 | 0.0053973 |
| ENSECAG00000033140 | 2.08781894 | 0.01475361 | 0.03259681 |
| ENSECAG00000010182 | 2.08699947 | 9.24E-11 | 1.62E-09 |
| ENSECAG00000006765 | 2.08662357 | 2.08E-24 | 3.23E-22 |
| ENSECAG00000047128 | 2.0841855 | 0.00131865 | 0.00403426 |
| ENSECAG00000018619 | 2.08364079 | 1.98E-11 | 3.89E-10 |
| ENSECAG00000007338 | 2.08284411 | 4.56E-33 | 2.21E-30 |
| ENSECAG00000006296 | 2.08267956 | 0.00011078 | 0.00046123 |
| ENSECAG00000014155 | 2.08181166 | 0.00276975 | 0.00769716 |
| ENSECAG00000010721 | 2.08176839 | 7.57E-07 | 5.42E-06 |
| ENSECAG00000022828 | 2.08005784 | 0.02413535 | 0.04962081 |
| ENSECAG00000018229 | 2.0786038 | 2.41E-33 | 1.19E-30 |
| ENSECAG00000001900 | 2.07839813 | 5.15E-13 | 1.34E-11 |
| ENSECAG00000053682 | 2.07666728 | 0.00089792 | 0.0028767 |
| ENSECAG00000012185 | 2.07399202 | 0.00034683 | 0.00125024 |
| ENSECAG00000007210 | 2.07204814 | 6.87E-06 | 3.86E-05 |
| ENSECAG00000053300 | 2.06996567 | 3.12E-11 | 5.95E-10 |
| ENSECAG00000057979 | 2.0697513 | 0.00012556 | 0.00051239 |
| ENSECAG00000030224 | 2.06958281 | 4.93E-05 | 0.00022493 |
| ENSECAG00000009011 | 2.06872451 | 1.03E-10 | 1.79E-09 |
| ENSECAG00000024715 | 2.06657655 | 2.58E-06 | 1.63E-05 |
| ENSECAG00000021211 | 2.06547285 | 9.00E-14 | 2.66E-12 |
| ENSECAG00000052959 | 2.06457076 | 0.00458346 | 0.01194984 |
| ENSECAG00000056165 | 2.06359644 | 2.44E-09 | 3.22E-08 |
| ENSECAG00000005475 | 2.06306595 | 0.00925639 | 0.02186272 |
| ENSECAG00000024683 | 2.06116267 | 4.46E-22 | 4.79E-20 |
| ENSECAG00000034040 | 2.06024598 | 0.003218 | 0.00877158 |
| ENSECAG00000010553 | 2.05934796 | 2.53E-05 | 0.00012386 |
| ENSECAG00000048317 | 2.05687216 | 0.01118216 | 0.02571806 |
| ENSECAG00000046289 | 2.05625014 | 5.02E-09 | 6.15E-08 |
| ENSECAG00000059212 | 2.05527876 | 0.01149922 | 0.02634355 |
| ENSECAG00000023839 | 2.05122529 | 3.99E-07 | 3.06E-06 |
| ENSECAG00000058403 | 2.05099883 | 9.43E-09 | 1.08E-07 |
| ENSECAG00000024757 | 2.04943011 | 3.04E-05 | 0.0001455 |
| ENSECAG00000035101 | 2.04923846 | 2.44E-07 | 1.97E-06 |
| ENSECAG00000012650 | 2.04840687 | 0.00066045 | 0.00219197 |
| ENSECAG00000045798 | 2.0469899 | 7.99E-07 | 5.68E-06 |
| ENSECAG00000002984 | 2.04551054 | 8.13E-05 | 0.00035085 |
| ENSECAG00000000106 | 2.04496478 | 4.97E-38 | 5.05E-35 |
| ENSECAG00000009256 | 2.04448695 | 1.32E-05 | 6.92E-05 |
| ENSECAG00000018760 | 2.04388342 | 2.00E-26 | 4.10E-24 |
| ENSECAG00000010617 | 2.0436769 | 4.93E-19 | 3.54E-17 |
| ENSECAG00000011271 | 2.04130013 | 1.55E-06 | 1.03E-05 |
| ENSECAG00000009218 | 2.03529812 | 0.00647491 | 0.0161069 |
| ENSECAG00000000593 | 2.03314789 | 0.00575577 | 0.01454465 |
| ENSECAG00000050529 | 2.03272428 | 1.03E-23 | 1.43E-21 |
| ENSECAG00000020341 | 2.03268176 | 0.01253537 | 0.02829735 |
| ENSECAG00000020148 | 2.03260353 | 2.60E-31 | 1.08E-28 |
| ENSECAG00000034307 | 2.0313099 | 1.05E-05 | 5.65E-05 |
| ENSECAG00000050367 | 2.03020052 | 0.00963506 | 0.02264288 |
| ENSECAG00000014945 | 2.02942861 | 0.00049513 | 0.00169929 |
| ENSECAG00000048094 | 2.02867779 | 0.00110576 | 0.00344747 |
| ENSECAG00000023325 | 2.02471564 | 9.58E-15 | 3.37E-13 |
| ENSECAG00000022345 | 2.02208978 | 1.35E-23 | 1.81E-21 |
| ENSECAG00000020122 | 2.02179655 | 0.00036809 | 0.00131353 |
| ENSECAG00000015898 | 2.02051138 | 7.92E-19 | 5.45E-17 |
| ENSECAG00000056876 | 2.01993364 | 1.75E-05 | 8.90E-05 |
| ENSECAG00000000982 | 2.01901941 | 1.70E-11 | 3.41E-10 |
| ENSECAG00000057145 | 2.01796284 | 0.00808242 | 0.0195085 |
| ENSECAG00000009515 | 2.01667064 | 3.16E-11 | 6.03E-10 |
| ENSECAG00000017267 | 2.01659714 | 0.00016876 | 0.00066405 |
| ENSECAG00000051551 | 2.01463822 | 0.000899 | 0.00287972 |
| ENSECAG00000009542 | 2.01304811 | 7.96E-05 | 0.00034473 |
| ENSECAG00000011948 | 2.01276171 | 6.57E-05 | 0.00029044 |
| ENSECAG00000000932 | 2.0118766 | 0.00843565 | 0.02023153 |
| ENSECAG00000020003 | 2.01167279 | 1.64E-05 | 8.37E-05 |
| ENSECAG00000055292 | 2.01154642 | 0.01783532 | 0.03837358 |
| ENSECAG00000023330 | 2.01079711 | 2.49E-07 | 2.01E-06 |
| ENSECAG00000033164 | 2.01026792 | 0.00046243 | 0.00160456 |
| ENSECAG00000058771 | 2.00751984 | 0.00091632 | 0.00292724 |
| ENSECAG00000059877 | 2.00719806 | 0.00091413 | 0.00292149 |
| ENSECAG00000006409 | 2.00595859 | 6.02E-44 | 1.12E-40 |
| ENSECAG00000058331 | 2.00582949 | 2.69E-06 | 1.68E-05 |
| ENSECAG00000005713 | 2.0052972 | 0.00251568 | 0.00709074 |
| ENSECAG00000017607 | 2.00517628 | 0.00018649 | 0.00072361 |
| ENSECAG00000012496 | 2.00503016 | 1.44E-24 | 2.29E-22 |
| ENSECAG00000039151 | 2.00161003 | 0.00362271 | 0.00974165 |
| ENSECAG00000001454 | 2.00115119 | 2.27E-06 | 1.45E-05 |
| ENSECAG00000055668 | 2.00097232 | 0.00154137 | 0.00461771 |
| ENSECAG00000012334 | 2.00041754 | 1.48E-14 | 4.98E-13 |
| ENSECAG00000000775 | 2.00015638 | 3.99E-10 | 6.12E-09 |
| ENSECAG00000014638 | 1.99749809 | 3.28E-05 | 0.00015613 |
| ENSECAG00000012423 | 1.997396 | 7.37E-05 | 0.0003221 |
| ENSECAG00000010512 | 1.99724671 | 6.98E-17 | 3.44E-15 |
| ENSECAG00000032213 | 1.99719613 | 1.27E-06 | 8.63E-06 |
| ENSECAG00000017037 | 1.99717389 | 3.22E-05 | 0.00015331 |
| ENSECAG00000022842 | 1.99477042 | 2.12E-13 | 5.89E-12 |
| ENSECAG00000019691 | 1.99219591 | 8.66E-12 | 1.81E-10 |
| ENSECAG00000054598 | 1.99099426 | 2.96E-11 | 5.67E-10 |
| ENSECAG00000044148 | 1.99051758 | 2.02E-09 | 2.72E-08 |
| ENSECAG00000025041 | 1.98955219 | 1.15E-07 | 1.01E-06 |
| ENSECAG00000024997 | 1.98929747 | 3.17E-14 | 1.02E-12 |
| ENSECAG00000018717 | 1.98771763 | 9.90E-09 | 1.12E-07 |
| ENSECAG00000010619 | 1.98743469 | 0.00031286 | 0.00114259 |
| ENSECAG00000015766 | 1.98655641 | 0.00113251 | 0.00351829 |
| ENSECAG00000055523 | 1.9847285 | 1.66E-30 | 5.88E-28 |
| ENSECAG00000019324 | 1.98250938 | 0.00346762 | 0.00936856 |
| ENSECAG00000037752 | 1.98075829 | 0.01725113 | 0.03726377 |
| ENSECAG00000055025 | 1.98001036 | 0.01654686 | 0.03595432 |
| ENSECAG00000016586 | 1.97930473 | 2.85E-24 | 4.36E-22 |
| ENSECAG00000017606 | 1.97845109 | 2.60E-29 | 7.55E-27 |
| ENSECAG00000058146 | 1.97730892 | 0.00474849 | 0.01232831 |
| ENSECAG00000056575 | 1.97722347 | 0.00087693 | 0.00281875 |
| ENSECAG00000035050 | 1.97709538 | 0.00396782 | 0.01054906 |
| ENSECAG00000016940 | 1.97534112 | 1.24E-20 | 1.08E-18 |
| ENSECAG00000057238 | 1.97517561 | 0.0241653 | 0.04965954 |
| ENSECAG00000019138 | 1.97494003 | 0.01579656 | 0.03459667 |
| ENSECAG00000021689 | 1.97379836 | 9.11E-06 | 4.97E-05 |
| ENSECAG00000036967 | 1.97378275 | 0.02057416 | 0.04335631 |
| ENSECAG00000049195 | 1.97090957 | 0.00085059 | 0.00274315 |
| ENSECAG00000013415 | 1.96971693 | 6.65E-05 | 0.00029325 |
| ENSECAG00000011294 | 1.96867149 | 0.0023737 | 0.00674758 |
| ENSECAG00000008727 | 1.96784481 | 0.00085776 | 0.0027631 |
| ENSECAG00000047726 | 1.9667902 | 0.00267814 | 0.0074854 |
| ENSECAG00000016408 | 1.96616077 | 0.00233602 | 0.00665685 |
| ENSECAG00000014338 | 1.9659856 | 0.00233613 | 0.00665685 |
| ENSECAG00000011731 | 1.96579599 | 0.02271908 | 0.0471384 |
| ENSECAG00000006306 | 1.96469137 | 6.76E-11 | 1.21E-09 |
| ENSECAG00000015487 | 1.96436534 | 1.72E-07 | 1.45E-06 |
| ENSECAG00000038446 | 1.96249497 | 5.79E-11 | 1.05E-09 |
| ENSECAG00000014056 | 1.9619172 | 0.00018818 | 0.00072892 |
| ENSECAG00000049482 | 1.96182136 | 1.34E-07 | 1.16E-06 |
| ENSECAG00000046030 | 1.96158374 | 0.00053681 | 0.00182692 |
| ENSECAG00000016349 | 1.9603739 | 6.88E-05 | 0.00030255 |
| ENSECAG00000017042 | 1.9572042 | 2.55E-10 | 4.09E-09 |
| ENSECAG00000006665 | 1.95354454 | 4.72E-09 | 5.80E-08 |
| ENSECAG00000014361 | 1.95310629 | 5.58E-07 | 4.14E-06 |
| ENSECAG00000052587 | 1.95241639 | 0.00052262 | 0.00178515 |
| ENSECAG00000021083 | 1.95124626 | 0.00172115 | 0.00508875 |
| ENSECAG00000057223 | 1.95028439 | 0.00038415 | 0.00136302 |
| ENSECAG00000044386 | 1.94896835 | 6.34E-05 | 0.00028177 |
| ENSECAG00000017682 | 1.94826416 | 1.70E-09 | 2.32E-08 |
| ENSECAG00000029368 | 1.9478466 | 3.05E-17 | 1.62E-15 |
| ENSECAG00000055629 | 1.94667795 | 1.34E-05 | 6.99E-05 |
| ENSECAG00000053048 | 1.94497855 | 4.17E-08 | 4.04E-07 |
| ENSECAG00000025065 | 1.94425425 | 3.58E-17 | 1.85E-15 |
| ENSECAG00000054180 | 1.93893305 | 0.00433306 | 0.01137397 |
| ENSECAG00000019854 | 1.93587445 | 4.33E-10 | 6.58E-09 |
| ENSECAG00000054981 | 1.9354987 | 0.01326296 | 0.02974747 |
| ENSECAG00000048924 | 1.93420656 | 0.013268 | 0.0297528 |
| ENSECAG00000050900 | 1.93371144 | 3.54E-07 | 2.75E-06 |
| ENSECAG00000016110 | 1.93332671 | 1.53E-27 | 3.63E-25 |
| ENSECAG00000057815 | 1.93145808 | 0.01513788 | 0.03333376 |
| ENSECAG00000058518 | 1.93124707 | 0.00146002 | 0.00440767 |
| ENSECAG00000013457 | 1.93004936 | 0.01508033 | 0.03321455 |
| ENSECAG00000005887 | 1.92853147 | 2.73E-06 | 1.70E-05 |
| ENSECAG00000035746 | 1.92750003 | 0.00019313 | 0.00074654 |
| ENSECAG00000039959 | 1.92681011 | 6.54E-08 | 6.05E-07 |
| ENSECAG00000046205 | 1.92630423 | 0.00033489 | 0.00121286 |
| ENSECAG00000008004 | 1.92613597 | 2.82E-09 | 3.67E-08 |
| ENSECAG00000053975 | 1.92568969 | 0.00287896 | 0.00795809 |
| ENSECAG00000007023 | 1.92490073 | 2.38E-21 | 2.28E-19 |
| ENSECAG00000016274 | 1.9226157 | 1.20E-16 | 5.69E-15 |
| ENSECAG00000010702 | 1.92233215 | 0.0001436 | 0.00057673 |
| ENSECAG00000038241 | 1.92182017 | 8.98E-05 | 0.00038323 |
| ENSECAG00000021627 | 1.92125151 | 6.69E-14 | 2.04E-12 |
| ENSECAG00000051649 | 1.92046186 | 0.00133185 | 0.00406746 |
| ENSECAG00000053519 | 1.91989094 | 0.00030484 | 0.00111644 |
| ENSECAG00000016328 | 1.91971017 | 6.80E-14 | 2.07E-12 |
| ENSECAG00000024328 | 1.91561137 | 6.75E-18 | 3.98E-16 |
| ENSECAG00000048481 | 1.91516295 | 0.00720147 | 0.0176413 |
| ENSECAG00000048221 | 1.91362024 | 1.13E-05 | 6.00E-05 |
| ENSECAG00000058207 | 1.9132755 | 5.44E-05 | 0.00024533 |
| ENSECAG00000007681 | 1.91314119 | 9.70E-06 | 5.26E-05 |
| ENSECAG00000048008 | 1.91304696 | 6.67E-09 | 7.92E-08 |
| ENSECAG00000055839 | 1.91190545 | 0.0001667 | 0.00065675 |
| ENSECAG00000010281 | 1.9110946 | 3.74E-05 | 0.00017518 |
| ENSECAG00000023397 | 1.91049621 | 2.06E-18 | 1.30E-16 |
| ENSECAG00000059747 | 1.91034921 | 0.01208152 | 0.02743877 |
| ENSECAG00000057613 | 1.91023008 | 0.00039036 | 0.00138284 |
| ENSECAG00000015640 | 1.91019412 | 0.00018792 | 0.00072806 |
| ENSECAG00000021048 | 1.9100566 | 1.08E-22 | 1.24E-20 |
| ENSECAG00000008830 | 1.90866964 | 8.33E-08 | 7.52E-07 |
| ENSECAG00000052666 | 1.90844915 | 0.00077991 | 0.00253976 |
| ENSECAG00000024875 | 1.90548831 | 0.0037348 | 0.01000333 |
| ENSECAG00000023315 | 1.90545268 | 1.66E-14 | 5.57E-13 |
| ENSECAG00000015234 | 1.90540392 | 0.00027688 | 0.00102624 |
| ENSECAG00000016620 | 1.90536419 | 0.00205403 | 0.00594858 |
| ENSECAG00000028786 | 1.90392213 | 0.0006045 | 0.00203008 |
| ENSECAG00000053031 | 1.90342618 | 0.01531182 | 0.03367364 |
| ENSECAG00000003173 | 1.8991901 | 6.50E-08 | 6.02E-07 |
| ENSECAG00000005100 | 1.89728622 | 8.14E-22 | 8.62E-20 |
| ENSECAG00000032952 | 1.89582713 | 6.67E-07 | 4.84E-06 |
| ENSECAG00000016928 | 1.89505588 | 8.08E-05 | 0.00034909 |
| ENSECAG00000036563 | 1.89491688 | 4.35E-05 | 0.00020115 |
| ENSECAG00000014034 | 1.89388637 | 0.00157863 | 0.00471354 |
| ENSECAG00000055343 | 1.89300513 | 6.84E-06 | 3.84E-05 |
| ENSECAG00000049865 | 1.89032665 | 1.24E-05 | 6.55E-05 |
| ENSECAG00000009740 | 1.88882469 | 0.00041223 | 0.00145065 |
| ENSECAG00000057721 | 1.88675894 | 4.33E-07 | 3.29E-06 |
| ENSECAG00000045151 | 1.88627945 | 0.00264061 | 0.00739809 |
| ENSECAG00000011246 | 1.88463761 | 0.00482829 | 0.01251081 |
| ENSECAG00000022446 | 1.88344149 | 2.29E-06 | 1.46E-05 |
| ENSECAG00000052100 | 1.88309766 | 3.09E-05 | 0.00014767 |
| ENSECAG00000018802 | 1.88286826 | 8.33E-07 | 5.89E-06 |
| ENSECAG00000052935 | 1.88123495 | 0.00115507 | 0.0035819 |
| ENSECAG00000057296 | 1.88066434 | 0.00795613 | 0.01923485 |
| ENSECAG00000021314 | 1.88035367 | 7.21E-14 | 2.18E-12 |
| ENSECAG00000003159 | 1.87801464 | 0.00052287 | 0.00178575 |
| ENSECAG00000013102 | 1.87657744 | 0.00291206 | 0.00804163 |
| ENSECAG00000033105 | 1.87506411 | 0.00486436 | 0.01259547 |
| ENSECAG00000021600 | 1.87203147 | 1.02E-06 | 7.12E-06 |
| ENSECAG00000043494 | 1.87075661 | 3.62E-07 | 2.81E-06 |
| ENSECAG00000001688 | 1.87036533 | 1.97E-05 | 9.89E-05 |
| ENSECAG00000021552 | 1.86798437 | 0.0005628 | 0.00190463 |
| ENSECAG00000021849 | 1.86777892 | 1.04E-21 | 1.08E-19 |
| ENSECAG00000053448 | 1.86725496 | 7.01E-07 | 5.06E-06 |
| ENSECAG00000059849 | 1.86317591 | 5.56E-07 | 4.13E-06 |
| ENSECAG00000050706 | 1.86248781 | 0.00014882 | 0.00059524 |
| ENSECAG00000009387 | 1.86093515 | 9.39E-12 | 1.96E-10 |
| ENSECAG00000021776 | 1.86070681 | 0.00516189 | 0.01325378 |
| ENSECAG00000013547 | 1.8593906 | 7.36E-11 | 1.31E-09 |
| ENSECAG00000054751 | 1.85891959 | 0.00169301 | 0.00501749 |
| ENSECAG00000015483 | 1.85815896 | 0.01273803 | 0.02869975 |
| ENSECAG00000018963 | 1.85439834 | 2.12E-12 | 4.95E-11 |
| ENSECAG00000018067 | 1.85293235 | 1.51E-08 | 1.64E-07 |
| ENSECAG00000030728 | 1.85255682 | 0.00065468 | 0.00217441 |
| ENSECAG00000020045 | 1.85254494 | 5.23E-09 | 6.35E-08 |
| ENSECAG00000029716 | 1.850768 | 3.15E-08 | 3.16E-07 |
| ENSECAG00000019014 | 1.84952993 | 9.22E-24 | 1.30E-21 |
| ENSECAG00000008848 | 1.84810854 | 4.46E-40 | 5.54E-37 |
| ENSECAG00000010716 | 1.84673553 | 0.00141191 | 0.00427977 |
| ENSECAG00000051123 | 1.84670856 | 0.00018068 | 0.00070451 |
| ENSECAG00000020155 | 1.84614868 | 2.47E-07 | 1.99E-06 |
| ENSECAG00000023071 | 1.84477429 | 3.09E-05 | 0.00014804 |
| ENSECAG00000055322 | 1.84459094 | 0.00281875 | 0.00781197 |
| ENSECAG00000017357 | 1.84346133 | 0.00064037 | 0.00213388 |
| ENSECAG00000050019 | 1.84265148 | 6.64E-05 | 0.00029284 |
| ENSECAG00000006722 | 1.84053971 | 0.00010459 | 0.00043884 |
| ENSECAG00000055109 | 1.84031468 | 0.01251233 | 0.02825675 |
| ENSECAG00000051512 | 1.83950126 | 0.00240944 | 0.00683006 |
| ENSECAG00000037942 | 1.83406726 | 2.42E-08 | 2.50E-07 |
| ENSECAG00000029616 | 1.832912 | 0.00136485 | 0.00415686 |
| ENSECAG00000033857 | 1.83237788 | 4.07E-08 | 3.97E-07 |
| ENSECAG00000045189 | 1.8322284 | 0.01416864 | 0.03147554 |
| ENSECAG00000015807 | 1.83094081 | 5.54E-05 | 0.00024938 |
| ENSECAG00000022615 | 1.82906241 | 0.02131587 | 0.04466239 |
| ENSECAG00000051728 | 1.82689885 | 5.67E-07 | 4.19E-06 |
| ENSECAG00000033602 | 1.82486646 | 2.66E-07 | 2.13E-06 |
| ENSECAG00000004727 | 1.82452947 | 1.25E-22 | 1.41E-20 |
| ENSECAG00000056825 | 1.82287787 | 0.00537641 | 0.01373043 |
| ENSECAG00000009990 | 1.82275848 | 8.78E-15 | 3.11E-13 |
| ENSECAG00000009864 | 1.8224347 | 3.62E-05 | 0.00017015 |
| ENSECAG00000050690 | 1.82154338 | 2.40E-06 | 1.52E-05 |
| ENSECAG00000013376 | 1.81756916 | 0.01158798 | 0.02652399 |
| ENSECAG00000021025 | 1.81483524 | 0.00042248 | 0.00148369 |
| ENSECAG00000049789 | 1.81455506 | 0.02298393 | 0.04759722 |
| ENSECAG00000008923 | 1.81440431 | 1.87E-07 | 1.56E-06 |
| ENSECAG00000008164 | 1.81416995 | 2.45E-35 | 1.61E-32 |
| ENSECAG00000017436 | 1.80951992 | 0.02125714 | 0.04455606 |
| ENSECAG00000048330 | 1.8082437 | 0.0044145 | 0.01155652 |
| ENSECAG00000008814 | 1.80788862 | 0.00148704 | 0.00447532 |
| ENSECAG00000017718 | 1.80768225 | 2.15E-12 | 4.99E-11 |
| ENSECAG00000003758 | 1.80705387 | 1.58E-25 | 2.96E-23 |
| ENSECAG00000018095 | 1.8069965 | 6.98E-19 | 4.86E-17 |
| ENSECAG00000057942 | 1.80686468 | 0.00030199 | 0.00110737 |
| ENSECAG00000054706 | 1.80629065 | 8.83E-06 | 4.84E-05 |
| ENSECAG00000013425 | 1.80574879 | 7.56E-05 | 0.00032931 |
| ENSECAG00000021206 | 1.79872596 | 0.00030858 | 0.00112929 |
| ENSECAG00000014973 | 1.79788874 | 2.51E-07 | 2.02E-06 |
| ENSECAG00000032416 | 1.79689749 | 0.0001136 | 0.00047107 |
| ENSECAG00000031941 | 1.79329599 | 1.25E-07 | 1.08E-06 |
| ENSECAG00000022641 | 1.79314057 | 1.92E-16 | 8.82E-15 |
| ENSECAG00000017227 | 1.79307262 | 2.30E-14 | 7.52E-13 |
| ENSECAG00000019158 | 1.79290967 | 0.00032805 | 0.00119081 |
| ENSECAG00000024790 | 1.79277006 | 6.32E-08 | 5.87E-07 |
| ENSECAG00000008193 | 1.79109638 | 0.00063076 | 0.00210718 |
| ENSECAG00000019111 | 1.78989012 | 7.83E-08 | 7.12E-07 |
| ENSECAG00000015566 | 1.78870873 | 1.58E-05 | 8.13E-05 |
| ENSECAG00000010484 | 1.7857704 | 0.01365863 | 0.03050023 |
| ENSECAG00000020142 | 1.78487459 | 0.00280221 | 0.00777093 |
| ENSECAG00000053755 | 1.7827686 | 0.00368418 | 0.00988671 |
| ENSECAG00000019266 | 1.7823892 | 5.53E-07 | 4.11E-06 |
| ENSECAG00000015029 | 1.78230303 | 1.64E-08 | 1.77E-07 |
| ENSECAG00000007173 | 1.78124291 | 2.75E-13 | 7.52E-12 |
| ENSECAG00000024322 | 1.7790783 | 1.99E-07 | 1.64E-06 |
| ENSECAG00000010840 | 1.7779675 | 1.80E-11 | 3.59E-10 |
| ENSECAG00000057868 | 1.77747636 | 0.00036454 | 0.00130421 |
| ENSECAG00000033327 | 1.77738869 | 7.49E-06 | 4.17E-05 |
| ENSECAG00000022899 | 1.77595125 | 3.35E-08 | 3.34E-07 |
| ENSECAG00000046517 | 1.77541992 | 1.81E-10 | 2.99E-09 |
| ENSECAG00000021859 | 1.77493095 | 7.31E-06 | 4.08E-05 |
| ENSECAG00000023348 | 1.77270021 | 1.17E-07 | 1.02E-06 |
| ENSECAG00000009580 | 1.77264145 | 0.00039422 | 0.00139474 |
| ENSECAG00000023484 | 1.77119542 | 0.00459259 | 0.01197224 |
| ENSECAG00000010106 | 1.77101763 | 1.00E-13 | 2.92E-12 |
| ENSECAG00000012405 | 1.77040445 | 0.01077288 | 0.02490747 |
| ENSECAG00000007143 | 1.76655068 | 0.02166443 | 0.04526539 |
| ENSECAG00000010085 | 1.76569156 | 1.73E-45 | 5.54E-42 |
| ENSECAG00000021387 | 1.76532126 | 0.01052841 | 0.0244358 |
| ENSECAG00000044785 | 1.76529885 | 0.02362176 | 0.04867685 |
| ENSECAG00000013081 | 1.7651385 | 0.01766627 | 0.03806484 |
| ENSECAG00000006500 | 1.76477428 | 9.19E-05 | 0.00039112 |
| ENSECAG00000016060 | 1.76385356 | 1.60E-19 | 1.24E-17 |
| ENSECAG00000046173 | 1.76244733 | 0.02333651 | 0.04820018 |
| ENSECAG00000055429 | 1.76191436 | 0.00012456 | 0.00050906 |
| ENSECAG00000002797 | 1.76155384 | 0.00650765 | 0.01617214 |
| ENSECAG00000030746 | 1.76108148 | 0.00875132 | 0.02086164 |
| ENSECAG00000015644 | 1.75951246 | 0.00020566 | 0.00078992 |
| ENSECAG00000008274 | 1.75950431 | 0.00571423 | 0.01445277 |
| ENSECAG00000028085 | 1.75911184 | 0.00544249 | 0.01387676 |
| ENSECAG00000012876 | 1.75816816 | 2.19E-15 | 8.53E-14 |
| ENSECAG00000047540 | 1.75784683 | 0.01454967 | 0.03220673 |
| ENSECAG00000017164 | 1.75658397 | 2.43E-48 | 1.08E-44 |
| ENSECAG00000038727 | 1.75642732 | 0.02387505 | 0.04914074 |
| ENSECAG00000056693 | 1.75605607 | 7.70E-05 | 0.00033476 |
| ENSECAG00000024710 | 1.75576583 | 3.11E-36 | 2.24E-33 |
| ENSECAG00000046740 | 1.75542897 | 1.71E-13 | 4.82E-12 |
| ENSECAG00000050577 | 1.75517711 | 0.00048696 | 0.00167641 |
| ENSECAG00000043506 | 1.75332498 | 6.23E-05 | 0.00027758 |
| ENSECAG00000046676 | 1.75223545 | 0.00043379 | 0.00151672 |
| ENSECAG00000016984 | 1.75180278 | 2.01E-07 | 1.66E-06 |
| ENSECAG00000003789 | 1.7508869 | 1.36E-07 | 1.17E-06 |
| ENSECAG00000021675 | 1.74959131 | 3.48E-17 | 1.82E-15 |
| ENSECAG00000016560 | 1.74912006 | 9.85E-12 | 2.04E-10 |
| ENSECAG00000010405 | 1.7490448 | 1.23E-12 | 3.00E-11 |
| ENSECAG00000056156 | 1.74895471 | 0.020017 | 0.04234992 |
| ENSECAG00000049322 | 1.74797109 | 6.74E-05 | 0.00029685 |
| ENSECAG00000007145 | 1.74749556 | 7.09E-13 | 1.80E-11 |
| ENSECAG00000018066 | 1.74686403 | 0.01167493 | 0.02669477 |
| ENSECAG00000022129 | 1.74502634 | 4.25E-06 | 2.52E-05 |
| ENSECAG00000022448 | 1.74413317 | 3.13E-09 | 4.01E-08 |
| ENSECAG00000056923 | 1.74399943 | 0.02293114 | 0.04751221 |
| ENSECAG00000005590 | 1.74085382 | 2.98E-19 | 2.22E-17 |
| ENSECAG00000015276 | 1.73927554 | 1.49E-12 | 3.58E-11 |
| ENSECAG00000046563 | 1.7370366 | 0.01325332 | 0.02973183 |
| ENSECAG00000045394 | 1.73626517 | 1.12E-09 | 1.57E-08 |
| ENSECAG00000021890 | 1.73519427 | 2.07E-10 | 3.37E-09 |
| ENSECAG00000006229 | 1.73478388 | 0.0025557 | 0.00719173 |
| ENSECAG00000038947 | 1.73473519 | 2.58E-05 | 0.00012578 |
| ENSECAG00000035229 | 1.73390995 | 2.12E-21 | 2.08E-19 |
| ENSECAG00000056835 | 1.73206772 | 8.06E-07 | 5.72E-06 |
| ENSECAG00000008409 | 1.730944 | 6.40E-26 | 1.27E-23 |
| ENSECAG00000050319 | 1.73002166 | 5.70E-09 | 6.89E-08 |
| ENSECAG00000002707 | 1.72870076 | 0.00155957 | 0.00466161 |
| ENSECAG00000048737 | 1.72833179 | 0.00746345 | 0.01818691 |
| ENSECAG00000014599 | 1.72638981 | 0.01211323 | 0.02749718 |
| ENSECAG00000045543 | 1.72564531 | 0.00309974 | 0.00847509 |
| ENSECAG00000039579 | 1.72547136 | 0.00180714 | 0.00531277 |
| ENSECAG00000002597 | 1.72426106 | 5.16E-09 | 6.30E-08 |
| ENSECAG00000017746 | 1.72348341 | 5.63E-20 | 4.66E-18 |
| ENSECAG00000035853 | 1.72320977 | 1.18E-05 | 6.25E-05 |
| ENSECAG00000050008 | 1.72299753 | 1.02E-05 | 5.50E-05 |
| ENSECAG00000003981 | 1.72295266 | 6.79E-09 | 8.05E-08 |
| ENSECAG00000006249 | 1.72288442 | 6.30E-12 | 1.34E-10 |
| ENSECAG00000036593 | 1.7223215 | 8.23E-08 | 7.44E-07 |
| ENSECAG00000050420 | 1.72161998 | 0.01458772 | 0.03228456 |
| ENSECAG00000004240 | 1.72100825 | 8.89E-16 | 3.67E-14 |
| ENSECAG00000020351 | 1.71915433 | 2.37E-34 | 1.36E-31 |
| ENSECAG00000003105 | 1.71887678 | 0.00303066 | 0.00831879 |
| ENSECAG00000056841 | 1.71833511 | 0.00138211 | 0.00420256 |
| ENSECAG00000014250 | 1.71712687 | 1.96E-06 | 1.27E-05 |
| ENSECAG00000008351 | 1.71586733 | 2.51E-13 | 6.93E-12 |
| ENSECAG00000005682 | 1.71541939 | 0.00068875 | 0.00227541 |
| ENSECAG00000003748 | 1.71177674 | 1.42E-10 | 2.39E-09 |
| ENSECAG00000023531 | 1.71170714 | 7.39E-12 | 1.57E-10 |
| ENSECAG00000012940 | 1.71159582 | 0.01385623 | 0.03089825 |
| ENSECAG00000012768 | 1.71148419 | 2.51E-07 | 2.02E-06 |
| ENSECAG00000015382 | 1.70948066 | 2.29E-07 | 1.86E-06 |
| ENSECAG00000000214 | 1.70831832 | 0.00108055 | 0.00337707 |
| ENSECAG00000028030 | 1.70786106 | 3.19E-13 | 8.60E-12 |
| ENSECAG00000013555 | 1.70739798 | 1.94E-08 | 2.06E-07 |
| ENSECAG00000014561 | 1.70675466 | 1.27E-36 | 1.05E-33 |
| ENSECAG00000031322 | 1.70642464 | 4.53E-07 | 3.44E-06 |
| ENSECAG00000037169 | 1.70515047 | 0.01592867 | 0.03484593 |
| ENSECAG00000000701 | 1.70377751 | 4.80E-05 | 0.00021926 |
| ENSECAG00000024590 | 1.70247099 | 2.66E-16 | 1.19E-14 |
| ENSECAG00000039582 | 1.70170515 | 4.35E-05 | 0.00020109 |
| ENSECAG00000011462 | 1.70105018 | 1.24E-13 | 3.57E-12 |
| ENSECAG00000049297 | 1.70056541 | 5.32E-12 | 1.15E-10 |
| ENSECAG00000014069 | 1.69850647 | 1.30E-17 | 7.36E-16 |
| ENSECAG00000007706 | 1.69686407 | 1.45E-08 | 1.58E-07 |
| ENSECAG00000057139 | 1.69632026 | 0.01682418 | 0.0364782 |
| ENSECAG00000022126 | 1.69590407 | 4.41E-05 | 0.00020342 |
| ENSECAG00000052827 | 1.69422978 | 0.0081762 | 0.01970506 |
| ENSECAG00000000239 | 1.69217052 | 0.00060096 | 0.00201879 |
| ENSECAG00000019934 | 1.69140935 | 8.09E-05 | 0.00034938 |
| ENSECAG00000006846 | 1.6912538 | 0.00146875 | 0.00442924 |
| ENSECAG00000053105 | 1.69073575 | 0.00968506 | 0.02273822 |
| ENSECAG00000011314 | 1.68953843 | 5.36E-08 | 5.06E-07 |
| ENSECAG00000000435 | 1.6891334 | 0.00353118 | 0.00952187 |
| ENSECAG00000044368 | 1.68874212 | 2.73E-08 | 2.78E-07 |
| ENSECAG00000040873 | 1.68571736 | 0.0087026 | 0.02076765 |
| ENSECAG00000011938 | 1.68512591 | 1.45E-30 | 5.21E-28 |
| ENSECAG00000010881 | 1.68445871 | 3.96E-11 | 7.42E-10 |
| ENSECAG00000011897 | 1.68424433 | 0.00632145 | 0.01579019 |
| ENSECAG00000047697 | 1.68397418 | 0.00099605 | 0.00315168 |
| ENSECAG00000037706 | 1.68265574 | 6.10E-06 | 3.48E-05 |
| ENSECAG00000018191 | 1.68233544 | 1.74E-11 | 3.48E-10 |
| ENSECAG00000009179 | 1.68174463 | 0.00739249 | 0.01804199 |
| ENSECAG00000009623 | 1.67957812 | 1.28E-05 | 6.71E-05 |
| ENSECAG00000011600 | 1.67813377 | 0.00980476 | 0.02297821 |
| ENSECAG00000026840 | 1.67565986 | 0.00066251 | 0.00219781 |
| ENSECAG00000022047 | 1.67408331 | 0.00104863 | 0.00329388 |
| ENSECAG00000025161 | 1.67406914 | 4.24E-16 | 1.81E-14 |
| ENSECAG00000011588 | 1.67241535 | 1.80E-06 | 1.18E-05 |
| ENSECAG00000058921 | 1.6715657 | 0.0070537 | 0.01733633 |
| ENSECAG00000010783 | 1.66754845 | 2.66E-05 | 0.00012937 |
| ENSECAG00000054008 | 1.66706816 | 0.00748703 | 0.01822894 |
| ENSECAG00000044783 | 1.66656942 | 1.01E-14 | 3.51E-13 |
| ENSECAG00000017239 | 1.66619957 | 0.00176375 | 0.00519957 |
| ENSECAG00000011067 | 1.66551273 | 5.49E-07 | 4.09E-06 |
| ENSECAG00000010560 | 1.66542578 | 0.01441222 | 0.03195312 |
| ENSECAG00000009637 | 1.66524997 | 3.18E-08 | 3.18E-07 |
| ENSECAG00000009335 | 1.66484979 | 1.23E-23 | 1.66E-21 |
| ENSECAG00000004757 | 1.66472993 | 2.19E-15 | 8.53E-14 |
| ENSECAG00000021370 | 1.6645072 | 1.27E-06 | 8.60E-06 |
| ENSECAG00000010286 | 1.66438057 | 0.00983861 | 0.02304933 |
| ENSECAG00000008564 | 1.66384273 | 3.35E-06 | 2.04E-05 |
| ENSECAG00000034839 | 1.66338351 | 1.05E-13 | 3.04E-12 |
| ENSECAG00000050833 | 1.66248851 | 0.01184355 | 0.02701949 |
| ENSECAG00000060315 | 1.66161975 | 0.00222097 | 0.00637179 |
| ENSECAG00000023596 | 1.661113 | 2.27E-07 | 1.85E-06 |
| ENSECAG00000023104 | 1.6609177 | 8.87E-06 | 4.85E-05 |
| ENSECAG00000015034 | 1.66080794 | 0.00026779 | 0.00099733 |
| ENSECAG00000013870 | 1.65983757 | 2.58E-14 | 8.35E-13 |
| ENSECAG00000010971 | 1.65952234 | 9.28E-05 | 0.00039426 |
| ENSECAG00000057243 | 1.65784527 | 0.00818209 | 0.01971714 |
| ENSECAG00000047858 | 1.6576925 | 0.0089022 | 0.02116678 |
| ENSECAG00000056434 | 1.65716875 | 0.02113819 | 0.04435248 |
| ENSECAG00000011942 | 1.65622288 | 1.22E-16 | 5.78E-15 |
| ENSECAG00000003553 | 1.65412143 | 0.00118787 | 0.00367443 |
| ENSECAG00000024622 | 1.65394369 | 4.06E-24 | 6.04E-22 |
| ENSECAG00000056592 | 1.65227864 | 4.98E-06 | 2.90E-05 |
| ENSECAG00000021815 | 1.65174132 | 7.95E-33 | 3.78E-30 |
| ENSECAG00000000893 | 1.65124618 | 8.05E-13 | 2.02E-11 |
| ENSECAG00000048368 | 1.64910394 | 0.00305401 | 0.0083736 |
| ENSECAG00000057204 | 1.64908942 | 0.00293179 | 0.00808813 |
| ENSECAG00000024810 | 1.64771741 | 8.14E-11 | 1.44E-09 |
| ENSECAG00000000404 | 1.64745206 | 1.08E-21 | 1.12E-19 |
| ENSECAG00000003398 | 1.6469172 | 1.88E-11 | 3.71E-10 |
| ENSECAG00000018778 | 1.64685215 | 0.01453221 | 0.03217446 |
| ENSECAG00000048957 | 1.6461022 | 0.02318092 | 0.04794085 |
| ENSECAG00000029581 | 1.64562567 | 1.34E-18 | 8.75E-17 |
| ENSECAG00000008044 | 1.64432955 | 0.00809793 | 0.01953751 |
| ENSECAG00000026965 | 1.64177714 | 8.10E-09 | 9.40E-08 |
| ENSECAG00000002257 | 1.64170169 | 8.28E-06 | 4.56E-05 |
| ENSECAG00000056419 | 1.64160335 | 0.01090544 | 0.02516709 |
| ENSECAG00000022715 | 1.6411223 | 4.83E-19 | 3.48E-17 |
| ENSECAG00000017122 | 1.63947176 | 1.60E-07 | 1.36E-06 |
| ENSECAG00000039746 | 1.63865787 | 0.02156438 | 0.0450943 |
| ENSECAG00000058663 | 1.63854973 | 7.86E-05 | 0.00034083 |
| ENSECAG00000013979 | 1.63848679 | 1.56E-15 | 6.30E-14 |
| ENSECAG00000033207 | 1.63834404 | 6.12E-19 | 4.30E-17 |
| ENSECAG00000053118 | 1.6383067 | 7.32E-08 | 6.70E-07 |
| ENSECAG00000020320 | 1.63817353 | 2.98E-11 | 5.70E-10 |
| ENSECAG00000035495 | 1.6378985 | 0.0006577 | 0.0021838 |
| ENSECAG00000007527 | 1.63635983 | 2.09E-10 | 3.40E-09 |
| ENSECAG00000035561 | 1.63402455 | 3.53E-05 | 0.00016637 |
| ENSECAG00000014064 | 1.63339999 | 0.00658534 | 0.01633778 |
| ENSECAG00000016241 | 1.6329437 | 4.93E-08 | 4.70E-07 |
| ENSECAG00000041480 | 1.63238183 | 0.00016077 | 0.00063621 |
| ENSECAG00000053350 | 1.63205292 | 0.00037882 | 0.00134707 |
| ENSECAG00000007607 | 1.6318058 | 1.19E-14 | 4.07E-13 |
| ENSECAG00000021013 | 1.63109812 | 0.00238354 | 0.0067678 |
| ENSECAG00000052987 | 1.62959586 | 3.01E-11 | 5.75E-10 |
| ENSECAG00000022408 | 1.62911513 | 0.00111334 | 0.00346645 |
| ENSECAG00000020875 | 1.62767107 | 2.16E-15 | 8.46E-14 |
| ENSECAG00000058458 | 1.62663671 | 1.35E-05 | 7.05E-05 |
| ENSECAG00000055927 | 1.62627827 | 0.00017895 | 0.00069848 |
| ENSECAG00000000011 | 1.62541929 | 7.28E-24 | 1.05E-21 |
| ENSECAG00000036814 | 1.62444234 | 0.00234322 | 0.00667536 |
| ENSECAG00000006860 | 1.62397284 | 0.0047811 | 0.01240576 |
| ENSECAG00000047192 | 1.62392354 | 0.01478053 | 0.03264338 |
| ENSECAG00000046387 | 1.62368066 | 0.00049349 | 0.00169446 |
| ENSECAG00000023720 | 1.6200779 | 3.25E-05 | 0.00015489 |
| ENSECAG00000052056 | 1.61844918 | 0.00181909 | 0.00534578 |
| ENSECAG00000015238 | 1.6182124 | 6.44E-06 | 3.64E-05 |
| ENSECAG00000012955 | 1.61810371 | 1.45E-05 | 7.52E-05 |
| ENSECAG00000003837 | 1.61762712 | 6.85E-05 | 0.00030122 |
| ENSECAG00000010888 | 1.61740041 | 0.00200778 | 0.00583865 |
| ENSECAG00000012199 | 1.61610455 | 0.00017071 | 0.00067018 |
| ENSECAG00000030526 | 1.61543472 | 2.34E-06 | 1.48E-05 |
| ENSECAG00000029054 | 1.61540256 | 0.00457562 | 0.01193077 |
| ENSECAG00000056418 | 1.61529051 | 0.00017456 | 0.00068338 |
| ENSECAG00000043329 | 1.6146759 | 0.00048785 | 0.00167897 |
| ENSECAG00000031395 | 1.61370073 | 2.30E-23 | 2.96E-21 |
| ENSECAG00000053579 | 1.61316055 | 2.77E-05 | 0.00013423 |
| ENSECAG00000006518 | 1.6126522 | 4.13E-17 | 2.11E-15 |
| ENSECAG00000052301 | 1.61164646 | 0.00095884 | 0.00304695 |
| ENSECAG00000009783 | 1.61132704 | 5.01E-07 | 3.77E-06 |
| ENSECAG00000015385 | 1.61121437 | 9.39E-12 | 1.96E-10 |
| ENSECAG00000010409 | 1.61010484 | 7.44E-09 | 8.73E-08 |
| ENSECAG00000023587 | 1.60908274 | 0.00032156 | 0.00117137 |
| ENSECAG00000005400 | 1.6088308 | 0.0023677 | 0.00673395 |
| ENSECAG00000001407 | 1.60785983 | 3.27E-06 | 2.00E-05 |
| ENSECAG00000057972 | 1.60408215 | 2.49E-13 | 6.86E-12 |
| ENSECAG00000008083 | 1.60350976 | 1.72E-11 | 3.43E-10 |
| ENSECAG00000009297 | 1.60292249 | 4.59E-25 | 7.72E-23 |
| ENSECAG00000017707 | 1.60171861 | 2.84E-06 | 1.76E-05 |
| ENSECAG00000048863 | 1.59956546 | 0.00027226 | 0.00101132 |
| ENSECAG00000006273 | 1.59911746 | 1.90E-13 | 5.32E-12 |
| ENSECAG00000009217 | 1.5987753 | 0.01623382 | 0.03539477 |
| ENSECAG00000038389 | 1.59789288 | 0.00727907 | 0.01780019 |
| ENSECAG00000049223 | 1.59784707 | 0.00268448 | 0.00749843 |
| ENSECAG00000022804 | 1.59777832 | 4.72E-09 | 5.80E-08 |
| ENSECAG00000053703 | 1.59607666 | 3.96E-06 | 2.37E-05 |
| ENSECAG00000051636 | 1.59519007 | 0.00499664 | 0.0128783 |
| ENSECAG00000045989 | 1.59397183 | 4.94E-11 | 9.11E-10 |
| ENSECAG00000049375 | 1.59376947 | 5.00E-05 | 0.00022778 |
| ENSECAG00000023766 | 1.59119135 | 6.56E-05 | 0.00029031 |
| ENSECAG00000000384 | 1.5903127 | 1.24E-41 | 1.85E-38 |
| ENSECAG00000054042 | 1.59003417 | 1.24E-08 | 1.38E-07 |
| ENSECAG00000055926 | 1.58976942 | 0.01187101 | 0.02707108 |
| ENSECAG00000059075 | 1.58948874 | 1.92E-09 | 2.60E-08 |
| ENSECAG00000001069 | 1.58861777 | 3.37E-05 | 0.0001599 |
| ENSECAG00000024205 | 1.58809311 | 3.37E-28 | 8.67E-26 |
| ENSECAG00000000323 | 1.5837531 | 0.01145177 | 0.02626356 |
| ENSECAG00000019037 | 1.5837308 | 5.95E-08 | 5.56E-07 |
| ENSECAG00000018005 | 1.58359119 | 2.07E-10 | 3.37E-09 |
| ENSECAG00000006886 | 1.58316756 | 3.32E-20 | 2.80E-18 |
| ENSECAG00000027974 | 1.58308613 | 0.00966963 | 0.02270676 |
| ENSECAG00000000887 | 1.58135958 | 5.95E-07 | 4.37E-06 |
| ENSECAG00000007214 | 1.58103761 | 3.93E-05 | 0.00018369 |
| ENSECAG00000035289 | 1.58094592 | 0.0219243 | 0.04572712 |
| ENSECAG00000059071 | 1.58028222 | 0.01997803 | 0.04227547 |
| ENSECAG00000023788 | 1.57997421 | 2.71E-05 | 0.00013154 |
| ENSECAG00000020399 | 1.57891989 | 0.00726351 | 0.0177699 |
| ENSECAG00000047371 | 1.57869755 | 6.16E-07 | 4.51E-06 |
| ENSECAG00000009787 | 1.5779344 | 6.54E-06 | 3.70E-05 |
| ENSECAG00000004638 | 1.57684419 | 0.0039981 | 0.0106083 |
| ENSECAG00000046395 | 1.57645567 | 0.01636092 | 0.03563364 |
| ENSECAG00000009707 | 1.57491216 | 5.64E-07 | 4.17E-06 |
| ENSECAG00000041793 | 1.57488451 | 6.00E-06 | 3.42E-05 |
| ENSECAG00000004031 | 1.57448413 | 4.67E-07 | 3.54E-06 |
| ENSECAG00000010726 | 1.57408322 | 3.61E-09 | 4.55E-08 |
| ENSECAG00000020280 | 1.57389652 | 1.11E-22 | 1.26E-20 |
| ENSECAG00000017186 | 1.57361284 | 6.10E-05 | 0.00027212 |
| ENSECAG00000015449 | 1.57350257 | 2.69E-05 | 0.00013073 |
| ENSECAG00000026856 | 1.57326688 | 2.87E-06 | 1.78E-05 |
| ENSECAG00000014585 | 1.57127813 | 0.00043036 | 0.00150615 |
| ENSECAG00000045292 | 1.5711909 | 9.35E-09 | 1.07E-07 |
| ENSECAG00000023844 | 1.5685298 | 4.85E-09 | 5.95E-08 |
| ENSECAG00000013989 | 1.56750635 | 0.00553921 | 0.01408183 |
| ENSECAG00000014112 | 1.56745339 | 3.88E-10 | 5.97E-09 |
| ENSECAG00000018126 | 1.56738269 | 6.93E-07 | 5.01E-06 |
| ENSECAG00000007279 | 1.56682687 | 0.00151267 | 0.00454453 |
| ENSECAG00000021174 | 1.56609232 | 1.05E-11 | 2.16E-10 |
| ENSECAG00000001129 | 1.56465917 | 1.74E-05 | 8.87E-05 |
| ENSECAG00000040945 | 1.564249 | 2.07E-09 | 2.78E-08 |
| ENSECAG00000031389 | 1.56354649 | 0.00350215 | 0.009455 |
| ENSECAG00000030798 | 1.56182138 | 7.90E-10 | 1.14E-08 |
| ENSECAG00000012166 | 1.56173557 | 4.42E-15 | 1.65E-13 |
| ENSECAG00000048455 | 1.56162713 | 8.33E-08 | 7.52E-07 |
| ENSECAG00000012907 | 1.56006808 | 3.65E-06 | 2.20E-05 |
| ENSECAG00000000373 | 1.55917085 | 2.32E-06 | 1.47E-05 |
| ENSECAG00000018784 | 1.55898051 | 1.22E-06 | 8.36E-06 |
| ENSECAG00000008434 | 1.55820575 | 1.12E-10 | 1.94E-09 |
| ENSECAG00000055136 | 1.55778737 | 0.02206325 | 0.04598688 |
| ENSECAG00000009660 | 1.55631409 | 5.04E-11 | 9.28E-10 |
| ENSECAG00000020975 | 1.55588085 | 6.47E-21 | 5.76E-19 |
| ENSECAG00000000121 | 1.55517744 | 1.52E-05 | 7.85E-05 |
| ENSECAG00000008795 | 1.55463153 | 0.00273476 | 0.00761793 |
| ENSECAG00000014364 | 1.55434406 | 7.29E-19 | 5.04E-17 |
| ENSECAG00000049095 | 1.55404183 | 8.31E-07 | 5.88E-06 |
| ENSECAG00000013301 | 1.55400413 | 5.59E-08 | 5.25E-07 |
| ENSECAG00000022500 | 1.55372411 | 7.09E-17 | 3.48E-15 |
| ENSECAG00000012961 | 1.55221398 | 2.94E-06 | 1.82E-05 |
| ENSECAG00000049434 | 1.5514446 | 6.40E-05 | 0.00028401 |
| ENSECAG00000015571 | 1.55142841 | 6.23E-06 | 3.53E-05 |
| ENSECAG00000009941 | 1.5503512 | 9.49E-13 | 2.36E-11 |
| ENSECAG00000040253 | 1.5499531 | 0.01718389 | 0.03713848 |
| ENSECAG00000058184 | 1.54797722 | 0.00268501 | 0.00749889 |
| ENSECAG00000051038 | 1.54784714 | 0.01988954 | 0.04210817 |
| ENSECAG00000024152 | 1.54719552 | 0.00227432 | 0.00650647 |
| ENSECAG00000053747 | 1.54676773 | 6.17E-06 | 3.51E-05 |
| ENSECAG00000019423 | 1.5466352 | 3.13E-14 | 1.01E-12 |
| ENSECAG00000022714 | 1.54619959 | 0.0014568 | 0.00439854 |
| ENSECAG00000046907 | 1.54397878 | 0.00380336 | 0.01016747 |
| ENSECAG00000019446 | 1.54275231 | 3.58E-10 | 5.55E-09 |
| ENSECAG00000020896 | 1.54142527 | 0.01400139 | 0.03117217 |
| ENSECAG00000057368 | 1.54104252 | 0.01179813 | 0.02692963 |
| ENSECAG00000036248 | 1.53979464 | 0.00139112 | 0.00422594 |
| ENSECAG00000024436 | 1.5390363 | 4.58E-11 | 8.50E-10 |
| ENSECAG00000019087 | 1.53729667 | 1.56E-11 | 3.15E-10 |
| ENSECAG00000021255 | 1.53674285 | 3.33E-23 | 4.18E-21 |
| ENSECAG00000018642 | 1.5365926 | 1.76E-15 | 6.97E-14 |
| ENSECAG00000054490 | 1.53651148 | 0.00840724 | 0.02017919 |
| ENSECAG00000006153 | 1.53535506 | 7.02E-05 | 0.00030808 |
| ENSECAG00000050970 | 1.53517407 | 0.00017415 | 0.00068238 |
| ENSECAG00000032664 | 1.53440599 | 3.11E-08 | 3.12E-07 |
| ENSECAG00000015143 | 1.53423155 | 0.00976519 | 0.02290469 |
| ENSECAG00000035675 | 1.53382785 | 0.01018288 | 0.02373243 |
| ENSECAG00000026930 | 1.53380468 | 4.61E-34 | 2.51E-31 |
| ENSECAG00000019884 | 1.53361141 | 7.13E-10 | 1.04E-08 |
| ENSECAG00000044905 | 1.53342653 | 6.67E-05 | 0.00029414 |
| ENSECAG00000046941 | 1.53220202 | 3.66E-06 | 2.21E-05 |
| ENSECAG00000040631 | 1.53149111 | 6.84E-13 | 1.75E-11 |
| ENSECAG00000043589 | 1.53118335 | 0.00203842 | 0.00590874 |
| ENSECAG00000000281 | 1.53103954 | 9.77E-05 | 0.00041244 |
| ENSECAG00000037002 | 1.5308883 | 5.84E-08 | 5.46E-07 |
| ENSECAG00000016423 | 1.53040414 | 0.00185495 | 0.00543683 |
| ENSECAG00000002868 | 1.52975887 | 0.00169822 | 0.00503095 |
| ENSECAG00000035454 | 1.52973803 | 0.00285479 | 0.00789909 |
| ENSECAG00000024852 | 1.52967265 | 0.00048569 | 0.00167384 |
| ENSECAG00000055230 | 1.52899727 | 0.00509444 | 0.01310166 |
| ENSECAG00000004837 | 1.52883546 | 0.02010288 | 0.04248737 |
| ENSECAG00000016870 | 1.528611 | 2.42E-10 | 3.89E-09 |
| ENSECAG00000011051 | 1.52750963 | 0.00051358 | 0.00175668 |
| ENSECAG00000059949 | 1.52660292 | 1.30E-08 | 1.43E-07 |
| ENSECAG00000054673 | 1.52654318 | 0.00388566 | 0.01036194 |
| ENSECAG00000040083 | 1.52582634 | 0.00242123 | 0.00685652 |
| ENSECAG00000058069 | 1.5243741 | 6.22E-17 | 3.10E-15 |
| ENSECAG00000008087 | 1.52374109 | 3.58E-05 | 0.00016846 |
| ENSECAG00000024195 | 1.51869411 | 3.05E-36 | 2.24E-33 |
| ENSECAG00000003007 | 1.51758371 | 2.04E-12 | 4.77E-11 |
| ENSECAG00000017501 | 1.51747171 | 3.27E-08 | 3.26E-07 |
| ENSECAG00000043401 | 1.51681597 | 4.78E-07 | 3.60E-06 |
| ENSECAG00000021310 | 1.51661521 | 1.51E-26 | 3.22E-24 |
| ENSECAG00000046959 | 1.51637812 | 1.37E-21 | 1.38E-19 |
| ENSECAG00000054666 | 1.51543958 | 0.00398747 | 0.01058862 |
| ENSECAG00000029071 | 1.51419056 | 0.00083791 | 0.0027089 |
| ENSECAG00000039473 | 1.51381257 | 0.00016487 | 0.00065091 |
| ENSECAG00000015405 | 1.51346268 | 0.00111968 | 0.00348376 |
| ENSECAG00000018261 | 1.51307145 | 0.00018393 | 0.00071566 |
| ENSECAG00000020200 | 1.51241864 | 5.74E-07 | 4.24E-06 |
| ENSECAG00000014771 | 1.51225425 | 1.17E-07 | 1.02E-06 |
| ENSECAG00000020833 | 1.51155889 | 7.71E-07 | 5.50E-06 |
| ENSECAG00000058558 | 1.51137894 | 0.00840214 | 0.0201691 |
| ENSECAG00000017469 | 1.50955749 | 4.50E-15 | 1.68E-13 |
| ENSECAG00000020654 | 1.50880297 | 1.89E-06 | 1.23E-05 |
| ENSECAG00000018309 | 1.50521188 | 1.71E-21 | 1.69E-19 |
| ENSECAG00000007149 | 1.50237745 | 3.56E-12 | 7.90E-11 |
| ENSECAG00000009132 | 1.50233911 | 0.00163835 | 0.00486909 |
| ENSECAG00000054107 | 1.50224141 | 2.43E-06 | 1.54E-05 |
| ENSECAG00000047670 | 1.50077652 | 2.13E-05 | 0.0001064 |
| ENSECAG00000015095 | 1.50038819 | 0.00121159 | 0.00373745 |
| ENSECAG00000017917 | 1.49564966 | 1.32E-05 | 6.92E-05 |
| ENSECAG00000002250 | 1.49509785 | 6.58E-06 | 3.72E-05 |
| ENSECAG00000039559 | 1.49486645 | 0.00407218 | 0.01077142 |
| ENSECAG00000008545 | 1.49486524 | 5.30E-06 | 3.06E-05 |
| ENSECAG00000000178 | 1.49426198 | 9.74E-13 | 2.41E-11 |
| ENSECAG00000008079 | 1.49383151 | 1.85E-07 | 1.55E-06 |
| ENSECAG00000009444 | 1.49341366 | 2.90E-07 | 2.30E-06 |
| ENSECAG00000006115 | 1.49269811 | 0.00095369 | 0.00303319 |
| ENSECAG00000011516 | 1.49194784 | 1.55E-12 | 3.70E-11 |
| ENSECAG00000014841 | 1.49158065 | 1.27E-08 | 1.41E-07 |
| ENSECAG00000044529 | 1.49075339 | 3.04E-07 | 2.40E-06 |
| ENSECAG00000005460 | 1.48994231 | 2.56E-20 | 2.17E-18 |
| ENSECAG00000008507 | 1.48676918 | 4.88E-13 | 1.28E-11 |
| ENSECAG00000056605 | 1.48662282 | 4.12E-08 | 4.00E-07 |
| ENSECAG00000055283 | 1.48499991 | 0.01872808 | 0.04000168 |
| ENSECAG00000023746 | 1.48495637 | 0.02136901 | 0.04474857 |
| ENSECAG00000055544 | 1.4847695 | 0.00110528 | 0.00344663 |
| ENSECAG00000010218 | 1.48424353 | 2.80E-13 | 7.66E-12 |
| ENSECAG00000014022 | 1.48400445 | 5.44E-07 | 4.05E-06 |
| ENSECAG00000051565 | 1.48246403 | 0.01633384 | 0.0355816 |
| ENSECAG00000009261 | 1.48213287 | 2.93E-05 | 0.00014108 |
| ENSECAG00000005559 | 1.48130537 | 0.01166407 | 0.02667813 |
| ENSECAG00000021425 | 1.48051556 | 0.00326122 | 0.00887423 |
| ENSECAG00000013747 | 1.47992612 | 2.18E-06 | 1.39E-05 |
| ENSECAG00000020343 | 1.47977899 | 3.11E-06 | 1.91E-05 |
| ENSECAG00000020847 | 1.47921843 | 2.99E-12 | 6.71E-11 |
| ENSECAG00000009860 | 1.47841358 | 5.95E-09 | 7.15E-08 |
| ENSECAG00000056226 | 1.47677954 | 7.47E-05 | 0.00032597 |
| ENSECAG00000049199 | 1.47610983 | 1.68E-08 | 1.80E-07 |
| ENSECAG00000015060 | 1.47566194 | 0.00094123 | 0.00299887 |
| ENSECAG00000020711 | 1.47523037 | 3.69E-15 | 1.39E-13 |
| ENSECAG00000022335 | 1.4747089 | 7.97E-18 | 4.64E-16 |
| ENSECAG00000002211 | 1.47463708 | 9.30E-05 | 0.00039484 |
| ENSECAG00000055511 | 1.47446626 | 6.16E-10 | 9.15E-09 |
| ENSECAG00000057133 | 1.47164219 | 3.21E-07 | 2.52E-06 |
| ENSECAG00000000480 | 1.47140354 | 1.49E-05 | 7.70E-05 |
| ENSECAG00000047202 | 1.47017448 | 0.02229142 | 0.04640188 |
| ENSECAG00000022438 | 1.47009143 | 7.55E-09 | 8.84E-08 |
| ENSECAG00000045570 | 1.4691837 | 0.00065033 | 0.00216188 |
| ENSECAG00000008527 | 1.46910818 | 2.35E-22 | 2.56E-20 |
| ENSECAG00000012663 | 1.46781723 | 1.15E-06 | 7.91E-06 |
| ENSECAG00000015439 | 1.46721757 | 5.51E-16 | 2.32E-14 |
| ENSECAG00000022780 | 1.46713078 | 0.00125122 | 0.00384486 |
| ENSECAG00000028692 | 1.46653405 | 0.00016296 | 0.00064381 |
| ENSECAG00000019509 | 1.46648089 | 6.55E-13 | 1.68E-11 |
| ENSECAG00000013937 | 1.46612865 | 0.00011091 | 0.00046155 |
| ENSECAG00000020612 | 1.46555091 | 0.00486192 | 0.01259353 |
| ENSECAG00000032911 | 1.46467426 | 2.29E-06 | 1.46E-05 |
| ENSECAG00000015567 | 1.46446585 | 0.00062886 | 0.00210273 |
| ENSECAG00000045964 | 1.46360809 | 4.22E-05 | 0.00019573 |
| ENSECAG00000018902 | 1.463231 | 6.73E-23 | 7.99E-21 |
| ENSECAG00000021393 | 1.46301924 | 5.65E-19 | 4.02E-17 |
| ENSECAG00000013475 | 1.46259828 | 1.79E-39 | 2.00E-36 |
| ENSECAG00000006485 | 1.46209136 | 1.50E-09 | 2.07E-08 |
| ENSECAG00000038714 | 1.4613614 | 4.64E-09 | 5.72E-08 |
| ENSECAG00000016304 | 1.4608839 | 3.82E-19 | 2.78E-17 |
| ENSECAG00000009049 | 1.46006015 | 3.77E-06 | 2.27E-05 |
| ENSECAG00000011101 | 1.45984887 | 5.89E-06 | 3.37E-05 |
| ENSECAG00000024495 | 1.45952847 | 5.57E-17 | 2.80E-15 |
| ENSECAG00000001741 | 1.45855476 | 1.98E-05 | 9.92E-05 |
| ENSECAG00000039352 | 1.45835649 | 3.47E-06 | 2.11E-05 |
| ENSECAG00000015012 | 1.45821936 | 1.60E-05 | 8.21E-05 |
| ENSECAG00000021351 | 1.45805245 | 2.88E-07 | 2.29E-06 |
| ENSECAG00000059765 | 1.45802259 | 0.00011297 | 0.00046895 |
| ENSECAG00000001640 | 1.45590867 | 1.80E-06 | 1.18E-05 |
| ENSECAG00000034255 | 1.45558651 | 6.65E-06 | 3.76E-05 |
| ENSECAG00000051203 | 1.45528021 | 0.00406192 | 0.0107485 |
| ENSECAG00000023154 | 1.45435701 | 0.00013351 | 0.00053998 |
| ENSECAG00000016152 | 1.45416213 | 0.00303419 | 0.0083254 |
| ENSECAG00000057052 | 1.45333546 | 1.36E-06 | 9.16E-06 |
| ENSECAG00000009180 | 1.45230632 | 0.00220085 | 0.00632218 |
| ENSECAG00000007388 | 1.45182387 | 0.00901569 | 0.02139827 |
| ENSECAG00000051997 | 1.45089436 | 0.02176188 | 0.04543078 |
| ENSECAG00000000753 | 1.45087017 | 2.29E-21 | 2.21E-19 |
| ENSECAG00000012179 | 1.45006965 | 0.00013171 | 0.00053406 |
| ENSECAG00000032241 | 1.4494449 | 1.86E-07 | 1.56E-06 |
| ENSECAG00000016147 | 1.44932012 | 3.27E-10 | 5.12E-09 |
| ENSECAG00000021423 | 1.44889438 | 3.95E-16 | 1.70E-14 |
| ENSECAG00000020365 | 1.44888322 | 0.0115001 | 0.02634355 |
| ENSECAG00000046787 | 1.44784684 | 0.00023657 | 0.00089401 |
| ENSECAG00000022299 | 1.4473655 | 0.01214613 | 0.02755787 |
| ENSECAG00000003580 | 1.44672831 | 8.54E-05 | 0.00036651 |
| ENSECAG00000046748 | 1.44627673 | 0.0178728 | 0.03844312 |
| ENSECAG00000051897 | 1.44581882 | 0.00565518 | 0.01433262 |
| ENSECAG00000014780 | 1.44568031 | 2.07E-17 | 1.13E-15 |
| ENSECAG00000019106 | 1.44502731 | 1.09E-09 | 1.54E-08 |
| ENSECAG00000010934 | 1.44359373 | 0.018521 | 0.03962757 |
| ENSECAG00000008623 | 1.44296229 | 1.69E-22 | 1.86E-20 |
| ENSECAG00000006637 | 1.442712 | 1.23E-06 | 8.39E-06 |
| ENSECAG00000007417 | 1.44244523 | 0.00037504 | 0.00133515 |
| ENSECAG00000014855 | 1.44142458 | 4.07E-08 | 3.96E-07 |
| ENSECAG00000017891 | 1.44072043 | 2.73E-07 | 2.18E-06 |
| ENSECAG00000010184 | 1.44061537 | 0.00019239 | 0.00074392 |
| ENSECAG00000045308 | 1.43994986 | 0.01339461 | 0.02998858 |
| ENSECAG00000005659 | 1.43910957 | 7.91E-14 | 2.37E-12 |
| ENSECAG00000055037 | 1.43833204 | 3.16E-19 | 2.33E-17 |
| ENSECAG00000007474 | 1.43802975 | 1.39E-26 | 2.99E-24 |
| ENSECAG00000058467 | 1.4370856 | 2.80E-05 | 0.00013562 |
| ENSECAG00000042811 | 1.43659771 | 1.53E-22 | 1.71E-20 |
| ENSECAG00000030580 | 1.43576341 | 1.59E-07 | 1.35E-06 |
| ENSECAG00000023895 | 1.43459497 | 0.0002376 | 0.00089688 |
| ENSECAG00000028590 | 1.43397668 | 8.85E-05 | 0.00037841 |
| ENSECAG00000004794 | 1.43359566 | 1.12E-29 | 3.43E-27 |
| ENSECAG00000010891 | 1.43302464 | 1.84E-17 | 1.01E-15 |
| ENSECAG00000013188 | 1.43295682 | 0.00033809 | 0.00122307 |
| ENSECAG00000055678 | 1.43270856 | 0.00401954 | 0.01065363 |
| ENSECAG00000022695 | 1.43269426 | 6.98E-10 | 1.02E-08 |
| ENSECAG00000048458 | 1.43245905 | 0.00171852 | 0.00508299 |
| ENSECAG00000001141 | 1.43138558 | 0.00294544 | 0.00812077 |
| ENSECAG00000006755 | 1.43137928 | 8.87E-11 | 1.56E-09 |
| ENSECAG00000033693 | 1.43102089 | 0.0201648 | 0.04260614 |
| ENSECAG00000059340 | 1.43097837 | 1.90E-06 | 1.23E-05 |
| ENSECAG00000014448 | 1.42765092 | 0.00171916 | 0.00508423 |
| ENSECAG00000011592 | 1.42648328 | 9.34E-07 | 6.54E-06 |
| ENSECAG00000058868 | 1.42578857 | 0.01812773 | 0.03892027 |
| ENSECAG00000015510 | 1.42571249 | 4.12E-08 | 4.00E-07 |
| ENSECAG00000033960 | 1.42555168 | 8.75E-07 | 6.17E-06 |
| ENSECAG00000055680 | 1.42541338 | 0.01045194 | 0.02428607 |
| ENSECAG00000029926 | 1.42494064 | 0.00516509 | 0.01325742 |
| ENSECAG00000023268 | 1.42403101 | 4.69E-05 | 0.00021482 |
| ENSECAG00000008259 | 1.42395596 | 7.42E-10 | 1.08E-08 |
| ENSECAG00000021961 | 1.42277168 | 0.00086124 | 0.0027727 |
| ENSECAG00000000141 | 1.42237179 | 0.00295584 | 0.00814842 |
| ENSECAG00000018996 | 1.42181704 | 0.00861936 | 0.02060862 |
| ENSECAG00000057345 | 1.42114119 | 0.02106318 | 0.04423663 |
| ENSECAG00000004296 | 1.42069366 | 0.01136316 | 0.026086 |
| ENSECAG00000003532 | 1.41818897 | 7.68E-10 | 1.11E-08 |
| ENSECAG00000032794 | 1.41796111 | 1.11E-05 | 5.93E-05 |
| ENSECAG00000023201 | 1.41790842 | 4.09E-07 | 3.13E-06 |
| ENSECAG00000004167 | 1.41500066 | 1.33E-15 | 5.37E-14 |
| ENSECAG00000053885 | 1.41300982 | 0.00235048 | 0.00669008 |
| ENSECAG00000047345 | 1.41075959 | 0.01117038 | 0.02569889 |
| ENSECAG00000046556 | 1.41015925 | 7.30E-05 | 0.00031962 |
| ENSECAG00000003260 | 1.40833313 | 7.49E-08 | 6.84E-07 |
| ENSECAG00000009846 | 1.40802839 | 8.53E-14 | 2.53E-12 |
| ENSECAG00000037191 | 1.40642224 | 0.00018497 | 0.00071885 |
| ENSECAG00000009583 | 1.40632142 | 9.48E-34 | 4.81E-31 |
| ENSECAG00000015715 | 1.40572033 | 3.91E-06 | 2.34E-05 |
| ENSECAG00000047821 | 1.40560156 | 0.00483436 | 0.01252361 |
| ENSECAG00000004151 | 1.40557759 | 0.01518034 | 0.03341409 |
| ENSECAG00000052663 | 1.40551407 | 0.00976755 | 0.02290767 |
| ENSECAG00000054007 | 1.40532812 | 7.91E-08 | 7.17E-07 |
| ENSECAG00000019892 | 1.40470016 | 0.01038543 | 0.02415918 |
| ENSECAG00000017419 | 1.40398475 | 6.64E-10 | 9.80E-09 |
| ENSECAG00000021895 | 1.40386847 | 2.06E-12 | 4.80E-11 |
| ENSECAG00000013357 | 1.40352658 | 0.00603925 | 0.01516837 |
| ENSECAG00000016509 | 1.40187639 | 2.31E-12 | 5.31E-11 |
| ENSECAG00000010291 | 1.40081533 | 3.89E-07 | 2.99E-06 |
| ENSECAG00000042563 | 1.39981187 | 0.00746489 | 0.01818691 |
| ENSECAG00000044030 | 1.39679218 | 5.13E-05 | 0.00023268 |
| ENSECAG00000055513 | 1.39673305 | 9.00E-07 | 6.32E-06 |
| ENSECAG00000023774 | 1.39584898 | 7.96E-30 | 2.51E-27 |
| ENSECAG00000007540 | 1.39524401 | 0.00014727 | 0.00058989 |
| ENSECAG00000051409 | 1.39474308 | 1.12E-13 | 3.23E-12 |
| ENSECAG00000023841 | 1.39367514 | 0.00773974 | 0.01876041 |
| ENSECAG00000018528 | 1.39354492 | 2.74E-30 | 9.14E-28 |
| ENSECAG00000022182 | 1.39330369 | 0.00150162 | 0.00451497 |
| ENSECAG00000016902 | 1.39235575 | 0.00242661 | 0.00687001 |
| ENSECAG00000024986 | 1.39229447 | 1.02E-23 | 1.42E-21 |
| ENSECAG00000018916 | 1.39201167 | 1.93E-05 | 9.74E-05 |
| ENSECAG00000053137 | 1.39199924 | 3.12E-05 | 0.00014932 |
| ENSECAG00000019161 | 1.39153892 | 0.00010616 | 0.00044416 |
| ENSECAG00000014480 | 1.39057825 | 0.00011395 | 0.00047222 |
| ENSECAG00000016667 | 1.38974754 | 5.56E-05 | 0.00025007 |
| ENSECAG00000033923 | 1.38924893 | 2.47E-13 | 6.81E-12 |
| ENSECAG00000031558 | 1.3887102 | 2.49E-05 | 0.00012182 |
| ENSECAG00000046794 | 1.38831317 | 0.00081389 | 0.0026385 |
| ENSECAG00000042607 | 1.38771104 | 1.51E-17 | 8.47E-16 |
| ENSECAG00000006496 | 1.38722452 | 2.28E-05 | 0.00011256 |
| ENSECAG00000020178 | 1.38454295 | 6.75E-10 | 9.94E-09 |
| ENSECAG00000019465 | 1.38281836 | 3.42E-06 | 2.08E-05 |
| ENSECAG00000022696 | 1.38241499 | 0.00400712 | 0.010627 |
| ENSECAG00000015456 | 1.38221481 | 8.13E-10 | 1.17E-08 |
| ENSECAG00000055190 | 1.3820738 | 0.00071977 | 0.0023625 |
| ENSECAG00000017101 | 1.38185247 | 1.48E-21 | 1.49E-19 |
| ENSECAG00000049762 | 1.38110203 | 0.0086189 | 0.02060862 |
| ENSECAG00000045599 | 1.38025338 | 0.00072839 | 0.00238904 |
| ENSECAG00000007949 | 1.37943284 | 3.78E-11 | 7.10E-10 |
| ENSECAG00000002313 | 1.37938458 | 4.74E-21 | 4.30E-19 |
| ENSECAG00000049930 | 1.3789205 | 0.00541193 | 0.01380537 |
| ENSECAG00000049829 | 1.3777603 | 0.02004527 | 0.04238566 |
| ENSECAG00000011991 | 1.37576663 | 4.37E-13 | 1.16E-11 |
| ENSECAG00000023722 | 1.37472099 | 4.48E-13 | 1.18E-11 |
| ENSECAG00000019948 | 1.37458292 | 0.00057257 | 0.00193507 |
| ENSECAG00000012838 | 1.37432991 | 9.10E-12 | 1.90E-10 |
| ENSECAG00000024529 | 1.37282368 | 2.42E-06 | 1.53E-05 |
| ENSECAG00000041820 | 1.37280748 | 0.00142672 | 0.00431763 |
| ENSECAG00000020973 | 1.37252139 | 0.00415369 | 0.01095202 |
| ENSECAG00000021440 | 1.37207476 | 3.99E-15 | 1.50E-13 |
| ENSECAG00000015984 | 1.37179789 | 5.65E-11 | 1.03E-09 |
| ENSECAG00000038231 | 1.37123701 | 2.71E-06 | 1.70E-05 |
| ENSECAG00000015788 | 1.36686464 | 0.00824436 | 0.01984366 |
| ENSECAG00000017237 | 1.36685392 | 0.000927 | 0.00295754 |
| ENSECAG00000002607 | 1.36608735 | 3.52E-18 | 2.17E-16 |
| ENSECAG00000023275 | 1.36531789 | 0.00187319 | 0.00548459 |
| ENSECAG00000039279 | 1.36492736 | 0.01551471 | 0.03406955 |
| ENSECAG00000012441 | 1.36218806 | 0.00156854 | 0.00468647 |
| ENSECAG00000004586 | 1.36166268 | 0.00401949 | 0.01065363 |
| ENSECAG00000019354 | 1.36081041 | 2.33E-12 | 5.37E-11 |
| ENSECAG00000023141 | 1.35911216 | 4.03E-09 | 5.04E-08 |
| ENSECAG00000011249 | 1.3587077 | 1.41E-14 | 4.79E-13 |
| ENSECAG00000020535 | 1.35719176 | 3.46E-12 | 7.70E-11 |
| ENSECAG00000031240 | 1.35684186 | 2.40E-11 | 4.65E-10 |
| ENSECAG00000011660 | 1.3559199 | 0.01720664 | 0.03717846 |
| ENSECAG00000047741 | 1.35571024 | 0.01659723 | 0.03604642 |
| ENSECAG00000000438 | 1.35511021 | 2.45E-18 | 1.53E-16 |
| ENSECAG00000017671 | 1.35434035 | 3.88E-16 | 1.68E-14 |
| ENSECAG00000015748 | 1.35408532 | 0.00094886 | 0.00301826 |
| ENSECAG00000020594 | 1.35374612 | 7.33E-08 | 6.70E-07 |
| ENSECAG00000013905 | 1.35356203 | 7.29E-14 | 2.20E-12 |
| ENSECAG00000050973 | 1.35331549 | 0.00200226 | 0.00582509 |
| ENSECAG00000020244 | 1.35180767 | 6.43E-13 | 1.65E-11 |
| ENSECAG00000014896 | 1.35056088 | 5.72E-06 | 3.28E-05 |
| ENSECAG00000016706 | 1.35006712 | 1.43E-05 | 7.43E-05 |
| ENSECAG00000014696 | 1.34939478 | 0.0133982 | 0.0299893 |
| ENSECAG00000021209 | 1.34924226 | 7.76E-13 | 1.95E-11 |
| ENSECAG00000010993 | 1.34850402 | 3.61E-14 | 1.15E-12 |
| ENSECAG00000050230 | 1.34819276 | 3.69E-10 | 5.70E-09 |
| ENSECAG00000049764 | 1.34749057 | 6.23E-11 | 1.12E-09 |
| ENSECAG00000002784 | 1.34694122 | 0.02069831 | 0.04357682 |
| ENSECAG00000034840 | 1.34653187 | 4.05E-08 | 3.95E-07 |
| ENSECAG00000009425 | 1.34562193 | 0.00736249 | 0.01798058 |
| ENSECAG00000021429 | 1.34413706 | 0.00022561 | 0.0008568 |
| ENSECAG00000011292 | 1.34409386 | 0.00281722 | 0.00780868 |
| ENSECAG00000018874 | 1.34176897 | 9.90E-08 | 8.78E-07 |
| ENSECAG00000031458 | 1.34172463 | 2.54E-13 | 6.97E-12 |
| ENSECAG00000010914 | 1.3410421 | 1.45E-08 | 1.58E-07 |
| ENSECAG00000017478 | 1.34100831 | 1.30E-06 | 8.84E-06 |
| ENSECAG00000053298 | 1.34042349 | 0.00488548 | 0.01262969 |
| ENSECAG00000001491 | 1.34016606 | 2.58E-19 | 1.93E-17 |
| ENSECAG00000051463 | 1.33995247 | 2.62E-08 | 2.68E-07 |
| ENSECAG00000036146 | 1.33901087 | 0.00198119 | 0.00576678 |
| ENSECAG00000019495 | 1.33791422 | 0.00916275 | 0.02168051 |
| ENSECAG00000054965 | 1.3378374 | 0.01466866 | 0.03244761 |
| ENSECAG00000020767 | 1.33681346 | 0.0004271 | 0.00149711 |
| ENSECAG00000042287 | 1.33538919 | 0.01671213 | 0.03626777 |
| ENSECAG00000047376 | 1.33523619 | 0.00417722 | 0.01101016 |
| ENSECAG00000012066 | 1.33511331 | 1.37E-08 | 1.50E-07 |
| ENSECAG00000030791 | 1.33511081 | 1.73E-15 | 6.88E-14 |
| ENSECAG00000022611 | 1.33500124 | 0.00632529 | 0.01579802 |
| ENSECAG00000020440 | 1.33460805 | 1.54E-07 | 1.31E-06 |
| ENSECAG00000045559 | 1.33372415 | 0.01319832 | 0.02962925 |
| ENSECAG00000020094 | 1.3329883 | 6.52E-05 | 0.00028881 |
| ENSECAG00000039203 | 1.33259697 | 6.40E-08 | 5.93E-07 |
| ENSECAG00000043336 | 1.33121582 | 0.0011124 | 0.00346426 |
| ENSECAG00000010426 | 1.33020352 | 1.28E-10 | 2.18E-09 |
| ENSECAG00000018278 | 1.32981939 | 2.63E-37 | 2.45E-34 |
| ENSECAG00000051713 | 1.3288739 | 0.00029005 | 0.00106902 |
| ENSECAG00000028937 | 1.32814138 | 1.40E-06 | 9.40E-06 |
| ENSECAG00000005570 | 1.32806996 | 1.12E-09 | 1.57E-08 |
| ENSECAG00000014124 | 1.32806991 | 7.57E-14 | 2.27E-12 |
| ENSECAG00000001634 | 1.32605363 | 7.18E-13 | 1.82E-11 |
| ENSECAG00000053444 | 1.3252629 | 0.00382461 | 0.01021572 |
| ENSECAG00000038912 | 1.32459019 | 8.22E-17 | 3.98E-15 |
| ENSECAG00000060081 | 1.32171678 | 0.01349523 | 0.03017153 |
| ENSECAG00000047679 | 1.32004996 | 1.30E-05 | 6.83E-05 |
| ENSECAG00000011406 | 1.31848692 | 6.02E-07 | 4.42E-06 |
| ENSECAG00000010207 | 1.31838008 | 0.0136563 | 0.03049808 |
| ENSECAG00000024991 | 1.31813708 | 7.88E-11 | 1.40E-09 |
| ENSECAG00000009296 | 1.31785427 | 8.61E-05 | 0.00036902 |
| ENSECAG00000050998 | 1.31765894 | 7.97E-06 | 4.40E-05 |
| ENSECAG00000037937 | 1.31646162 | 0.00028334 | 0.00104705 |
| ENSECAG00000011392 | 1.31602396 | 0.0204391 | 0.04312468 |
| ENSECAG00000007722 | 1.31460912 | 0.01314702 | 0.0295342 |
| ENSECAG00000037807 | 1.31446918 | 0.0002078 | 0.00079661 |
| ENSECAG00000052635 | 1.3138312 | 0.00062256 | 0.00208403 |
| ENSECAG00000059599 | 1.31342786 | 0.0042827 | 0.01125896 |
| ENSECAG00000058457 | 1.31317218 | 5.45E-05 | 0.00024568 |
| ENSECAG00000048969 | 1.31235592 | 1.75E-08 | 1.88E-07 |
| ENSECAG00000022407 | 1.31229668 | 2.04E-05 | 0.00010211 |
| ENSECAG00000045573 | 1.31178615 | 0.00795227 | 0.01922759 |
| ENSECAG00000007696 | 1.30989599 | 0.0003912 | 0.00138518 |
| ENSECAG00000036591 | 1.3096947 | 0.00122569 | 0.00377627 |
| ENSECAG00000000119 | 1.30898591 | 6.62E-09 | 7.87E-08 |
| ENSECAG00000060042 | 1.30889462 | 5.78E-05 | 0.00025845 |
| ENSECAG00000044075 | 1.30850127 | 0.00064606 | 0.00214994 |
| ENSECAG00000018108 | 1.30650427 | 2.84E-05 | 0.00013703 |
| ENSECAG00000022845 | 1.30620245 | 1.68E-05 | 8.58E-05 |
| ENSECAG00000015814 | 1.305558 | 1.28E-08 | 1.41E-07 |
| ENSECAG00000057790 | 1.30528385 | 6.15E-07 | 4.50E-06 |
| ENSECAG00000017907 | 1.30486948 | 5.12E-12 | 1.11E-10 |
| ENSECAG00000058986 | 1.30394894 | 0.02078977 | 0.04373642 |
| ENSECAG00000020252 | 1.30281408 | 7.92E-34 | 4.14E-31 |
| ENSECAG00000058674 | 1.30203337 | 0.00049985 | 0.00171313 |
| ENSECAG00000010263 | 1.30147818 | 0.00156496 | 0.00467647 |
| ENSECAG00000000343 | 1.30142121 | 1.23E-05 | 6.49E-05 |
| ENSECAG00000003816 | 1.30123906 | 0.00421772 | 0.01110121 |
| ENSECAG00000013724 | 1.30112531 | 2.30E-08 | 2.40E-07 |
| ENSECAG00000018927 | 1.3008664 | 0.00036861 | 0.00131483 |
| ENSECAG00000008723 | 1.29932662 | 0.0001548 | 0.00061574 |
| ENSECAG00000049167 | 1.29851219 | 0.00671212 | 0.01660456 |
| ENSECAG00000019217 | 1.298069 | 6.86E-17 | 3.39E-15 |
| ENSECAG00000038020 | 1.29759261 | 0.00034996 | 0.0012599 |
| ENSECAG00000022644 | 1.29737095 | 0.00855362 | 0.02047682 |
| ENSECAG00000012128 | 1.2967916 | 0.00012727 | 0.00051831 |
| ENSECAG00000030028 | 1.29508653 | 0.00332293 | 0.00903225 |
| ENSECAG00000024363 | 1.29363961 | 1.80E-19 | 1.39E-17 |
| ENSECAG00000020007 | 1.29359338 | 1.01E-06 | 7.03E-06 |
| ENSECAG00000001114 | 1.29311504 | 0.0006319 | 0.00211036 |
| ENSECAG00000034424 | 1.29279029 | 0.00012686 | 0.00051711 |
| ENSECAG00000021913 | 1.29202911 | 4.68E-18 | 2.82E-16 |
| ENSECAG00000019222 | 1.29161555 | 0.00036062 | 0.00129183 |
| ENSECAG00000002943 | 1.29085874 | 5.84E-09 | 7.04E-08 |
| ENSECAG00000039032 | 1.28874126 | 4.58E-06 | 2.68E-05 |
| ENSECAG00000006964 | 1.28830912 | 1.35E-21 | 1.38E-19 |
| ENSECAG00000003473 | 1.28819443 | 6.27E-08 | 5.83E-07 |
| ENSECAG00000030452 | 1.28797451 | 2.02E-08 | 2.14E-07 |
| ENSECAG00000017839 | 1.28738556 | 9.34E-05 | 0.00039648 |
| ENSECAG00000007317 | 1.28378411 | 0.00030912 | 0.00113056 |
| ENSECAG00000002438 | 1.28377441 | 0.00217126 | 0.00624439 |
| ENSECAG00000045453 | 1.2833228 | 0.01842547 | 0.0394647 |
| ENSECAG00000013223 | 1.28299653 | 0.00238762 | 0.00677766 |
| ENSECAG00000015115 | 1.28291581 | 1.27E-08 | 1.40E-07 |
| ENSECAG00000011223 | 1.28247703 | 0.00111237 | 0.00346426 |
| ENSECAG00000021844 | 1.28236596 | 0.00400979 | 0.01063283 |
| ENSECAG00000045968 | 1.28166418 | 4.24E-08 | 4.11E-07 |
| ENSECAG00000060333 | 1.28107419 | 0.00112331 | 0.00349247 |
| ENSECAG00000022632 | 1.28047461 | 0.00079666 | 0.00258864 |
| ENSECAG00000037430 | 1.27912912 | 4.77E-10 | 7.22E-09 |
| ENSECAG00000053473 | 1.27911381 | 0.00447608 | 0.01169444 |
| ENSECAG00000037336 | 1.27901666 | 0.00141168 | 0.00427965 |
| ENSECAG00000027206 | 1.27888747 | 0.01645027 | 0.03578936 |
| ENSECAG00000041433 | 1.27841416 | 3.66E-11 | 6.90E-10 |
| ENSECAG00000055991 | 1.27770922 | 0.00505828 | 0.01301916 |
| ENSECAG00000012293 | 1.27737123 | 0.00655296 | 0.01627388 |
| ENSECAG00000010698 | 1.27703897 | 0.0064809 | 0.01611925 |
| ENSECAG00000055105 | 1.27602949 | 2.94E-12 | 6.62E-11 |
| ENSECAG00000007589 | 1.27550144 | 3.77E-21 | 3.50E-19 |
| ENSECAG00000054412 | 1.27521128 | 3.12E-06 | 1.92E-05 |
| ENSECAG00000059362 | 1.2750809 | 0.01832118 | 0.03927189 |
| ENSECAG00000011891 | 1.27382106 | 8.95E-07 | 6.29E-06 |
| ENSECAG00000009642 | 1.27370202 | 0.00083348 | 0.00269731 |
| ENSECAG00000043865 | 1.27245568 | 0.00161849 | 0.00481389 |
| ENSECAG00000004386 | 1.27180993 | 3.37E-09 | 4.27E-08 |
| ENSECAG00000034172 | 1.27160232 | 0.01677588 | 0.03638846 |
| ENSECAG00000015846 | 1.27153033 | 2.70E-06 | 1.69E-05 |
| ENSECAG00000020970 | 1.27149734 | 1.90E-07 | 1.58E-06 |
| ENSECAG00000005017 | 1.27000185 | 5.61E-10 | 8.40E-09 |
| ENSECAG00000041577 | 1.26995679 | 7.46E-11 | 1.33E-09 |
| ENSECAG00000020653 | 1.26995186 | 1.88E-30 | 6.46E-28 |
| ENSECAG00000000381 | 1.26783678 | 3.89E-07 | 2.99E-06 |
| ENSECAG00000008762 | 1.26304318 | 0.00012211 | 0.00050069 |
| ENSECAG00000049483 | 1.26293435 | 2.88E-05 | 0.0001387 |
| ENSECAG00000005282 | 1.26265108 | 0.00621008 | 0.01554329 |
| ENSECAG00000020147 | 1.26264866 | 7.61E-10 | 1.10E-08 |
| ENSECAG00000045421 | 1.26255021 | 0.00010152 | 0.0004269 |
| ENSECAG00000016346 | 1.26148299 | 6.65E-26 | 1.30E-23 |
| ENSECAG00000012502 | 1.26073427 | 1.03E-05 | 5.53E-05 |
| ENSECAG00000017871 | 1.26038376 | 1.16E-08 | 1.30E-07 |
| ENSECAG00000013740 | 1.26015173 | 1.95E-24 | 3.05E-22 |
| ENSECAG00000019958 | 1.25947754 | 0.00022971 | 0.00087076 |
| ENSECAG00000052302 | 1.25857473 | 1.34E-37 | 1.30E-34 |
| ENSECAG00000054860 | 1.25851608 | 0.0002766 | 0.00102538 |
| ENSECAG00000042596 | 1.2581321 | 2.72E-08 | 2.77E-07 |
| ENSECAG00000005235 | 1.25768317 | 2.01E-08 | 2.13E-07 |
| ENSECAG00000022461 | 1.25740185 | 1.00E-32 | 4.56E-30 |
| ENSECAG00000020967 | 1.2571414 | 0.0015454 | 0.00462722 |
| ENSECAG00000042315 | 1.25699141 | 0.02171424 | 0.04535675 |
| ENSECAG00000009951 | 1.2569497 | 0.00279489 | 0.00775257 |
| ENSECAG00000057925 | 1.25658996 | 1.03E-10 | 1.79E-09 |
| ENSECAG00000004442 | 1.25586868 | 0.00139309 | 0.00423078 |
| ENSECAG00000019305 | 1.25553694 | 9.08E-05 | 0.00038732 |
| ENSECAG00000049296 | 1.255417 | 1.21E-21 | 1.23E-19 |
| ENSECAG00000048674 | 1.25523465 | 0.0235244 | 0.04852618 |
| ENSECAG00000017827 | 1.25473597 | 0.00021215 | 0.00081149 |
| ENSECAG00000017538 | 1.25470554 | 3.25E-10 | 5.09E-09 |
| ENSECAG00000037376 | 1.25436865 | 0.00010636 | 0.00044483 |
| ENSECAG00000047914 | 1.25316988 | 0.00851947 | 0.02040691 |
| ENSECAG00000010227 | 1.2529338 | 1.21E-08 | 1.35E-07 |
| ENSECAG00000010466 | 1.25260445 | 0.01921545 | 0.04086689 |
| ENSECAG00000015369 | 1.24899288 | 9.26E-10 | 1.32E-08 |
| ENSECAG00000031864 | 1.24809889 | 4.08E-06 | 2.43E-05 |
| ENSECAG00000050143 | 1.24799455 | 2.27E-05 | 0.00011246 |
| ENSECAG00000021480 | 1.24755756 | 3.53E-05 | 0.00016637 |
| ENSECAG00000044733 | 1.24707065 | 0.00025201 | 0.00094533 |
| ENSECAG00000037147 | 1.24705799 | 8.87E-05 | 0.000379 |
| ENSECAG00000016154 | 1.24622077 | 0.00997435 | 0.02331697 |
| ENSECAG00000011126 | 1.24616883 | 0.00536806 | 0.01371067 |
| ENSECAG00000018338 | 1.24606684 | 4.87E-13 | 1.28E-11 |
| ENSECAG00000021595 | 1.24579912 | 6.08E-15 | 2.20E-13 |
| ENSECAG00000013335 | 1.24537851 | 0.01244086 | 0.02813515 |
| ENSECAG00000038429 | 1.24506173 | 1.07E-07 | 9.45E-07 |
| ENSECAG00000015550 | 1.24504911 | 2.42E-14 | 7.86E-13 |
| ENSECAG00000049618 | 1.2450078 | 0.00241235 | 0.00683657 |
| ENSECAG00000007788 | 1.24455153 | 1.63E-09 | 2.23E-08 |
| ENSECAG00000045263 | 1.2442257 | 0.00029871 | 0.00109658 |
| ENSECAG00000031921 | 1.24365547 | 6.26E-14 | 1.92E-12 |
| ENSECAG00000023241 | 1.24253495 | 0.00396933 | 0.01054948 |
| ENSECAG00000017903 | 1.24199869 | 0.00344871 | 0.00932946 |
| ENSECAG00000023738 | 1.24190357 | 5.19E-10 | 7.81E-09 |
| ENSECAG00000016535 | 1.24143883 | 0.00014903 | 0.00059595 |
| ENSECAG00000044967 | 1.2411857 | 7.64E-13 | 1.92E-11 |
| ENSECAG00000005914 | 1.24065186 | 0.01389929 | 0.03098337 |
| ENSECAG00000007704 | 1.24036101 | 3.76E-08 | 3.69E-07 |
| ENSECAG00000023280 | 1.24001998 | 3.73E-06 | 2.24E-05 |
| ENSECAG00000009610 | 1.23917428 | 1.61E-10 | 2.67E-09 |
| ENSECAG00000020325 | 1.23868778 | 0.00155636 | 0.00465463 |
| ENSECAG00000029306 | 1.23839842 | 0.01071109 | 0.02478771 |
| ENSECAG00000003466 | 1.23839474 | 2.35E-06 | 1.49E-05 |
| ENSECAG00000014760 | 1.23777576 | 3.67E-20 | 3.09E-18 |
| ENSECAG00000036090 | 1.23712396 | 6.99E-08 | 6.43E-07 |
| ENSECAG00000043808 | 1.23581402 | 0.00278044 | 0.00772049 |
| ENSECAG00000045743 | 1.23568985 | 0.00646579 | 0.01609319 |
| ENSECAG00000044562 | 1.23513656 | 1.66E-14 | 5.57E-13 |
| ENSECAG00000019768 | 1.23430379 | 7.82E-05 | 0.00033912 |
| ENSECAG00000021968 | 1.23408805 | 5.20E-06 | 3.01E-05 |
| ENSECAG00000024310 | 1.23230816 | 0.00017798 | 0.00069543 |
| ENSECAG00000054147 | 1.23230627 | 0.00611998 | 0.01534699 |
| ENSECAG00000000157 | 1.23158705 | 0.01217439 | 0.02761918 |
| ENSECAG00000021525 | 1.23140494 | 3.84E-07 | 2.96E-06 |
| ENSECAG00000010916 | 1.23108482 | 1.52E-06 | 1.01E-05 |
| ENSECAG00000007047 | 1.23095892 | 7.80E-05 | 0.00033862 |
| ENSECAG00000015317 | 1.2306941 | 5.36E-07 | 4.00E-06 |
| ENSECAG00000022498 | 1.22955612 | 3.10E-16 | 1.37E-14 |
| ENSECAG00000012106 | 1.2295276 | 2.15E-14 | 7.06E-13 |
| ENSECAG00000053742 | 1.22920994 | 0.00105911 | 0.00332074 |
| ENSECAG00000033385 | 1.22898294 | 0.00051856 | 0.00177284 |
| ENSECAG00000055081 | 1.22856485 | 0.00488198 | 0.01262209 |
| ENSECAG00000017561 | 1.22812091 | 7.23E-06 | 4.04E-05 |
| ENSECAG00000005510 | 1.2279947 | 0.02234993 | 0.04650633 |
| ENSECAG00000019499 | 1.22706999 | 0.0041322 | 0.01090436 |
| ENSECAG00000010601 | 1.22531994 | 2.67E-14 | 8.66E-13 |
| ENSECAG00000018961 | 1.22467573 | 0.00614166 | 0.01538409 |
| ENSECAG00000021588 | 1.22450155 | 2.18E-16 | 9.85E-15 |
| ENSECAG00000011559 | 1.2241704 | 3.51E-14 | 1.12E-12 |
| ENSECAG00000001214 | 1.22348798 | 0.00103087 | 0.00324724 |
| ENSECAG00000015460 | 1.2226166 | 0.00011353 | 0.00047093 |
| ENSECAG00000041756 | 1.22259657 | 0.00354697 | 0.00955753 |
| ENSECAG00000055679 | 1.22257135 | 0.00679288 | 0.01676722 |
| ENSECAG00000046331 | 1.2218357 | 0.00427511 | 0.01124035 |
| ENSECAG00000045885 | 1.22177227 | 1.68E-07 | 1.42E-06 |
| ENSECAG00000019437 | 1.22171471 | 0.0004761 | 0.00164357 |
| ENSECAG00000052364 | 1.22169364 | 0.01192183 | 0.0271648 |
| ENSECAG00000018294 | 1.22143436 | 2.23E-10 | 3.61E-09 |
| ENSECAG00000008056 | 1.22075576 | 1.07E-08 | 1.20E-07 |
| ENSECAG00000036597 | 1.22067718 | 0.01429586 | 0.03172031 |
| ENSECAG00000020163 | 1.22044363 | 0.01252656 | 0.02828032 |
| ENSECAG00000037655 | 1.2200909 | 0.02139194 | 0.04479171 |
| ENSECAG00000001989 | 1.22001814 | 2.10E-11 | 4.10E-10 |
| ENSECAG00000012163 | 1.21998752 | 4.31E-13 | 1.14E-11 |
| ENSECAG00000017760 | 1.21955427 | 2.09E-06 | 1.34E-05 |
| ENSECAG00000013261 | 1.21950091 | 6.49E-07 | 4.73E-06 |
| ENSECAG00000030778 | 1.21757035 | 5.82E-08 | 5.44E-07 |
| ENSECAG00000016534 | 1.21749322 | 0.0026799 | 0.00748749 |
| ENSECAG00000050212 | 1.21709812 | 0.00687872 | 0.01695288 |
| ENSECAG00000058549 | 1.21638259 | 0.00629775 | 0.0157398 |
| ENSECAG00000020886 | 1.216382 | 3.29E-10 | 5.14E-09 |
| ENSECAG00000050191 | 1.21632365 | 0.00065627 | 0.00217937 |
| ENSECAG00000046475 | 1.21572383 | 7.34E-19 | 5.06E-17 |
| ENSECAG00000020104 | 1.21552998 | 1.24E-15 | 5.07E-14 |
| ENSECAG00000022098 | 1.2152602 | 1.22E-17 | 6.94E-16 |
| ENSECAG00000020728 | 1.21493235 | 5.68E-05 | 0.00025491 |
| ENSECAG00000018171 | 1.21486474 | 9.27E-07 | 6.50E-06 |
| ENSECAG00000025050 | 1.21426336 | 0.00015965 | 0.00063242 |
| ENSECAG00000013080 | 1.21393375 | 1.83E-18 | 1.18E-16 |
| ENSECAG00000001649 | 1.21263006 | 0.00228377 | 0.00652933 |
| ENSECAG00000008277 | 1.21234003 | 0.00090838 | 0.00290479 |
| ENSECAG00000008859 | 1.2120354 | 6.20E-08 | 5.77E-07 |
| ENSECAG00000019790 | 1.21087407 | 1.01E-05 | 5.44E-05 |
| ENSECAG00000059197 | 1.21083911 | 4.77E-05 | 0.00021835 |
| ENSECAG00000015840 | 1.21071804 | 0.00538869 | 0.01375707 |
| ENSECAG00000001394 | 1.210676 | 0.01788671 | 0.03845823 |
| ENSECAG00000010458 | 1.21022943 | 1.25E-10 | 2.13E-09 |
| ENSECAG00000024637 | 1.20992443 | 0.00072818 | 0.0023887 |
| ENSECAG00000003511 | 1.20807539 | 0.00125116 | 0.00384486 |
| ENSECAG00000018236 | 1.20758272 | 2.11E-17 | 1.15E-15 |
| ENSECAG00000008446 | 1.20738603 | 1.51E-05 | 7.78E-05 |
| ENSECAG00000047415 | 1.20702805 | 0.02081454 | 0.04377204 |
| ENSECAG00000008085 | 1.20683724 | 0.00155931 | 0.00466161 |
| ENSECAG00000019938 | 1.20683371 | 6.94E-19 | 4.85E-17 |
| ENSECAG00000008485 | 1.2059225 | 0.00013218 | 0.00053568 |
| ENSECAG00000007960 | 1.20548067 | 3.52E-05 | 0.00016592 |
| ENSECAG00000018455 | 1.20546766 | 0.00158729 | 0.00473434 |
| ENSECAG00000033498 | 1.20510796 | 0.00442454 | 0.01157821 |
| ENSECAG00000012064 | 1.2041293 | 0.00360473 | 0.00969797 |
| ENSECAG00000053434 | 1.20412813 | 0.01075056 | 0.02486101 |
| ENSECAG00000057503 | 1.20252593 | 2.62E-11 | 5.04E-10 |
| ENSECAG00000004611 | 1.20070509 | 0.00504704 | 0.01299323 |
| ENSECAG00000019633 | 1.20044033 | 0.00122402 | 0.00377164 |
| ENSECAG00000060149 | 1.20007343 | 1.02E-09 | 1.45E-08 |
| ENSECAG00000036791 | 1.19977693 | 0.00546054 | 0.01391347 |
| ENSECAG00000022971 | 1.19936596 | 0.00430959 | 0.011323 |
| ENSECAG00000049724 | 1.1990424 | 0.00116931 | 0.00362206 |
| ENSECAG00000001166 | 1.19889849 | 0.00013575 | 0.00054805 |
| ENSECAG00000007609 | 1.19689752 | 1.82E-12 | 4.29E-11 |
| ENSECAG00000030455 | 1.19642437 | 0.00103537 | 0.00325958 |
| ENSECAG00000052667 | 1.19611545 | 2.10E-05 | 0.00010493 |
| ENSECAG00000012913 | 1.19436248 | 0.01234869 | 0.02796334 |
| ENSECAG00000057564 | 1.19390666 | 9.79E-05 | 0.00041322 |
| ENSECAG00000019453 | 1.19314166 | 7.65E-17 | 3.73E-15 |
| ENSECAG00000034290 | 1.19290135 | 2.79E-07 | 2.23E-06 |
| ENSECAG00000017763 | 1.19231006 | 2.10E-07 | 1.73E-06 |
| ENSECAG00000024918 | 1.19108788 | 0.00053516 | 0.00182213 |
| ENSECAG00000016959 | 1.19095649 | 4.20E-09 | 5.24E-08 |
| ENSECAG00000009479 | 1.1902836 | 0.00051917 | 0.00177445 |
| ENSECAG00000021192 | 1.18935853 | 3.41E-06 | 2.07E-05 |
| ENSECAG00000010884 | 1.18924714 | 9.38E-10 | 1.34E-08 |
| ENSECAG00000010068 | 1.18908911 | 1.28E-05 | 6.71E-05 |
| ENSECAG00000016693 | 1.18861157 | 6.30E-17 | 3.14E-15 |
| ENSECAG00000021888 | 1.18785607 | 0.00388697 | 0.01036371 |
| ENSECAG00000053446 | 1.18765524 | 0.00034398 | 0.00124156 |
| ENSECAG00000018381 | 1.18535281 | 1.53E-26 | 3.23E-24 |
| ENSECAG00000008957 | 1.18159436 | 0.00015805 | 0.00062731 |
| ENSECAG00000010999 | 1.18153276 | 2.03E-08 | 2.15E-07 |
| ENSECAG00000047155 | 1.18086877 | 0.00015063 | 0.00060106 |
| ENSECAG00000022275 | 1.18028491 | 2.53E-13 | 6.96E-12 |
| ENSECAG00000052343 | 1.17907355 | 0.0021586 | 0.00621197 |
| ENSECAG00000004219 | 1.17785046 | 0.00158059 | 0.0047175 |
| ENSECAG00000024593 | 1.17756496 | 0.02110409 | 0.04431007 |
| ENSECAG00000000599 | 1.17698416 | 2.07E-07 | 1.71E-06 |
| ENSECAG00000043276 | 1.17675946 | 0.00053484 | 0.00182171 |
| ENSECAG00000007610 | 1.17666737 | 0.00312883 | 0.00854591 |
| ENSECAG00000022716 | 1.17651653 | 0.00285386 | 0.0078975 |
| ENSECAG00000023779 | 1.17609384 | 9.32E-19 | 6.30E-17 |
| ENSECAG00000022675 | 1.17552406 | 5.81E-23 | 7.05E-21 |
| ENSECAG00000009372 | 1.17512521 | 0.00034479 | 0.00124368 |
| ENSECAG00000018189 | 1.17480971 | 5.38E-15 | 1.97E-13 |
| ENSECAG00000020685 | 1.17471554 | 0.00032879 | 0.0011929 |
| ENSECAG00000060049 | 1.17385469 | 7.85E-06 | 4.35E-05 |
| ENSECAG00000015128 | 1.1738523 | 3.67E-07 | 2.84E-06 |
| ENSECAG00000010406 | 1.17382529 | 0.00364765 | 0.00980398 |
| ENSECAG00000004162 | 1.17359909 | 0.00148876 | 0.00447937 |
| ENSECAG00000011111 | 1.17344807 | 4.89E-08 | 4.67E-07 |
| ENSECAG00000051336 | 1.17305515 | 0.01864235 | 0.03984905 |
| ENSECAG00000011401 | 1.17297856 | 0.00032802 | 0.00119081 |
| ENSECAG00000024065 | 1.17225297 | 0.015655 | 0.03433133 |
| ENSECAG00000018711 | 1.17218561 | 7.27E-07 | 5.23E-06 |
| ENSECAG00000011656 | 1.17144498 | 1.42E-06 | 9.54E-06 |
| ENSECAG00000046524 | 1.1707544 | 4.11E-25 | 6.95E-23 |
| ENSECAG00000015524 | 1.17051656 | 0.00048132 | 0.00165931 |
| ENSECAG00000043788 | 1.17028637 | 2.35E-07 | 1.91E-06 |
| ENSECAG00000011512 | 1.17020955 | 8.27E-09 | 9.56E-08 |
| ENSECAG00000007283 | 1.16926259 | 0.00991469 | 0.0231969 |
| ENSECAG00000019512 | 1.16904268 | 5.36E-07 | 4.00E-06 |
| ENSECAG00000049142 | 1.16676063 | 0.01257508 | 0.02837838 |
| ENSECAG00000007148 | 1.16616195 | 8.46E-05 | 0.00036333 |
| ENSECAG00000016811 | 1.16555085 | 0.00011944 | 0.00049152 |
| ENSECAG00000024614 | 1.16525293 | 1.58E-15 | 6.35E-14 |
| ENSECAG00000002165 | 1.16482891 | 1.39E-13 | 3.96E-12 |
| ENSECAG00000007470 | 1.16406158 | 0.00601956 | 0.01512572 |
| ENSECAG00000007492 | 1.16399086 | 1.33E-08 | 1.47E-07 |
| ENSECAG00000007007 | 1.16386188 | 5.09E-12 | 1.11E-10 |
| ENSECAG00000012769 | 1.16299098 | 2.41E-24 | 3.71E-22 |
| ENSECAG00000009492 | 1.16268213 | 2.45E-21 | 2.33E-19 |
| ENSECAG00000000773 | 1.16232138 | 7.83E-16 | 3.26E-14 |
| ENSECAG00000022197 | 1.16134649 | 2.71E-10 | 4.30E-09 |
| ENSECAG00000010809 | 1.16096181 | 2.60E-17 | 1.40E-15 |
| ENSECAG00000010804 | 1.16053755 | 0.00053947 | 0.00183543 |
| ENSECAG00000016635 | 1.16031753 | 1.44E-12 | 3.47E-11 |
| ENSECAG00000011874 | 1.16011853 | 9.63E-18 | 5.57E-16 |
| ENSECAG00000004992 | 1.16009191 | 9.29E-33 | 4.32E-30 |
| ENSECAG00000019520 | 1.15992254 | 3.78E-16 | 1.64E-14 |
| ENSECAG00000021162 | 1.15976729 | 0.00023271 | 0.00088049 |
| ENSECAG00000022687 | 1.15867004 | 1.55E-17 | 8.66E-16 |
| ENSECAG00000055095 | 1.15852712 | 0.00294444 | 0.00811901 |
| ENSECAG00000006921 | 1.15813465 | 4.77E-07 | 3.60E-06 |
| ENSECAG00000006668 | 1.15805719 | 0.00747616 | 0.01820643 |
| ENSECAG00000001373 | 1.15783801 | 1.37E-06 | 9.24E-06 |
| ENSECAG00000032362 | 1.1572524 | 0.01251902 | 0.02826614 |
| ENSECAG00000019868 | 1.15696563 | 0.00089674 | 0.00287374 |
| ENSECAG00000023356 | 1.15626132 | 6.90E-12 | 1.47E-10 |
| ENSECAG00000021098 | 1.15614284 | 0.00076408 | 0.00249292 |
| ENSECAG00000013597 | 1.15590016 | 8.07E-06 | 4.45E-05 |
| ENSECAG00000005768 | 1.15529004 | 5.76E-11 | 1.05E-09 |
| ENSECAG00000014435 | 1.15515813 | 0.00017906 | 0.00069865 |
| ENSECAG00000015469 | 1.15507751 | 4.10E-10 | 6.26E-09 |
| ENSECAG00000049739 | 1.15474274 | 0.001771 | 0.00521819 |
| ENSECAG00000049214 | 1.15463796 | 0.00215289 | 0.00619878 |
| ENSECAG00000022862 | 1.15435519 | 0.00854036 | 0.02045475 |
| ENSECAG00000015202 | 1.15375523 | 1.15E-09 | 1.61E-08 |
| ENSECAG00000018404 | 1.15366449 | 0.00049612 | 0.00170243 |
| ENSECAG00000004316 | 1.15313073 | 0.00017965 | 0.00070059 |
| ENSECAG00000021658 | 1.15291914 | 5.37E-20 | 4.48E-18 |
| ENSECAG00000010620 | 1.15235378 | 3.66E-07 | 2.83E-06 |
| ENSECAG00000059184 | 1.15204309 | 0.00095815 | 0.00304562 |
| ENSECAG00000012045 | 1.15172284 | 3.54E-14 | 1.13E-12 |
| ENSECAG00000028930 | 1.15158023 | 1.04E-14 | 3.64E-13 |
| ENSECAG00000023632 | 1.15150484 | 3.22E-07 | 2.53E-06 |
| ENSECAG00000040395 | 1.1508185 | 0.01467677 | 0.03246236 |
| ENSECAG00000046638 | 1.15066751 | 0.00125442 | 0.00385363 |
| ENSECAG00000000390 | 1.15039835 | 2.49E-08 | 2.57E-07 |
| ENSECAG00000052175 | 1.15021937 | 0.00081726 | 0.0026479 |
| ENSECAG00000049992 | 1.14961453 | 0.00148938 | 0.00448056 |
| ENSECAG00000049085 | 1.14916161 | 2.64E-05 | 0.00012848 |
| ENSECAG00000031341 | 1.14786741 | 3.36E-25 | 5.91E-23 |
| ENSECAG00000015351 | 1.14504765 | 0.0108538 | 0.02507122 |
| ENSECAG00000018024 | 1.14499708 | 0.00023436 | 0.00088642 |
| ENSECAG00000024727 | 1.14457718 | 0.00108456 | 0.00338816 |
| ENSECAG00000024909 | 1.1445248 | 0.00045143 | 0.00157078 |
| ENSECAG00000057245 | 1.14437862 | 3.13E-05 | 0.00014947 |
| ENSECAG00000020307 | 1.14411299 | 3.07E-07 | 2.42E-06 |
| ENSECAG00000023441 | 1.14361995 | 2.17E-07 | 1.78E-06 |
| ENSECAG00000016064 | 1.14298677 | 5.56E-13 | 1.44E-11 |
| ENSECAG00000002988 | 1.14111486 | 0.00012086 | 0.0004963 |
| ENSECAG00000010539 | 1.13916883 | 1.69E-09 | 2.32E-08 |
| ENSECAG00000017661 | 1.13879574 | 3.73E-07 | 2.89E-06 |
| ENSECAG00000039059 | 1.1384507 | 5.26E-16 | 2.22E-14 |
| ENSECAG00000022166 | 1.13836022 | 0.02141567 | 0.04482108 |
| ENSECAG00000025502 | 1.13818693 | 0.00595051 | 0.01497074 |
| ENSECAG00000009699 | 1.13798471 | 0.00023078 | 0.00087424 |
| ENSECAG00000043880 | 1.13754602 | 0.01954369 | 0.04147025 |
| ENSECAG00000009638 | 1.13603103 | 0.00055226 | 0.00187323 |
| ENSECAG00000018530 | 1.13602465 | 5.77E-10 | 8.64E-09 |
| ENSECAG00000020812 | 1.13590601 | 0.02139362 | 0.04479171 |
| ENSECAG00000046018 | 1.13570126 | 0.01938356 | 0.04116957 |
| ENSECAG00000010118 | 1.13538801 | 5.06E-07 | 3.80E-06 |
| ENSECAG00000015792 | 1.13467931 | 0.00072006 | 0.00236312 |
| ENSECAG00000007179 | 1.13465019 | 2.02E-23 | 2.63E-21 |
| ENSECAG00000006663 | 1.13332405 | 0.00715724 | 0.01754674 |
| ENSECAG00000045761 | 1.13328732 | 0.00064221 | 0.00213936 |
| ENSECAG00000010951 | 1.13225308 | 4.81E-08 | 4.61E-07 |
| ENSECAG00000013746 | 1.13215581 | 1.89E-06 | 1.23E-05 |
| ENSECAG00000002414 | 1.13196287 | 1.09E-08 | 1.23E-07 |
| ENSECAG00000045773 | 1.13096052 | 5.94E-09 | 7.14E-08 |
| ENSECAG00000011659 | 1.13047111 | 0.00240989 | 0.00683046 |
| ENSECAG00000027909 | 1.13033552 | 9.84E-10 | 1.40E-08 |
| ENSECAG00000058983 | 1.13019883 | 0.00018677 | 0.00072444 |
| ENSECAG00000033122 | 1.13009937 | 2.74E-08 | 2.79E-07 |
| ENSECAG00000022050 | 1.12930311 | 1.56E-06 | 1.04E-05 |
| ENSECAG00000040088 | 1.12898956 | 2.47E-12 | 5.67E-11 |
| ENSECAG00000022011 | 1.12885737 | 3.53E-05 | 0.0001666 |
| ENSECAG00000017906 | 1.12837695 | 0.00759542 | 0.01845663 |
| ENSECAG00000060213 | 1.12780619 | 3.02E-05 | 0.00014484 |
| ENSECAG00000012523 | 1.12703296 | 3.33E-11 | 6.32E-10 |
| ENSECAG00000008447 | 1.12583043 | 9.62E-14 | 2.82E-12 |
| ENSECAG00000020193 | 1.12311705 | 1.81E-05 | 9.19E-05 |
| ENSECAG00000011614 | 1.12207313 | 7.36E-12 | 1.56E-10 |
| ENSECAG00000034536 | 1.12203357 | 0.00339136 | 0.00919702 |
| ENSECAG00000022708 | 1.12137738 | 0.00048905 | 0.00168231 |
| ENSECAG00000010326 | 1.12115064 | 0.00941084 | 0.02219235 |
| ENSECAG00000008517 | 1.12019117 | 0.00851811 | 0.02040585 |
| ENSECAG00000011471 | 1.11965405 | 1.50E-08 | 1.63E-07 |
| ENSECAG00000047772 | 1.11929824 | 1.59E-08 | 1.72E-07 |
| ENSECAG00000015687 | 1.11830538 | 7.48E-08 | 6.82E-07 |
| ENSECAG00000059977 | 1.11766808 | 3.26E-10 | 5.11E-09 |
| ENSECAG00000003056 | 1.11665369 | 0.00016528 | 0.00065244 |
| ENSECAG00000024201 | 1.11613553 | 0.00382003 | 0.01020838 |
| ENSECAG00000001035 | 1.11566149 | 5.45E-14 | 1.69E-12 |
| ENSECAG00000058276 | 1.11559943 | 1.49E-05 | 7.69E-05 |
| ENSECAG00000015049 | 1.11506226 | 2.08E-16 | 9.47E-15 |
| ENSECAG00000002569 | 1.11408202 | 0.00042869 | 0.0015015 |
| ENSECAG00000012899 | 1.11318443 | 0.00137372 | 0.00418104 |
| ENSECAG00000013359 | 1.11291013 | 2.95E-16 | 1.31E-14 |
| ENSECAG00000006670 | 1.11121769 | 3.96E-23 | 4.89E-21 |
| ENSECAG00000012646 | 1.11066022 | 1.38E-05 | 7.22E-05 |
| ENSECAG00000033049 | 1.11055033 | 2.07E-19 | 1.57E-17 |
| ENSECAG00000023182 | 1.11041323 | 1.38E-10 | 2.34E-09 |
| ENSECAG00000020942 | 1.11002015 | 0.01554747 | 0.03413478 |
| ENSECAG00000023479 | 1.10931332 | 1.18E-10 | 2.03E-09 |
| ENSECAG00000003331 | 1.10914137 | 4.28E-06 | 2.53E-05 |
| ENSECAG00000013779 | 1.10903013 | 8.17E-12 | 1.72E-10 |
| ENSECAG00000000081 | 1.10897588 | 1.21E-05 | 6.37E-05 |
| ENSECAG00000054242 | 1.1085909 | 0.00239989 | 0.00680904 |
| ENSECAG00000014826 | 1.10739348 | 1.82E-19 | 1.40E-17 |
| ENSECAG00000007999 | 1.10725932 | 2.09E-23 | 2.70E-21 |
| ENSECAG00000013663 | 1.1070108 | 0.00028844 | 0.00106451 |
| ENSECAG00000054990 | 1.10700499 | 0.0018784 | 0.00549694 |
| ENSECAG00000016570 | 1.10660438 | 0.00103958 | 0.00327005 |
| ENSECAG00000000666 | 1.10623137 | 4.47E-06 | 2.63E-05 |
| ENSECAG00000050101 | 1.1059609 | 0.00014656 | 0.00058713 |
| ENSECAG00000034555 | 1.10580276 | 1.99E-12 | 4.66E-11 |
| ENSECAG00000024742 | 1.10563887 | 1.50E-10 | 2.51E-09 |
| ENSECAG00000017704 | 1.10425588 | 2.85E-16 | 1.27E-14 |
| ENSECAG00000016155 | 1.10414799 | 0.00068107 | 0.00225404 |
| ENSECAG00000025441 | 1.10383926 | 0.00056932 | 0.00192524 |
| ENSECAG00000007275 | 1.10355775 | 5.36E-08 | 5.07E-07 |
| ENSECAG00000015881 | 1.1034898 | 2.46E-16 | 1.11E-14 |
| ENSECAG00000016123 | 1.10321745 | 6.53E-24 | 9.54E-22 |
| ENSECAG00000008058 | 1.10299027 | 1.15E-08 | 1.28E-07 |
| ENSECAG00000010008 | 1.10237306 | 7.67E-06 | 4.26E-05 |
| ENSECAG00000013766 | 1.10231641 | 1.33E-05 | 6.98E-05 |
| ENSECAG00000051604 | 1.1022197 | 1.14E-05 | 6.09E-05 |
| ENSECAG00000010297 | 1.10184195 | 0.00611928 | 0.01534695 |
| ENSECAG00000022769 | 1.10096959 | 2.09E-08 | 2.20E-07 |
| ENSECAG00000056149 | 1.10067185 | 3.17E-05 | 0.00015133 |
| ENSECAG00000023150 | 1.10065713 | 1.03E-13 | 2.99E-12 |
| ENSECAG00000024654 | 1.10042769 | 5.31E-14 | 1.65E-12 |
| ENSECAG00000024675 | 1.10041535 | 1.16E-14 | 4.00E-13 |
| ENSECAG00000049465 | 1.09916096 | 0.00746738 | 0.01818914 |
| ENSECAG00000055452 | 1.09710652 | 0.00125995 | 0.00386825 |
| ENSECAG00000017207 | 1.096871 | 6.08E-23 | 7.35E-21 |
| ENSECAG00000023105 | 1.09676023 | 1.63E-06 | 1.08E-05 |
| ENSECAG00000056471 | 1.09673037 | 2.96E-05 | 0.00014223 |
| ENSECAG00000023033 | 1.09657975 | 0.00052883 | 0.00180425 |
| ENSECAG00000013534 | 1.09530705 | 0.0006982 | 0.00230048 |
| ENSECAG00000016480 | 1.09456693 | 2.30E-19 | 1.73E-17 |
| ENSECAG00000016782 | 1.09435752 | 0.00204192 | 0.00591736 |
| ENSECAG00000014782 | 1.0938845 | 2.45E-11 | 4.75E-10 |
| ENSECAG00000017335 | 1.09354026 | 2.01E-09 | 2.71E-08 |
| ENSECAG00000024284 | 1.09350909 | 5.34E-11 | 9.76E-10 |
| ENSECAG00000013963 | 1.09298675 | 9.68E-13 | 2.40E-11 |
| ENSECAG00000013169 | 1.09246681 | 7.79E-12 | 1.65E-10 |
| ENSECAG00000024301 | 1.09207675 | 2.96E-05 | 0.00014246 |
| ENSECAG00000017522 | 1.09117547 | 0.00412915 | 0.01090105 |
| ENSECAG00000020856 | 1.09026267 | 3.75E-21 | 3.49E-19 |
| ENSECAG00000017156 | 1.08998405 | 2.33E-10 | 3.76E-09 |
| ENSECAG00000051470 | 1.08926675 | 0.01488368 | 0.03284846 |
| ENSECAG00000011229 | 1.08924346 | 0.00012455 | 0.00050906 |
| ENSECAG00000046810 | 1.08918994 | 1.15E-05 | 6.11E-05 |
| ENSECAG00000047474 | 1.08811457 | 0.00680315 | 0.01679072 |
| ENSECAG00000024148 | 1.08810326 | 0.00274123 | 0.00763118 |
| ENSECAG00000014391 | 1.08775888 | 2.84E-15 | 1.09E-13 |
| ENSECAG00000023256 | 1.08772037 | 0.00442135 | 0.01157173 |
| ENSECAG00000020411 | 1.08745183 | 0.02288394 | 0.04743642 |
| ENSECAG00000032753 | 1.08722802 | 0.01955208 | 0.04147779 |
| ENSECAG00000008438 | 1.08720939 | 0.00020731 | 0.00079515 |
| ENSECAG00000025034 | 1.08708856 | 1.88E-14 | 6.25E-13 |
| ENSECAG00000020739 | 1.08484946 | 1.14E-11 | 2.34E-10 |
| ENSECAG00000032438 | 1.08478939 | 2.77E-06 | 1.73E-05 |
| ENSECAG00000014218 | 1.08439727 | 0.01452286 | 0.03215693 |
| ENSECAG00000005924 | 1.08439025 | 2.04E-07 | 1.68E-06 |
| ENSECAG00000007542 | 1.08393803 | 5.11E-11 | 9.39E-10 |
| ENSECAG00000000587 | 1.08306331 | 0.00084398 | 0.00272583 |
| ENSECAG00000021012 | 1.0827612 | 4.34E-08 | 4.20E-07 |
| ENSECAG00000008730 | 1.08218866 | 2.22E-16 | 1.00E-14 |
| ENSECAG00000007859 | 1.0813172 | 1.43E-11 | 2.90E-10 |
| ENSECAG00000010728 | 1.07961918 | 0.00020143 | 0.00077618 |
| ENSECAG00000037399 | 1.07874723 | 0.0078228 | 0.01894529 |
| ENSECAG00000023967 | 1.07811636 | 2.53E-08 | 2.60E-07 |
| ENSECAG00000006343 | 1.07779354 | 1.60E-07 | 1.36E-06 |
| ENSECAG00000026834 | 1.07728176 | 3.96E-21 | 3.66E-19 |
| ENSECAG00000047525 | 1.07664929 | 0.0009081 | 0.0029043 |
| ENSECAG00000024776 | 1.07575707 | 2.34E-11 | 4.54E-10 |
| ENSECAG00000020196 | 1.07433874 | 6.44E-13 | 1.65E-11 |
| ENSECAG00000009402 | 1.07353374 | 0.00307911 | 0.00842794 |
| ENSECAG00000013011 | 1.07315867 | 1.80E-12 | 4.24E-11 |
| ENSECAG00000012455 | 1.0726699 | 1.87E-28 | 5.03E-26 |
| ENSECAG00000020551 | 1.07232856 | 0.00022988 | 0.00087125 |
| ENSECAG00000035596 | 1.0721715 | 0.00023685 | 0.00089492 |
| ENSECAG00000044826 | 1.07206907 | 0.00772451 | 0.01873975 |
| ENSECAG00000009716 | 1.07181869 | 0.00170902 | 0.00505892 |
| ENSECAG00000001898 | 1.07092977 | 9.71E-15 | 3.41E-13 |
| ENSECAG00000011563 | 1.0703727 | 7.01E-11 | 1.25E-09 |
| ENSECAG00000024871 | 1.07032406 | 7.29E-21 | 6.46E-19 |
| ENSECAG00000014825 | 1.07030121 | 1.32E-29 | 3.98E-27 |
| ENSECAG00000012612 | 1.07025944 | 1.63E-18 | 1.06E-16 |
| ENSECAG00000053638 | 1.06761834 | 0.01181802 | 0.02696676 |
| ENSECAG00000021295 | 1.06645871 | 1.11E-12 | 2.73E-11 |
| ENSECAG00000009302 | 1.06621594 | 1.52E-05 | 7.85E-05 |
| ENSECAG00000006038 | 1.06620825 | 0.00394185 | 0.01048877 |
| ENSECAG00000008506 | 1.06582052 | 0.00185566 | 0.00543752 |
| ENSECAG00000049135 | 1.06576074 | 1.33E-13 | 3.81E-12 |
| ENSECAG00000025117 | 1.06468487 | 0.00704771 | 0.01732923 |
| ENSECAG00000045790 | 1.06440442 | 2.58E-05 | 0.00012583 |
| ENSECAG00000041134 | 1.06403697 | 1.66E-21 | 1.65E-19 |
| ENSECAG00000020159 | 1.06357512 | 0.02193142 | 0.0457377 |
| ENSECAG00000024054 | 1.06304797 | 0.02104033 | 0.0441928 |
| ENSECAG00000020784 | 1.06236947 | 1.52E-08 | 1.64E-07 |
| ENSECAG00000014425 | 1.06210321 | 6.10E-21 | 5.45E-19 |
| ENSECAG00000019549 | 1.06191646 | 5.50E-05 | 0.00024767 |
| ENSECAG00000035852 | 1.06181979 | 4.53E-09 | 5.61E-08 |
| ENSECAG00000010284 | 1.06060049 | 1.59E-09 | 2.18E-08 |
| ENSECAG00000021086 | 1.06049724 | 3.68E-05 | 0.00017293 |
| ENSECAG00000009950 | 1.06035587 | 1.00E-27 | 2.46E-25 |
| ENSECAG00000003386 | 1.06015239 | 2.09E-07 | 1.72E-06 |
| ENSECAG00000015972 | 1.06011212 | 0.00073743 | 0.00241481 |
| ENSECAG00000047012 | 1.06009384 | 0.00393511 | 0.01047832 |
| ENSECAG00000014032 | 1.05944119 | 0.00031044 | 0.00113498 |
| ENSECAG00000015525 | 1.0589778 | 4.29E-12 | 9.39E-11 |
| ENSECAG00000012054 | 1.05853529 | 1.27E-09 | 1.77E-08 |
| ENSECAG00000050881 | 1.05805225 | 0.00558955 | 0.01419216 |
| ENSECAG00000018049 | 1.05743375 | 0.02029593 | 0.04286294 |
| ENSECAG00000044311 | 1.05658848 | 0.02030011 | 0.04286773 |
| ENSECAG00000020204 | 1.05656882 | 0.00040571 | 0.00142973 |
| ENSECAG00000056537 | 1.05649037 | 0.01466722 | 0.03244761 |
| ENSECAG00000007716 | 1.05620488 | 5.21E-15 | 1.91E-13 |
| ENSECAG00000016720 | 1.05610311 | 0.01200458 | 0.02729857 |
| ENSECAG00000026830 | 1.05582632 | 0.00057093 | 0.00193039 |
| ENSECAG00000022556 | 1.05460553 | 0.01031673 | 0.02401933 |
| ENSECAG00000014660 | 1.05445879 | 5.64E-06 | 3.24E-05 |
| ENSECAG00000024082 | 1.0543203 | 0.0096344 | 0.02264288 |
| ENSECAG00000010084 | 1.05430224 | 1.06E-12 | 2.62E-11 |
| ENSECAG00000002083 | 1.05395089 | 0.00038374 | 0.00136184 |
| ENSECAG00000009563 | 1.05266694 | 1.73E-08 | 1.86E-07 |
| ENSECAG00000021503 | 1.05206265 | 0.00054818 | 0.00186136 |
| ENSECAG00000008173 | 1.05179398 | 2.47E-09 | 3.25E-08 |
| ENSECAG00000017048 | 1.05172305 | 1.92E-05 | 9.68E-05 |
| ENSECAG00000054967 | 1.05165906 | 9.80E-10 | 1.39E-08 |
| ENSECAG00000019798 | 1.05132754 | 0.00014316 | 0.00057504 |
| ENSECAG00000020631 | 1.05127316 | 7.21E-17 | 3.53E-15 |
| ENSECAG00000032097 | 1.05114224 | 3.01E-05 | 0.00014479 |
| ENSECAG00000006321 | 1.05102196 | 4.90E-16 | 2.08E-14 |
| ENSECAG00000017104 | 1.05015054 | 0.00133425 | 0.00407423 |
| ENSECAG00000020689 | 1.04967123 | 0.00010468 | 0.00043913 |
| ENSECAG00000009723 | 1.04962315 | 0.00096825 | 0.00307292 |
| ENSECAG00000006573 | 1.04959522 | 1.56E-16 | 7.25E-15 |
| ENSECAG00000059252 | 1.04921405 | 1.90E-11 | 3.75E-10 |
| ENSECAG00000044184 | 1.04908375 | 1.07E-05 | 5.73E-05 |
| ENSECAG00000021603 | 1.0489687 | 0.00016673 | 0.00065675 |
| ENSECAG00000011090 | 1.04856266 | 0.00011475 | 0.00047478 |
| ENSECAG00000011212 | 1.04813017 | 0.00049303 | 0.00169341 |
| ENSECAG00000019968 | 1.0465067 | 8.06E-11 | 1.43E-09 |
| ENSECAG00000007941 | 1.04583345 | 1.00E-05 | 5.42E-05 |
| ENSECAG00000014474 | 1.04571387 | 0.00961984 | 0.02261599 |
| ENSECAG00000044441 | 1.04523415 | 0.00067652 | 0.00223997 |
| ENSECAG00000015885 | 1.04483177 | 0.00139105 | 0.00422594 |
| ENSECAG00000000153 | 1.04476538 | 6.19E-05 | 0.00027567 |
| ENSECAG00000005988 | 1.04409357 | 2.00E-09 | 2.69E-08 |
| ENSECAG00000007907 | 1.04394593 | 0.00119392 | 0.00368959 |
| ENSECAG00000032528 | 1.04364809 | 0.00487694 | 0.01261198 |
| ENSECAG00000021199 | 1.04351583 | 2.98E-30 | 9.78E-28 |
| ENSECAG00000026317 | 1.04279461 | 1.06E-16 | 5.11E-15 |
| ENSECAG00000008441 | 1.04261981 | 0.00409889 | 0.01083438 |
| ENSECAG00000012286 | 1.04232263 | 8.09E-16 | 3.36E-14 |
| ENSECAG00000020376 | 1.0421772 | 6.93E-10 | 1.02E-08 |
| ENSECAG00000017878 | 1.04191832 | 0.0105373 | 0.02445134 |
| ENSECAG00000001262 | 1.04190454 | 0.00063879 | 0.00212922 |
| ENSECAG00000026851 | 1.04189995 | 0.00099758 | 0.00315525 |
| ENSECAG00000022964 | 1.04154526 | 0.00061085 | 0.00204834 |
| ENSECAG00000010324 | 1.04145988 | 3.11E-13 | 8.42E-12 |
| ENSECAG00000009797 | 1.04045207 | 2.97E-07 | 2.35E-06 |
| ENSECAG00000001648 | 1.04017881 | 3.90E-19 | 2.83E-17 |
| ENSECAG00000015697 | 1.03993444 | 1.57E-10 | 2.62E-09 |
| ENSECAG00000060369 | 1.03958788 | 0.00119342 | 0.00368855 |
| ENSECAG00000014764 | 1.03856322 | 2.05E-12 | 4.78E-11 |
| ENSECAG00000055535 | 1.03828059 | 0.00203197 | 0.00589312 |
| ENSECAG00000047219 | 1.03802112 | 0.0116183 | 0.0265816 |
| ENSECAG00000042977 | 1.03754248 | 0.00016989 | 0.00066736 |
| ENSECAG00000017160 | 1.03713941 | 0.00011296 | 0.00046895 |
| ENSECAG00000013662 | 1.03601547 | 0.00855352 | 0.02047682 |
| ENSECAG00000000263 | 1.03575988 | 1.56E-08 | 1.69E-07 |
| ENSECAG00000017976 | 1.03514031 | 0.00114338 | 0.00354909 |
| ENSECAG00000045771 | 1.03478863 | 0.00248342 | 0.00700688 |
| ENSECAG00000005081 | 1.034576 | 0.010085 | 0.02353622 |
| ENSECAG00000053279 | 1.03439633 | 0.0033523 | 0.00910767 |
| ENSECAG00000034219 | 1.03411844 | 3.44E-06 | 2.09E-05 |
| ENSECAG00000021378 | 1.0340895 | 0.01521465 | 0.03347972 |
| ENSECAG00000017743 | 1.03324983 | 4.91E-05 | 0.00022384 |
| ENSECAG00000024881 | 1.03287741 | 0.00012326 | 0.00050464 |
| ENSECAG00000015006 | 1.03256594 | 7.29E-12 | 1.55E-10 |
| ENSECAG00000010260 | 1.0325155 | 1.54E-07 | 1.31E-06 |
| ENSECAG00000047268 | 1.03250131 | 0.00527021 | 0.0134916 |
| ENSECAG00000007021 | 1.0323616 | 2.02E-05 | 0.0001013 |
| ENSECAG00000008365 | 1.03195635 | 0.00307087 | 0.00840976 |
| ENSECAG00000010990 | 1.03185508 | 0.01157647 | 0.0265049 |
| ENSECAG00000021589 | 1.03169805 | 0.00047046 | 0.00162826 |
| ENSECAG00000008510 | 1.03090805 | 2.84E-07 | 2.26E-06 |
| ENSECAG00000033033 | 1.03072623 | 0.003109 | 0.00849934 |
| ENSECAG00000024474 | 1.03025553 | 2.96E-11 | 5.67E-10 |
| ENSECAG00000014564 | 1.02959371 | 6.49E-18 | 3.85E-16 |
| ENSECAG00000018755 | 1.02914339 | 1.73E-07 | 1.46E-06 |
| ENSECAG00000059109 | 1.02845681 | 0.00571003 | 0.01444378 |
| ENSECAG00000019880 | 1.02845675 | 0.00422981 | 0.01112909 |
| ENSECAG00000030795 | 1.02831203 | 0.02358517 | 0.04861939 |
| ENSECAG00000052631 | 1.02828272 | 0.01780153 | 0.03831194 |
| ENSECAG00000029009 | 1.02817666 | 0.00047223 | 0.00163326 |
| ENSECAG00000016545 | 1.02724309 | 0.00336169 | 0.00912761 |
| ENSECAG00000000927 | 1.02699507 | 0.00036688 | 0.00131027 |
| ENSECAG00000000371 | 1.02627018 | 1.97E-21 | 1.94E-19 |
| ENSECAG00000049894 | 1.02622223 | 9.69E-06 | 5.26E-05 |
| ENSECAG00000000440 | 1.02601895 | 1.10E-06 | 7.61E-06 |
| ENSECAG00000021604 | 1.02506678 | 0.00239332 | 0.00679211 |
| ENSECAG00000013623 | 1.0247835 | 0.00065813 | 0.00218489 |
| ENSECAG00000009415 | 1.02440039 | 1.06E-14 | 3.66E-13 |
| ENSECAG00000058428 | 1.02433232 | 0.01261564 | 0.02845842 |
| ENSECAG00000029864 | 1.02409439 | 8.03E-08 | 7.27E-07 |
| ENSECAG00000058005 | 1.02362776 | 2.85E-10 | 4.50E-09 |
| ENSECAG00000017172 | 1.02351332 | 0.00203108 | 0.00589129 |
| ENSECAG00000012816 | 1.02348634 | 8.96E-18 | 5.20E-16 |
| ENSECAG00000009561 | 1.02333969 | 1.49E-10 | 2.49E-09 |
| ENSECAG00000010902 | 1.02321219 | 1.06E-10 | 1.85E-09 |
| ENSECAG00000016512 | 1.02193216 | 1.41E-05 | 7.35E-05 |
| ENSECAG00000036666 | 1.02172618 | 1.05E-23 | 1.45E-21 |
| ENSECAG00000013897 | 1.02162771 | 0.01324397 | 0.02971681 |
| ENSECAG00000000360 | 1.02108135 | 0.00983923 | 0.02304933 |
| ENSECAG00000026754 | 1.02072417 | 0.00336071 | 0.00912607 |
| ENSECAG00000040129 | 1.02052467 | 0.00486984 | 0.01259944 |
| ENSECAG00000044152 | 1.02046059 | 0.00878305 | 0.02092388 |
| ENSECAG00000019338 | 1.01954397 | 0.00026454 | 0.00098722 |
| ENSECAG00000020946 | 1.01856995 | 5.43E-10 | 8.15E-09 |
| ENSECAG00000036637 | 1.01853807 | 0.00212683 | 0.00613239 |
| ENSECAG00000017241 | 1.01749258 | 0.00187574 | 0.00549111 |
| ENSECAG00000015450 | 1.01722127 | 9.74E-05 | 0.0004117 |
| ENSECAG00000007692 | 1.01703193 | 8.36E-10 | 1.20E-08 |
| ENSECAG00000021447 | 1.01673106 | 8.62E-08 | 7.76E-07 |
| ENSECAG00000000426 | 1.01602096 | 0.01142545 | 0.02621555 |
| ENSECAG00000009244 | 1.01592246 | 0.02019413 | 0.04266408 |
| ENSECAG00000017085 | 1.01559539 | 3.11E-14 | 1.01E-12 |
| ENSECAG00000010965 | 1.0155008 | 3.28E-12 | 7.33E-11 |
| ENSECAG00000044536 | 1.01517077 | 0.01603222 | 0.03503725 |
| ENSECAG00000013196 | 1.01505758 | 0.00704913 | 0.01732937 |
| ENSECAG00000058325 | 1.01444021 | 0.00131969 | 0.00403584 |
| ENSECAG00000024074 | 1.01423655 | 8.76E-07 | 6.18E-06 |
| ENSECAG00000019758 | 1.01372027 | 0.01724194 | 0.03724753 |
| ENSECAG00000009865 | 1.01346286 | 0.0036509 | 0.0098092 |
| ENSECAG00000048099 | 1.01306438 | 5.02E-07 | 3.77E-06 |
| ENSECAG00000058329 | 1.01291646 | 3.94E-07 | 3.03E-06 |
| ENSECAG00000007108 | 1.01200745 | 0.00056931 | 0.00192524 |
| ENSECAG00000016066 | 1.01190196 | 6.64E-05 | 0.00029289 |
| ENSECAG00000037465 | 1.01188638 | 1.90E-08 | 2.02E-07 |
| ENSECAG00000010715 | 1.01178553 | 7.71E-11 | 1.37E-09 |
| ENSECAG00000020558 | 1.01171093 | 3.10E-07 | 2.44E-06 |
| ENSECAG00000057383 | 1.01147877 | 0.00045856 | 0.00159238 |
| ENSECAG00000054001 | 1.0114157 | 4.42E-12 | 9.67E-11 |
| ENSECAG00000020854 | 1.01126483 | 2.19E-10 | 3.54E-09 |
| ENSECAG00000037704 | 1.01110167 | 7.65E-08 | 6.96E-07 |
| ENSECAG00000021010 | 1.01021717 | 0.01426195 | 0.03165135 |
| ENSECAG00000049951 | 1.00910855 | 6.08E-06 | 3.46E-05 |
| ENSECAG00000037770 | 1.00870855 | 0.02235375 | 0.04650996 |
| ENSECAG00000016491 | 1.00870089 | 8.53E-14 | 2.53E-12 |
| ENSECAG00000046956 | 1.00794752 | 0.00030104 | 0.00110405 |
| ENSECAG00000054704 | 1.00789845 | 2.66E-07 | 2.13E-06 |
| ENSECAG00000019546 | 1.0075804 | 1.98E-19 | 1.51E-17 |
| ENSECAG00000022453 | 1.00735188 | 0.02283841 | 0.04735521 |
| ENSECAG00000010982 | 1.00655686 | 0.00506538 | 0.01303292 |
| ENSECAG00000019229 | 1.00636274 | 0.00022286 | 0.00084795 |
| ENSECAG00000018536 | 1.0052505 | 3.09E-06 | 1.90E-05 |
| ENSECAG00000017373 | 1.00482894 | 1.93E-05 | 9.74E-05 |
| ENSECAG00000038834 | 1.00414861 | 0.0020354 | 0.00590219 |
| ENSECAG00000009448 | 1.00345497 | 3.54E-06 | 2.14E-05 |
| ENSECAG00000000836 | 1.00296318 | 4.51E-09 | 5.60E-08 |
| ENSECAG00000019054 | 1.00173614 | 7.81E-12 | 1.65E-10 |
| ENSECAG00000054483 | 1.00167441 | 0.00029097 | 0.00107225 |
| ENSECAG00000000529 | 1.00163325 | 1.86E-06 | 1.21E-05 |
| ENSECAG00000022789 | 1.00109614 | 0.00824666 | 0.01984557 |
| ENSECAG00000010762 | 1.00047053 | 0.01508077 | 0.03321455 |
| ENSECAG00000013845 | 1.00039389 | 6.82E-08 | 6.29E-07 |
| ENSECAG00000013042 | 1.00028747 | 3.25E-11 | 6.19E-10 |
| ENSECAG00000000452 | -1.0000433 | 0.00608396 | 0.01526524 |
| ENSECAG00000055234 | -1.000262 | 0.01372719 | 0.0306472 |
| ENSECAG00000054629 | -1.001659 | 0.00406106 | 0.01074837 |
| ENSECAG00000024028 | -1.0028635 | 0.00435067 | 0.01141349 |
| ENSECAG00000024617 | -1.0029083 | 0.01956584 | 0.04149756 |
| ENSECAG00000021979 | -1.0031871 | 5.84E-07 | 4.30E-06 |
| ENSECAG00000024389 | -1.0037745 | 8.66E-08 | 7.78E-07 |
| ENSECAG00000017826 | -1.0044296 | 0.00481594 | 0.01248313 |
| ENSECAG00000006792 | -1.0047038 | 2.45E-09 | 3.22E-08 |
| ENSECAG00000035469 | -1.0047956 | 8.02E-07 | 5.70E-06 |
| ENSECAG00000017890 | -1.005565 | 0.01493869 | 0.03295686 |
| ENSECAG00000000586 | -1.0059942 | 0.00274649 | 0.00764298 |
| ENSECAG00000017986 | -1.0070543 | 0.00465033 | 0.0121058 |
| ENSECAG00000051819 | -1.0071452 | 5.02E-05 | 0.00022847 |
| ENSECAG00000017410 | -1.0072533 | 0.02366127 | 0.04875378 |
| ENSECAG00000011819 | -1.007475 | 0.02425526 | 0.04981503 |
| ENSECAG00000022078 | -1.0077533 | 3.54E-06 | 2.14E-05 |
| ENSECAG00000009702 | -1.008718 | 1.04E-06 | 7.22E-06 |
| ENSECAG00000001037 | -1.0092388 | 9.15E-05 | 0.00038977 |
| ENSECAG00000046166 | -1.009286 | 0.00024879 | 0.0009342 |
| ENSECAG00000033227 | -1.009696 | 7.94E-11 | 1.41E-09 |
| ENSECAG00000020465 | -1.0100628 | 8.73E-09 | 1.00E-07 |
| ENSECAG00000028231 | -1.010441 | 0.00017396 | 0.00068185 |
| ENSECAG00000042552 | -1.0105967 | 0.00273156 | 0.0076109 |
| ENSECAG00000054729 | -1.010825 | 0.0108672 | 0.02509181 |
| ENSECAG00000016022 | -1.0113465 | 1.75E-26 | 3.65E-24 |
| ENSECAG00000049654 | -1.0119651 | 0.01713459 | 0.03705503 |
| ENSECAG00000023628 | -1.0126605 | 4.64E-06 | 2.72E-05 |
| ENSECAG00000048971 | -1.0134342 | 0.00963562 | 0.02264288 |
| ENSECAG00000005447 | -1.0138961 | 0.00123599 | 0.00380222 |
| ENSECAG00000022067 | -1.014234 | 3.30E-07 | 2.58E-06 |
| ENSECAG00000029829 | -1.0142864 | 0.0012194 | 0.00375949 |
| ENSECAG00000029150 | -1.0144497 | 0.0005007 | 0.0017158 |
| ENSECAG00000024165 | -1.0157602 | 4.02E-12 | 8.86E-11 |
| ENSECAG00000014979 | -1.0158361 | 6.70E-05 | 0.00029497 |
| ENSECAG00000015058 | -1.0165793 | 7.34E-14 | 2.21E-12 |
| ENSECAG00000033821 | -1.0166948 | 9.79E-05 | 0.00041327 |
| ENSECAG00000022121 | -1.0173729 | 0.00171473 | 0.00507312 |
| ENSECAG00000002157 | -1.0174523 | 0.00084736 | 0.00273471 |
| ENSECAG00000017248 | -1.0181437 | 2.44E-08 | 2.52E-07 |
| ENSECAG00000003002 | -1.0198413 | 3.63E-05 | 0.0001708 |
| ENSECAG00000048687 | -1.0220749 | 5.64E-08 | 5.29E-07 |
| ENSECAG00000024029 | -1.0225671 | 1.68E-13 | 4.74E-12 |
| ENSECAG00000037214 | -1.0228805 | 0.00351802 | 0.0094921 |
| ENSECAG00000026898 | -1.0237318 | 1.85E-06 | 1.21E-05 |
| ENSECAG00000057140 | -1.0249036 | 0.0018041 | 0.00530594 |
| ENSECAG00000019429 | -1.0255368 | 2.20E-05 | 0.00010942 |
| ENSECAG00000018415 | -1.0260343 | 1.36E-29 | 4.05E-27 |
| ENSECAG00000019571 | -1.0264356 | 1.26E-07 | 1.09E-06 |
| ENSECAG00000017768 | -1.0265891 | 0.00388258 | 0.01035694 |
| ENSECAG00000015951 | -1.0267815 | 1.99E-08 | 2.11E-07 |
| ENSECAG00000056652 | -1.026804 | 0.00283103 | 0.00784014 |
| ENSECAG00000022980 | -1.0271007 | 8.35E-05 | 0.00035929 |
| ENSECAG00000059350 | -1.0271458 | 0.00083456 | 0.00269962 |
| ENSECAG00000000697 | -1.0275363 | 0.00023888 | 0.00090094 |
| ENSECAG00000017868 | -1.0281502 | 6.43E-28 | 1.63E-25 |
| ENSECAG00000049339 | -1.0281986 | 0.00613123 | 0.01536487 |
| ENSECAG00000019745 | -1.0287666 | 0.0005605 | 0.00189839 |
| ENSECAG00000036067 | -1.0293916 | 0.00808066 | 0.01950637 |
| ENSECAG00000051133 | -1.029858 | 0.0235144 | 0.04851379 |
| ENSECAG00000017912 | -1.0299076 | 8.62E-05 | 0.00036969 |
| ENSECAG00000016183 | -1.0301827 | 9.28E-06 | 5.05E-05 |
| ENSECAG00000008622 | -1.0304124 | 1.78E-06 | 1.17E-05 |
| ENSECAG00000024553 | -1.0312549 | 0.00027412 | 0.00101752 |
| ENSECAG00000013582 | -1.0312932 | 0.00035893 | 0.00128699 |
| ENSECAG00000005626 | -1.0313929 | 0.00176446 | 0.00520099 |
| ENSECAG00000019716 | -1.0315437 | 1.45E-07 | 1.24E-06 |
| ENSECAG00000013390 | -1.032709 | 0.0010496 | 0.00329553 |
| ENSECAG00000012514 | -1.0328641 | 3.86E-06 | 2.31E-05 |
| ENSECAG00000050921 | -1.0331109 | 0.01283295 | 0.02890195 |
| ENSECAG00000043083 | -1.0332756 | 5.80E-05 | 0.00025922 |
| ENSECAG00000009616 | -1.033896 | 6.32E-08 | 5.87E-07 |
| ENSECAG00000031911 | -1.0342084 | 2.48E-06 | 1.56E-05 |
| ENSECAG00000016734 | -1.034669 | 3.23E-18 | 2.00E-16 |
| ENSECAG00000046696 | -1.035391 | 0.01904474 | 0.04057867 |
| ENSECAG00000023588 | -1.0360652 | 0.00026944 | 0.00100264 |
| ENSECAG00000011921 | -1.0363506 | 3.94E-25 | 6.77E-23 |
| ENSECAG00000017812 | -1.0369425 | 0.01481685 | 0.03271712 |
| ENSECAG00000012962 | -1.0370841 | 0.00027023 | 0.00100491 |
| ENSECAG00000010414 | -1.0386169 | 1.36E-10 | 2.30E-09 |
| ENSECAG00000013796 | -1.0408619 | 6.69E-10 | 9.86E-09 |
| ENSECAG00000047495 | -1.0408822 | 1.27E-08 | 1.41E-07 |
| ENSECAG00000022288 | -1.0411143 | 6.49E-11 | 1.17E-09 |
| ENSECAG00000016505 | -1.0418009 | 0.00018583 | 0.0007217 |
| ENSECAG00000023731 | -1.0420879 | 5.72E-07 | 4.23E-06 |
| ENSECAG00000019802 | -1.0437278 | 7.24E-07 | 5.21E-06 |
| ENSECAG00000010098 | -1.0441147 | 4.08E-10 | 6.24E-09 |
| ENSECAG00000018202 | -1.0443767 | 4.36E-13 | 1.15E-11 |
| ENSECAG00000039864 | -1.0446408 | 0.00377338 | 0.010097 |
| ENSECAG00000008944 | -1.0451817 | 4.66E-06 | 2.72E-05 |
| ENSECAG00000026951 | -1.0452037 | 8.63E-08 | 7.76E-07 |
| ENSECAG00000019152 | -1.0473483 | 4.01E-07 | 3.07E-06 |
| ENSECAG00000045840 | -1.0474341 | 4.26E-06 | 2.52E-05 |
| ENSECAG00000025095 | -1.0474766 | 5.28E-08 | 5.01E-07 |
| ENSECAG00000018603 | -1.0479511 | 0.00486338 | 0.0125944 |
| ENSECAG00000014947 | -1.0483917 | 0.00202775 | 0.00588391 |
| ENSECAG00000024041 | -1.050217 | 6.43E-07 | 4.69E-06 |
| ENSECAG00000010523 | -1.0505368 | 0.00096371 | 0.00306144 |
| ENSECAG00000024650 | -1.0520326 | 0.00836618 | 0.02009985 |
| ENSECAG00000006717 | -1.0524311 | 5.74E-07 | 4.24E-06 |
| ENSECAG00000004810 | -1.052649 | 0.00013098 | 0.00053148 |
| ENSECAG00000022861 | -1.0528963 | 2.17E-06 | 1.39E-05 |
| ENSECAG00000052626 | -1.0529557 | 0.00094662 | 0.00301155 |
| ENSECAG00000016742 | -1.0532763 | 8.97E-19 | 6.09E-17 |
| ENSECAG00000059205 | -1.0533042 | 0.02159435 | 0.04515274 |
| ENSECAG00000024448 | -1.0553415 | 0.00523103 | 0.0134005 |
| ENSECAG00000001652 | -1.0559003 | 0.0016127 | 0.00480051 |
| ENSECAG00000010989 | -1.0561187 | 3.72E-06 | 2.24E-05 |
| ENSECAG00000008799 | -1.0565515 | 0.00085313 | 0.00274935 |
| ENSECAG00000008558 | -1.0567557 | 6.75E-09 | 8.00E-08 |
| ENSECAG00000018199 | -1.0577166 | 1.70E-06 | 1.12E-05 |
| ENSECAG00000028489 | -1.0598291 | 0.0062402 | 0.01560993 |
| ENSECAG00000032246 | -1.0606005 | 0.00632058 | 0.01579019 |
| ENSECAG00000019151 | -1.0606499 | 1.01E-14 | 3.54E-13 |
| ENSECAG00000033624 | -1.0609987 | 0.00647091 | 0.01609874 |
| ENSECAG00000047048 | -1.0612248 | 2.75E-05 | 0.00013345 |
| ENSECAG00000026998 | -1.0613409 | 0.00147437 | 0.00444378 |
| ENSECAG00000058972 | -1.0614198 | 0.00988361 | 0.02313873 |
| ENSECAG00000024247 | -1.0614645 | 0.00010789 | 0.00045032 |
| ENSECAG00000009258 | -1.0621902 | 4.10E-09 | 5.12E-08 |
| ENSECAG00000048122 | -1.062426 | 0.0015458 | 0.00462727 |
| ENSECAG00000027122 | -1.0626526 | 0.00347922 | 0.00939877 |
| ENSECAG00000026972 | -1.0627789 | 2.72E-08 | 2.77E-07 |
| ENSECAG00000047648 | -1.0632973 | 0.00718675 | 0.01761298 |
| ENSECAG00000006890 | -1.0651328 | 7.06E-07 | 5.09E-06 |
| ENSECAG00000059172 | -1.06562 | 0.02031535 | 0.04289584 |
| ENSECAG00000012244 | -1.0660581 | 0.01821952 | 0.03908373 |
| ENSECAG00000017983 | -1.066466 | 0.00112459 | 0.00349564 |
| ENSECAG00000000925 | -1.0682695 | 2.69E-06 | 1.69E-05 |
| ENSECAG00000005833 | -1.0684039 | 1.28E-11 | 2.61E-10 |
| ENSECAG00000057905 | -1.0684264 | 0.00032179 | 0.00117171 |
| ENSECAG00000005990 | -1.0685742 | 1.08E-07 | 9.51E-07 |
| ENSECAG00000022294 | -1.0688759 | 0.00035387 | 0.00127152 |
| ENSECAG00000059507 | -1.0689413 | 3.95E-08 | 3.86E-07 |
| ENSECAG00000052684 | -1.0692116 | 3.40E-06 | 2.07E-05 |
| ENSECAG00000048409 | -1.0703107 | 0.01537354 | 0.03380273 |
| ENSECAG00000015593 | -1.0703985 | 6.94E-08 | 6.38E-07 |
| ENSECAG00000034809 | -1.0746573 | 2.17E-05 | 0.00010793 |
| ENSECAG00000031182 | -1.0748463 | 0.00200948 | 0.00584078 |
| ENSECAG00000055561 | -1.075098 | 0.02291499 | 0.04748315 |
| ENSECAG00000023155 | -1.0752594 | 7.11E-09 | 8.39E-08 |
| ENSECAG00000014379 | -1.0765935 | 0.01470788 | 0.0325215 |
| ENSECAG00000005102 | -1.077858 | 3.95E-05 | 0.00018462 |
| ENSECAG00000010269 | -1.0791525 | 5.18E-05 | 0.00023449 |
| ENSECAG00000045965 | -1.0802468 | 0.00406668 | 0.01075815 |
| ENSECAG00000001441 | -1.0803738 | 1.96E-07 | 1.63E-06 |
| ENSECAG00000054413 | -1.0813387 | 0.00559375 | 0.01419948 |
| ENSECAG00000059399 | -1.081778 | 0.01848004 | 0.03956644 |
| ENSECAG00000049227 | -1.0824093 | 0.00263014 | 0.00737336 |
| ENSECAG00000020855 | -1.0845299 | 1.28E-08 | 1.41E-07 |
| ENSECAG00000025708 | -1.0853089 | 0.00228901 | 0.0065418 |
| ENSECAG00000014914 | -1.0865147 | 0.0217806 | 0.0454656 |
| ENSECAG00000059493 | -1.0886986 | 1.47E-05 | 7.62E-05 |
| ENSECAG00000024623 | -1.089068 | 1.20E-08 | 1.33E-07 |
| ENSECAG00000009165 | -1.0891322 | 0.00037594 | 0.00133776 |
| ENSECAG00000022311 | -1.0894284 | 1.75E-14 | 5.84E-13 |
| ENSECAG00000056248 | -1.0896186 | 0.00833356 | 0.0200303 |
| ENSECAG00000025415 | -1.0903045 | 7.50E-05 | 0.00032733 |
| ENSECAG00000017412 | -1.0928912 | 0.00016241 | 0.000642 |
| ENSECAG00000036031 | -1.0936785 | 0.00026071 | 0.00097487 |
| ENSECAG00000000046 | -1.0949863 | 0.00019665 | 0.00075946 |
| ENSECAG00000055840 | -1.0959559 | 9.21E-05 | 0.00039199 |
| ENSECAG00000007374 | -1.0960421 | 6.09E-11 | 1.10E-09 |
| ENSECAG00000053119 | -1.0967723 | 0.00257086 | 0.00722986 |
| ENSECAG00000023915 | -1.0969443 | 2.10E-14 | 6.93E-13 |
| ENSECAG00000047530 | -1.0971464 | 0.00013292 | 0.0005383 |
| ENSECAG00000052677 | -1.0977265 | 0.0001048 | 0.00043955 |
| ENSECAG00000054414 | -1.0988027 | 0.01314805 | 0.0295342 |
| ENSECAG00000009429 | -1.0991777 | 0.00015878 | 0.00062953 |
| ENSECAG00000025824 | -1.0996983 | 0.00304855 | 0.00836172 |
| ENSECAG00000025992 | -1.1000143 | 0.01999429 | 0.04230587 |
| ENSECAG00000022073 | -1.1001258 | 4.26E-06 | 2.52E-05 |
| ENSECAG00000011290 | -1.1003428 | 2.71E-06 | 1.69E-05 |
| ENSECAG00000049824 | -1.1003805 | 0.00285966 | 0.0079116 |
| ENSECAG00000027667 | -1.1005121 | 0.00397499 | 0.01056186 |
| ENSECAG00000010700 | -1.1010743 | 0.00228564 | 0.00653382 |
| ENSECAG00000013939 | -1.1018426 | 4.72E-05 | 0.0002162 |
| ENSECAG00000013382 | -1.1027904 | 3.94E-06 | 2.35E-05 |
| ENSECAG00000010773 | -1.1032928 | 0.00923168 | 0.02181669 |
| ENSECAG00000046584 | -1.1042417 | 0.01158859 | 0.02652399 |
| ENSECAG00000015163 | -1.1051884 | 0.00105112 | 0.00329985 |
| ENSECAG00000002225 | -1.1054197 | 1.19E-05 | 6.31E-05 |
| ENSECAG00000005896 | -1.105667 | 4.00E-06 | 2.39E-05 |
| ENSECAG00000019343 | -1.1065501 | 0.02337082 | 0.04826211 |
| ENSECAG00000012249 | -1.1066272 | 2.16E-08 | 2.27E-07 |
| ENSECAG00000012473 | -1.1070496 | 1.09E-07 | 9.54E-07 |
| ENSECAG00000036209 | -1.107653 | 0.00813193 | 0.01961107 |
| ENSECAG00000024396 | -1.1091726 | 7.48E-14 | 2.25E-12 |
| ENSECAG00000055158 | -1.1096706 | 0.00023039 | 0.00087288 |
| ENSECAG00000007328 | -1.1110719 | 7.46E-07 | 5.35E-06 |
| ENSECAG00000007348 | -1.1138533 | 2.16E-07 | 1.77E-06 |
| ENSECAG00000014298 | -1.1141525 | 9.50E-08 | 8.44E-07 |
| ENSECAG00000008652 | -1.1144685 | 9.23E-05 | 0.00039268 |
| ENSECAG00000057989 | -1.1145971 | 0.01602723 | 0.03502979 |
| ENSECAG00000011475 | -1.1149177 | 2.21E-08 | 2.31E-07 |
| ENSECAG00000031137 | -1.116337 | 4.34E-05 | 0.00020071 |
| ENSECAG00000051370 | -1.117243 | 4.37E-05 | 0.00020198 |
| ENSECAG00000047929 | -1.1182307 | 0.0056945 | 0.01441264 |
| ENSECAG00000012325 | -1.1187606 | 1.78E-08 | 1.90E-07 |
| ENSECAG00000053429 | -1.1199821 | 0.00536392 | 0.01370322 |
| ENSECAG00000014009 | -1.1209765 | 3.41E-13 | 9.19E-12 |
| ENSECAG00000023134 | -1.1223919 | 0.0006877 | 0.0022726 |
| ENSECAG00000013055 | -1.1229073 | 6.18E-08 | 5.75E-07 |
| ENSECAG00000020928 | -1.1249152 | 0.00043962 | 0.00153449 |
| ENSECAG00000021133 | -1.1254767 | 5.38E-14 | 1.67E-12 |
| ENSECAG00000028501 | -1.1257755 | 2.16E-11 | 4.21E-10 |
| ENSECAG00000015867 | -1.1261054 | 2.38E-06 | 1.51E-05 |
| ENSECAG00000024451 | -1.1269048 | 0.00096381 | 0.00306144 |
| ENSECAG00000016515 | -1.1271985 | 6.17E-13 | 1.59E-11 |
| ENSECAG00000008025 | -1.1297981 | 9.58E-06 | 5.20E-05 |
| ENSECAG00000008927 | -1.1304063 | 3.40E-11 | 6.44E-10 |
| ENSECAG00000010522 | -1.1312616 | 3.06E-07 | 2.41E-06 |
| ENSECAG00000024026 | -1.1312712 | 1.25E-23 | 1.68E-21 |
| ENSECAG00000024038 | -1.1324126 | 5.25E-11 | 9.61E-10 |
| ENSECAG00000016389 | -1.1325137 | 2.59E-08 | 2.66E-07 |
| ENSECAG00000009284 | -1.1328999 | 0.00572005 | 0.01446584 |
| ENSECAG00000009189 | -1.134076 | 5.84E-09 | 7.04E-08 |
| ENSECAG00000014501 | -1.1353406 | 0.00027943 | 0.00103414 |
| ENSECAG00000011074 | -1.1355909 | 5.47E-06 | 3.15E-05 |
| ENSECAG00000052735 | -1.1360535 | 8.50E-05 | 0.00036497 |
| ENSECAG00000025680 | -1.136172 | 0.00047529 | 0.00164131 |
| ENSECAG00000013708 | -1.136661 | 0.02415786 | 0.04964882 |
| ENSECAG00000031669 | -1.1369304 | 1.73E-12 | 4.10E-11 |
| ENSECAG00000049573 | -1.1369844 | 0.00049436 | 0.00169692 |
| ENSECAG00000011822 | -1.1374573 | 4.87E-08 | 4.65E-07 |
| ENSECAG00000027594 | -1.1389459 | 0.00153098 | 0.00459149 |
| ENSECAG00000008891 | -1.1390507 | 5.90E-19 | 4.18E-17 |
| ENSECAG00000023969 | -1.1390553 | 7.80E-07 | 5.56E-06 |
| ENSECAG00000054918 | -1.1404224 | 7.89E-05 | 0.00034186 |
| ENSECAG00000012700 | -1.1408037 | 2.35E-09 | 3.11E-08 |
| ENSECAG00000038153 | -1.1410485 | 1.02E-09 | 1.45E-08 |
| ENSECAG00000008919 | -1.1440994 | 5.94E-10 | 8.87E-09 |
| ENSECAG00000021201 | -1.1452333 | 0.00047158 | 0.00163126 |
| ENSECAG00000022090 | -1.1462843 | 9.89E-09 | 1.12E-07 |
| ENSECAG00000006866 | -1.1469698 | 7.85E-05 | 0.0003405 |
| ENSECAG00000047002 | -1.1472407 | 1.41E-05 | 7.35E-05 |
| ENSECAG00000032045 | -1.1475102 | 0.00640525 | 0.01596204 |
| ENSECAG00000055331 | -1.1487636 | 1.88E-10 | 3.09E-09 |
| ENSECAG00000016510 | -1.1489921 | 3.68E-05 | 0.00017293 |
| ENSECAG00000058285 | -1.1498208 | 0.00176301 | 0.00519876 |
| ENSECAG00000012953 | -1.1500528 | 2.43E-09 | 3.20E-08 |
| ENSECAG00000009911 | -1.1506349 | 1.66E-05 | 8.50E-05 |
| ENSECAG00000011171 | -1.1525373 | 0.00153727 | 0.00460789 |
| ENSECAG00000017728 | -1.1528215 | 7.10E-11 | 1.27E-09 |
| ENSECAG00000050248 | -1.1529687 | 0.01045926 | 0.02430055 |
| ENSECAG00000013884 | -1.1530899 | 0.00399817 | 0.0106083 |
| ENSECAG00000028346 | -1.1537967 | 0.00550257 | 0.01400401 |
| ENSECAG00000006870 | -1.1543752 | 0.00345261 | 0.00933706 |
| ENSECAG00000016896 | -1.1544103 | 0.01359612 | 0.03037885 |
| ENSECAG00000021250 | -1.155264 | 1.18E-05 | 6.27E-05 |
| ENSECAG00000021594 | -1.1553521 | 0.0055896 | 0.01419216 |
| ENSECAG00000019434 | -1.1554928 | 0.01169427 | 0.02672805 |
| ENSECAG00000023943 | -1.1561455 | 8.43E-05 | 0.00036237 |
| ENSECAG00000054519 | -1.1563491 | 9.69E-06 | 5.26E-05 |
| ENSECAG00000056127 | -1.157293 | 7.63E-07 | 5.45E-06 |
| ENSECAG00000019779 | -1.158247 | 0.00742213 | 0.01810445 |
| ENSECAG00000009835 | -1.1585581 | 0.00683568 | 0.01685984 |
| ENSECAG00000054094 | -1.1589541 | 0.00056235 | 0.00190342 |
| ENSECAG00000008135 | -1.1591927 | 0.00624538 | 0.0156194 |
| ENSECAG00000053743 | -1.1602239 | 0.00634765 | 0.01584325 |
| ENSECAG00000040014 | -1.1611474 | 0.00201309 | 0.00585049 |
| ENSECAG00000022735 | -1.1618044 | 7.19E-24 | 1.04E-21 |
| ENSECAG00000026130 | -1.162599 | 8.65E-05 | 0.00037072 |
| ENSECAG00000022521 | -1.1632836 | 6.36E-09 | 7.59E-08 |
| ENSECAG00000055039 | -1.1635473 | 0.00792641 | 0.01917129 |
| ENSECAG00000054401 | -1.1636162 | 0.00021463 | 0.00082014 |
| ENSECAG00000014769 | -1.1644521 | 5.15E-11 | 9.46E-10 |
| ENSECAG00000023234 | -1.165337 | 2.51E-12 | 5.72E-11 |
| ENSECAG00000023741 | -1.1654188 | 0.0119473 | 0.02719373 |
| ENSECAG00000014210 | -1.1654189 | 6.58E-09 | 7.84E-08 |
| ENSECAG00000036823 | -1.1654372 | 0.00320188 | 0.00873221 |
| ENSECAG00000003772 | -1.1673013 | 6.87E-13 | 1.75E-11 |
| ENSECAG00000000722 | -1.1678958 | 5.71E-18 | 3.40E-16 |
| ENSECAG00000042569 | -1.1680451 | 1.87E-14 | 6.23E-13 |
| ENSECAG00000006878 | -1.1684493 | 1.98E-10 | 3.23E-09 |
| ENSECAG00000017971 | -1.1699764 | 0.00299455 | 0.00823484 |
| ENSECAG00000018514 | -1.1703015 | 9.28E-06 | 5.06E-05 |
| ENSECAG00000008355 | -1.1713066 | 8.21E-05 | 0.00035397 |
| ENSECAG00000025414 | -1.1716298 | 0.00140086 | 0.00425322 |
| ENSECAG00000055422 | -1.1721461 | 0.00117083 | 0.00362575 |
| ENSECAG00000010447 | -1.1729298 | 0.00051662 | 0.00176683 |
| ENSECAG00000037919 | -1.1760932 | 0.00707073 | 0.01736674 |
| ENSECAG00000010791 | -1.1761113 | 3.80E-08 | 3.73E-07 |
| ENSECAG00000007165 | -1.1767902 | 1.83E-08 | 1.95E-07 |
| ENSECAG00000026831 | -1.1772628 | 0.00409713 | 0.01083101 |
| ENSECAG00000019065 | -1.1787717 | 0.00068028 | 0.00225209 |
| ENSECAG00000058751 | -1.1796969 | 0.01565041 | 0.03432706 |
| ENSECAG00000017108 | -1.1800201 | 1.58E-05 | 8.11E-05 |
| ENSECAG00000009949 | -1.1800362 | 1.17E-06 | 8.03E-06 |
| ENSECAG00000026073 | -1.1811732 | 0.00157202 | 0.00469506 |
| ENSECAG00000015249 | -1.1816049 | 0.00035475 | 0.00127384 |
| ENSECAG00000059457 | -1.182112 | 5.28E-10 | 7.93E-09 |
| ENSECAG00000020220 | -1.1822205 | 0.01392814 | 0.03103383 |
| ENSECAG00000018414 | -1.1824081 | 0.02059976 | 0.04340207 |
| ENSECAG00000004437 | -1.1830041 | 2.14E-09 | 2.85E-08 |
| ENSECAG00000009843 | -1.1835578 | 0.02220484 | 0.04624317 |
| ENSECAG00000050893 | -1.1840264 | 0.00264697 | 0.00740938 |
| ENSECAG00000051131 | -1.1856493 | 0.00074277 | 0.00243085 |
| ENSECAG00000019737 | -1.1881709 | 1.60E-08 | 1.73E-07 |
| ENSECAG00000051906 | -1.1882041 | 6.84E-06 | 3.85E-05 |
| ENSECAG00000041923 | -1.1883874 | 0.00029104 | 0.0010723 |
| ENSECAG00000009634 | -1.188484 | 4.77E-16 | 2.02E-14 |
| ENSECAG00000021951 | -1.1900922 | 9.86E-08 | 8.75E-07 |
| ENSECAG00000000105 | -1.1901829 | 2.31E-09 | 3.06E-08 |
| ENSECAG00000007962 | -1.1911799 | 4.75E-05 | 0.00021742 |
| ENSECAG00000025060 | -1.1915839 | 0.02390344 | 0.04918926 |
| ENSECAG00000045580 | -1.1923489 | 5.23E-05 | 0.00023669 |
| ENSECAG00000053259 | -1.1923917 | 0.00696494 | 0.01714836 |
| ENSECAG00000019805 | -1.1947668 | 0.00489 | 0.01263844 |
| ENSECAG00000024242 | -1.1959534 | 1.82E-07 | 1.52E-06 |
| ENSECAG00000049520 | -1.1964572 | 2.93E-05 | 0.0001409 |
| ENSECAG00000011716 | -1.1966032 | 3.83E-08 | 3.75E-07 |
| ENSECAG00000025891 | -1.1966805 | 0.00261957 | 0.00734834 |
| ENSECAG00000048555 | -1.1970166 | 1.52E-05 | 7.83E-05 |
| ENSECAG00000022677 | -1.1970977 | 9.71E-05 | 0.00041044 |
| ENSECAG00000017783 | -1.1973144 | 0.00165895 | 0.00492505 |
| ENSECAG00000050681 | -1.1979874 | 0.01210824 | 0.02748864 |
| ENSECAG00000009842 | -1.1981985 | 0.0159712 | 0.03492439 |
| ENSECAG00000023711 | -1.1995934 | 0.00023773 | 0.00089719 |
| ENSECAG00000050547 | -1.1998783 | 0.00881554 | 0.02098339 |
| ENSECAG00000052313 | -1.2002894 | 0.00096779 | 0.00307192 |
| ENSECAG00000044989 | -1.2004711 | 0.00080174 | 0.00260176 |
| ENSECAG00000020866 | -1.200685 | 1.17E-06 | 7.99E-06 |
| ENSECAG00000018280 | -1.2010106 | 5.92E-11 | 1.07E-09 |
| ENSECAG00000005628 | -1.2011332 | 4.35E-05 | 0.00020123 |
| ENSECAG00000023202 | -1.2011894 | 2.34E-08 | 2.43E-07 |
| ENSECAG00000027808 | -1.2023735 | 0.00102689 | 0.00323841 |
| ENSECAG00000011629 | -1.2032694 | 0.00904874 | 0.02146075 |
| ENSECAG00000047955 | -1.2032867 | 2.22E-05 | 0.00010978 |
| ENSECAG00000024763 | -1.2046644 | 0.00231519 | 0.00660563 |
| ENSECAG00000017925 | -1.2053355 | 8.67E-05 | 0.0003712 |
| ENSECAG00000020409 | -1.2064805 | 1.44E-16 | 6.71E-15 |
| ENSECAG00000022668 | -1.2069145 | 0.00164731 | 0.0048931 |
| ENSECAG00000049642 | -1.2076966 | 0.0205283 | 0.04328796 |
| ENSECAG00000036575 | -1.2086783 | 4.13E-07 | 3.15E-06 |
| ENSECAG00000011887 | -1.2087851 | 1.84E-05 | 9.30E-05 |
| ENSECAG00000045022 | -1.209747 | 5.87E-07 | 4.32E-06 |
| ENSECAG00000016283 | -1.2108446 | 0.00012064 | 0.00049555 |
| ENSECAG00000018150 | -1.2110701 | 1.14E-11 | 2.35E-10 |
| ENSECAG00000012954 | -1.2112613 | 1.97E-18 | 1.25E-16 |
| ENSECAG00000053265 | -1.2129971 | 0.00010503 | 0.00044018 |
| ENSECAG00000024798 | -1.2141917 | 1.02E-06 | 7.12E-06 |
| ENSECAG00000008411 | -1.2142088 | 1.15E-10 | 1.99E-09 |
| ENSECAG00000015358 | -1.214411 | 1.17E-08 | 1.31E-07 |
| ENSECAG00000006004 | -1.2149626 | 0.00027787 | 0.00102921 |
| ENSECAG00000046765 | -1.2171038 | 0.00720665 | 0.01765014 |
| ENSECAG00000005794 | -1.2191098 | 0.0058599 | 0.01478273 |
| ENSECAG00000047040 | -1.2192466 | 0.01537536 | 0.03380339 |
| ENSECAG00000032515 | -1.220296 | 1.06E-05 | 5.70E-05 |
| ENSECAG00000051749 | -1.2227425 | 0.00216761 | 0.00623549 |
| ENSECAG00000019598 | -1.2227656 | 1.68E-12 | 3.99E-11 |
| ENSECAG00000012484 | -1.2228857 | 2.61E-12 | 5.94E-11 |
| ENSECAG00000044077 | -1.2239123 | 0.00437129 | 0.01145952 |
| ENSECAG00000011386 | -1.2243134 | 0.00089416 | 0.00286588 |
| ENSECAG00000017755 | -1.2257402 | 1.29E-09 | 1.79E-08 |
| ENSECAG00000023942 | -1.2266295 | 5.26E-10 | 7.91E-09 |
| ENSECAG00000000292 | -1.227451 | 3.69E-06 | 2.22E-05 |
| ENSECAG00000012993 | -1.2276902 | 3.42E-08 | 3.39E-07 |
| ENSECAG00000010123 | -1.2282701 | 0.00178039 | 0.00524308 |
| ENSECAG00000009248 | -1.228536 | 1.28E-05 | 6.73E-05 |
| ENSECAG00000051910 | -1.2291835 | 0.02409384 | 0.04954914 |
| ENSECAG00000010554 | -1.2293651 | 2.82E-07 | 2.25E-06 |
| ENSECAG00000009067 | -1.2294444 | 1.62E-05 | 8.28E-05 |
| ENSECAG00000049241 | -1.2308444 | 0.00278872 | 0.0077364 |
| ENSECAG00000027586 | -1.2319097 | 0.0221693 | 0.04618208 |
| ENSECAG00000047808 | -1.2324608 | 4.35E-14 | 1.37E-12 |
| ENSECAG00000060222 | -1.2330593 | 1.33E-06 | 8.97E-06 |
| ENSECAG00000053414 | -1.2337847 | 1.12E-09 | 1.57E-08 |
| ENSECAG00000018023 | -1.2346128 | 3.71E-06 | 2.23E-05 |
| ENSECAG00000021708 | -1.2349618 | 1.42E-07 | 1.22E-06 |
| ENSECAG00000030329 | -1.2372192 | 1.19E-12 | 2.91E-11 |
| ENSECAG00000053635 | -1.2382222 | 0.00179379 | 0.00527838 |
| ENSECAG00000019364 | -1.2384663 | 1.21E-06 | 8.29E-06 |
| ENSECAG00000012470 | -1.2389122 | 0.00289983 | 0.00801381 |
| ENSECAG00000049166 | -1.2396584 | 0.0148347 | 0.03275006 |
| ENSECAG00000022499 | -1.2403866 | 1.59E-10 | 2.65E-09 |
| ENSECAG00000011391 | -1.2427187 | 5.26E-15 | 1.93E-13 |
| ENSECAG00000024113 | -1.2427554 | 6.23E-06 | 3.53E-05 |
| ENSECAG00000025451 | -1.2449901 | 0.0020375 | 0.00590683 |
| ENSECAG00000005870 | -1.2452382 | 3.28E-19 | 2.41E-17 |
| ENSECAG00000044902 | -1.2461798 | 0.00629073 | 0.015724 |
| ENSECAG00000052538 | -1.2469514 | 3.50E-06 | 2.12E-05 |
| ENSECAG00000015063 | -1.2474942 | 5.54E-05 | 0.00024927 |
| ENSECAG00000006632 | -1.2489871 | 0.00123931 | 0.00381192 |
| ENSECAG00000001267 | -1.2495411 | 0.01585736 | 0.03471282 |
| ENSECAG00000024992 | -1.2495756 | 0.00010556 | 0.00044196 |
| ENSECAG00000056212 | -1.2496366 | 0.00240516 | 0.00682053 |
| ENSECAG00000029715 | -1.2500624 | 1.30E-16 | 6.11E-15 |
| ENSECAG00000016662 | -1.2501655 | 6.02E-09 | 7.22E-08 |
| ENSECAG00000014695 | -1.2506624 | 0.00083726 | 0.00270759 |
| ENSECAG00000010065 | -1.2511775 | 0.00302684 | 0.00831237 |
| ENSECAG00000038550 | -1.2517051 | 0.00760992 | 0.01848585 |
| ENSECAG00000009445 | -1.2525132 | 0.01169954 | 0.02673736 |
| ENSECAG00000032623 | -1.2529151 | 0.0001077 | 0.00044961 |
| ENSECAG00000018696 | -1.253095 | 1.42E-12 | 3.43E-11 |
| ENSECAG00000015813 | -1.2534807 | 3.45E-24 | 5.17E-22 |
| ENSECAG00000056109 | -1.2537932 | 8.34E-05 | 0.00035889 |
| ENSECAG00000021651 | -1.254352 | 5.95E-14 | 1.83E-12 |
| ENSECAG00000038251 | -1.2560585 | 0.02192095 | 0.0457244 |
| ENSECAG00000024674 | -1.2567863 | 9.78E-09 | 1.11E-07 |
| ENSECAG00000011955 | -1.2572522 | 0.00031767 | 0.00115898 |
| ENSECAG00000001734 | -1.2575668 | 5.63E-08 | 5.29E-07 |
| ENSECAG00000019533 | -1.2585444 | 0.01449543 | 0.03210893 |
| ENSECAG00000044094 | -1.2595186 | 0.0180812 | 0.03883903 |
| ENSECAG00000043955 | -1.2598544 | 0.00956216 | 0.02249931 |
| ENSECAG00000006494 | -1.259926 | 0.01798152 | 0.0386435 |
| ENSECAG00000009269 | -1.2600808 | 3.60E-07 | 2.80E-06 |
| ENSECAG00000059351 | -1.2605738 | 0.01339648 | 0.0299893 |
| ENSECAG00000016573 | -1.2607428 | 9.24E-05 | 0.00039296 |
| ENSECAG00000021057 | -1.2610186 | 0.00095884 | 0.00304695 |
| ENSECAG00000020278 | -1.2625627 | 4.94E-06 | 2.87E-05 |
| ENSECAG00000013869 | -1.2633799 | 1.72E-06 | 1.13E-05 |
| ENSECAG00000045589 | -1.2646757 | 1.16E-07 | 1.01E-06 |
| ENSECAG00000024612 | -1.2656259 | 1.83E-07 | 1.54E-06 |
| ENSECAG00000000744 | -1.266586 | 2.84E-07 | 2.26E-06 |
| ENSECAG00000054129 | -1.2666427 | 1.28E-05 | 6.73E-05 |
| ENSECAG00000014174 | -1.2668677 | 1.05E-05 | 5.65E-05 |
| ENSECAG00000058838 | -1.2677636 | 0.01496996 | 0.03300629 |
| ENSECAG00000046528 | -1.2691 | 0.00359467 | 0.00967322 |
| ENSECAG00000047071 | -1.2692174 | 0.02238213 | 0.04655165 |
| ENSECAG00000007420 | -1.269586 | 0.00765599 | 0.01858968 |
| ENSECAG00000028593 | -1.2698365 | 0.01055124 | 0.02447861 |
| ENSECAG00000059092 | -1.2703573 | 0.02174722 | 0.04540866 |
| ENSECAG00000053894 | -1.2711319 | 2.22E-05 | 0.00010978 |
| ENSECAG00000009382 | -1.2712225 | 1.37E-14 | 4.65E-13 |
| ENSECAG00000028926 | -1.2712625 | 0.01591824 | 0.03483245 |
| ENSECAG00000047504 | -1.2722323 | 0.00272179 | 0.00758747 |
| ENSECAG00000018716 | -1.2724798 | 2.82E-05 | 0.00013646 |
| ENSECAG00000025017 | -1.2736145 | 1.03E-10 | 1.79E-09 |
| ENSECAG00000038177 | -1.2745458 | 0.01662947 | 0.03610593 |
| ENSECAG00000054657 | -1.2755949 | 9.01E-06 | 4.92E-05 |
| ENSECAG00000011177 | -1.2767962 | 1.24E-07 | 1.08E-06 |
| ENSECAG00000008560 | -1.2773515 | 0.00040077 | 0.00141464 |
| ENSECAG00000009485 | -1.2779321 | 0.00177227 | 0.00522055 |
| ENSECAG00000008363 | -1.2787249 | 0.00011312 | 0.00046943 |
| ENSECAG00000011423 | -1.2795512 | 1.46E-05 | 7.56E-05 |
| ENSECAG00000060384 | -1.2806856 | 0.00217575 | 0.00625488 |
| ENSECAG00000036516 | -1.2809034 | 0.00188132 | 0.00550477 |
| ENSECAG00000055766 | -1.2833033 | 0.02240653 | 0.0465894 |
| ENSECAG00000018638 | -1.2852522 | 0.00088417 | 0.00283834 |
| ENSECAG00000012927 | -1.2861109 | 2.21E-17 | 1.20E-15 |
| ENSECAG00000013660 | -1.2864002 | 0.00025188 | 0.00094501 |
| ENSECAG00000016429 | -1.2866086 | 0.00094425 | 0.0030068 |
| ENSECAG00000007742 | -1.2874372 | 2.17E-10 | 3.52E-09 |
| ENSECAG00000022405 | -1.2880588 | 2.60E-10 | 4.16E-09 |
| ENSECAG00000018301 | -1.2915104 | 2.21E-06 | 1.41E-05 |
| ENSECAG00000046946 | -1.2924091 | 0.00045266 | 0.00157458 |
| ENSECAG00000035548 | -1.2928447 | 3.86E-07 | 2.97E-06 |
| ENSECAG00000000734 | -1.2928925 | 0.00013199 | 0.00053498 |
| ENSECAG00000037450 | -1.2930411 | 6.42E-12 | 1.37E-10 |
| ENSECAG00000032992 | -1.2933612 | 3.56E-06 | 2.15E-05 |
| ENSECAG00000006824 | -1.2937306 | 0.00012703 | 0.00051752 |
| ENSECAG00000021684 | -1.2954534 | 9.72E-12 | 2.02E-10 |
| ENSECAG00000044092 | -1.2962284 | 5.68E-05 | 0.00025476 |
| ENSECAG00000010937 | -1.2983882 | 0.00936014 | 0.02208676 |
| ENSECAG00000008131 | -1.2989234 | 3.70E-09 | 4.66E-08 |
| ENSECAG00000031250 | -1.2994742 | 0.00017372 | 0.00068117 |
| ENSECAG00000009038 | -1.3000449 | 5.31E-07 | 3.97E-06 |
| ENSECAG00000015086 | -1.3000479 | 4.14E-07 | 3.15E-06 |
| ENSECAG00000011016 | -1.300301 | 0.00243981 | 0.0069004 |
| ENSECAG00000012830 | -1.3012118 | 3.57E-08 | 3.53E-07 |
| ENSECAG00000047191 | -1.3016249 | 0.00474368 | 0.01232298 |
| ENSECAG00000051782 | -1.3018166 | 0.00010511 | 0.00044042 |
| ENSECAG00000006986 | -1.3021152 | 0.00012099 | 0.00049664 |
| ENSECAG00000032966 | -1.3022806 | 7.13E-15 | 2.54E-13 |
| ENSECAG00000021108 | -1.3026994 | 3.42E-13 | 9.20E-12 |
| ENSECAG00000037619 | -1.3034164 | 1.46E-06 | 9.79E-06 |
| ENSECAG00000023084 | -1.3037801 | 1.28E-06 | 8.71E-06 |
| ENSECAG00000051212 | -1.3039316 | 0.02007463 | 0.04243569 |
| ENSECAG00000009903 | -1.3044973 | 0.00138293 | 0.00420447 |
| ENSECAG00000018245 | -1.3054658 | 3.78E-08 | 3.71E-07 |
| ENSECAG00000004449 | -1.3061893 | 6.98E-06 | 3.91E-05 |
| ENSECAG00000059303 | -1.3062138 | 0.00905805 | 0.02147826 |
| ENSECAG00000021621 | -1.3063394 | 4.13E-05 | 0.000192 |
| ENSECAG00000017800 | -1.3067518 | 6.91E-17 | 3.41E-15 |
| ENSECAG00000023119 | -1.3080785 | 0.00230174 | 0.0065748 |
| ENSECAG00000017512 | -1.3084408 | 0.00872981 | 0.02082369 |
| ENSECAG00000044999 | -1.308932 | 0.0007987 | 0.00259376 |
| ENSECAG00000024806 | -1.3089437 | 1.87E-05 | 9.47E-05 |
| ENSECAG00000053918 | -1.3093006 | 5.43E-11 | 9.92E-10 |
| ENSECAG00000052116 | -1.309313 | 2.89E-08 | 2.93E-07 |
| ENSECAG00000013998 | -1.3100144 | 1.34E-12 | 3.25E-11 |
| ENSECAG00000019451 | -1.3103515 | 2.37E-09 | 3.13E-08 |
| ENSECAG00000057114 | -1.3114664 | 0.02002595 | 0.04236101 |
| ENSECAG00000002661 | -1.3128317 | 1.96E-07 | 1.62E-06 |
| ENSECAG00000019733 | -1.3129842 | 3.39E-05 | 0.00016085 |
| ENSECAG00000040888 | -1.3131173 | 1.34E-06 | 9.05E-06 |
| ENSECAG00000008528 | -1.3141628 | 0.02170638 | 0.04534455 |
| ENSECAG00000016266 | -1.3142371 | 0.01626735 | 0.03546094 |
| ENSECAG00000051763 | -1.3143875 | 0.00644755 | 0.01605136 |
| ENSECAG00000022150 | -1.314594 | 2.18E-15 | 8.52E-14 |
| ENSECAG00000017450 | -1.3147393 | 0.00013432 | 0.00054286 |
| ENSECAG00000012032 | -1.3151729 | 3.18E-07 | 2.50E-06 |
| ENSECAG00000011747 | -1.318167 | 8.50E-06 | 4.67E-05 |
| ENSECAG00000020534 | -1.3187806 | 1.49E-08 | 1.62E-07 |
| ENSECAG00000021721 | -1.3197927 | 1.84E-10 | 3.03E-09 |
| ENSECAG00000020953 | -1.319896 | 0.00206437 | 0.00597242 |
| ENSECAG00000034483 | -1.3203241 | 0.00786976 | 0.01904457 |
| ENSECAG00000058586 | -1.3208027 | 0.01147627 | 0.02630514 |
| ENSECAG00000053536 | -1.3210954 | 5.05E-05 | 0.00022942 |
| ENSECAG00000050516 | -1.3218541 | 0.00292984 | 0.00808374 |
| ENSECAG00000010205 | -1.3220271 | 2.41E-08 | 2.50E-07 |
| ENSECAG00000021812 | -1.3224666 | 6.37E-07 | 4.65E-06 |
| ENSECAG00000020592 | -1.3225087 | 0.00065266 | 0.00216867 |
| ENSECAG00000017844 | -1.3226114 | 0.01157984 | 0.02650989 |
| ENSECAG00000052860 | -1.3238822 | 0.02242714 | 0.04662792 |
| ENSECAG00000017351 | -1.3245127 | 1.86E-18 | 1.20E-16 |
| ENSECAG00000025076 | -1.3249744 | 1.63E-07 | 1.38E-06 |
| ENSECAG00000031204 | -1.3250302 | 2.39E-05 | 0.0001174 |
| ENSECAG00000020912 | -1.3252373 | 0.00034619 | 0.0012482 |
| ENSECAG00000010537 | -1.3252795 | 0.01630173 | 0.03552896 |
| ENSECAG00000008982 | -1.3263465 | 0.00040563 | 0.00142973 |
| ENSECAG00000060013 | -1.3269714 | 0.01066787 | 0.02470046 |
| ENSECAG00000024977 | -1.3275663 | 1.61E-13 | 4.56E-12 |
| ENSECAG00000047249 | -1.328636 | 0.00271331 | 0.00756815 |
| ENSECAG00000045941 | -1.3287495 | 1.30E-08 | 1.43E-07 |
| ENSECAG00000017667 | -1.3297322 | 3.60E-08 | 3.56E-07 |
| ENSECAG00000008518 | -1.3302253 | 3.47E-05 | 0.00016385 |
| ENSECAG00000047052 | -1.3308497 | 0.00899061 | 0.02134554 |
| ENSECAG00000020233 | -1.3309544 | 0.00010292 | 0.00043214 |
| ENSECAG00000023787 | -1.3312249 | 0.0001351 | 0.00054562 |
| ENSECAG00000022738 | -1.3314604 | 0.0083308 | 0.02002583 |
| ENSECAG00000015857 | -1.3323769 | 4.51E-05 | 0.00020766 |
| ENSECAG00000019442 | -1.3330051 | 0.00754303 | 0.01834329 |
| ENSECAG00000059049 | -1.3337218 | 0.00865344 | 0.02068127 |
| ENSECAG00000010475 | -1.3337753 | 0.0053109 | 0.01358798 |
| ENSECAG00000023039 | -1.3344317 | 0.00225651 | 0.00646212 |
| ENSECAG00000000487 | -1.3347571 | 2.86E-06 | 1.78E-05 |
| ENSECAG00000035051 | -1.3350409 | 0.00737853 | 0.01801186 |
| ENSECAG00000047943 | -1.3365183 | 0.00311832 | 0.00852066 |
| ENSECAG00000051959 | -1.3377856 | 0.00013158 | 0.00053364 |
| ENSECAG00000010667 | -1.3382244 | 7.38E-08 | 6.75E-07 |
| ENSECAG00000020705 | -1.3400293 | 3.27E-06 | 2.00E-05 |
| ENSECAG00000029028 | -1.3405597 | 6.55E-07 | 4.76E-06 |
| ENSECAG00000038309 | -1.3411538 | 0.01930387 | 0.04102455 |
| ENSECAG00000009148 | -1.3418796 | 2.96E-05 | 0.00014237 |
| ENSECAG00000013411 | -1.3422676 | 2.58E-05 | 0.00012611 |
| ENSECAG00000009793 | -1.3430537 | 0.01512213 | 0.03330236 |
| ENSECAG00000011766 | -1.3435024 | 0.00168848 | 0.0050054 |
| ENSECAG00000052023 | -1.3443318 | 4.93E-06 | 2.87E-05 |
| ENSECAG00000007918 | -1.3448278 | 0.01995348 | 0.04223153 |
| ENSECAG00000024779 | -1.3459325 | 2.94E-05 | 0.00014161 |
| ENSECAG00000021976 | -1.3463606 | 1.84E-05 | 9.32E-05 |
| ENSECAG00000023454 | -1.3466403 | 0.00275628 | 0.00766736 |
| ENSECAG00000019756 | -1.3468676 | 0.00018828 | 0.00072919 |
| ENSECAG00000010624 | -1.3471975 | 2.28E-06 | 1.46E-05 |
| ENSECAG00000053349 | -1.3475523 | 0.00020186 | 0.00077759 |
| ENSECAG00000035790 | -1.3480504 | 0.00722556 | 0.01769063 |
| ENSECAG00000024358 | -1.348051 | 0.00588364 | 0.01483422 |
| ENSECAG00000009989 | -1.348829 | 0.01874789 | 0.04003252 |
| ENSECAG00000022063 | -1.3495411 | 4.60E-09 | 5.67E-08 |
| ENSECAG00000011686 | -1.3503809 | 6.81E-09 | 8.06E-08 |
| ENSECAG00000052373 | -1.350707 | 0.00151885 | 0.00456065 |
| ENSECAG00000026825 | -1.3512573 | 4.05E-07 | 3.10E-06 |
| ENSECAG00000010777 | -1.3512731 | 1.14E-05 | 6.07E-05 |
| ENSECAG00000019730 | -1.3525703 | 3.39E-07 | 2.64E-06 |
| ENSECAG00000059933 | -1.3532917 | 9.48E-05 | 0.00040125 |
| ENSECAG00000026988 | -1.3535997 | 0.01145225 | 0.02626356 |
| ENSECAG00000028243 | -1.3546166 | 0.00919253 | 0.02173946 |
| ENSECAG00000010178 | -1.3548844 | 0.00206157 | 0.00596655 |
| ENSECAG00000039211 | -1.3550015 | 0.00058186 | 0.00196164 |
| ENSECAG00000022071 | -1.355875 | 1.20E-08 | 1.33E-07 |
| ENSECAG00000009971 | -1.3558814 | 2.15E-14 | 7.06E-13 |
| ENSECAG00000002905 | -1.3564711 | 8.79E-10 | 1.26E-08 |
| ENSECAG00000024580 | -1.35661 | 5.68E-05 | 0.00025492 |
| ENSECAG00000008710 | -1.3567765 | 3.78E-07 | 2.92E-06 |
| ENSECAG00000054209 | -1.3578168 | 0.00911326 | 0.02158628 |
| ENSECAG00000019755 | -1.3578628 | 0.00149938 | 0.00450884 |
| ENSECAG00000057334 | -1.3595607 | 0.00773379 | 0.01875612 |
| ENSECAG00000033930 | -1.3597216 | 3.84E-05 | 0.0001797 |
| ENSECAG00000052438 | -1.3607385 | 6.62E-05 | 0.00029214 |
| ENSECAG00000031743 | -1.3608184 | 0.00517393 | 0.01327401 |
| ENSECAG00000050571 | -1.3608502 | 2.26E-05 | 0.00011192 |
| ENSECAG00000013732 | -1.3638086 | 0.00635819 | 0.01586425 |
| ENSECAG00000020518 | -1.363868 | 0.00871264 | 0.02078939 |
| ENSECAG00000006583 | -1.3640799 | 4.65E-05 | 0.00021319 |
| ENSECAG00000033478 | -1.3644371 | 0.00099702 | 0.00315394 |
| ENSECAG00000034955 | -1.3660671 | 8.93E-09 | 1.02E-07 |
| ENSECAG00000010505 | -1.3661119 | 2.32E-09 | 3.08E-08 |
| ENSECAG00000037572 | -1.3672082 | 0.00054917 | 0.00186417 |
| ENSECAG00000051511 | -1.3674397 | 0.00154133 | 0.00461771 |
| ENSECAG00000053713 | -1.3678016 | 0.00866388 | 0.02069956 |
| ENSECAG00000022004 | -1.3680964 | 0.00393803 | 0.01048359 |
| ENSECAG00000019372 | -1.3683817 | 6.54E-05 | 0.00028958 |
| ENSECAG00000039281 | -1.3686066 | 0.01117666 | 0.02571068 |
| ENSECAG00000008965 | -1.369245 | 0.0141753 | 0.03148721 |
| ENSECAG00000009715 | -1.3695948 | 0.00573697 | 0.01450175 |
| ENSECAG00000059668 | -1.3699363 | 3.38E-08 | 3.36E-07 |
| ENSECAG00000022551 | -1.3707257 | 1.79E-05 | 9.07E-05 |
| ENSECAG00000033900 | -1.3707785 | 0.01660242 | 0.03605421 |
| ENSECAG00000043168 | -1.371009 | 0.00188743 | 0.00552193 |
| ENSECAG00000009866 | -1.3717726 | 0.02385837 | 0.04911915 |
| ENSECAG00000052772 | -1.3722034 | 0.01085112 | 0.02506763 |
| ENSECAG00000009528 | -1.3738795 | 0.00768668 | 0.01865406 |
| ENSECAG00000056839 | -1.3746005 | 0.00524806 | 0.01344104 |
| ENSECAG00000031387 | -1.3749994 | 2.19E-11 | 4.26E-10 |
| ENSECAG00000055882 | -1.3751193 | 0.01311865 | 0.02948299 |
| ENSECAG00000017405 | -1.3752337 | 7.52E-18 | 4.41E-16 |
| ENSECAG00000050862 | -1.3754989 | 0.00015532 | 0.00061747 |
| ENSECAG00000045415 | -1.3756473 | 8.98E-06 | 4.91E-05 |
| ENSECAG00000029515 | -1.3756536 | 0.00039301 | 0.00139113 |
| ENSECAG00000023519 | -1.3758828 | 0.00516393 | 0.01325742 |
| ENSECAG00000036210 | -1.3761498 | 0.02339039 | 0.04829361 |
| ENSECAG00000049816 | -1.3763487 | 0.00567211 | 0.01436412 |
| ENSECAG00000004400 | -1.3771729 | 5.31E-07 | 3.97E-06 |
| ENSECAG00000016235 | -1.3774227 | 3.58E-10 | 5.55E-09 |
| ENSECAG00000034522 | -1.3778876 | 0.01944174 | 0.04126961 |
| ENSECAG00000037982 | -1.3788865 | 0.00215471 | 0.00620239 |
| ENSECAG00000006176 | -1.378954 | 0.00651017 | 0.0161766 |
| ENSECAG00000009586 | -1.3801195 | 0.00212214 | 0.00612179 |
| ENSECAG00000041215 | -1.3807196 | 0.01074672 | 0.0248547 |
| ENSECAG00000042030 | -1.3820389 | 0.00726989 | 0.01778163 |
| ENSECAG00000044200 | -1.3820802 | 0.00564122 | 0.01430048 |
| ENSECAG00000034852 | -1.3821787 | 0.0002084 | 0.00079852 |
| ENSECAG00000033952 | -1.382542 | 1.31E-19 | 1.03E-17 |
| ENSECAG00000012619 | -1.3833285 | 0.0119403 | 0.02718749 |
| ENSECAG00000024480 | -1.3834261 | 1.08E-10 | 1.88E-09 |
| ENSECAG00000008246 | -1.3836042 | 0.00524336 | 0.01343054 |
| ENSECAG00000024287 | -1.3845219 | 0.00062819 | 0.00210143 |
| ENSECAG00000018135 | -1.3852572 | 1.02E-06 | 7.12E-06 |
| ENSECAG00000031484 | -1.3858134 | 0.01727269 | 0.03729232 |
| ENSECAG00000015842 | -1.3865554 | 7.49E-06 | 4.17E-05 |
| ENSECAG00000023341 | -1.3866218 | 4.42E-09 | 5.50E-08 |
| ENSECAG00000013982 | -1.3871893 | 2.44E-10 | 3.91E-09 |
| ENSECAG00000008971 | -1.3882745 | 0.00086874 | 0.00279523 |
| ENSECAG00000010858 | -1.3885673 | 2.19E-05 | 0.00010889 |
| ENSECAG00000052102 | -1.3886237 | 0.0018451 | 0.00541155 |
| ENSECAG00000051462 | -1.3890173 | 2.49E-05 | 0.00012205 |
| ENSECAG00000049365 | -1.3894045 | 0.00240136 | 0.00681147 |
| ENSECAG00000042718 | -1.3921633 | 0.0002763 | 0.00102478 |
| ENSECAG00000060070 | -1.3925393 | 0.00518229 | 0.01328784 |
| ENSECAG00000057945 | -1.3932564 | 0.01269911 | 0.02862649 |
| ENSECAG00000008843 | -1.3937581 | 0.00042552 | 0.001493 |
| ENSECAG00000020908 | -1.3938268 | 1.25E-18 | 8.28E-17 |
| ENSECAG00000057471 | -1.395875 | 4.76E-11 | 8.81E-10 |
| ENSECAG00000003831 | -1.3960386 | 5.91E-06 | 3.37E-05 |
| ENSECAG00000012075 | -1.3962406 | 5.75E-06 | 3.30E-05 |
| ENSECAG00000025633 | -1.3963057 | 0.00012779 | 0.00052015 |
| ENSECAG00000024340 | -1.3965508 | 0.01076043 | 0.02488127 |
| ENSECAG00000053833 | -1.3973237 | 0.00296954 | 0.00817968 |
| ENSECAG00000054660 | -1.3985399 | 0.02257858 | 0.04688606 |
| ENSECAG00000015894 | -1.3985524 | 1.35E-06 | 9.10E-06 |
| ENSECAG00000054898 | -1.3985979 | 0.00037981 | 0.00135038 |
| ENSECAG00000055020 | -1.398902 | 0.00434909 | 0.01141202 |
| ENSECAG00000010739 | -1.3992309 | 0.00076435 | 0.00249346 |
| ENSECAG00000049886 | -1.3995817 | 0.00011583 | 0.00047842 |
| ENSECAG00000025944 | -1.40062 | 0.00777263 | 0.01883605 |
| ENSECAG00000009086 | -1.4013943 | 1.91E-07 | 1.59E-06 |
| ENSECAG00000022823 | -1.4022508 | 6.53E-06 | 3.70E-05 |
| ENSECAG00000013379 | -1.4047528 | 0.00227391 | 0.00650611 |
| ENSECAG00000003569 | -1.4050969 | 4.20E-13 | 1.12E-11 |
| ENSECAG00000014436 | -1.405369 | 0.00037313 | 0.00132895 |
| ENSECAG00000018973 | -1.4065666 | 0.00182004 | 0.00534787 |
| ENSECAG00000020562 | -1.406878 | 7.64E-05 | 0.00033251 |
| ENSECAG00000008763 | -1.4069938 | 0.01174558 | 0.02682366 |
| ENSECAG00000054882 | -1.4076105 | 0.00601718 | 0.01512144 |
| ENSECAG00000053283 | -1.4076122 | 0.00068055 | 0.00225266 |
| ENSECAG00000009315 | -1.4079834 | 7.28E-09 | 8.56E-08 |
| ENSECAG00000049794 | -1.4083784 | 0.00024675 | 0.00092732 |
| ENSECAG00000053051 | -1.4098514 | 0.01318245 | 0.02959958 |
| ENSECAG00000024778 | -1.4103375 | 3.81E-05 | 0.00017859 |
| ENSECAG00000007686 | -1.4122547 | 4.54E-17 | 2.32E-15 |
| ENSECAG00000017624 | -1.4134874 | 3.72E-25 | 6.45E-23 |
| ENSECAG00000024641 | -1.4136946 | 0.01236134 | 0.02798081 |
| ENSECAG00000017142 | -1.413793 | 0.02359427 | 0.04863366 |
| ENSECAG00000005576 | -1.4144222 | 1.89E-06 | 1.23E-05 |
| ENSECAG00000019282 | -1.4150138 | 0.00015501 | 0.00061646 |
| ENSECAG00000011730 | -1.4153822 | 0.00791719 | 0.01915314 |
| ENSECAG00000011301 | -1.415708 | 6.58E-05 | 0.00029086 |
| ENSECAG00000010160 | -1.4162914 | 0.00291403 | 0.00804607 |
| ENSECAG00000009294 | -1.4162944 | 0.000632 | 0.00211039 |
| ENSECAG00000055655 | -1.4174696 | 0.00898988 | 0.02134554 |
| ENSECAG00000021938 | -1.4187812 | 9.02E-10 | 1.29E-08 |
| ENSECAG00000019085 | -1.4212225 | 8.66E-05 | 0.00037093 |
| ENSECAG00000022844 | -1.4223021 | 2.31E-11 | 4.49E-10 |
| ENSECAG00000052643 | -1.4223819 | 0.00612562 | 0.01535597 |
| ENSECAG00000024468 | -1.4226918 | 0.00554605 | 0.01409761 |
| ENSECAG00000051907 | -1.4228286 | 0.00043491 | 0.00152019 |
| ENSECAG00000013502 | -1.4232409 | 3.68E-09 | 4.63E-08 |
| ENSECAG00000055646 | -1.4232949 | 0.01397983 | 0.03112727 |
| ENSECAG00000002896 | -1.4238338 | 4.67E-07 | 3.54E-06 |
| ENSECAG00000059167 | -1.4249368 | 0.00116396 | 0.00360647 |
| ENSECAG00000000484 | -1.4251272 | 7.14E-19 | 4.96E-17 |
| ENSECAG00000035205 | -1.4254759 | 2.10E-07 | 1.73E-06 |
| ENSECAG00000023972 | -1.4259744 | 5.22E-27 | 1.19E-24 |
| ENSECAG00000058047 | -1.4266391 | 0.00517102 | 0.01326807 |
| ENSECAG00000015079 | -1.4267666 | 1.61E-08 | 1.74E-07 |
| ENSECAG00000021731 | -1.4267871 | 2.27E-10 | 3.66E-09 |
| ENSECAG00000024053 | -1.4272412 | 2.40E-08 | 2.49E-07 |
| ENSECAG00000046660 | -1.4273725 | 9.78E-06 | 5.30E-05 |
| ENSECAG00000046591 | -1.4275932 | 0.00658442 | 0.01633749 |
| ENSECAG00000009136 | -1.427731 | 0.00040118 | 0.00141535 |
| ENSECAG00000059793 | -1.428392 | 3.41E-06 | 2.07E-05 |
| ENSECAG00000012767 | -1.4288665 | 1.24E-07 | 1.08E-06 |
| ENSECAG00000020074 | -1.429263 | 0.00390615 | 0.01040864 |
| ENSECAG00000007805 | -1.4293925 | 7.27E-09 | 8.56E-08 |
| ENSECAG00000023473 | -1.4296139 | 0.00842602 | 0.02021615 |
| ENSECAG00000059998 | -1.4300126 | 1.31E-05 | 6.87E-05 |
| ENSECAG00000016225 | -1.4303554 | 0.00012506 | 0.00051082 |
| ENSECAG00000018836 | -1.4305685 | 0.00506166 | 0.01302635 |
| ENSECAG00000017816 | -1.4306429 | 0.0097436 | 0.02286125 |
| ENSECAG00000026297 | -1.4307665 | 0.01504277 | 0.03314067 |
| ENSECAG00000004556 | -1.4315598 | 2.05E-08 | 2.16E-07 |
| ENSECAG00000015418 | -1.4324605 | 5.77E-08 | 5.40E-07 |
| ENSECAG00000008465 | -1.4332935 | 2.75E-08 | 2.80E-07 |
| ENSECAG00000016664 | -1.4341047 | 1.80E-08 | 1.93E-07 |
| ENSECAG00000016299 | -1.4347779 | 2.88E-23 | 3.66E-21 |
| ENSECAG00000007006 | -1.4353816 | 0.01814072 | 0.03894442 |
| ENSECAG00000008538 | -1.4353955 | 1.39E-17 | 7.86E-16 |
| ENSECAG00000013743 | -1.4372138 | 8.71E-09 | 1.00E-07 |
| ENSECAG00000003923 | -1.4382291 | 0.00039981 | 0.00141161 |
| ENSECAG00000012668 | -1.438752 | 0.00015062 | 0.00060106 |
| ENSECAG00000008427 | -1.4396029 | 6.06E-07 | 4.45E-06 |
| ENSECAG00000008637 | -1.4399627 | 2.31E-06 | 1.47E-05 |
| ENSECAG00000022015 | -1.440051 | 0.01857227 | 0.03971824 |
| ENSECAG00000009306 | -1.4410054 | 8.53E-06 | 4.68E-05 |
| ENSECAG00000046105 | -1.4415845 | 0.00107842 | 0.00337182 |
| ENSECAG00000014709 | -1.4416174 | 3.11E-05 | 0.00014874 |
| ENSECAG00000028509 | -1.4421061 | 8.28E-05 | 0.00035653 |
| ENSECAG00000016372 | -1.4423023 | 2.50E-12 | 5.72E-11 |
| ENSECAG00000021145 | -1.4424361 | 0.00011992 | 0.00049334 |
| ENSECAG00000052341 | -1.4437679 | 0.02400286 | 0.04938022 |
| ENSECAG00000011041 | -1.4438036 | 0.00152835 | 0.00458484 |
| ENSECAG00000008199 | -1.4438125 | 3.43E-05 | 0.00016249 |
| ENSECAG00000057737 | -1.4438746 | 0.01788217 | 0.03845216 |
| ENSECAG00000015059 | -1.4445167 | 0.02203035 | 0.04592688 |
| ENSECAG00000015545 | -1.4445522 | 3.85E-17 | 1.98E-15 |
| ENSECAG00000060278 | -1.4449712 | 5.99E-09 | 7.18E-08 |
| ENSECAG00000001641 | -1.4459205 | 0.01546306 | 0.03396613 |
| ENSECAG00000016070 | -1.4466251 | 6.38E-12 | 1.36E-10 |
| ENSECAG00000055212 | -1.4469735 | 0.01113826 | 0.02563028 |
| ENSECAG00000048111 | -1.4486247 | 0.00010515 | 0.00044049 |
| ENSECAG00000015194 | -1.4502653 | 1.28E-07 | 1.11E-06 |
| ENSECAG00000023333 | -1.4506944 | 0.00590739 | 0.01488237 |
| ENSECAG00000019290 | -1.4510125 | 1.62E-16 | 7.51E-15 |
| ENSECAG00000047925 | -1.4511654 | 7.23E-06 | 4.04E-05 |
| ENSECAG00000018069 | -1.4525609 | 2.34E-05 | 0.00011551 |
| ENSECAG00000043296 | -1.4533003 | 0.005722 | 0.01446915 |
| ENSECAG00000046903 | -1.4538351 | 1.74E-06 | 1.15E-05 |
| ENSECAG00000000395 | -1.4543476 | 4.91E-12 | 1.07E-10 |
| ENSECAG00000014081 | -1.4550164 | 0.01840701 | 0.03944032 |
| ENSECAG00000022747 | -1.4550794 | 7.15E-07 | 5.15E-06 |
| ENSECAG00000024621 | -1.4550878 | 0.00766598 | 0.01860987 |
| ENSECAG00000009453 | -1.4567803 | 1.03E-12 | 2.55E-11 |
| ENSECAG00000019008 | -1.4575792 | 1.95E-06 | 1.26E-05 |
| ENSECAG00000025860 | -1.4580344 | 2.15E-07 | 1.76E-06 |
| ENSECAG00000006110 | -1.4582686 | 2.84E-12 | 6.40E-11 |
| ENSECAG00000032008 | -1.4587274 | 0.00341348 | 0.00925139 |
| ENSECAG00000051914 | -1.4593328 | 4.99E-05 | 0.00022731 |
| ENSECAG00000004022 | -1.4595337 | 1.54E-07 | 1.31E-06 |
| ENSECAG00000049525 | -1.4596896 | 0.01373214 | 0.03065518 |
| ENSECAG00000018579 | -1.4602079 | 3.71E-10 | 5.73E-09 |
| ENSECAG00000009392 | -1.4603573 | 6.68E-06 | 3.77E-05 |
| ENSECAG00000015760 | -1.4604015 | 5.16E-05 | 0.00023388 |
| ENSECAG00000001568 | -1.4604021 | 5.55E-05 | 0.00024966 |
| ENSECAG00000037532 | -1.4612533 | 0.02073959 | 0.04365551 |
| ENSECAG00000043023 | -1.4615827 | 0.00839381 | 0.02015344 |
| ENSECAG00000033053 | -1.4622226 | 0.02160169 | 0.04516387 |
| ENSECAG00000016113 | -1.4629207 | 0.01133191 | 0.02602228 |
| ENSECAG00000026520 | -1.4629322 | 8.19E-09 | 9.50E-08 |
| ENSECAG00000041925 | -1.4635411 | 0.00066963 | 0.00221848 |
| ENSECAG00000050474 | -1.4649768 | 3.05E-07 | 2.41E-06 |
| ENSECAG00000001854 | -1.4649793 | 6.16E-10 | 9.15E-09 |
| ENSECAG00000023650 | -1.4654483 | 5.61E-05 | 0.00025186 |
| ENSECAG00000010720 | -1.4658092 | 4.83E-05 | 0.00022046 |
| ENSECAG00000036352 | -1.467962 | 0.0047463 | 0.01232548 |
| ENSECAG00000057823 | -1.4685115 | 0.00040812 | 0.00143733 |
| ENSECAG00000011749 | -1.4686116 | 0.00010134 | 0.0004263 |
| ENSECAG00000060108 | -1.4686154 | 0.00277967 | 0.00771994 |
| ENSECAG00000058723 | -1.4689002 | 0.00267978 | 0.00748749 |
| ENSECAG00000045770 | -1.4693236 | 1.52E-12 | 3.63E-11 |
| ENSECAG00000007989 | -1.4694594 | 0.00832308 | 0.02001254 |
| ENSECAG00000045725 | -1.4699635 | 0.0053352 | 0.01363921 |
| ENSECAG00000022239 | -1.4700886 | 0.01147498 | 0.02630514 |
| ENSECAG00000017090 | -1.4703138 | 0.00108127 | 0.00337885 |
| ENSECAG00000013336 | -1.4705934 | 0.00369016 | 0.00990158 |
| ENSECAG00000049671 | -1.4708395 | 4.61E-07 | 3.49E-06 |
| ENSECAG00000043950 | -1.4710624 | 0.00061517 | 0.00206083 |
| ENSECAG00000006493 | -1.4718236 | 2.30E-09 | 3.06E-08 |
| ENSECAG00000057585 | -1.4730365 | 0.00868182 | 0.02072912 |
| ENSECAG00000034083 | -1.4736039 | 0.00158275 | 0.00472267 |
| ENSECAG00000049713 | -1.4736371 | 0.00809317 | 0.01953024 |
| ENSECAG00000012373 | -1.4737505 | 9.43E-10 | 1.35E-08 |
| ENSECAG00000034327 | -1.47491 | 0.01673675 | 0.03631414 |
| ENSECAG00000017845 | -1.4754128 | 2.84E-05 | 0.00013705 |
| ENSECAG00000057627 | -1.4755755 | 0.00022583 | 0.00085749 |
| ENSECAG00000044299 | -1.4757199 | 0.005217 | 0.01337069 |
| ENSECAG00000059574 | -1.4757477 | 1.43E-06 | 9.62E-06 |
| ENSECAG00000024098 | -1.4760233 | 6.37E-13 | 1.64E-11 |
| ENSECAG00000014710 | -1.476882 | 1.16E-06 | 7.94E-06 |
| ENSECAG00000021132 | -1.4791459 | 7.74E-06 | 4.29E-05 |
| ENSECAG00000022300 | -1.4792266 | 1.31E-07 | 1.13E-06 |
| ENSECAG00000050467 | -1.4795199 | 0.00106299 | 0.00333008 |
| ENSECAG00000023383 | -1.4803275 | 0.0125037 | 0.02824012 |
| ENSECAG00000054157 | -1.4808008 | 4.48E-05 | 0.00020623 |
| ENSECAG00000057666 | -1.4810296 | 2.46E-05 | 0.00012068 |
| ENSECAG00000022191 | -1.4816689 | 0.0049414 | 0.01276096 |
| ENSECAG00000052012 | -1.4837762 | 3.39E-08 | 3.37E-07 |
| ENSECAG00000051515 | -1.4863114 | 3.08E-08 | 3.09E-07 |
| ENSECAG00000008739 | -1.4875018 | 3.36E-06 | 2.05E-05 |
| ENSECAG00000008094 | -1.4883118 | 1.02E-06 | 7.12E-06 |
| ENSECAG00000019629 | -1.4885344 | 0.00020128 | 0.00077575 |
| ENSECAG00000011528 | -1.4898083 | 3.01E-09 | 3.87E-08 |
| ENSECAG00000022905 | -1.4910838 | 1.02E-21 | 1.07E-19 |
| ENSECAG00000019950 | -1.4915715 | 2.33E-20 | 1.98E-18 |
| ENSECAG00000053074 | -1.4918304 | 0.01276125 | 0.02874625 |
| ENSECAG00000045134 | -1.4932867 | 0.00192644 | 0.00562428 |
| ENSECAG00000015980 | -1.4933909 | 0.01381246 | 0.03080987 |
| ENSECAG00000032529 | -1.493577 | 0.00020418 | 0.00078517 |
| ENSECAG00000014680 | -1.4936164 | 6.89E-07 | 4.98E-06 |
| ENSECAG00000001010 | -1.4943818 | 0.01080211 | 0.02496498 |
| ENSECAG00000011109 | -1.4949981 | 2.34E-13 | 6.47E-12 |
| ENSECAG00000057428 | -1.4970053 | 0.00059902 | 0.00201321 |
| ENSECAG00000059267 | -1.4971858 | 0.00272821 | 0.00760347 |
| ENSECAG00000041924 | -1.4975584 | 0.00011493 | 0.00047541 |
| ENSECAG00000053059 | -1.4976126 | 1.03E-17 | 5.90E-16 |
| ENSECAG00000031323 | -1.4977344 | 3.49E-14 | 1.12E-12 |
| ENSECAG00000057104 | -1.4979179 | 0.00448103 | 0.01170599 |
| ENSECAG00000045464 | -1.4981309 | 0.00057234 | 0.00193457 |
| ENSECAG00000046283 | -1.4993291 | 0.01784715 | 0.03839535 |
| ENSECAG00000049468 | -1.5002215 | 0.00670198 | 0.01658681 |
| ENSECAG00000016987 | -1.5004183 | 5.76E-17 | 2.89E-15 |
| ENSECAG00000015106 | -1.501607 | 5.61E-14 | 1.73E-12 |
| ENSECAG00000055995 | -1.5016153 | 2.12E-06 | 1.36E-05 |
| ENSECAG00000023703 | -1.5016481 | 2.53E-09 | 3.33E-08 |
| ENSECAG00000055986 | -1.5037264 | 0.01917563 | 0.04079386 |
| ENSECAG00000013650 | -1.5039246 | 5.62E-05 | 0.00025259 |
| ENSECAG00000010805 | -1.5042184 | 9.92E-07 | 6.91E-06 |
| ENSECAG00000052899 | -1.5055157 | 3.56E-06 | 2.15E-05 |
| ENSECAG00000001445 | -1.5059163 | 9.30E-11 | 1.63E-09 |
| ENSECAG00000011960 | -1.5068423 | 3.89E-12 | 8.58E-11 |
| ENSECAG00000022003 | -1.5070897 | 3.77E-12 | 8.32E-11 |
| ENSECAG00000049526 | -1.5073447 | 0.00297275 | 0.00818496 |
| ENSECAG00000010603 | -1.5073631 | 2.82E-05 | 0.00013635 |
| ENSECAG00000000852 | -1.5082837 | 5.00E-06 | 2.90E-05 |
| ENSECAG00000012922 | -1.5106598 | 0.01286837 | 0.02895837 |
| ENSECAG00000005070 | -1.51074 | 0.00013836 | 0.00055746 |
| ENSECAG00000010898 | -1.5108553 | 6.25E-06 | 3.54E-05 |
| ENSECAG00000013400 | -1.511906 | 0.00277335 | 0.00770524 |
| ENSECAG00000048323 | -1.5126864 | 0.01896202 | 0.04043567 |
| ENSECAG00000023266 | -1.5132266 | 1.43E-16 | 6.66E-15 |
| ENSECAG00000012621 | -1.5135097 | 0.00291882 | 0.0080583 |
| ENSECAG00000009820 | -1.5137279 | 0.00975117 | 0.02287661 |
| ENSECAG00000002810 | -1.5140167 | 0.00824363 | 0.01984366 |
| ENSECAG00000012196 | -1.515486 | 0.00026751 | 0.00099672 |
| ENSECAG00000009276 | -1.5173987 | 2.17E-09 | 2.90E-08 |
| ENSECAG00000020208 | -1.5174195 | 6.67E-07 | 4.84E-06 |
| ENSECAG00000044106 | -1.5177707 | 0.00496353 | 0.01280775 |
| ENSECAG00000059096 | -1.5189691 | 0.00375084 | 0.01004268 |
| ENSECAG00000019590 | -1.5190012 | 2.05E-11 | 4.01E-10 |
| ENSECAG00000017771 | -1.519046 | 8.20E-11 | 1.45E-09 |
| ENSECAG00000017598 | -1.5205227 | 6.08E-06 | 3.46E-05 |
| ENSECAG00000016431 | -1.5207105 | 9.95E-16 | 4.09E-14 |
| ENSECAG00000043038 | -1.5214541 | 0.00940498 | 0.02218321 |
| ENSECAG00000012099 | -1.5224113 | 0.00066776 | 0.002213 |
| ENSECAG00000012750 | -1.5238877 | 9.07E-11 | 1.59E-09 |
| ENSECAG00000042020 | -1.5239637 | 0.00232868 | 0.00663903 |
| ENSECAG00000020106 | -1.5243809 | 0.00032069 | 0.00116846 |
| ENSECAG00000059780 | -1.5246117 | 0.01238781 | 0.02802937 |
| ENSECAG00000005515 | -1.5248845 | 0.00607025 | 0.01523425 |
| ENSECAG00000055384 | -1.5256807 | 0.01927469 | 0.04097549 |
| ENSECAG00000052513 | -1.5258012 | 5.94E-06 | 3.39E-05 |
| ENSECAG00000014767 | -1.5261736 | 1.12E-06 | 7.71E-06 |
| ENSECAG00000048227 | -1.5267836 | 0.00512539 | 0.01316913 |
| ENSECAG00000023149 | -1.5268909 | 0.00038348 | 0.00136151 |
| ENSECAG00000059110 | -1.5271202 | 0.00286774 | 0.00793199 |
| ENSECAG00000052640 | -1.5277905 | 0.02218354 | 0.04620313 |
| ENSECAG00000057234 | -1.5283622 | 0.00064836 | 0.00215602 |
| ENSECAG00000016140 | -1.5300814 | 0.00302802 | 0.00831377 |
| ENSECAG00000039651 | -1.5300849 | 4.77E-06 | 2.78E-05 |
| ENSECAG00000052939 | -1.5305049 | 0.01864866 | 0.03985492 |
| ENSECAG00000023226 | -1.5307033 | 1.90E-06 | 1.24E-05 |
| ENSECAG00000016907 | -1.531929 | 7.79E-05 | 0.00033845 |
| ENSECAG00000044141 | -1.5332587 | 2.25E-06 | 1.44E-05 |
| ENSECAG00000012109 | -1.5337249 | 0.00086967 | 0.00279783 |
| ENSECAG00000031881 | -1.534302 | 0.01856722 | 0.03971506 |
| ENSECAG00000056171 | -1.5354905 | 0.00070214 | 0.00231143 |
| ENSECAG00000016856 | -1.5378601 | 0.00012217 | 0.00050084 |
| ENSECAG00000010648 | -1.5379703 | 0.00786134 | 0.01902626 |
| ENSECAG00000050208 | -1.5381058 | 0.00152409 | 0.00457455 |
| ENSECAG00000058791 | -1.5381413 | 2.46E-06 | 1.56E-05 |
| ENSECAG00000037888 | -1.5393153 | 0.00488906 | 0.01263748 |
| ENSECAG00000054445 | -1.53968 | 0.00035183 | 0.00126562 |
| ENSECAG00000034963 | -1.5400833 | 0.02089077 | 0.04391582 |
| ENSECAG00000024132 | -1.5409862 | 0.01651423 | 0.0358978 |
| ENSECAG00000015064 | -1.5414335 | 0.0005943 | 0.00199883 |
| ENSECAG00000017359 | -1.5416623 | 5.43E-08 | 5.12E-07 |
| ENSECAG00000008993 | -1.5431 | 6.78E-10 | 9.98E-09 |
| ENSECAG00000030328 | -1.5435761 | 0.01321884 | 0.02966935 |
| ENSECAG00000015094 | -1.5437867 | 0.00815749 | 0.01966846 |
| ENSECAG00000055542 | -1.5450136 | 0.00037817 | 0.001345 |
| ENSECAG00000014880 | -1.5459816 | 0.00044743 | 0.00155883 |
| ENSECAG00000019427 | -1.5460503 | 0.02357356 | 0.04860444 |
| ENSECAG00000031118 | -1.5467195 | 4.20E-06 | 2.49E-05 |
| ENSECAG00000016368 | -1.5477503 | 1.54E-05 | 7.92E-05 |
| ENSECAG00000019954 | -1.5485527 | 0.00062877 | 0.00210273 |
| ENSECAG00000049389 | -1.5490817 | 4.13E-06 | 2.45E-05 |
| ENSECAG00000039596 | -1.5495357 | 0.01551241 | 0.03406785 |
| ENSECAG00000023360 | -1.5495998 | 0.02023711 | 0.04275085 |
| ENSECAG00000007194 | -1.5505881 | 1.42E-05 | 7.41E-05 |
| ENSECAG00000012105 | -1.5508436 | 8.10E-05 | 0.00034956 |
| ENSECAG00000052866 | -1.5513401 | 6.99E-06 | 3.92E-05 |
| ENSECAG00000049936 | -1.5516287 | 3.04E-05 | 0.00014569 |
| ENSECAG00000002139 | -1.5517402 | 1.48E-06 | 9.91E-06 |
| ENSECAG00000052260 | -1.5528601 | 6.30E-06 | 3.57E-05 |
| ENSECAG00000044586 | -1.5531062 | 2.32E-09 | 3.08E-08 |
| ENSECAG00000053377 | -1.5541115 | 0.02331786 | 0.048175 |
| ENSECAG00000017460 | -1.5542944 | 0.01977114 | 0.0418813 |
| ENSECAG00000025789 | -1.5547692 | 0.0111818 | 0.02571806 |
| ENSECAG00000023404 | -1.5554392 | 2.94E-09 | 3.80E-08 |
| ENSECAG00000013683 | -1.55544 | 0.00184962 | 0.00542338 |
| ENSECAG00000046807 | -1.5563141 | 0.00298704 | 0.00821722 |
| ENSECAG00000019851 | -1.5576003 | 0.00382995 | 0.01022753 |
| ENSECAG00000016402 | -1.5577315 | 0.00023215 | 0.00087897 |
| ENSECAG00000058298 | -1.5579489 | 0.00111073 | 0.00346072 |
| ENSECAG00000013680 | -1.5581099 | 5.46E-09 | 6.61E-08 |
| ENSECAG00000007688 | -1.5588298 | 4.14E-07 | 3.16E-06 |
| ENSECAG00000000924 | -1.5590044 | 0.01477254 | 0.03262895 |
| ENSECAG00000019710 | -1.5605154 | 0.02349611 | 0.04848052 |
| ENSECAG00000029603 | -1.5612909 | 0.00125848 | 0.00386504 |
| ENSECAG00000018752 | -1.5615887 | 0.00560212 | 0.01421749 |
| ENSECAG00000012220 | -1.5641776 | 4.00E-45 | 1.02E-41 |
| ENSECAG00000019562 | -1.5643014 | 0.00038262 | 0.00135909 |
| ENSECAG00000008679 | -1.5643606 | 1.41E-10 | 2.38E-09 |
| ENSECAG00000051371 | -1.5685677 | 0.01039399 | 0.02417403 |
| ENSECAG00000049637 | -1.5693106 | 6.38E-05 | 0.00028304 |
| ENSECAG00000032350 | -1.5695152 | 0.02127593 | 0.04459125 |
| ENSECAG00000007355 | -1.5699138 | 8.94E-07 | 6.29E-06 |
| ENSECAG00000033788 | -1.5699216 | 0.00147809 | 0.00445199 |
| ENSECAG00000016829 | -1.5714664 | 8.59E-07 | 6.06E-06 |
| ENSECAG00000023496 | -1.5716819 | 5.81E-08 | 5.44E-07 |
| ENSECAG00000057741 | -1.5718462 | 1.69E-07 | 1.42E-06 |
| ENSECAG00000009280 | -1.5720655 | 3.32E-07 | 2.60E-06 |
| ENSECAG00000019820 | -1.5733733 | 1.01E-05 | 5.46E-05 |
| ENSECAG00000060383 | -1.5734566 | 0.01518774 | 0.03342709 |
| ENSECAG00000025107 | -1.5735371 | 3.50E-13 | 9.38E-12 |
| ENSECAG00000040127 | -1.5735373 | 0.0021874 | 0.00628598 |
| ENSECAG00000024752 | -1.5735382 | 0.00052683 | 0.00179842 |
| ENSECAG00000045417 | -1.5741367 | 0.00159474 | 0.00475401 |
| ENSECAG00000016098 | -1.5760245 | 2.76E-09 | 3.59E-08 |
| ENSECAG00000015417 | -1.5771736 | 0.00292475 | 0.00807269 |
| ENSECAG00000013529 | -1.5777974 | 1.23E-10 | 2.11E-09 |
| ENSECAG00000049866 | -1.5784311 | 0.01042972 | 0.024242 |
| ENSECAG00000055370 | -1.5794853 | 0.00658036 | 0.01632923 |
| ENSECAG00000012695 | -1.5795059 | 0.00635689 | 0.01586278 |
| ENSECAG00000015457 | -1.5799316 | 0.01338531 | 0.02997376 |
| ENSECAG00000011856 | -1.5804915 | 8.09E-05 | 0.00034938 |
| ENSECAG00000021234 | -1.5805623 | 1.73E-07 | 1.46E-06 |
| ENSECAG00000030677 | -1.5807003 | 0.00039641 | 0.00140094 |
| ENSECAG00000039464 | -1.5815545 | 0.00074554 | 0.00243851 |
| ENSECAG00000000758 | -1.581693 | 1.23E-07 | 1.07E-06 |
| ENSECAG00000047339 | -1.5820823 | 4.77E-07 | 3.60E-06 |
| ENSECAG00000026462 | -1.5829148 | 0.02312183 | 0.04783191 |
| ENSECAG00000009948 | -1.5829338 | 0.00082412 | 0.00266891 |
| ENSECAG00000043615 | -1.58493 | 0.00018782 | 0.00072788 |
| ENSECAG00000045104 | -1.5852376 | 0.00052886 | 0.00180425 |
| ENSECAG00000015044 | -1.5860227 | 4.50E-06 | 2.65E-05 |
| ENSECAG00000013482 | -1.5864913 | 0.01541621 | 0.03387989 |
| ENSECAG00000059468 | -1.5869776 | 1.40E-16 | 6.54E-15 |
| ENSECAG00000039487 | -1.5870289 | 1.05E-05 | 5.63E-05 |
| ENSECAG00000007247 | -1.5878162 | 0.00369491 | 0.00991168 |
| ENSECAG00000051878 | -1.5879236 | 0.01484961 | 0.0327765 |
| ENSECAG00000018341 | -1.5880221 | 0.00012658 | 0.00051627 |
| ENSECAG00000009570 | -1.5892493 | 5.33E-09 | 6.46E-08 |
| ENSECAG00000023663 | -1.5892912 | 2.07E-06 | 1.33E-05 |
| ENSECAG00000033137 | -1.5931146 | 0.00033085 | 0.00119921 |
| ENSECAG00000050985 | -1.5932767 | 0.00923684 | 0.02182347 |
| ENSECAG00000017808 | -1.5945933 | 4.36E-19 | 3.16E-17 |
| ENSECAG00000037383 | -1.5947932 | 2.93E-06 | 1.82E-05 |
| ENSECAG00000011994 | -1.5965966 | 2.28E-06 | 1.45E-05 |
| ENSECAG00000055622 | -1.5968495 | 2.31E-05 | 0.0001139 |
| ENSECAG00000007901 | -1.5976952 | 1.45E-06 | 9.71E-06 |
| ENSECAG00000052188 | -1.5988612 | 0.01774498 | 0.03821601 |
| ENSECAG00000020364 | -1.6009646 | 0.0011377 | 0.00353294 |
| ENSECAG00000024526 | -1.6020293 | 1.33E-07 | 1.15E-06 |
| ENSECAG00000022926 | -1.602813 | 0.00526762 | 0.01348805 |
| ENSECAG00000039517 | -1.6050598 | 0.0006148 | 0.00206034 |
| ENSECAG00000023960 | -1.6053031 | 4.99E-05 | 0.00022704 |
| ENSECAG00000029088 | -1.6053532 | 0.00606363 | 0.01522277 |
| ENSECAG00000025093 | -1.6053855 | 0.00557432 | 0.0141598 |
| ENSECAG00000012449 | -1.6054569 | 1.09E-14 | 3.76E-13 |
| ENSECAG00000022125 | -1.6060396 | 0.00676086 | 0.01670479 |
| ENSECAG00000048490 | -1.6078011 | 2.94E-06 | 1.82E-05 |
| ENSECAG00000055827 | -1.6088288 | 0.00018484 | 0.00071858 |
| ENSECAG00000046603 | -1.60888 | 0.00691478 | 0.01703426 |
| ENSECAG00000051894 | -1.6095226 | 0.02086613 | 0.04387227 |
| ENSECAG00000012150 | -1.6097401 | 1.92E-08 | 2.04E-07 |
| ENSECAG00000051273 | -1.6102113 | 1.53E-18 | 9.95E-17 |
| ENSECAG00000021000 | -1.6111278 | 0.0113068 | 0.02597264 |
| ENSECAG00000010346 | -1.6129876 | 0.0093356 | 0.02203118 |
| ENSECAG00000013279 | -1.6132799 | 0.00020635 | 0.00079201 |
| ENSECAG00000018746 | -1.6152312 | 9.76E-10 | 1.39E-08 |
| ENSECAG00000016893 | -1.6159036 | 6.85E-05 | 0.00030128 |
| ENSECAG00000030351 | -1.6166135 | 0.00015356 | 0.00061146 |
| ENSECAG00000007711 | -1.6168172 | 0.01125962 | 0.02587554 |
| ENSECAG00000008614 | -1.6169642 | 0.00021435 | 0.00081918 |
| ENSECAG00000048983 | -1.6174139 | 3.89E-05 | 0.00018187 |
| ENSECAG00000040185 | -1.6184662 | 0.00948245 | 0.02233999 |
| ENSECAG00000022873 | -1.6186785 | 2.67E-12 | 6.07E-11 |
| ENSECAG00000017468 | -1.6192475 | 0.0017129 | 0.00506838 |
| ENSECAG00000000416 | -1.6197549 | 0.00123189 | 0.0037917 |
| ENSECAG00000027354 | -1.6202615 | 0.00821192 | 0.01978124 |
| ENSECAG00000023163 | -1.6206838 | 0.0049623 | 0.01280605 |
| ENSECAG00000034127 | -1.6212426 | 3.08E-07 | 2.43E-06 |
| ENSECAG00000012853 | -1.6214506 | 0.00028938 | 0.00106709 |
| ENSECAG00000011871 | -1.62147 | 0.00046142 | 0.00160131 |
| ENSECAG00000014045 | -1.6215173 | 0.02122605 | 0.04450341 |
| ENSECAG00000011697 | -1.6217366 | 7.06E-10 | 1.03E-08 |
| ENSECAG00000032603 | -1.621919 | 0.00147656 | 0.00444919 |
| ENSECAG00000053040 | -1.6219935 | 0.00197002 | 0.00573803 |
| ENSECAG00000055555 | -1.6220311 | 6.05E-05 | 0.00027004 |
| ENSECAG00000030432 | -1.6225323 | 1.57E-21 | 1.57E-19 |
| ENSECAG00000015220 | -1.6230469 | 0.0212873 | 0.0446109 |
| ENSECAG00000054801 | -1.6236833 | 0.0026944 | 0.00752142 |
| ENSECAG00000013387 | -1.6240899 | 0.02436063 | 0.04999197 |
| ENSECAG00000002117 | -1.6249153 | 0.0193982 | 0.04119283 |
| ENSECAG00000011308 | -1.6249198 | 0.01065656 | 0.02468196 |
| ENSECAG00000053080 | -1.6253523 | 1.48E-12 | 3.55E-11 |
| ENSECAG00000015708 | -1.6257595 | 0.01624201 | 0.03540916 |
| ENSECAG00000052975 | -1.6259318 | 9.89E-07 | 6.89E-06 |
| ENSECAG00000022037 | -1.6260818 | 2.09E-05 | 0.00010449 |
| ENSECAG00000058187 | -1.6261572 | 5.51E-06 | 3.17E-05 |
| ENSECAG00000008059 | -1.6262253 | 5.19E-05 | 0.00023479 |
| ENSECAG00000024109 | -1.6273648 | 0.00029249 | 0.00107642 |
| ENSECAG00000020118 | -1.6281187 | 7.66E-10 | 1.11E-08 |
| ENSECAG00000004092 | -1.6291695 | 0.01345504 | 0.03009071 |
| ENSECAG00000003033 | -1.6292839 | 0.00220777 | 0.00634044 |
| ENSECAG00000009815 | -1.6294778 | 8.44E-05 | 0.0003627 |
| ENSECAG00000000751 | -1.6301468 | 1.99E-16 | 9.11E-15 |
| ENSECAG00000048408 | -1.6308208 | 0.00747209 | 0.01819851 |
| ENSECAG00000037674 | -1.6309901 | 0.00078943 | 0.00256815 |
| ENSECAG00000045230 | -1.6318882 | 0.00150436 | 0.00452136 |
| ENSECAG00000055538 | -1.6320298 | 0.00016743 | 0.00065926 |
| ENSECAG00000010627 | -1.6327749 | 3.50E-05 | 0.0001651 |
| ENSECAG00000023896 | -1.6329999 | 0.00200266 | 0.00582549 |
| ENSECAG00000023619 | -1.6330838 | 0.02348598 | 0.04846409 |
| ENSECAG00000023416 | -1.6339335 | 1.91E-05 | 9.64E-05 |
| ENSECAG00000041748 | -1.63426 | 0.00115258 | 0.00357517 |
| ENSECAG00000003725 | -1.6347448 | 1.09E-05 | 5.80E-05 |
| ENSECAG00000047252 | -1.6349138 | 0.00022137 | 0.00084284 |
| ENSECAG00000018196 | -1.6369982 | 6.76E-23 | 7.99E-21 |
| ENSECAG00000021405 | -1.6370474 | 2.91E-14 | 9.41E-13 |
| ENSECAG00000058058 | -1.6377077 | 0.00029699 | 0.00109154 |
| ENSECAG00000016997 | -1.639284 | 1.21E-14 | 4.13E-13 |
| ENSECAG00000015621 | -1.6396431 | 0.00487578 | 0.01261043 |
| ENSECAG00000000136 | -1.6397003 | 0.00355918 | 0.00958582 |
| ENSECAG00000044003 | -1.6398241 | 0.00247115 | 0.00698187 |
| ENSECAG00000018314 | -1.6407247 | 0.00021119 | 0.00080795 |
| ENSECAG00000056210 | -1.6409592 | 3.32E-05 | 0.00015774 |
| ENSECAG00000037673 | -1.6418118 | 0.00262518 | 0.00736287 |
| ENSECAG00000050803 | -1.6418185 | 0.01111615 | 0.02558875 |
| ENSECAG00000001411 | -1.6424321 | 0.00469293 | 0.01220675 |
| ENSECAG00000020807 | -1.6427141 | 0.00021519 | 0.00082184 |
| ENSECAG00000024945 | -1.6427933 | 4.04E-16 | 1.74E-14 |
| ENSECAG00000058395 | -1.6434249 | 0.00141145 | 0.00427953 |
| ENSECAG00000050097 | -1.6435946 | 0.00053925 | 0.00183494 |
| ENSECAG00000042683 | -1.6439135 | 0.0156094 | 0.0342472 |
| ENSECAG00000019071 | -1.6444246 | 1.17E-13 | 3.36E-12 |
| ENSECAG00000012183 | -1.6445084 | 1.90E-13 | 5.33E-12 |
| ENSECAG00000021673 | -1.6462382 | 4.35E-10 | 6.61E-09 |
| ENSECAG00000020239 | -1.646852 | 1.60E-07 | 1.36E-06 |
| ENSECAG00000043987 | -1.6469343 | 9.60E-06 | 5.22E-05 |
| ENSECAG00000050991 | -1.6474453 | 0.00032978 | 0.00119573 |
| ENSECAG00000006840 | -1.6474711 | 4.53E-09 | 5.61E-08 |
| ENSECAG00000048876 | -1.649787 | 0.00202022 | 0.00586665 |
| ENSECAG00000019230 | -1.6500884 | 2.28E-15 | 8.84E-14 |
| ENSECAG00000022087 | -1.6501305 | 7.89E-08 | 7.16E-07 |
| ENSECAG00000015997 | -1.6505813 | 0.00487214 | 0.01260251 |
| ENSECAG00000029121 | -1.6506877 | 7.74E-05 | 0.0003363 |
| ENSECAG00000050943 | -1.6510157 | 0.00124901 | 0.00383964 |
| ENSECAG00000034084 | -1.6519713 | 5.74E-06 | 3.29E-05 |
| ENSECAG00000053428 | -1.6526678 | 0.00010883 | 0.00045364 |
| ENSECAG00000059307 | -1.652934 | 0.01396036 | 0.03109322 |
| ENSECAG00000031607 | -1.6529381 | 0.00436755 | 0.0114524 |
| ENSECAG00000012686 | -1.6530953 | 1.30E-15 | 5.29E-14 |
| ENSECAG00000017260 | -1.653308 | 0.00752461 | 0.01830845 |
| ENSECAG00000047036 | -1.6541193 | 2.74E-08 | 2.79E-07 |
| ENSECAG00000016791 | -1.6545261 | 0.00090197 | 0.00288802 |
| ENSECAG00000018016 | -1.6546719 | 0.00100379 | 0.00317311 |
| ENSECAG00000007601 | -1.6549942 | 3.25E-28 | 8.45E-26 |
| ENSECAG00000025010 | -1.6552729 | 7.12E-07 | 5.13E-06 |
| ENSECAG00000019055 | -1.6557214 | 0.000128 | 0.00052089 |
| ENSECAG00000024900 | -1.6557319 | 0.01866812 | 0.03989269 |
| ENSECAG00000051000 | -1.6561448 | 0.0182196 | 0.03908373 |
| ENSECAG00000009088 | -1.6570301 | 6.78E-08 | 6.25E-07 |
| ENSECAG00000016514 | -1.6573313 | 0.00087329 | 0.00280825 |
| ENSECAG00000056480 | -1.6577173 | 0.00873157 | 0.02082565 |
| ENSECAG00000048477 | -1.6581303 | 1.79E-09 | 2.43E-08 |
| ENSECAG00000015008 | -1.6603493 | 3.64E-13 | 9.75E-12 |
| ENSECAG00000048523 | -1.6608029 | 0.00152578 | 0.00457775 |
| ENSECAG00000016384 | -1.6608053 | 4.91E-13 | 1.28E-11 |
| ENSECAG00000011463 | -1.6611447 | 8.90E-08 | 7.98E-07 |
| ENSECAG00000013079 | -1.661716 | 7.39E-10 | 1.07E-08 |
| ENSECAG00000054489 | -1.663584 | 7.80E-09 | 9.10E-08 |
| ENSECAG00000009126 | -1.6637054 | 0.0003594 | 0.00128821 |
| ENSECAG00000007880 | -1.6638605 | 1.48E-06 | 9.90E-06 |
| ENSECAG00000011504 | -1.6642164 | 4.40E-06 | 2.59E-05 |
| ENSECAG00000010215 | -1.6665406 | 1.54E-13 | 4.37E-12 |
| ENSECAG00000024291 | -1.6675713 | 2.31E-06 | 1.47E-05 |
| ENSECAG00000052977 | -1.6675805 | 9.38E-05 | 0.00039795 |
| ENSECAG00000017218 | -1.6676654 | 2.43E-07 | 1.97E-06 |
| ENSECAG00000030290 | -1.6676962 | 4.98E-07 | 3.74E-06 |
| ENSECAG00000038863 | -1.6677102 | 0.00717937 | 0.01759682 |
| ENSECAG00000010385 | -1.6683489 | 4.03E-09 | 5.04E-08 |
| ENSECAG00000023329 | -1.6693705 | 0.0084448 | 0.02024759 |
| ENSECAG00000019566 | -1.6698223 | 0.00212527 | 0.0061287 |
| ENSECAG00000010114 | -1.670603 | 9.39E-08 | 8.36E-07 |
| ENSECAG00000024454 | -1.6709564 | 0.00143074 | 0.00432806 |
| ENSECAG00000007176 | -1.6716603 | 0.00251301 | 0.00708411 |
| ENSECAG00000007305 | -1.6723483 | 1.70E-09 | 2.32E-08 |
| ENSECAG00000044533 | -1.6724418 | 0.00272883 | 0.00760425 |
| ENSECAG00000054296 | -1.6734947 | 0.00606775 | 0.0152314 |
| ENSECAG00000000791 | -1.6745557 | 4.07E-14 | 1.29E-12 |
| ENSECAG00000020700 | -1.6753847 | 8.24E-05 | 0.00035522 |
| ENSECAG00000007455 | -1.6754673 | 0.01575297 | 0.03450798 |
| ENSECAG00000009278 | -1.6771044 | 8.57E-09 | 9.88E-08 |
| ENSECAG00000021017 | -1.6783828 | 0.00848052 | 0.02032014 |
| ENSECAG00000018917 | -1.6785776 | 4.02E-05 | 0.00018729 |
| ENSECAG00000016857 | -1.6798803 | 1.45E-13 | 4.14E-12 |
| ENSECAG00000015834 | -1.6823085 | 3.73E-12 | 8.25E-11 |
| ENSECAG00000047886 | -1.682452 | 2.97E-07 | 2.35E-06 |
| ENSECAG00000011781 | -1.6826099 | 0.01395796 | 0.03109097 |
| ENSECAG00000024094 | -1.6830717 | 4.61E-05 | 0.00021193 |
| ENSECAG00000058574 | -1.6833757 | 0.0011433 | 0.00354909 |
| ENSECAG00000028204 | -1.6835336 | 3.18E-09 | 4.07E-08 |
| ENSECAG00000011760 | -1.6853309 | 2.20E-05 | 0.00010942 |
| ENSECAG00000054205 | -1.6863446 | 9.59E-09 | 1.09E-07 |
| ENSECAG00000027674 | -1.6872863 | 2.21E-05 | 0.00010961 |
| ENSECAG00000035266 | -1.6873599 | 0.00608681 | 0.01527067 |
| ENSECAG00000059749 | -1.6909035 | 0.00164557 | 0.00488859 |
| ENSECAG00000021813 | -1.6911676 | 0.00073036 | 0.00239446 |
| ENSECAG00000010546 | -1.6927498 | 5.80E-05 | 0.00025941 |
| ENSECAG00000007619 | -1.6927837 | 8.83E-20 | 7.04E-18 |
| ENSECAG00000003027 | -1.693309 | 0.00899743 | 0.02135946 |
| ENSECAG00000014798 | -1.6957913 | 4.02E-09 | 5.03E-08 |
| ENSECAG00000010966 | -1.6959345 | 9.81E-12 | 2.03E-10 |
| ENSECAG00000024568 | -1.6976761 | 1.29E-08 | 1.43E-07 |
| ENSECAG00000051394 | -1.6980348 | 5.11E-06 | 2.96E-05 |
| ENSECAG00000018835 | -1.6983708 | 0.00045807 | 0.00159119 |
| ENSECAG00000009009 | -1.6983861 | 0.00176523 | 0.00520255 |
| ENSECAG00000010748 | -1.6985613 | 0.00010279 | 0.00043177 |
| ENSECAG00000031034 | -1.6994015 | 0.00519088 | 0.01330679 |
| ENSECAG00000001609 | -1.6996793 | 0.00054613 | 0.00185609 |
| ENSECAG00000023887 | -1.6996999 | 5.97E-05 | 0.00026668 |
| ENSECAG00000015553 | -1.6999514 | 0.00382078 | 0.01020917 |
| ENSECAG00000020788 | -1.7002125 | 8.89E-09 | 1.02E-07 |
| ENSECAG00000012024 | -1.7017008 | 1.79E-18 | 1.16E-16 |
| ENSECAG00000005001 | -1.7020345 | 0.01980459 | 0.04194422 |
| ENSECAG00000019949 | -1.7020375 | 8.65E-05 | 0.00037058 |
| ENSECAG00000031572 | -1.7026145 | 0.01475298 | 0.03259681 |
| ENSECAG00000010418 | -1.7029231 | 0.00021603 | 0.00082449 |
| ENSECAG00000019455 | -1.703419 | 2.19E-05 | 0.00010903 |
| ENSECAG00000012793 | -1.7039068 | 1.00E-12 | 2.47E-11 |
| ENSECAG00000023526 | -1.7041947 | 2.03E-05 | 0.00010172 |
| ENSECAG00000010956 | -1.7047146 | 0.00189226 | 0.00553316 |
| ENSECAG00000051899 | -1.7047438 | 0.00590872 | 0.01488402 |
| ENSECAG00000032109 | -1.7057723 | 0.00260817 | 0.00731913 |
| ENSECAG00000004778 | -1.7058062 | 0.00616629 | 0.01544059 |
| ENSECAG00000000574 | -1.7060697 | 0.00978034 | 0.02293059 |
| ENSECAG00000050593 | -1.706466 | 0.01896 | 0.04043522 |
| ENSECAG00000003090 | -1.7068533 | 0.00634529 | 0.01583912 |
| ENSECAG00000010142 | -1.7074565 | 3.32E-06 | 2.03E-05 |
| ENSECAG00000019125 | -1.7085491 | 1.94E-11 | 3.81E-10 |
| ENSECAG00000048031 | -1.7087448 | 0.00237222 | 0.00674593 |
| ENSECAG00000014907 | -1.7092633 | 0.00282877 | 0.00783583 |
| ENSECAG00000046594 | -1.7093456 | 1.30E-10 | 2.21E-09 |
| ENSECAG00000047273 | -1.7094382 | 0.01432692 | 0.03178292 |
| ENSECAG00000012530 | -1.710628 | 0.00621884 | 0.01556171 |
| ENSECAG00000017083 | -1.7116258 | 1.14E-05 | 6.05E-05 |
| ENSECAG00000020743 | -1.7116761 | 5.20E-07 | 3.90E-06 |
| ENSECAG00000050021 | -1.7121221 | 0.00105158 | 0.00330083 |
| ENSECAG00000031501 | -1.7123878 | 5.78E-09 | 6.97E-08 |
| ENSECAG00000010778 | -1.712828 | 2.38E-10 | 3.83E-09 |
| ENSECAG00000052359 | -1.7137795 | 0.00020101 | 0.00077485 |
| ENSECAG00000004678 | -1.7144677 | 1.81E-11 | 3.60E-10 |
| ENSECAG00000032285 | -1.7147254 | 2.04E-13 | 5.69E-12 |
| ENSECAG00000018044 | -1.7169916 | 4.64E-05 | 0.00021267 |
| ENSECAG00000017304 | -1.7178494 | 3.16E-06 | 1.94E-05 |
| ENSECAG00000057045 | -1.7186171 | 0.00028152 | 0.00104067 |
| ENSECAG00000057622 | -1.7204796 | 0.00756873 | 0.01839577 |
| ENSECAG00000011024 | -1.7206011 | 3.09E-06 | 1.90E-05 |
| ENSECAG00000040667 | -1.7214768 | 0.00097333 | 0.00308555 |
| ENSECAG00000000133 | -1.7215053 | 0.00013651 | 0.00055074 |
| ENSECAG00000060368 | -1.7229859 | 8.13E-07 | 5.77E-06 |
| ENSECAG00000024137 | -1.7234843 | 4.24E-05 | 0.00019652 |
| ENSECAG00000011618 | -1.7235211 | 2.73E-06 | 1.70E-05 |
| ENSECAG00000059625 | -1.7236891 | 0.00388255 | 0.01035694 |
| ENSECAG00000018949 | -1.723811 | 1.32E-15 | 5.37E-14 |
| ENSECAG00000022956 | -1.7243802 | 1.02E-08 | 1.15E-07 |
| ENSECAG00000046739 | -1.7257416 | 0.01058479 | 0.02454115 |
| ENSECAG00000016284 | -1.7257879 | 0.00016554 | 0.00065309 |
| ENSECAG00000044750 | -1.7271231 | 0.0025726 | 0.00723384 |
| ENSECAG00000049778 | -1.727572 | 0.00069319 | 0.0022877 |
| ENSECAG00000025128 | -1.7277084 | 0.00040471 | 0.00142736 |
| ENSECAG00000050153 | -1.728863 | 0.00031907 | 0.00116312 |
| ENSECAG00000001098 | -1.7289896 | 0.00097138 | 0.00308068 |
| ENSECAG00000007276 | -1.7293938 | 0.00074126 | 0.00242665 |
| ENSECAG00000025278 | -1.7297669 | 0.0006361 | 0.00212184 |
| ENSECAG00000047261 | -1.7298622 | 0.00212847 | 0.00613635 |
| ENSECAG00000019869 | -1.7301966 | 1.90E-06 | 1.24E-05 |
| ENSECAG00000059476 | -1.7313164 | 0.007091 | 0.01741079 |
| ENSECAG00000024488 | -1.7324218 | 0.00593259 | 0.01493575 |
| ENSECAG00000012053 | -1.7329706 | 0.00011505 | 0.00047585 |
| ENSECAG00000009311 | -1.7334905 | 1.63E-08 | 1.76E-07 |
| ENSECAG00000001443 | -1.7343878 | 4.05E-16 | 1.74E-14 |
| ENSECAG00000016232 | -1.7351566 | 0.00836699 | 0.02009985 |
| ENSECAG00000045391 | -1.7353034 | 0.00302661 | 0.00831237 |
| ENSECAG00000033026 | -1.7362539 | 3.32E-06 | 2.02E-05 |
| ENSECAG00000025921 | -1.7372136 | 0.00044084 | 0.0015385 |
| ENSECAG00000015853 | -1.7377774 | 6.51E-11 | 1.17E-09 |
| ENSECAG00000013087 | -1.738161 | 6.81E-12 | 1.45E-10 |
| ENSECAG00000008681 | -1.7385843 | 0.00599995 | 0.01508154 |
| ENSECAG00000058481 | -1.739279 | 0.00561104 | 0.01423527 |
| ENSECAG00000034558 | -1.7401025 | 2.80E-05 | 0.0001356 |
| ENSECAG00000022313 | -1.7411183 | 0.00331237 | 0.00900683 |
| ENSECAG00000009767 | -1.7413032 | 4.89E-14 | 1.53E-12 |
| ENSECAG00000044431 | -1.7429481 | 2.86E-09 | 3.71E-08 |
| ENSECAG00000055198 | -1.7431389 | 0.01936843 | 0.04114526 |
| ENSECAG00000020097 | -1.7438242 | 0.00215617 | 0.00620578 |
| ENSECAG00000014817 | -1.7444224 | 1.15E-16 | 5.47E-15 |
| ENSECAG00000027014 | -1.7449932 | 1.04E-05 | 5.58E-05 |
| ENSECAG00000015923 | -1.7459074 | 1.65E-06 | 1.09E-05 |
| ENSECAG00000026615 | -1.7464683 | 2.64E-06 | 1.66E-05 |
| ENSECAG00000006011 | -1.7467299 | 4.90E-05 | 0.00022365 |
| ENSECAG00000000813 | -1.7469995 | 0.00319301 | 0.0087109 |
| ENSECAG00000038632 | -1.7485295 | 1.92E-18 | 1.23E-16 |
| ENSECAG00000059041 | -1.7485903 | 0.00160503 | 0.00478068 |
| ENSECAG00000016141 | -1.7493015 | 6.46E-06 | 3.65E-05 |
| ENSECAG00000017697 | -1.7502335 | 8.23E-14 | 2.45E-12 |
| ENSECAG00000059021 | -1.7504608 | 0.01744292 | 0.03761988 |
| ENSECAG00000055148 | -1.750503 | 0.0027371 | 0.0076207 |
| ENSECAG00000010004 | -1.7509559 | 1.02E-07 | 9.05E-07 |
| ENSECAG00000055119 | -1.752023 | 0.00734329 | 0.01794348 |
| ENSECAG00000022093 | -1.7550531 | 0.00119464 | 0.00369129 |
| ENSECAG00000043864 | -1.7552456 | 1.48E-10 | 2.48E-09 |
| ENSECAG00000021583 | -1.755374 | 2.44E-07 | 1.97E-06 |
| ENSECAG00000042441 | -1.755514 | 0.00860162 | 0.0205728 |
| ENSECAG00000022618 | -1.7561028 | 0.00625962 | 0.0156515 |
| ENSECAG00000039031 | -1.7587313 | 0.00252675 | 0.00711875 |
| ENSECAG00000017471 | -1.7597542 | 0.00214036 | 0.00616822 |
| ENSECAG00000059791 | -1.7604323 | 0.00014211 | 0.00057146 |
| ENSECAG00000044489 | -1.7608837 | 1.02E-05 | 5.50E-05 |
| ENSECAG00000021101 | -1.7613101 | 3.10E-19 | 2.30E-17 |
| ENSECAG00000012871 | -1.7616833 | 7.68E-07 | 5.49E-06 |
| ENSECAG00000020130 | -1.7622883 | 0.01785531 | 0.03840919 |
| ENSECAG00000036001 | -1.762753 | 0.00292259 | 0.00806771 |
| ENSECAG00000014750 | -1.7628221 | 0.00389428 | 0.01038072 |
| ENSECAG00000015435 | -1.7643597 | 0.00126886 | 0.00389317 |
| ENSECAG00000021518 | -1.7644717 | 2.14E-16 | 9.75E-15 |
| ENSECAG00000012348 | -1.7646915 | 3.70E-08 | 3.64E-07 |
| ENSECAG00000014867 | -1.7651834 | 5.38E-08 | 5.08E-07 |
| ENSECAG00000000514 | -1.7667608 | 2.75E-07 | 2.20E-06 |
| ENSECAG00000046147 | -1.769755 | 0.01483091 | 0.03274495 |
| ENSECAG00000008765 | -1.770031 | 4.04E-05 | 0.0001881 |
| ENSECAG00000008032 | -1.7704309 | 6.77E-08 | 6.25E-07 |
| ENSECAG00000048245 | -1.7722075 | 0.00079937 | 0.00259491 |
| ENSECAG00000044954 | -1.7729886 | 9.49E-08 | 8.44E-07 |
| ENSECAG00000021646 | -1.774194 | 0.00022794 | 0.00086494 |
| ENSECAG00000057205 | -1.774564 | 0.00037075 | 0.00132135 |
| ENSECAG00000023512 | -1.7757221 | 1.18E-16 | 5.62E-15 |
| ENSECAG00000046087 | -1.7759562 | 0.00044687 | 0.00155735 |
| ENSECAG00000017375 | -1.7761403 | 0.00036377 | 0.00130186 |
| ENSECAG00000013175 | -1.7767517 | 0.01194664 | 0.02719373 |
| ENSECAG00000055708 | -1.7768957 | 0.00622767 | 0.01558208 |
| ENSECAG00000055489 | -1.776982 | 5.31E-08 | 5.02E-07 |
| ENSECAG00000021284 | -1.7773511 | 0.00023493 | 0.00088828 |
| ENSECAG00000024064 | -1.7780426 | 5.84E-06 | 3.34E-05 |
| ENSECAG00000009140 | -1.7781317 | 2.06E-09 | 2.76E-08 |
| ENSECAG00000016196 | -1.7807052 | 0.00201349 | 0.0058509 |
| ENSECAG00000006419 | -1.7807461 | 4.65E-15 | 1.72E-13 |
| ENSECAG00000038775 | -1.7809829 | 0.00300389 | 0.00825646 |
| ENSECAG00000019677 | -1.7809907 | 3.90E-06 | 2.34E-05 |
| ENSECAG00000000634 | -1.7813673 | 3.38E-06 | 2.06E-05 |
| ENSECAG00000051149 | -1.7838405 | 0.01037483 | 0.024142 |
| ENSECAG00000059411 | -1.7841722 | 0.00010283 | 0.00043183 |
| ENSECAG00000016256 | -1.7874981 | 0.00036922 | 0.0013165 |
| ENSECAG00000016483 | -1.7879142 | 7.96E-06 | 4.40E-05 |
| ENSECAG00000001074 | -1.788081 | 1.19E-07 | 1.04E-06 |
| ENSECAG00000023757 | -1.7898091 | 1.16E-11 | 2.38E-10 |
| ENSECAG00000017998 | -1.789907 | 8.34E-09 | 9.64E-08 |
| ENSECAG00000020664 | -1.7908748 | 2.11E-19 | 1.59E-17 |
| ENSECAG00000018546 | -1.7912222 | 0.00254618 | 0.00716947 |
| ENSECAG00000057824 | -1.7916173 | 1.16E-05 | 6.15E-05 |
| ENSECAG00000009250 | -1.7930983 | 1.05E-19 | 8.33E-18 |
| ENSECAG00000009712 | -1.7941801 | 3.40E-09 | 4.31E-08 |
| ENSECAG00000011884 | -1.794641 | 0.02119079 | 0.04444615 |
| ENSECAG00000016498 | -1.7954755 | 0.00995777 | 0.02328308 |
| ENSECAG00000023509 | -1.7984955 | 7.35E-09 | 8.64E-08 |
| ENSECAG00000043163 | -1.7985749 | 9.65E-07 | 6.74E-06 |
| ENSECAG00000010579 | -1.7989638 | 0.00229638 | 0.00656032 |
| ENSECAG00000002551 | -1.7991136 | 0.00026772 | 0.00099725 |
| ENSECAG00000021168 | -1.7997756 | 1.53E-05 | 7.86E-05 |
| ENSECAG00000019943 | -1.8006721 | 3.34E-22 | 3.62E-20 |
| ENSECAG00000043237 | -1.8025604 | 0.0009834 | 0.00311393 |
| ENSECAG00000051410 | -1.8041168 | 2.03E-07 | 1.68E-06 |
| ENSECAG00000030444 | -1.8041925 | 0.01720084 | 0.03716951 |
| ENSECAG00000056303 | -1.8058045 | 7.54E-07 | 5.40E-06 |
| ENSECAG00000052324 | -1.8081455 | 0.00012156 | 0.00049868 |
| ENSECAG00000057381 | -1.8088991 | 0.00013784 | 0.00055557 |
| ENSECAG00000025630 | -1.8092174 | 0.00470326 | 0.01222902 |
| ENSECAG00000045822 | -1.8103554 | 0.00804959 | 0.01943767 |
| ENSECAG00000051876 | -1.8104938 | 0.0055525 | 0.01411239 |
| ENSECAG00000057891 | -1.8105526 | 8.81E-07 | 6.20E-06 |
| ENSECAG00000022549 | -1.8107613 | 0.01180567 | 0.02694409 |
| ENSECAG00000024422 | -1.8121118 | 0.00865445 | 0.02068146 |
| ENSECAG00000006853 | -1.8137452 | 5.86E-06 | 3.35E-05 |
| ENSECAG00000009475 | -1.8139096 | 0.00437733 | 0.01147399 |
| ENSECAG00000028812 | -1.8143638 | 0.01940223 | 0.04119573 |
| ENSECAG00000036739 | -1.8144685 | 0.00054094 | 0.00183957 |
| ENSECAG00000060335 | -1.8149874 | 0.00058 | 0.00195691 |
| ENSECAG00000009004 | -1.8151228 | 0.02428034 | 0.04985926 |
| ENSECAG00000018420 | -1.8162159 | 2.38E-10 | 3.83E-09 |
| ENSECAG00000010717 | -1.816428 | 6.37E-05 | 0.00028288 |
| ENSECAG00000024348 | -1.8168374 | 0.00919869 | 0.02174955 |
| ENSECAG00000009541 | -1.8176319 | 0.00032171 | 0.00117159 |
| ENSECAG00000018714 | -1.8182257 | 3.73E-07 | 2.88E-06 |
| ENSECAG00000017112 | -1.8183614 | 0.00194977 | 0.00568497 |
| ENSECAG00000010925 | -1.8186055 | 2.56E-06 | 1.61E-05 |
| ENSECAG00000007690 | -1.8195792 | 4.09E-16 | 1.75E-14 |
| ENSECAG00000006337 | -1.8195971 | 2.14E-08 | 2.25E-07 |
| ENSECAG00000037540 | -1.8202145 | 0.00063488 | 0.00211873 |
| ENSECAG00000020000 | -1.8207031 | 0.00076115 | 0.00248482 |
| ENSECAG00000008699 | -1.8209639 | 9.03E-08 | 8.08E-07 |
| ENSECAG00000021383 | -1.8211719 | 9.31E-11 | 1.63E-09 |
| ENSECAG00000020464 | -1.8214512 | 0.00012883 | 0.00052391 |
| ENSECAG00000014312 | -1.8235999 | 7.19E-08 | 6.59E-07 |
| ENSECAG00000044428 | -1.8237987 | 0.00195075 | 0.00568633 |
| ENSECAG00000017366 | -1.8238663 | 2.45E-07 | 1.98E-06 |
| ENSECAG00000033235 | -1.8249842 | 0.00511922 | 0.01315481 |
| ENSECAG00000006267 | -1.8258377 | 3.74E-05 | 0.00017516 |
| ENSECAG00000023882 | -1.8265125 | 1.37E-05 | 7.14E-05 |
| ENSECAG00000026968 | -1.826897 | 0.00013838 | 0.00055747 |
| ENSECAG00000021259 | -1.8269864 | 0.00116269 | 0.00360354 |
| ENSECAG00000005958 | -1.8271134 | 4.11E-08 | 4.00E-07 |
| ENSECAG00000056636 | -1.8274775 | 5.13E-06 | 2.97E-05 |
| ENSECAG00000005507 | -1.8278127 | 8.93E-07 | 6.28E-06 |
| ENSECAG00000054155 | -1.8281248 | 0.00308078 | 0.00843148 |
| ENSECAG00000037341 | -1.8284451 | 0.01698561 | 0.03676843 |
| ENSECAG00000053464 | -1.8298525 | 1.51E-06 | 1.01E-05 |
| ENSECAG00000012644 | -1.8301148 | 0.0041313 | 0.01090328 |
| ENSECAG00000009649 | -1.8302458 | 5.49E-07 | 4.09E-06 |
| ENSECAG00000014078 | -1.8303417 | 0.0164305 | 0.03575382 |
| ENSECAG00000022256 | -1.830651 | 2.69E-05 | 0.00013071 |
| ENSECAG00000024388 | -1.8319162 | 0.00098016 | 0.00310413 |
| ENSECAG00000048120 | -1.8331607 | 0.01284539 | 0.02892357 |
| ENSECAG00000050612 | -1.8332858 | 0.00197902 | 0.00576198 |
| ENSECAG00000009125 | -1.8333282 | 0.00018636 | 0.00072324 |
| ENSECAG00000005285 | -1.8335247 | 8.79E-14 | 2.60E-12 |
| ENSECAG00000019543 | -1.8343666 | 0.00023136 | 0.00087614 |
| ENSECAG00000052743 | -1.8343826 | 0.01515691 | 0.0333691 |
| ENSECAG00000051474 | -1.8358256 | 0.02163468 | 0.04522014 |
| ENSECAG00000011138 | -1.839351 | 1.93E-06 | 1.25E-05 |
| ENSECAG00000034304 | -1.8396997 | 0.00668343 | 0.01654824 |
| ENSECAG00000038178 | -1.8397185 | 3.01E-09 | 3.87E-08 |
| ENSECAG00000018019 | -1.8402417 | 0.00166232 | 0.00493375 |
| ENSECAG00000057885 | -1.8405537 | 0.00736841 | 0.0179911 |
| ENSECAG00000002692 | -1.840785 | 5.01E-14 | 1.57E-12 |
| ENSECAG00000011985 | -1.842539 | 1.23E-07 | 1.07E-06 |
| ENSECAG00000016809 | -1.842821 | 2.30E-11 | 4.47E-10 |
| ENSECAG00000044272 | -1.8434683 | 0.00683969 | 0.01686787 |
| ENSECAG00000010380 | -1.8449889 | 7.49E-05 | 0.0003268 |
| ENSECAG00000011570 | -1.8465795 | 1.30E-08 | 1.43E-07 |
| ENSECAG00000013651 | -1.8468751 | 0.00735666 | 0.01796828 |
| ENSECAG00000024954 | -1.8469657 | 6.37E-11 | 1.15E-09 |
| ENSECAG00000050038 | -1.8471436 | 8.92E-10 | 1.28E-08 |
| ENSECAG00000054636 | -1.8479338 | 0.00799961 | 0.01933369 |
| ENSECAG00000050808 | -1.8488301 | 4.60E-11 | 8.53E-10 |
| ENSECAG00000031428 | -1.8503724 | 2.43E-06 | 1.54E-05 |
| ENSECAG00000001068 | -1.8520163 | 0.01614971 | 0.03524922 |
| ENSECAG00000057583 | -1.8520329 | 0.008592 | 0.020552 |
| ENSECAG00000010419 | -1.852292 | 1.31E-05 | 6.89E-05 |
| ENSECAG00000023819 | -1.8528325 | 0.01230562 | 0.02788293 |
| ENSECAG00000052703 | -1.8533957 | 1.09E-09 | 1.54E-08 |
| ENSECAG00000015145 | -1.8550318 | 2.17E-16 | 9.83E-15 |
| ENSECAG00000003167 | -1.8552733 | 0.00024116 | 0.00090831 |
| ENSECAG00000026344 | -1.8557813 | 0.01914943 | 0.04076141 |
| ENSECAG00000045314 | -1.8566083 | 6.64E-09 | 7.89E-08 |
| ENSECAG00000045083 | -1.8568187 | 0.00269746 | 0.00752903 |
| ENSECAG00000012910 | -1.8568701 | 5.17E-06 | 2.99E-05 |
| ENSECAG00000050104 | -1.8572401 | 0.00097697 | 0.00309571 |
| ENSECAG00000002508 | -1.8574249 | 1.46E-10 | 2.44E-09 |
| ENSECAG00000039676 | -1.8574708 | 0.00047342 | 0.00163634 |
| ENSECAG00000056542 | -1.8575329 | 0.02413405 | 0.04962081 |
| ENSECAG00000024578 | -1.8579794 | 1.83E-10 | 3.02E-09 |
| ENSECAG00000001845 | -1.8583643 | 2.82E-05 | 0.00013636 |
| ENSECAG00000023247 | -1.8585545 | 0.00230599 | 0.0065819 |
| ENSECAG00000050852 | -1.8592742 | 0.02256066 | 0.04685898 |
| ENSECAG00000047620 | -1.8601098 | 0.00055736 | 0.00188907 |
| ENSECAG00000029610 | -1.8615035 | 0.00012875 | 0.00052366 |
| ENSECAG00000024904 | -1.8621056 | 0.00365757 | 0.00982475 |
| ENSECAG00000021418 | -1.8634904 | 0.00490431 | 0.01267249 |
| ENSECAG00000028448 | -1.8635197 | 0.00056488 | 0.00191116 |
| ENSECAG00000014031 | -1.86665 | 0.0027832 | 0.00772494 |
| ENSECAG00000054605 | -1.8674538 | 0.01071599 | 0.0247942 |
| ENSECAG00000034930 | -1.8675842 | 0.00034439 | 0.00124244 |
| ENSECAG00000017379 | -1.8680682 | 0.00086069 | 0.00277132 |
| ENSECAG00000018904 | -1.8684644 | 2.50E-08 | 2.58E-07 |
| ENSECAG00000050787 | -1.8684878 | 3.80E-14 | 1.21E-12 |
| ENSECAG00000021674 | -1.8685639 | 0.00587802 | 0.01482342 |
| ENSECAG00000043951 | -1.8690101 | 6.67E-05 | 0.00029413 |
| ENSECAG00000016522 | -1.8690142 | 1.15E-05 | 6.12E-05 |
| ENSECAG00000019211 | -1.8706996 | 0.00219432 | 0.00630422 |
| ENSECAG00000005039 | -1.8707322 | 4.19E-14 | 1.32E-12 |
| ENSECAG00000058735 | -1.8714082 | 0.00113623 | 0.00352887 |
| ENSECAG00000020835 | -1.8722121 | 2.12E-05 | 0.00010572 |
| ENSECAG00000048369 | -1.8744404 | 0.00011527 | 0.00047656 |
| ENSECAG00000038509 | -1.8746091 | 6.81E-08 | 6.28E-07 |
| ENSECAG00000022555 | -1.8747569 | 0.00827245 | 0.01990055 |
| ENSECAG00000007574 | -1.8750481 | 0.00058351 | 0.00196637 |
| ENSECAG00000016323 | -1.8753431 | 5.71E-05 | 0.0002558 |
| ENSECAG00000045995 | -1.8753498 | 0.00238067 | 0.00676222 |
| ENSECAG00000014247 | -1.8753927 | 1.67E-13 | 4.73E-12 |
| ENSECAG00000020468 | -1.8783079 | 5.03E-13 | 1.32E-11 |
| ENSECAG00000024940 | -1.8784504 | 0.00908222 | 0.02152416 |
| ENSECAG00000057595 | -1.8792434 | 0.00010727 | 0.00044811 |
| ENSECAG00000057211 | -1.8799816 | 0.00036837 | 0.00131431 |
| ENSECAG00000037866 | -1.8815932 | 0.01147603 | 0.02630514 |
| ENSECAG00000041945 | -1.8816345 | 0.00202582 | 0.0058791 |
| ENSECAG00000056548 | -1.8822409 | 0.00555472 | 0.01411644 |
| ENSECAG00000041448 | -1.8838581 | 0.00491717 | 0.01270425 |
| ENSECAG00000026356 | -1.8860792 | 0.00017445 | 0.00068308 |
| ENSECAG00000049020 | -1.8862874 | 3.12E-06 | 1.92E-05 |
| ENSECAG00000023570 | -1.8875323 | 7.79E-09 | 9.09E-08 |
| ENSECAG00000019515 | -1.8878093 | 0.01197542 | 0.02724246 |
| ENSECAG00000044802 | -1.8890664 | 0.01898117 | 0.04046877 |
| ENSECAG00000058822 | -1.8891175 | 2.01E-05 | 0.00010077 |
| ENSECAG00000018218 | -1.8899268 | 6.33E-13 | 1.63E-11 |
| ENSECAG00000057295 | -1.8911412 | 0.00564068 | 0.01430048 |
| ENSECAG00000047314 | -1.8912745 | 0.00174033 | 0.00514205 |
| ENSECAG00000008565 | -1.8933499 | 4.11E-05 | 0.00019112 |
| ENSECAG00000010262 | -1.8946522 | 0.00170256 | 0.00504179 |
| ENSECAG00000012967 | -1.8948379 | 1.86E-10 | 3.05E-09 |
| ENSECAG00000059557 | -1.8951244 | 0.02269373 | 0.04710329 |
| ENSECAG00000013821 | -1.8969622 | 9.45E-05 | 0.00040014 |
| ENSECAG00000014566 | -1.8971717 | 5.33E-07 | 3.99E-06 |
| ENSECAG00000015359 | -1.8981021 | 9.05E-10 | 1.30E-08 |
| ENSECAG00000017232 | -1.8996026 | 9.81E-11 | 1.72E-09 |
| ENSECAG00000015554 | -1.8996744 | 1.07E-16 | 5.11E-15 |
| ENSECAG00000014358 | -1.8998724 | 1.96E-09 | 2.64E-08 |
| ENSECAG00000011305 | -1.8999004 | 8.83E-08 | 7.92E-07 |
| ENSECAG00000018954 | -1.9030365 | 0.00998154 | 0.02333133 |
| ENSECAG00000012131 | -1.9030667 | 3.79E-06 | 2.28E-05 |
| ENSECAG00000037736 | -1.9032264 | 0.01107038 | 0.02549769 |
| ENSECAG00000022054 | -1.9032485 | 1.74E-05 | 8.86E-05 |
| ENSECAG00000037382 | -1.9041591 | 6.95E-06 | 3.89E-05 |
| ENSECAG00000045477 | -1.9045189 | 9.42E-05 | 0.00039908 |
| ENSECAG00000060246 | -1.9057105 | 1.67E-07 | 1.41E-06 |
| ENSECAG00000019060 | -1.9063933 | 1.08E-08 | 1.22E-07 |
| ENSECAG00000017364 | -1.9082825 | 0.00117299 | 0.00363194 |
| ENSECAG00000015337 | -1.9087216 | 1.41E-05 | 7.34E-05 |
| ENSECAG00000050686 | -1.9087554 | 0.00530895 | 0.01358517 |
| ENSECAG00000057558 | -1.9089806 | 0.00697168 | 0.01715739 |
| ENSECAG00000024829 | -1.9092294 | 0.01821756 | 0.03908373 |
| ENSECAG00000038427 | -1.9100836 | 7.48E-06 | 4.17E-05 |
| ENSECAG00000021043 | -1.9107961 | 0.00019769 | 0.00076337 |
| ENSECAG00000002755 | -1.9110302 | 0.00038926 | 0.00137937 |
| ENSECAG00000028762 | -1.9120538 | 0.00641403 | 0.01598215 |
| ENSECAG00000055915 | -1.9121015 | 7.25E-09 | 8.53E-08 |
| ENSECAG00000020489 | -1.9129625 | 6.11E-05 | 0.00027257 |
| ENSECAG00000048720 | -1.913407 | 2.37E-05 | 0.00011675 |
| ENSECAG00000010143 | -1.9139645 | 1.72E-05 | 8.77E-05 |
| ENSECAG00000060223 | -1.9145141 | 0.00636718 | 0.01588312 |
| ENSECAG00000058885 | -1.914747 | 0.00015628 | 0.00062075 |
| ENSECAG00000052542 | -1.9151424 | 0.00085605 | 0.00275796 |
| ENSECAG00000027676 | -1.9162243 | 2.38E-08 | 2.47E-07 |
| ENSECAG00000010880 | -1.9162961 | 7.41E-05 | 0.00032381 |
| ENSECAG00000053274 | -1.9165751 | 0.00696672 | 0.01714841 |
| ENSECAG00000034308 | -1.9167335 | 0.00015188 | 0.0006053 |
| ENSECAG00000024899 | -1.9174333 | 3.85E-13 | 1.03E-11 |
| ENSECAG00000005173 | -1.9181724 | 5.09E-09 | 6.22E-08 |
| ENSECAG00000045194 | -1.9182989 | 0.00653322 | 0.01622666 |
| ENSECAG00000055773 | -1.9184501 | 6.07E-05 | 0.00027069 |
| ENSECAG00000057499 | -1.9185045 | 0.00015009 | 0.00059922 |
| ENSECAG00000049706 | -1.9187036 | 0.00111248 | 0.00346426 |
| ENSECAG00000053353 | -1.9188805 | 0.00108545 | 0.00339048 |
| ENSECAG00000031378 | -1.9205091 | 0.00467301 | 0.01216059 |
| ENSECAG00000016134 | -1.9212605 | 1.15E-13 | 3.32E-12 |
| ENSECAG00000018373 | -1.9213744 | 1.65E-17 | 9.16E-16 |
| ENSECAG00000011365 | -1.9218003 | 1.67E-09 | 2.28E-08 |
| ENSECAG00000049279 | -1.9219234 | 0.01423302 | 0.03159344 |
| ENSECAG00000045182 | -1.922403 | 0.02398909 | 0.04935644 |
| ENSECAG00000050801 | -1.9227928 | 0.00095949 | 0.00304858 |
| ENSECAG00000032692 | -1.9229925 | 0.00132906 | 0.00406003 |
| ENSECAG00000010713 | -1.9231589 | 0.00013025 | 0.00052892 |
| ENSECAG00000014418 | -1.9246334 | 0.01965474 | 0.04166237 |
| ENSECAG00000037681 | -1.9246718 | 2.67E-09 | 3.48E-08 |
| ENSECAG00000016768 | -1.9262399 | 4.56E-15 | 1.69E-13 |
| ENSECAG00000016320 | -1.9263852 | 5.66E-05 | 0.00025384 |
| ENSECAG00000008347 | -1.9265717 | 5.73E-05 | 0.00025668 |
| ENSECAG00000011222 | -1.926728 | 0.00117035 | 0.00362475 |
| ENSECAG00000003067 | -1.9268286 | 0.00094314 | 0.00300392 |
| ENSECAG00000026975 | -1.9289718 | 0.00102141 | 0.00322425 |
| ENSECAG00000053457 | -1.9289901 | 0.0005501 | 0.00186702 |
| ENSECAG00000029679 | -1.9296565 | 0.00094095 | 0.00299865 |
| ENSECAG00000008038 | -1.930221 | 1.62E-06 | 1.07E-05 |
| ENSECAG00000019668 | -1.9315115 | 0.00015004 | 0.00059922 |
| ENSECAG00000024910 | -1.9324835 | 2.40E-05 | 0.000118 |
| ENSECAG00000018474 | -1.9332835 | 1.37E-06 | 9.24E-06 |
| ENSECAG00000019919 | -1.9334679 | 0.00043134 | 0.00150886 |
| ENSECAG00000024890 | -1.9338582 | 1.57E-15 | 6.30E-14 |
| ENSECAG00000039123 | -1.9356954 | 0.01291405 | 0.0290553 |
| ENSECAG00000024293 | -1.9361503 | 0.00036461 | 0.00130425 |
| ENSECAG00000000532 | -1.936364 | 0.00023741 | 0.00089646 |
| ENSECAG00000008408 | -1.9363854 | 5.61E-08 | 5.27E-07 |
| ENSECAG00000007220 | -1.9365691 | 3.85E-17 | 1.98E-15 |
| ENSECAG00000007044 | -1.9367648 | 3.26E-05 | 0.0001552 |
| ENSECAG00000017035 | -1.9376815 | 3.13E-09 | 4.01E-08 |
| ENSECAG00000010412 | -1.9382272 | 3.32E-17 | 1.74E-15 |
| ENSECAG00000045750 | -1.9385504 | 7.01E-06 | 3.93E-05 |
| ENSECAG00000016129 | -1.9394954 | 9.24E-14 | 2.72E-12 |
| ENSECAG00000007281 | -1.9404408 | 2.90E-23 | 3.66E-21 |
| ENSECAG00000021034 | -1.9419379 | 1.07E-07 | 9.42E-07 |
| ENSECAG00000058258 | -1.9420922 | 0.00020552 | 0.0007895 |
| ENSECAG00000059921 | -1.9421261 | 0.00011526 | 0.00047656 |
| ENSECAG00000019692 | -1.9453523 | 0.00077323 | 0.00252019 |
| ENSECAG00000001290 | -1.9458987 | 9.30E-13 | 2.32E-11 |
| ENSECAG00000044660 | -1.9474592 | 4.85E-06 | 2.83E-05 |
| ENSECAG00000016260 | -1.9487152 | 2.38E-05 | 0.00011706 |
| ENSECAG00000022817 | -1.9501317 | 3.84E-07 | 2.96E-06 |
| ENSECAG00000040907 | -1.9504692 | 0.0010696 | 0.00334751 |
| ENSECAG00000007330 | -1.9507908 | 4.37E-08 | 4.22E-07 |
| ENSECAG00000024219 | -1.9524498 | 1.50E-08 | 1.63E-07 |
| ENSECAG00000014851 | -1.954105 | 6.92E-15 | 2.48E-13 |
| ENSECAG00000046240 | -1.954888 | 1.07E-13 | 3.10E-12 |
| ENSECAG00000055409 | -1.9553745 | 1.00E-06 | 6.98E-06 |
| ENSECAG00000029287 | -1.9554156 | 1.52E-07 | 1.30E-06 |
| ENSECAG00000021331 | -1.9582243 | 5.35E-07 | 4.00E-06 |
| ENSECAG00000018292 | -1.9590271 | 6.12E-15 | 2.21E-13 |
| ENSECAG00000052278 | -1.9593329 | 4.16E-06 | 2.47E-05 |
| ENSECAG00000022681 | -1.9595962 | 9.24E-14 | 2.72E-12 |
| ENSECAG00000017789 | -1.9599473 | 7.11E-05 | 0.0003114 |
| ENSECAG00000059375 | -1.9609045 | 0.00091871 | 0.0029332 |
| ENSECAG00000000134 | -1.9616865 | 4.76E-16 | 2.02E-14 |
| ENSECAG00000038712 | -1.9621704 | 0.00049668 | 0.00170357 |
| ENSECAG00000050442 | -1.9623689 | 0.02318344 | 0.04794161 |
| ENSECAG00000007878 | -1.9628736 | 8.54E-13 | 2.13E-11 |
| ENSECAG00000014758 | -1.9628803 | 0.00699256 | 0.0172012 |
| ENSECAG00000008764 | -1.9636842 | 4.19E-07 | 3.19E-06 |
| ENSECAG00000046525 | -1.9637376 | 0.00031521 | 0.00115037 |
| ENSECAG00000012734 | -1.9652291 | 8.00E-06 | 4.42E-05 |
| ENSECAG00000053773 | -1.9662243 | 0.00677717 | 0.01673769 |
| ENSECAG00000008304 | -1.9667266 | 0.00013335 | 0.0005396 |
| ENSECAG00000015001 | -1.9680282 | 0.00413036 | 0.01090208 |
| ENSECAG00000008200 | -1.9681314 | 2.88E-07 | 2.29E-06 |
| ENSECAG00000022420 | -1.9682767 | 2.20E-12 | 5.10E-11 |
| ENSECAG00000057281 | -1.9687474 | 0.00287102 | 0.00793993 |
| ENSECAG00000011044 | -1.9693129 | 3.98E-06 | 2.37E-05 |
| ENSECAG00000057502 | -1.9694991 | 0.0006448 | 0.00214669 |
| ENSECAG00000001261 | -1.969944 | 4.96E-05 | 0.00022591 |
| ENSECAG00000000863 | -1.9702492 | 4.31E-07 | 3.28E-06 |
| ENSECAG00000013520 | -1.9709701 | 0.00953263 | 0.02244165 |
| ENSECAG00000014321 | -1.9719704 | 1.62E-06 | 1.07E-05 |
| ENSECAG00000045233 | -1.9723881 | 0.00923606 | 0.02182347 |
| ENSECAG00000056702 | -1.9729295 | 0.0021415 | 0.00617073 |
| ENSECAG00000049018 | -1.9743049 | 6.25E-05 | 0.00027804 |
| ENSECAG00000058918 | -1.97453 | 0.00411658 | 0.01087085 |
| ENSECAG00000008728 | -1.9759932 | 2.37E-09 | 3.13E-08 |
| ENSECAG00000014445 | -1.9788211 | 1.01E-10 | 1.76E-09 |
| ENSECAG00000007624 | -1.9789378 | 0.00068368 | 0.00226192 |
| ENSECAG00000051156 | -1.979508 | 0.00206139 | 0.00596655 |
| ENSECAG00000011940 | -1.9798769 | 1.61E-10 | 2.67E-09 |
| ENSECAG00000049498 | -1.9806124 | 0.00098455 | 0.00311669 |
| ENSECAG00000009998 | -1.9809722 | 0.0001388 | 0.00055896 |
| ENSECAG00000055416 | -1.9826933 | 9.14E-06 | 4.98E-05 |
| ENSECAG00000011973 | -1.9827022 | 0.00046073 | 0.00159917 |
| ENSECAG00000030484 | -1.9846467 | 3.23E-06 | 1.98E-05 |
| ENSECAG00000050151 | -1.9859676 | 2.27E-06 | 1.45E-05 |
| ENSECAG00000055241 | -1.98621 | 0.00029156 | 0.00107403 |
| ENSECAG00000007919 | -1.9864179 | 1.16E-21 | 1.20E-19 |
| ENSECAG00000053984 | -1.9865674 | 0.00474588 | 0.01232548 |
| ENSECAG00000011719 | -1.987008 | 1.00E-06 | 6.98E-06 |
| ENSECAG00000048625 | -1.9889294 | 0.01364107 | 0.03047014 |
| ENSECAG00000020198 | -1.9900739 | 0.00014285 | 0.00057402 |
| ENSECAG00000009935 | -1.9902409 | 0.00038618 | 0.00136892 |
| ENSECAG00000056223 | -1.9904869 | 0.00356195 | 0.00959211 |
| ENSECAG00000011653 | -1.9908382 | 2.68E-08 | 2.74E-07 |
| ENSECAG00000020657 | -1.9919789 | 5.84E-14 | 1.80E-12 |
| ENSECAG00000015077 | -1.9935589 | 0.00514779 | 0.01322062 |
| ENSECAG00000023578 | -1.9942075 | 0.00023999 | 0.00090458 |
| ENSECAG00000020710 | -1.9947202 | 6.58E-06 | 3.72E-05 |
| ENSECAG00000004515 | -1.996855 | 0.00495272 | 0.01278724 |
| ENSECAG00000041368 | -1.9975175 | 0.0001267 | 0.00051656 |
| ENSECAG00000007612 | -1.9976489 | 8.40E-06 | 4.62E-05 |
| ENSECAG00000051421 | -1.997754 | 0.00665409 | 0.01648291 |
| ENSECAG00000051294 | -1.9980655 | 0.00640121 | 0.01595646 |
| ENSECAG00000014649 | -1.9981191 | 0.01841594 | 0.03945189 |
| ENSECAG00000016056 | -1.9981599 | 5.27E-09 | 6.40E-08 |
| ENSECAG00000056339 | -1.9994709 | 1.46E-06 | 9.79E-06 |
| ENSECAG00000049583 | -2.0001959 | 0.00238401 | 0.00676827 |
| ENSECAG00000030006 | -2.0013617 | 1.23E-17 | 7.01E-16 |
| ENSECAG00000057219 | -2.0015891 | 0.00164861 | 0.00489631 |
| ENSECAG00000043986 | -2.0019439 | 0.00049175 | 0.00168979 |
| ENSECAG00000057509 | -2.0027473 | 1.46E-08 | 1.59E-07 |
| ENSECAG00000009700 | -2.0049416 | 1.47E-08 | 1.60E-07 |
| ENSECAG00000017982 | -2.0051876 | 0.01832537 | 0.03927669 |
| ENSECAG00000042229 | -2.005976 | 1.97E-06 | 1.27E-05 |
| ENSECAG00000020561 | -2.0071316 | 1.40E-09 | 1.94E-08 |
| ENSECAG00000011885 | -2.008036 | 1.03E-08 | 1.16E-07 |
| ENSECAG00000038109 | -2.0095409 | 0.01851335 | 0.03961577 |
| ENSECAG00000009553 | -2.0101667 | 8.89E-06 | 4.86E-05 |
| ENSECAG00000009418 | -2.0104719 | 8.73E-08 | 7.84E-07 |
| ENSECAG00000008881 | -2.0113492 | 8.00E-07 | 5.68E-06 |
| ENSECAG00000051898 | -2.0119546 | 0.01067913 | 0.02472397 |
| ENSECAG00000022564 | -2.012256 | 0.00420298 | 0.01106608 |
| ENSECAG00000015958 | -2.0130526 | 0.00018344 | 0.000714 |
| ENSECAG00000008392 | -2.0140678 | 0.00102769 | 0.0032404 |
| ENSECAG00000015134 | -2.0140757 | 4.92E-32 | 2.11E-29 |
| ENSECAG00000000267 | -2.0143889 | 0.01018127 | 0.02373116 |
| ENSECAG00000015613 | -2.0162677 | 4.62E-05 | 0.00021207 |
| ENSECAG00000013252 | -2.0169037 | 2.34E-14 | 7.66E-13 |
| ENSECAG00000044487 | -2.0171504 | 0.00070395 | 0.00231635 |
| ENSECAG00000006732 | -2.0179539 | 0.00161836 | 0.00481389 |
| ENSECAG00000045004 | -2.0191894 | 0.0027364 | 0.00762059 |
| ENSECAG00000056929 | -2.0216515 | 0.01058588 | 0.02454115 |
| ENSECAG00000022537 | -2.022857 | 0.00321886 | 0.00877286 |
| ENSECAG00000013677 | -2.0238305 | 5.68E-18 | 3.40E-16 |
| ENSECAG00000026664 | -2.0240272 | 0.00088082 | 0.00282881 |
| ENSECAG00000024302 | -2.0247112 | 0.00051074 | 0.00174857 |
| ENSECAG00000059097 | -2.0254911 | 1.83E-08 | 1.95E-07 |
| ENSECAG00000007024 | -2.0258186 | 5.19E-06 | 3.00E-05 |
| ENSECAG00000045242 | -2.0275367 | 1.01E-07 | 8.89E-07 |
| ENSECAG00000044771 | -2.0276599 | 0.00238313 | 0.00676749 |
| ENSECAG00000006996 | -2.0279468 | 0.00164465 | 0.00488716 |
| ENSECAG00000044037 | -2.0282977 | 0.01178257 | 0.02689685 |
| ENSECAG00000022981 | -2.0285927 | 7.12E-05 | 0.00031201 |
| ENSECAG00000030129 | -2.0288873 | 0.00042966 | 0.00150441 |
| ENSECAG00000010567 | -2.029794 | 0.00107099 | 0.0033511 |
| ENSECAG00000031779 | -2.0299047 | 2.07E-05 | 0.00010354 |
| ENSECAG00000058933 | -2.0323444 | 0.00755132 | 0.01835947 |
| ENSECAG00000030424 | -2.0331934 | 1.53E-05 | 7.89E-05 |
| ENSECAG00000024011 | -2.0337523 | 0.00943689 | 0.02224204 |
| ENSECAG00000059181 | -2.0348042 | 0.01939287 | 0.04118543 |
| ENSECAG00000049562 | -2.0355003 | 0.00672373 | 0.01662776 |
| ENSECAG00000022515 | -2.0355539 | 0.00047576 | 0.00164268 |
| ENSECAG00000048718 | -2.0357136 | 0.00273441 | 0.0076179 |
| ENSECAG00000049037 | -2.0358828 | 0.01204626 | 0.02737574 |
| ENSECAG00000049969 | -2.036418 | 0.00773954 | 0.01876041 |
| ENSECAG00000016582 | -2.0376438 | 8.42E-09 | 9.72E-08 |
| ENSECAG00000015671 | -2.0378832 | 0.00271015 | 0.00756068 |
| ENSECAG00000018907 | -2.0384139 | 0.00569145 | 0.01440657 |
| ENSECAG00000046830 | -2.0387073 | 0.00838684 | 0.02013887 |
| ENSECAG00000050794 | -2.0402606 | 3.24E-06 | 1.98E-05 |
| ENSECAG00000051079 | -2.0406116 | 0.00141601 | 0.00428988 |
| ENSECAG00000046707 | -2.0415381 | 0.00090738 | 0.00290326 |
| ENSECAG00000022624 | -2.041566 | 4.40E-06 | 2.59E-05 |
| ENSECAG00000021395 | -2.042274 | 1.28E-05 | 6.74E-05 |
| ENSECAG00000013363 | -2.0426869 | 9.80E-10 | 1.39E-08 |
| ENSECAG00000014625 | -2.0429473 | 0.00439625 | 0.01151955 |
| ENSECAG00000015509 | -2.0435175 | 5.26E-05 | 0.00023769 |
| ENSECAG00000055577 | -2.0458756 | 0.00024471 | 0.0009206 |
| ENSECAG00000023370 | -2.046419 | 0.00442486 | 0.01157821 |
| ENSECAG00000006609 | -2.046792 | 2.61E-12 | 5.95E-11 |
| ENSECAG00000044206 | -2.0474119 | 0.00020277 | 0.00078046 |
| ENSECAG00000010573 | -2.0491828 | 0.00018609 | 0.00072233 |
| ENSECAG00000037796 | -2.0494663 | 0.00225203 | 0.00645012 |
| ENSECAG00000016017 | -2.0500409 | 3.01E-10 | 4.74E-09 |
| ENSECAG00000047588 | -2.0503944 | 0.00156873 | 0.00468647 |
| ENSECAG00000020314 | -2.0507956 | 4.06E-08 | 3.95E-07 |
| ENSECAG00000007482 | -2.05081 | 0.00939365 | 0.02215991 |
| ENSECAG00000038617 | -2.0525999 | 0.00027478 | 0.00101946 |
| ENSECAG00000015892 | -2.0532109 | 2.34E-07 | 1.90E-06 |
| ENSECAG00000010194 | -2.0537392 | 1.92E-06 | 1.25E-05 |
| ENSECAG00000045951 | -2.0542047 | 0.00482825 | 0.01251081 |
| ENSECAG00000056678 | -2.0555511 | 4.94E-09 | 6.06E-08 |
| ENSECAG00000007471 | -2.0557663 | 0.00023808 | 0.00089835 |
| ENSECAG00000036921 | -2.0567741 | 7.83E-16 | 3.26E-14 |
| ENSECAG00000029611 | -2.0575042 | 1.45E-05 | 7.53E-05 |
| ENSECAG00000011962 | -2.0577628 | 4.49E-13 | 1.18E-11 |
| ENSECAG00000046753 | -2.0581642 | 0.00331717 | 0.00901769 |
| ENSECAG00000058446 | -2.0582081 | 0.01240191 | 0.02805842 |
| ENSECAG00000012975 | -2.0592841 | 0.01405203 | 0.03126 |
| ENSECAG00000009735 | -2.0598238 | 0.0127046 | 0.02863596 |
| ENSECAG00000042707 | -2.0602302 | 0.01238037 | 0.02801819 |
| ENSECAG00000006948 | -2.0627425 | 7.77E-10 | 1.12E-08 |
| ENSECAG00000003053 | -2.0631525 | 4.99E-07 | 3.75E-06 |
| ENSECAG00000035924 | -2.0637225 | 1.75E-05 | 8.91E-05 |
| ENSECAG00000021134 | -2.0641676 | 0.00061513 | 0.00206083 |
| ENSECAG00000037764 | -2.064263 | 0.00909667 | 0.02155384 |
| ENSECAG00000011932 | -2.064685 | 0.0018431 | 0.00540638 |
| ENSECAG00000050756 | -2.0647179 | 0.00822585 | 0.01980978 |
| ENSECAG00000019471 | -2.0650886 | 0.00012105 | 0.00049679 |
| ENSECAG00000049924 | -2.0662747 | 1.94E-05 | 9.77E-05 |
| ENSECAG00000024847 | -2.0663683 | 2.48E-08 | 2.56E-07 |
| ENSECAG00000045978 | -2.0664422 | 1.79E-08 | 1.92E-07 |
| ENSECAG00000006391 | -2.0668696 | 1.59E-09 | 2.19E-08 |
| ENSECAG00000000257 | -2.0669632 | 5.69E-05 | 0.0002552 |
| ENSECAG00000018730 | -2.0687018 | 0.01040769 | 0.02419835 |
| ENSECAG00000058965 | -2.0687523 | 0.00828871 | 0.01993536 |
| ENSECAG00000023080 | -2.0689962 | 2.24E-36 | 1.79E-33 |
| ENSECAG00000035440 | -2.069964 | 0.00512968 | 0.01317864 |
| ENSECAG00000044146 | -2.0700158 | 0.00061771 | 0.00206854 |
| ENSECAG00000027686 | -2.0702975 | 7.73E-05 | 0.00033627 |
| ENSECAG00000006192 | -2.0705376 | 9.88E-06 | 5.34E-05 |
| ENSECAG00000051730 | -2.0713783 | 0.01809997 | 0.03886814 |
| ENSECAG00000052751 | -2.0741897 | 0.00668696 | 0.01655514 |
| ENSECAG00000007684 | -2.0742392 | 1.67E-07 | 1.41E-06 |
| ENSECAG00000044784 | -2.0756161 | 3.09E-12 | 6.92E-11 |
| ENSECAG00000019553 | -2.0761386 | 2.37E-14 | 7.71E-13 |
| ENSECAG00000008354 | -2.0765508 | 3.60E-10 | 5.58E-09 |
| ENSECAG00000000740 | -2.0768948 | 0.0022298 | 0.00639464 |
| ENSECAG00000007511 | -2.077378 | 0.00089284 | 0.00286246 |
| ENSECAG00000022900 | -2.0775035 | 0.00020456 | 0.00078637 |
| ENSECAG00000047608 | -2.0790171 | 0.00907079 | 0.02150618 |
| ENSECAG00000050687 | -2.082103 | 0.00495388 | 0.01278781 |
| ENSECAG00000045766 | -2.0837434 | 0.00138909 | 0.00422092 |
| ENSECAG00000052128 | -2.0842094 | 0.00645817 | 0.016076 |
| ENSECAG00000012335 | -2.0848513 | 2.60E-06 | 1.63E-05 |
| ENSECAG00000048289 | -2.0858948 | 0.02179379 | 0.04548889 |
| ENSECAG00000009273 | -2.0859492 | 7.01E-07 | 5.06E-06 |
| ENSECAG00000050236 | -2.0860189 | 0.0042094 | 0.01108059 |
| ENSECAG00000051582 | -2.0871893 | 0.01687344 | 0.03656813 |
| ENSECAG00000015341 | -2.088366 | 4.45E-06 | 2.62E-05 |
| ENSECAG00000030952 | -2.0885605 | 0.00369526 | 0.00991168 |
| ENSECAG00000024269 | -2.0885822 | 1.48E-05 | 7.64E-05 |
| ENSECAG00000012789 | -2.090196 | 1.65E-06 | 1.09E-05 |
| ENSECAG00000036870 | -2.090333 | 0.00079915 | 0.00259485 |
| ENSECAG00000018933 | -2.0928192 | 0.0001165 | 0.00048074 |
| ENSECAG00000007409 | -2.0933401 | 0.00518217 | 0.01328784 |
| ENSECAG00000007701 | -2.0935377 | 0.00179166 | 0.0052728 |
| ENSECAG00000021691 | -2.0942108 | 0.00054663 | 0.00185694 |
| ENSECAG00000047907 | -2.0942836 | 0.01651594 | 0.0358978 |
| ENSECAG00000044569 | -2.0947931 | 0.00036969 | 0.00131778 |
| ENSECAG00000015854 | -2.0948061 | 8.99E-05 | 0.00038339 |
| ENSECAG00000045585 | -2.0951982 | 0.00227351 | 0.00650581 |
| ENSECAG00000050431 | -2.0954274 | 0.00395689 | 0.01052375 |
| ENSECAG00000007395 | -2.0964308 | 0.00027443 | 0.0010185 |
| ENSECAG00000042031 | -2.0984569 | 0.0013206 | 0.00403805 |
| ENSECAG00000035685 | -2.0989452 | 0.00390237 | 0.01039982 |
| ENSECAG00000009559 | -2.0991109 | 0.00908821 | 0.02153608 |
| ENSECAG00000052074 | -2.1004714 | 0.005223 | 0.01338299 |
| ENSECAG00000007548 | -2.1005114 | 0.00019986 | 0.00077115 |
| ENSECAG00000059330 | -2.1013827 | 0.00371147 | 0.00994921 |
| ENSECAG00000047032 | -2.1019871 | 6.01E-05 | 0.00026832 |
| ENSECAG00000026617 | -2.1020351 | 5.61E-08 | 5.27E-07 |
| ENSECAG00000011293 | -2.1027124 | 0.00040531 | 0.00142902 |
| ENSECAG00000012826 | -2.1030329 | 8.36E-11 | 1.48E-09 |
| ENSECAG00000023905 | -2.1035026 | 4.34E-18 | 2.63E-16 |
| ENSECAG00000059282 | -2.1039138 | 0.00344939 | 0.00932946 |
| ENSECAG00000052708 | -2.1048484 | 0.00250899 | 0.00707367 |
| ENSECAG00000044796 | -2.1066924 | 6.51E-05 | 0.00028834 |
| ENSECAG00000037803 | -2.107497 | 0.00110464 | 0.00344511 |
| ENSECAG00000017555 | -2.107603 | 3.81E-07 | 2.94E-06 |
| ENSECAG00000000506 | -2.1087732 | 1.14E-12 | 2.81E-11 |
| ENSECAG00000047716 | -2.1087838 | 0.0035206 | 0.00949794 |
| ENSECAG00000033359 | -2.1091173 | 0.0007834 | 0.00255038 |
| ENSECAG00000014332 | -2.1092925 | 8.17E-07 | 5.79E-06 |
| ENSECAG00000045148 | -2.1104763 | 0.01831138 | 0.03926144 |
| ENSECAG00000043202 | -2.1104974 | 0.01017199 | 0.0237219 |
| ENSECAG00000056041 | -2.1110999 | 4.26E-05 | 0.00019739 |
| ENSECAG00000008248 | -2.1114264 | 1.57E-12 | 3.74E-11 |
| ENSECAG00000059524 | -2.111631 | 0.00191371 | 0.00559077 |
| ENSECAG00000000784 | -2.1127858 | 0.00390903 | 0.01041382 |
| ENSECAG00000024611 | -2.1128414 | 1.99E-06 | 1.28E-05 |
| ENSECAG00000037408 | -2.1131649 | 0.01064969 | 0.02466861 |
| ENSECAG00000008177 | -2.113415 | 0.00017249 | 0.00067657 |
| ENSECAG00000029560 | -2.1137455 | 0.00031811 | 0.0011602 |
| ENSECAG00000025084 | -2.1139721 | 0.00985054 | 0.02307339 |
| ENSECAG00000043989 | -2.1141619 | 0.00044707 | 0.00155782 |
| ENSECAG00000015365 | -2.1145027 | 3.88E-10 | 5.97E-09 |
| ENSECAG00000039827 | -2.1146048 | 0.01648625 | 0.0358507 |
| ENSECAG00000022208 | -2.115338 | 0.00031507 | 0.00115003 |
| ENSECAG00000003841 | -2.1153489 | 0.01587181 | 0.03474107 |
| ENSECAG00000036383 | -2.1156858 | 0.01226985 | 0.02780751 |
| ENSECAG00000051942 | -2.1169181 | 0.00348969 | 0.00942363 |
| ENSECAG00000017284 | -2.117169 | 0.001019 | 0.0032171 |
| ENSECAG00000023018 | -2.1175182 | 0.00076073 | 0.00248381 |
| ENSECAG00000022328 | -2.119837 | 0.00869258 | 0.02074597 |
| ENSECAG00000000639 | -2.1200232 | 1.43E-05 | 7.44E-05 |
| ENSECAG00000013641 | -2.1201797 | 0.00084804 | 0.00273612 |
| ENSECAG00000036064 | -2.1205343 | 0.0004975 | 0.00170615 |
| ENSECAG00000045517 | -2.1215701 | 0.00377727 | 0.01010498 |
| ENSECAG00000023712 | -2.1218105 | 0.00319765 | 0.0087225 |
| ENSECAG00000012343 | -2.1226338 | 0.00185435 | 0.00543655 |
| ENSECAG00000019164 | -2.1227312 | 3.37E-08 | 3.35E-07 |
| ENSECAG00000007997 | -2.123925 | 0.000451 | 0.00156954 |
| ENSECAG00000009748 | -2.1247031 | 0.00071857 | 0.00235928 |
| ENSECAG00000002709 | -2.126482 | 0.00070225 | 0.00231146 |
| ENSECAG00000004220 | -2.1265729 | 0.00282119 | 0.00781773 |
| ENSECAG00000013807 | -2.1273429 | 2.22E-05 | 0.00010978 |
| ENSECAG00000030236 | -2.1281164 | 8.05E-05 | 0.00034809 |
| ENSECAG00000033916 | -2.1285269 | 0.01475649 | 0.03259996 |
| ENSECAG00000015934 | -2.1289731 | 3.59E-11 | 6.77E-10 |
| ENSECAG00000032860 | -2.1297597 | 4.36E-31 | 1.74E-28 |
| ENSECAG00000011848 | -2.1300058 | 6.43E-08 | 5.96E-07 |
| ENSECAG00000013347 | -2.1305403 | 0.00033531 | 0.001214 |
| ENSECAG00000009779 | -2.1313898 | 3.16E-07 | 2.49E-06 |
| ENSECAG00000016208 | -2.1353339 | 0.00175213 | 0.00517215 |
| ENSECAG00000019015 | -2.1353754 | 4.45E-07 | 3.38E-06 |
| ENSECAG00000017208 | -2.1353921 | 0.00065031 | 0.00216188 |
| ENSECAG00000022351 | -2.1359619 | 3.17E-08 | 3.17E-07 |
| ENSECAG00000041314 | -2.1368502 | 0.00102798 | 0.00324086 |
| ENSECAG00000035887 | -2.1373746 | 0.00035362 | 0.00127104 |
| ENSECAG00000048293 | -2.1390371 | 0.00019058 | 0.00073732 |
| ENSECAG00000055063 | -2.1391095 | 2.49E-05 | 0.00012215 |
| ENSECAG00000058844 | -2.1399335 | 8.06E-06 | 4.45E-05 |
| ENSECAG00000007899 | -2.1401346 | 0.00091071 | 0.002911 |
| ENSECAG00000008322 | -2.1401513 | 0.00471693 | 0.01225918 |
| ENSECAG00000040585 | -2.1401937 | 0.00715737 | 0.01754674 |
| ENSECAG00000021955 | -2.1406407 | 0.00990269 | 0.02317368 |
| ENSECAG00000024812 | -2.1418546 | 2.38E-07 | 1.93E-06 |
| ENSECAG00000043873 | -2.1421294 | 0.00373384 | 0.01000198 |
| ENSECAG00000023983 | -2.1424488 | 1.15E-06 | 7.89E-06 |
| ENSECAG00000025135 | -2.1430241 | 5.21E-06 | 3.01E-05 |
| ENSECAG00000056215 | -2.143134 | 2.10E-05 | 0.00010475 |
| ENSECAG00000011668 | -2.1454886 | 0.0002159 | 0.00082426 |
| ENSECAG00000059832 | -2.1472649 | 1.28E-05 | 6.73E-05 |
| ENSECAG00000034113 | -2.1502394 | 3.65E-07 | 2.83E-06 |
| ENSECAG00000004098 | -2.1503583 | 7.22E-09 | 8.52E-08 |
| ENSECAG00000029062 | -2.1503615 | 4.55E-15 | 1.69E-13 |
| ENSECAG00000011815 | -2.1507042 | 1.90E-05 | 9.60E-05 |
| ENSECAG00000058197 | -2.1513432 | 0.00052781 | 0.00180124 |
| ENSECAG00000034333 | -2.152213 | 0.01246226 | 0.02817322 |
| ENSECAG00000013274 | -2.1542665 | 6.56E-07 | 4.77E-06 |
| ENSECAG00000039619 | -2.1543483 | 2.27E-21 | 2.19E-19 |
| ENSECAG00000035761 | -2.1547184 | 0.00121304 | 0.00374141 |
| ENSECAG00000059497 | -2.155122 | 0.00328281 | 0.00892972 |
| ENSECAG00000014030 | -2.1570793 | 3.98E-14 | 1.26E-12 |
| ENSECAG00000011484 | -2.1571682 | 0.00185468 | 0.00543679 |
| ENSECAG00000014152 | -2.157304 | 7.12E-07 | 5.13E-06 |
| ENSECAG00000053629 | -2.1574978 | 0.00070714 | 0.00232617 |
| ENSECAG00000051120 | -2.1584247 | 0.00039117 | 0.00138518 |
| ENSECAG00000050446 | -2.1614622 | 0.0105194 | 0.02441997 |
| ENSECAG00000000202 | -2.1616955 | 0.00999693 | 0.02335998 |
| ENSECAG00000005796 | -2.1632308 | 9.96E-05 | 0.00041972 |
| ENSECAG00000035543 | -2.1637705 | 4.99E-09 | 6.11E-08 |
| ENSECAG00000019664 | -2.1654185 | 6.13E-31 | 2.36E-28 |
| ENSECAG00000028845 | -2.165553 | 0.00220175 | 0.00632394 |
| ENSECAG00000012116 | -2.1672473 | 4.16E-09 | 5.18E-08 |
| ENSECAG00000050266 | -2.1677739 | 0.00068499 | 0.00226514 |
| ENSECAG00000010626 | -2.1680332 | 0.0103759 | 0.024142 |
| ENSECAG00000048673 | -2.1683984 | 5.73E-11 | 1.04E-09 |
| ENSECAG00000059593 | -2.1691231 | 0.01824712 | 0.03913524 |
| ENSECAG00000003388 | -2.1692139 | 4.07E-06 | 2.42E-05 |
| ENSECAG00000051690 | -2.1695058 | 0.00263382 | 0.00738091 |
| ENSECAG00000001142 | -2.1695226 | 0.00218015 | 0.00626675 |
| ENSECAG00000020443 | -2.1699549 | 0.00502349 | 0.01293707 |
| ENSECAG00000052323 | -2.1701206 | 1.06E-06 | 7.31E-06 |
| ENSECAG00000015436 | -2.1704078 | 0.00133529 | 0.00407532 |
| ENSECAG00000001371 | -2.1729143 | 2.68E-08 | 2.74E-07 |
| ENSECAG00000022399 | -2.1730029 | 4.32E-05 | 0.00019981 |
| ENSECAG00000003311 | -2.1732815 | 6.57E-05 | 0.00029062 |
| ENSECAG00000019819 | -2.1733518 | 1.01E-08 | 1.14E-07 |
| ENSECAG00000058011 | -2.1739054 | 1.89E-10 | 3.09E-09 |
| ENSECAG00000012440 | -2.1740162 | 0.0002558 | 0.00095839 |
| ENSECAG00000058754 | -2.1741253 | 1.05E-07 | 9.30E-07 |
| ENSECAG00000023874 | -2.1742271 | 2.19E-05 | 0.000109 |
| ENSECAG00000022433 | -2.1742284 | 2.40E-06 | 1.52E-05 |
| ENSECAG00000012558 | -2.1762401 | 0.0033849 | 0.00918395 |
| ENSECAG00000048284 | -2.1764207 | 0.00216243 | 0.00622221 |
| ENSECAG00000057597 | -2.1768243 | 0.00073589 | 0.00241047 |
| ENSECAG00000060312 | -2.1771049 | 0.00419062 | 0.01103897 |
| ENSECAG00000021292 | -2.1773574 | 0.01319581 | 0.0296266 |
| ENSECAG00000021670 | -2.1782615 | 5.09E-14 | 1.59E-12 |
| ENSECAG00000025078 | -2.1783716 | 6.40E-06 | 3.62E-05 |
| ENSECAG00000016663 | -2.1807272 | 1.05E-07 | 9.28E-07 |
| ENSECAG00000022950 | -2.1807444 | 1.44E-14 | 4.87E-13 |
| ENSECAG00000016627 | -2.1814617 | 4.67E-22 | 4.99E-20 |
| ENSECAG00000024186 | -2.1821297 | 2.27E-06 | 1.45E-05 |
| ENSECAG00000012358 | -2.1822039 | 0.00193333 | 0.00564144 |
| ENSECAG00000010028 | -2.1822287 | 0.00039528 | 0.00139718 |
| ENSECAG00000008360 | -2.1827484 | 6.63E-19 | 4.64E-17 |
| ENSECAG00000000874 | -2.183443 | 7.93E-07 | 5.64E-06 |
| ENSECAG00000049032 | -2.1863383 | 1.84E-06 | 1.20E-05 |
| ENSECAG00000014408 | -2.1866367 | 0.00010734 | 0.00044826 |
| ENSECAG00000044509 | -2.1869978 | 7.10E-05 | 0.00031102 |
| ENSECAG00000058407 | -2.1892058 | 0.00856724 | 0.02049936 |
| ENSECAG00000057553 | -2.1893838 | 0.01166302 | 0.02667813 |
| ENSECAG00000014935 | -2.1901595 | 9.78E-08 | 8.68E-07 |
| ENSECAG00000001643 | -2.1918147 | 7.54E-06 | 4.19E-05 |
| ENSECAG00000057036 | -2.1937016 | 0.002236 | 0.0064108 |
| ENSECAG00000023424 | -2.194624 | 0.00302639 | 0.00831237 |
| ENSECAG00000051550 | -2.1953589 | 0.00971236 | 0.02279992 |
| ENSECAG00000033759 | -2.1954158 | 0.00122094 | 0.00376372 |
| ENSECAG00000009954 | -2.1960262 | 0.00142631 | 0.00431697 |
| ENSECAG00000021664 | -2.1972876 | 4.08E-10 | 6.24E-09 |
| ENSECAG00000015345 | -2.1978378 | 7.06E-14 | 2.14E-12 |
| ENSECAG00000011620 | -2.1978807 | 3.72E-07 | 2.88E-06 |
| ENSECAG00000060073 | -2.1983695 | 0.00179993 | 0.00529505 |
| ENSECAG00000014557 | -2.1984023 | 1.37E-35 | 9.31E-33 |
| ENSECAG00000054088 | -2.1990866 | 6.15E-08 | 5.73E-07 |
| ENSECAG00000013494 | -2.2000143 | 7.81E-05 | 0.00033892 |
| ENSECAG00000024828 | -2.2000332 | 5.69E-09 | 6.88E-08 |
| ENSECAG00000027699 | -2.2021859 | 8.25E-15 | 2.93E-13 |
| ENSECAG00000008204 | -2.2025805 | 1.64E-05 | 8.37E-05 |
| ENSECAG00000014742 | -2.2026243 | 2.96E-09 | 3.82E-08 |
| ENSECAG00000001205 | -2.2028069 | 2.37E-05 | 0.00011677 |
| ENSECAG00000020972 | -2.202807 | 5.31E-06 | 3.06E-05 |
| ENSECAG00000049755 | -2.2046877 | 3.03E-06 | 1.87E-05 |
| ENSECAG00000004707 | -2.2056591 | 2.76E-10 | 4.37E-09 |
| ENSECAG00000050599 | -2.2058151 | 0.00080774 | 0.00262007 |
| ENSECAG00000020393 | -2.2059272 | 8.34E-19 | 5.70E-17 |
| ENSECAG00000009575 | -2.2073872 | 0.00996105 | 0.02328831 |
| ENSECAG00000053320 | -2.2075404 | 0.00170183 | 0.00504029 |
| ENSECAG00000030748 | -2.2081458 | 0.00062119 | 0.00207988 |
| ENSECAG00000008335 | -2.2083446 | 2.28E-07 | 1.86E-06 |
| ENSECAG00000020108 | -2.2095337 | 1.54E-05 | 7.93E-05 |
| ENSECAG00000025597 | -2.2096355 | 0.00034763 | 0.00125292 |
| ENSECAG00000003802 | -2.2104496 | 1.43E-06 | 9.60E-06 |
| ENSECAG00000022605 | -2.2108281 | 0.00027227 | 0.00101132 |
| ENSECAG00000022044 | -2.2112591 | 9.87E-09 | 1.12E-07 |
| ENSECAG00000000538 | -2.2112599 | 0.00035394 | 0.00127156 |
| ENSECAG00000055789 | -2.2124135 | 4.44E-08 | 4.29E-07 |
| ENSECAG00000001197 | -2.2135749 | 6.60E-07 | 4.80E-06 |
| ENSECAG00000047207 | -2.2137997 | 3.17E-10 | 4.98E-09 |
| ENSECAG00000024597 | -2.2144823 | 0.00371349 | 0.00995222 |
| ENSECAG00000013026 | -2.214749 | 7.81E-08 | 7.10E-07 |
| ENSECAG00000019176 | -2.2156357 | 0.00221223 | 0.00635078 |
| ENSECAG00000033282 | -2.2162018 | 0.00639968 | 0.01595528 |
| ENSECAG00000005681 | -2.2164178 | 3.54E-09 | 4.47E-08 |
| ENSECAG00000015475 | -2.2164937 | 0.001455 | 0.00439371 |
| ENSECAG00000013931 | -2.2178956 | 0.00058432 | 0.00196882 |
| ENSECAG00000013036 | -2.2182428 | 6.54E-18 | 3.87E-16 |
| ENSECAG00000011349 | -2.2194089 | 0.00042644 | 0.00149502 |
| ENSECAG00000047267 | -2.2209605 | 3.91E-06 | 2.34E-05 |
| ENSECAG00000001276 | -2.2211534 | 0.0029103 | 0.00803778 |
| ENSECAG00000020215 | -2.2219152 | 5.07E-06 | 2.94E-05 |
| ENSECAG00000022969 | -2.2225569 | 0.00334605 | 0.00909178 |
| ENSECAG00000011351 | -2.2225752 | 2.84E-05 | 0.00013703 |
| ENSECAG00000049181 | -2.223832 | 1.42E-10 | 2.38E-09 |
| ENSECAG00000012311 | -2.224016 | 0.00140653 | 0.00426695 |
| ENSECAG00000029103 | -2.2252741 | 0.01207053 | 0.02741975 |
| ENSECAG00000024624 | -2.2257407 | 0.00018325 | 0.00071339 |
| ENSECAG00000014041 | -2.2267652 | 0.00244378 | 0.00691075 |
| ENSECAG00000010917 | -2.2285896 | 2.58E-07 | 2.08E-06 |
| ENSECAG00000043214 | -2.2295768 | 9.18E-07 | 6.44E-06 |
| ENSECAG00000048140 | -2.2299329 | 3.79E-09 | 4.76E-08 |
| ENSECAG00000045261 | -2.2300359 | 1.08E-05 | 5.78E-05 |
| ENSECAG00000032640 | -2.2302608 | 0.00297227 | 0.00818464 |
| ENSECAG00000011734 | -2.2303997 | 7.02E-09 | 8.29E-08 |
| ENSECAG00000019807 | -2.2305967 | 0.00455251 | 0.01187744 |
| ENSECAG00000015003 | -2.2307403 | 1.68E-05 | 8.56E-05 |
| ENSECAG00000031671 | -2.2318058 | 0.00486684 | 0.01259751 |
| ENSECAG00000014993 | -2.2323955 | 0.0003111 | 0.00113722 |
| ENSECAG00000020232 | -2.2329119 | 0.00442044 | 0.01157071 |
| ENSECAG00000014812 | -2.2334908 | 0.0013669 | 0.00416198 |
| ENSECAG00000046590 | -2.2348633 | 0.00237806 | 0.0067574 |
| ENSECAG00000055172 | -2.2357656 | 0.00167422 | 0.00496708 |
| ENSECAG00000022941 | -2.2358747 | 0.00018504 | 0.00071898 |
| ENSECAG00000020294 | -2.2363778 | 1.96E-12 | 4.61E-11 |
| ENSECAG00000014698 | -2.2365792 | 1.02E-11 | 2.10E-10 |
| ENSECAG00000024467 | -2.2384957 | 0.00191792 | 0.00560159 |
| ENSECAG00000010465 | -2.2385439 | 2.40E-05 | 0.00011784 |
| ENSECAG00000019638 | -2.2399818 | 3.39E-08 | 3.37E-07 |
| ENSECAG00000047762 | -2.2401654 | 0.00035183 | 0.00126562 |
| ENSECAG00000050954 | -2.2422441 | 0.01921741 | 0.04086717 |
| ENSECAG00000007512 | -2.2432559 | 0.00060932 | 0.00204353 |
| ENSECAG00000022135 | -2.2435965 | 0.00537837 | 0.01373387 |
| ENSECAG00000048512 | -2.2443388 | 0.00157527 | 0.00470414 |
| ENSECAG00000023252 | -2.2446638 | 7.80E-07 | 5.56E-06 |
| ENSECAG00000036730 | -2.2458579 | 0.00915015 | 0.02165529 |
| ENSECAG00000012482 | -2.2479898 | 0.00062275 | 0.00208417 |
| ENSECAG00000028788 | -2.2479978 | 1.98E-27 | 4.60E-25 |
| ENSECAG00000053295 | -2.2482639 | 0.00933357 | 0.02202872 |
| ENSECAG00000009101 | -2.2529207 | 6.27E-13 | 1.61E-11 |
| ENSECAG00000050662 | -2.2551665 | 0.00017407 | 0.00068217 |
| ENSECAG00000000515 | -2.2553337 | 0.00018141 | 0.0007071 |
| ENSECAG00000012282 | -2.2576512 | 5.08E-10 | 7.65E-09 |
| ENSECAG00000001347 | -2.2585094 | 3.55E-06 | 2.15E-05 |
| ENSECAG00000059894 | -2.258791 | 0.01108897 | 0.02553526 |
| ENSECAG00000030869 | -2.2589243 | 1.62E-12 | 3.84E-11 |
| ENSECAG00000022387 | -2.2590532 | 7.95E-17 | 3.85E-15 |
| ENSECAG00000053311 | -2.2593488 | 0.00149174 | 0.00448705 |
| ENSECAG00000020254 | -2.259532 | 0.00088819 | 0.00285002 |
| ENSECAG00000032134 | -2.2598571 | 0.00509389 | 0.01310166 |
| ENSECAG00000052740 | -2.260076 | 0.00011821 | 0.00048726 |
| ENSECAG00000025267 | -2.2604928 | 0.0030483 | 0.00836172 |
| ENSECAG00000057675 | -2.261251 | 1.49E-07 | 1.28E-06 |
| ENSECAG00000018082 | -2.265203 | 1.27E-05 | 6.66E-05 |
| ENSECAG00000042632 | -2.2655661 | 1.98E-18 | 1.26E-16 |
| ENSECAG00000024845 | -2.2656634 | 3.51E-05 | 0.00016567 |
| ENSECAG00000017045 | -2.2677181 | 1.27E-18 | 8.39E-17 |
| ENSECAG00000043417 | -2.2705218 | 1.40E-06 | 9.43E-06 |
| ENSECAG00000026456 | -2.271181 | 3.46E-06 | 2.10E-05 |
| ENSECAG00000006591 | -2.2722814 | 1.44E-05 | 7.47E-05 |
| ENSECAG00000008154 | -2.2723989 | 5.94E-07 | 4.37E-06 |
| ENSECAG00000046029 | -2.2725993 | 0.00366378 | 0.00983907 |
| ENSECAG00000059271 | -2.2728409 | 0.0002187 | 0.00083354 |
| ENSECAG00000000280 | -2.2729432 | 0.00012294 | 0.00050341 |
| ENSECAG00000040618 | -2.2734805 | 0.00594776 | 0.01496551 |
| ENSECAG00000013037 | -2.2751679 | 2.28E-07 | 1.86E-06 |
| ENSECAG00000044012 | -2.2763233 | 0.00017198 | 0.00067494 |
| ENSECAG00000002519 | -2.2767686 | 0.00071517 | 0.00234915 |
| ENSECAG00000000662 | -2.2768176 | 0.00059761 | 0.00200876 |
| ENSECAG00000052153 | -2.2768925 | 0.00253235 | 0.00713323 |
| ENSECAG00000049837 | -2.2779897 | 0.00045362 | 0.00157768 |
| ENSECAG00000013567 | -2.2794108 | 2.39E-05 | 0.00011772 |
| ENSECAG00000055174 | -2.2802213 | 0.01042669 | 0.02423748 |
| ENSECAG00000000960 | -2.2814664 | 1.07E-11 | 2.20E-10 |
| ENSECAG00000047803 | -2.2824723 | 0.00719223 | 0.01762254 |
| ENSECAG00000020272 | -2.282971 | 4.91E-05 | 0.00022412 |
| ENSECAG00000027666 | -2.284472 | 1.04E-13 | 3.01E-12 |
| ENSECAG00000017125 | -2.2846677 | 6.99E-05 | 0.00030701 |
| ENSECAG00000045541 | -2.2860546 | 0.00013044 | 0.00052957 |
| ENSECAG00000008726 | -2.2864662 | 0.00076006 | 0.00248201 |
| ENSECAG00000012520 | -2.2866914 | 4.57E-09 | 5.66E-08 |
| ENSECAG00000045088 | -2.2869915 | 0.02152892 | 0.04503279 |
| ENSECAG00000021758 | -2.2873975 | 0.00142802 | 0.00432042 |
| ENSECAG00000021360 | -2.2881936 | 7.34E-18 | 4.32E-16 |
| ENSECAG00000053290 | -2.2908444 | 0.00012604 | 0.00051426 |
| ENSECAG00000015240 | -2.2911256 | 0.00088874 | 0.00285095 |
| ENSECAG00000055643 | -2.2918353 | 0.00104339 | 0.00328111 |
| ENSECAG00000008870 | -2.2927251 | 1.04E-06 | 7.19E-06 |
| ENSECAG00000059273 | -2.2938152 | 0.00270486 | 0.00754781 |
| ENSECAG00000012719 | -2.294574 | 0.00167943 | 0.00498189 |
| ENSECAG00000004242 | -2.294886 | 0.00032973 | 0.00119573 |
| ENSECAG00000015548 | -2.296911 | 2.98E-07 | 2.36E-06 |
| ENSECAG00000054363 | -2.2978226 | 4.60E-07 | 3.48E-06 |
| ENSECAG00000049148 | -2.2987014 | 0.00140409 | 0.0042607 |
| ENSECAG00000051383 | -2.2988858 | 1.07E-06 | 7.41E-06 |
| ENSECAG00000021413 | -2.2994322 | 2.99E-08 | 3.02E-07 |
| ENSECAG00000010895 | -2.3004135 | 1.80E-05 | 9.10E-05 |
| ENSECAG00000004666 | -2.3014673 | 1.21E-05 | 6.41E-05 |
| ENSECAG00000016625 | -2.3015476 | 3.49E-07 | 2.72E-06 |
| ENSECAG00000036703 | -2.3037815 | 0.01093509 | 0.02522249 |
| ENSECAG00000019267 | -2.3039173 | 0.00284843 | 0.00788347 |
| ENSECAG00000049001 | -2.3059175 | 0.00044383 | 0.00154774 |
| ENSECAG00000031841 | -2.3074848 | 1.68E-06 | 1.11E-05 |
| ENSECAG00000026836 | -2.3084295 | 0.00044518 | 0.00155169 |
| ENSECAG00000016111 | -2.3106519 | 4.32E-10 | 6.57E-09 |
| ENSECAG00000049080 | -2.311259 | 0.00016649 | 0.00065616 |
| ENSECAG00000049779 | -2.3129459 | 2.06E-14 | 6.79E-13 |
| ENSECAG00000052431 | -2.3137783 | 1.38E-08 | 1.51E-07 |
| ENSECAG00000040037 | -2.31598 | 0.00881396 | 0.02098187 |
| ENSECAG00000006074 | -2.3161098 | 0.00087274 | 0.00280689 |
| ENSECAG00000055431 | -2.3162846 | 0.00104745 | 0.00329157 |
| ENSECAG00000040433 | -2.3167905 | 0.01776827 | 0.0382551 |
| ENSECAG00000013465 | -2.3220414 | 0.00681223 | 0.01681127 |
| ENSECAG00000046404 | -2.3227907 | 0.00344925 | 0.00932946 |
| ENSECAG00000038630 | -2.3232675 | 0.00014931 | 0.00059685 |
| ENSECAG00000051260 | -2.3235603 | 0.01148054 | 0.02630954 |
| ENSECAG00000023606 | -2.3244585 | 0.00836978 | 0.02010438 |
| ENSECAG00000045691 | -2.3257274 | 0.01472991 | 0.0325619 |
| ENSECAG00000006756 | -2.3271899 | 0.00012133 | 0.00049785 |
| ENSECAG00000054579 | -2.3276089 | 0.00057658 | 0.00194685 |
| ENSECAG00000057142 | -2.3277927 | 1.19E-21 | 1.22E-19 |
| ENSECAG00000000310 | -2.32914 | 0.00010538 | 0.00044131 |
| ENSECAG00000014521 | -2.3293669 | 1.50E-06 | 9.99E-06 |
| ENSECAG00000013290 | -2.3309876 | 6.61E-05 | 0.00029197 |
| ENSECAG00000017737 | -2.3312712 | 8.10E-05 | 0.00034956 |
| ENSECAG00000001988 | -2.3346619 | 7.66E-08 | 6.98E-07 |
| ENSECAG00000024405 | -2.3417479 | 2.60E-05 | 0.00012684 |
| ENSECAG00000017825 | -2.3425422 | 3.49E-06 | 2.11E-05 |
| ENSECAG00000018814 | -2.3429111 | 2.68E-10 | 4.28E-09 |
| ENSECAG00000022241 | -2.3429343 | 8.45E-13 | 2.11E-11 |
| ENSECAG00000034476 | -2.3443549 | 4.02E-05 | 0.00018723 |
| ENSECAG00000035300 | -2.3449646 | 5.40E-11 | 9.87E-10 |
| ENSECAG00000057320 | -2.3464364 | 0.00018538 | 0.00072019 |
| ENSECAG00000013039 | -2.3465918 | 3.86E-10 | 5.95E-09 |
| ENSECAG00000049137 | -2.3479864 | 0.00033686 | 0.00121923 |
| ENSECAG00000013082 | -2.3480884 | 0.02043916 | 0.04312468 |
| ENSECAG00000030595 | -2.3488103 | 0.00187161 | 0.00548066 |
| ENSECAG00000011908 | -2.3493754 | 0.00580378 | 0.01465439 |
| ENSECAG00000017774 | -2.3500836 | 0.00091743 | 0.00292996 |
| ENSECAG00000014225 | -2.3517455 | 1.22E-10 | 2.09E-09 |
| ENSECAG00000046948 | -2.3522901 | 0.00396939 | 0.01054948 |
| ENSECAG00000017658 | -2.3525538 | 0.00269181 | 0.00751515 |
| ENSECAG00000020732 | -2.352797 | 0.00026375 | 0.00098488 |
| ENSECAG00000048944 | -2.355296 | 2.39E-07 | 1.93E-06 |
| ENSECAG00000009385 | -2.3558783 | 1.01E-05 | 5.45E-05 |
| ENSECAG00000010926 | -2.3559515 | 0.00077892 | 0.00253695 |
| ENSECAG00000020556 | -2.3560813 | 1.94E-08 | 2.06E-07 |
| ENSECAG00000011256 | -2.3567696 | 3.14E-08 | 3.14E-07 |
| ENSECAG00000017074 | -2.3568536 | 3.52E-17 | 1.83E-15 |
| ENSECAG00000027748 | -2.3575598 | 0.01246995 | 0.0281857 |
| ENSECAG00000014683 | -2.3604185 | 0.00038368 | 0.00136184 |
| ENSECAG00000014766 | -2.3623715 | 6.85E-20 | 5.56E-18 |
| ENSECAG00000046304 | -2.362432 | 1.20E-07 | 1.04E-06 |
| ENSECAG00000020101 | -2.3626198 | 0.01514398 | 0.03334392 |
| ENSECAG00000019732 | -2.3626582 | 5.08E-06 | 2.95E-05 |
| ENSECAG00000014200 | -2.3644158 | 6.05E-09 | 7.24E-08 |
| ENSECAG00000047034 | -2.364961 | 0.00266597 | 0.00746007 |
| ENSECAG00000052182 | -2.366273 | 0.00107696 | 0.00336772 |
| ENSECAG00000058198 | -2.3663643 | 0.00030866 | 0.00112942 |
| ENSECAG00000025101 | -2.3668277 | 2.17E-06 | 1.39E-05 |
| ENSECAG00000044347 | -2.373066 | 0.0002618 | 0.0009786 |
| ENSECAG00000016485 | -2.3730997 | 2.37E-05 | 0.00011695 |
| ENSECAG00000009100 | -2.3733723 | 2.06E-07 | 1.70E-06 |
| ENSECAG00000017155 | -2.3744457 | 3.16E-05 | 0.00015103 |
| ENSECAG00000053639 | -2.3744617 | 0.0113282 | 0.02601645 |
| ENSECAG00000050391 | -2.3744762 | 0.00618992 | 0.01549629 |
| ENSECAG00000008224 | -2.3765608 | 0.00034437 | 0.00124244 |
| ENSECAG00000056888 | -2.3767648 | 3.32E-05 | 0.00015786 |
| ENSECAG00000021262 | -2.3768293 | 0.00234791 | 0.00668605 |
| ENSECAG00000016477 | -2.376893 | 0.00235548 | 0.00670174 |
| ENSECAG00000037472 | -2.3777616 | 1.11E-05 | 5.91E-05 |
| ENSECAG00000032014 | -2.3785827 | 0.00017904 | 0.00069865 |
| ENSECAG00000011358 | -2.3790044 | 6.66E-10 | 9.82E-09 |
| ENSECAG00000044511 | -2.3791223 | 0.00020447 | 0.00078615 |
| ENSECAG00000058848 | -2.379712 | 0.00071241 | 0.00234076 |
| ENSECAG00000055666 | -2.3800832 | 4.39E-06 | 2.59E-05 |
| ENSECAG00000022865 | -2.3809044 | 0.00182316 | 0.00535636 |
| ENSECAG00000002425 | -2.3812315 | 5.76E-09 | 6.96E-08 |
| ENSECAG00000023387 | -2.3841755 | 2.03E-05 | 0.00010149 |
| ENSECAG00000046811 | -2.3842969 | 0.00127593 | 0.00391272 |
| ENSECAG00000053575 | -2.3848016 | 0.00309701 | 0.00846864 |
| ENSECAG00000046064 | -2.3853769 | 1.14E-06 | 7.83E-06 |
| ENSECAG00000019356 | -2.3856828 | 0.00880785 | 0.02097403 |
| ENSECAG00000031859 | -2.3859147 | 0.00016357 | 0.00064611 |
| ENSECAG00000023128 | -2.3870541 | 9.50E-09 | 1.08E-07 |
| ENSECAG00000020093 | -2.387379 | 3.53E-16 | 1.54E-14 |
| ENSECAG00000020301 | -2.3891861 | 1.20E-12 | 2.92E-11 |
| ENSECAG00000057254 | -2.3905424 | 0.00082598 | 0.00267422 |
| ENSECAG00000007435 | -2.3913465 | 0.00038216 | 0.00135769 |
| ENSECAG00000020585 | -2.3916536 | 7.76E-06 | 4.30E-05 |
| ENSECAG00000010280 | -2.3918927 | 0.00028545 | 0.00105467 |
| ENSECAG00000000509 | -2.3927852 | 1.02E-13 | 2.97E-12 |
| ENSECAG00000044926 | -2.3932053 | 8.19E-05 | 0.0003532 |
| ENSECAG00000040463 | -2.393362 | 2.57E-05 | 0.00012569 |
| ENSECAG00000014681 | -2.3934828 | 2.69E-18 | 1.68E-16 |
| ENSECAG00000030532 | -2.3940094 | 0.00337274 | 0.0091554 |
| ENSECAG00000038004 | -2.3945949 | 0.00026997 | 0.0010041 |
| ENSECAG00000010747 | -2.3951802 | 5.05E-06 | 2.93E-05 |
| ENSECAG00000020769 | -2.396584 | 2.05E-05 | 0.0001027 |
| ENSECAG00000002917 | -2.3966962 | 1.75E-07 | 1.47E-06 |
| ENSECAG00000030283 | -2.3967951 | 0.01111678 | 0.02558875 |
| ENSECAG00000022875 | -2.397442 | 4.57E-08 | 4.39E-07 |
| ENSECAG00000011987 | -2.3982489 | 1.56E-42 | 2.50E-39 |
| ENSECAG00000048487 | -2.3991622 | 0.00053497 | 0.00182176 |
| ENSECAG00000054060 | -2.3998301 | 0.00185518 | 0.00543683 |
| ENSECAG00000003151 | -2.4001544 | 0.0001476 | 0.00059098 |
| ENSECAG00000059293 | -2.4002501 | 0.00035888 | 0.00128699 |
| ENSECAG00000023174 | -2.4005617 | 6.02E-17 | 3.01E-15 |
| ENSECAG00000017000 | -2.4014425 | 2.60E-09 | 3.41E-08 |
| ENSECAG00000013681 | -2.4016643 | 0.00057783 | 0.00195017 |
| ENSECAG00000021854 | -2.4020412 | 3.85E-07 | 2.96E-06 |
| ENSECAG00000018511 | -2.4030172 | 8.19E-23 | 9.49E-21 |
| ENSECAG00000012155 | -2.4037084 | 1.91E-26 | 3.94E-24 |
| ENSECAG00000014483 | -2.4038113 | 0.00192803 | 0.00562818 |
| ENSECAG00000047017 | -2.4043575 | 1.98E-05 | 9.92E-05 |
| ENSECAG00000027835 | -2.4054693 | 4.17E-05 | 0.00019369 |
| ENSECAG00000018254 | -2.4061437 | 9.94E-14 | 2.91E-12 |
| ENSECAG00000054324 | -2.4068242 | 6.51E-07 | 4.74E-06 |
| ENSECAG00000016934 | -2.4080623 | 1.73E-06 | 1.14E-05 |
| ENSECAG00000022188 | -2.4080997 | 9.54E-08 | 8.48E-07 |
| ENSECAG00000006058 | -2.4085059 | 1.63E-05 | 8.35E-05 |
| ENSECAG00000008933 | -2.408746 | 2.35E-07 | 1.91E-06 |
| ENSECAG00000020557 | -2.4096484 | 2.31E-09 | 3.06E-08 |
| ENSECAG00000029387 | -2.4099449 | 0.00024254 | 0.00091304 |
| ENSECAG00000017150 | -2.4101132 | 1.26E-05 | 6.62E-05 |
| ENSECAG00000013932 | -2.4102896 | 0.0010298 | 0.00324488 |
| ENSECAG00000021113 | -2.4109436 | 5.65E-07 | 4.18E-06 |
| ENSECAG00000009319 | -2.4121915 | 0.00254122 | 0.0071564 |
| ENSECAG00000011735 | -2.4132874 | 3.16E-15 | 1.21E-13 |
| ENSECAG00000024574 | -2.4137258 | 1.40E-10 | 2.36E-09 |
| ENSECAG00000016756 | -2.4140139 | 5.63E-12 | 1.21E-10 |
| ENSECAG00000018656 | -2.4148601 | 7.53E-08 | 6.87E-07 |
| ENSECAG00000013748 | -2.4153986 | 0.00728204 | 0.01780551 |
| ENSECAG00000029529 | -2.4155468 | 1.42E-10 | 2.38E-09 |
| ENSECAG00000023763 | -2.4164495 | 5.52E-08 | 5.20E-07 |
| ENSECAG00000019461 | -2.4170831 | 3.44E-07 | 2.69E-06 |
| ENSECAG00000013682 | -2.4178305 | 0.00072165 | 0.00236765 |
| ENSECAG00000044956 | -2.418187 | 1.56E-12 | 3.71E-11 |
| ENSECAG00000051127 | -2.4185894 | 0.02073193 | 0.04364349 |
| ENSECAG00000022370 | -2.4193459 | 4.12E-06 | 2.45E-05 |
| ENSECAG00000024688 | -2.4237522 | 0.003693 | 0.00990801 |
| ENSECAG00000011919 | -2.4239597 | 5.37E-06 | 3.10E-05 |
| ENSECAG00000012823 | -2.4242825 | 1.74E-05 | 8.84E-05 |
| ENSECAG00000059760 | -2.4248987 | 1.63E-11 | 3.27E-10 |
| ENSECAG00000021252 | -2.4249546 | 0.00153282 | 0.0045964 |
| ENSECAG00000044691 | -2.4251788 | 2.82E-10 | 4.47E-09 |
| ENSECAG00000035443 | -2.4256924 | 0.00124697 | 0.00383391 |
| ENSECAG00000056759 | -2.4292851 | 1.37E-10 | 2.32E-09 |
| ENSECAG00000013862 | -2.4309906 | 6.33E-16 | 2.66E-14 |
| ENSECAG00000003318 | -2.4312045 | 7.62E-05 | 0.00033173 |
| ENSECAG00000038420 | -2.4315687 | 1.52E-05 | 7.82E-05 |
| ENSECAG00000021602 | -2.4325202 | 7.63E-18 | 4.46E-16 |
| ENSECAG00000054587 | -2.4339587 | 0.01967295 | 0.04169702 |
| ENSECAG00000012643 | -2.434518 | 0.01905533 | 0.04059589 |
| ENSECAG00000009135 | -2.4360844 | 4.27E-12 | 9.35E-11 |
| ENSECAG00000053266 | -2.4387299 | 0.00190692 | 0.00557238 |
| ENSECAG00000057542 | -2.4392903 | 4.15E-06 | 2.47E-05 |
| ENSECAG00000024484 | -2.4401884 | 0.00428453 | 0.01126245 |
| ENSECAG00000021760 | -2.4410863 | 0.00154631 | 0.00462755 |
| ENSECAG00000013572 | -2.4415805 | 1.75E-06 | 1.15E-05 |
| ENSECAG00000019689 | -2.4432455 | 0.00966735 | 0.02270488 |
| ENSECAG00000021630 | -2.444216 | 4.38E-06 | 2.58E-05 |
| ENSECAG00000016891 | -2.4444925 | 1.07E-05 | 5.70E-05 |
| ENSECAG00000021274 | -2.4446248 | 2.08E-09 | 2.79E-08 |
| ENSECAG00000049368 | -2.4457393 | 0.00709719 | 0.01742405 |
| ENSECAG00000023427 | -2.446364 | 1.74E-05 | 8.85E-05 |
| ENSECAG00000008973 | -2.4465512 | 2.67E-12 | 6.07E-11 |
| ENSECAG00000020277 | -2.4469443 | 1.25E-14 | 4.27E-13 |
| ENSECAG00000048984 | -2.4490793 | 0.00043112 | 0.00150835 |
| ENSECAG00000059677 | -2.4492073 | 2.85E-05 | 0.00013747 |
| ENSECAG00000038375 | -2.4502511 | 4.47E-08 | 4.31E-07 |
| ENSECAG00000053253 | -2.4527626 | 1.40E-08 | 1.53E-07 |
| ENSECAG00000030741 | -2.4550691 | 0.00160403 | 0.00477851 |
| ENSECAG00000050497 | -2.4564575 | 0.00258685 | 0.00726751 |
| ENSECAG00000015313 | -2.4577751 | 0.00648293 | 0.01612147 |
| ENSECAG00000000896 | -2.4588486 | 0.00054595 | 0.00185575 |
| ENSECAG00000044258 | -2.459519 | 0.00743048 | 0.01812088 |
| ENSECAG00000000693 | -2.4595248 | 0.00020532 | 0.00078885 |
| ENSECAG00000020738 | -2.4612963 | 0.00340059 | 0.00921981 |
| ENSECAG00000023950 | -2.4632843 | 0.00012048 | 0.00049499 |
| ENSECAG00000047700 | -2.4633499 | 0.00097087 | 0.00307951 |
| ENSECAG00000009254 | -2.4638312 | 1.51E-12 | 3.62E-11 |
| ENSECAG00000015048 | -2.4643596 | 1.97E-06 | 1.28E-05 |
| ENSECAG00000020979 | -2.4662442 | 4.01E-13 | 1.07E-11 |
| ENSECAG00000031375 | -2.4664037 | 2.90E-08 | 2.93E-07 |
| ENSECAG00000017701 | -2.4670724 | 7.18E-07 | 5.16E-06 |
| ENSECAG00000017268 | -2.4675589 | 8.46E-06 | 4.65E-05 |
| ENSECAG00000037218 | -2.4681183 | 0.00447041 | 0.01168098 |
| ENSECAG00000034702 | -2.4681641 | 6.83E-15 | 2.45E-13 |
| ENSECAG00000033160 | -2.4685776 | 1.46E-07 | 1.25E-06 |
| ENSECAG00000044130 | -2.4689488 | 0.01437538 | 0.03188092 |
| ENSECAG00000024615 | -2.4698508 | 5.12E-05 | 0.00023251 |
| ENSECAG00000020964 | -2.4701293 | 0.00558458 | 0.01418314 |
| ENSECAG00000049299 | -2.4702172 | 8.69E-14 | 2.57E-12 |
| ENSECAG00000009274 | -2.4718782 | 0.00476031 | 0.01235469 |
| ENSECAG00000015969 | -2.4734604 | 1.17E-05 | 6.20E-05 |
| ENSECAG00000003050 | -2.4735742 | 5.91E-06 | 3.37E-05 |
| ENSECAG00000022794 | -2.4739417 | 0.00068949 | 0.00227685 |
| ENSECAG00000009118 | -2.4755424 | 1.39E-08 | 1.53E-07 |
| ENSECAG00000012901 | -2.4758329 | 9.80E-14 | 2.87E-12 |
| ENSECAG00000028989 | -2.4759545 | 5.11E-05 | 0.00023215 |
| ENSECAG00000011934 | -2.4780174 | 0.0006457 | 0.00214907 |
| ENSECAG00000054221 | -2.478094 | 0.00740393 | 0.01806401 |
| ENSECAG00000053816 | -2.4782511 | 0.01343542 | 0.03006489 |
| ENSECAG00000057512 | -2.4797067 | 0.00050241 | 0.00172084 |
| ENSECAG00000041352 | -2.4798868 | 2.20E-07 | 1.80E-06 |
| ENSECAG00000055545 | -2.4799453 | 0.00088154 | 0.00283071 |
| ENSECAG00000047723 | -2.4815133 | 0.00201632 | 0.00585762 |
| ENSECAG00000047162 | -2.4839788 | 0.02360036 | 0.04864174 |
| ENSECAG00000021214 | -2.4845356 | 0.00104369 | 0.0032816 |
| ENSECAG00000023279 | -2.4847962 | 2.05E-08 | 2.16E-07 |
| ENSECAG00000039338 | -2.4857966 | 0.00365065 | 0.0098092 |
| ENSECAG00000016046 | -2.4862818 | 0.02297669 | 0.04758895 |
| ENSECAG00000018351 | -2.4863705 | 0.0010156 | 0.00320682 |
| ENSECAG00000052212 | -2.4868053 | 8.26E-06 | 4.54E-05 |
| ENSECAG00000003006 | -2.4869479 | 6.15E-22 | 6.55E-20 |
| ENSECAG00000045075 | -2.4879802 | 0.0006403 | 0.00213388 |
| ENSECAG00000019565 | -2.4890213 | 5.85E-11 | 1.06E-09 |
| ENSECAG00000016871 | -2.4890301 | 2.32E-05 | 0.00011449 |
| ENSECAG00000000240 | -2.4890537 | 0.00062262 | 0.00208403 |
| ENSECAG00000051602 | -2.4901431 | 0.00797056 | 0.01926556 |
| ENSECAG00000051681 | -2.4904742 | 0.00013291 | 0.0005383 |
| ENSECAG00000000659 | -2.4905604 | 3.28E-07 | 2.57E-06 |
| ENSECAG00000057610 | -2.4917204 | 2.60E-16 | 1.16E-14 |
| ENSECAG00000049857 | -2.4933189 | 0.00290624 | 0.00802954 |
| ENSECAG00000023751 | -2.4937602 | 2.43E-10 | 3.90E-09 |
| ENSECAG00000024805 | -2.4940104 | 0.00034141 | 0.0012335 |
| ENSECAG00000017301 | -2.494066 | 1.14E-05 | 6.08E-05 |
| ENSECAG00000015157 | -2.4948607 | 7.51E-07 | 5.38E-06 |
| ENSECAG00000027688 | -2.4957154 | 1.36E-07 | 1.17E-06 |
| ENSECAG00000002523 | -2.4961122 | 0.00198843 | 0.00578637 |
| ENSECAG00000059795 | -2.4974191 | 0.01407399 | 0.03129016 |
| ENSECAG00000047707 | -2.4979722 | 0.00012893 | 0.00052419 |
| ENSECAG00000015917 | -2.4981767 | 6.88E-23 | 8.09E-21 |
| ENSECAG00000047408 | -2.4981915 | 4.42E-12 | 9.67E-11 |
| ENSECAG00000047006 | -2.498763 | 0.00063272 | 0.00211246 |
| ENSECAG00000055352 | -2.4988521 | 2.12E-07 | 1.74E-06 |
| ENSECAG00000009704 | -2.4996284 | 3.52E-07 | 2.74E-06 |
| ENSECAG00000023608 | -2.4998383 | 4.22E-16 | 1.80E-14 |
| ENSECAG00000058498 | -2.5032215 | 1.23E-08 | 1.36E-07 |
| ENSECAG00000059428 | -2.5048187 | 1.97E-07 | 1.63E-06 |
| ENSECAG00000029773 | -2.5049591 | 2.03E-05 | 0.00010168 |
| ENSECAG00000030691 | -2.5053844 | 0.00180283 | 0.0053029 |
| ENSECAG00000036408 | -2.50544 | 0.00656695 | 0.01630138 |
| ENSECAG00000019990 | -2.5062449 | 7.92E-06 | 4.38E-05 |
| ENSECAG00000054225 | -2.5071952 | 6.63E-06 | 3.74E-05 |
| ENSECAG00000034047 | -2.5076604 | 0.0080852 | 0.01951312 |
| ENSECAG00000055342 | -2.5092268 | 0.0178291 | 0.03836389 |
| ENSECAG00000008182 | -2.5102014 | 2.72E-10 | 4.32E-09 |
| ENSECAG00000033092 | -2.5123841 | 1.26E-18 | 8.30E-17 |
| ENSECAG00000023569 | -2.5155105 | 0.00012527 | 0.00051157 |
| ENSECAG00000059609 | -2.5164284 | 4.46E-05 | 0.00020556 |
| ENSECAG00000020810 | -2.5178527 | 4.79E-08 | 4.58E-07 |
| ENSECAG00000053868 | -2.5183587 | 9.90E-09 | 1.12E-07 |
| ENSECAG00000008624 | -2.5186025 | 3.42E-05 | 0.00016176 |
| ENSECAG00000033073 | -2.5193605 | 1.68E-27 | 3.96E-25 |
| ENSECAG00000044778 | -2.5214499 | 0.0012639 | 0.00387902 |
| ENSECAG00000035477 | -2.5217755 | 6.53E-05 | 0.00028925 |
| ENSECAG00000032427 | -2.5223385 | 0.00104778 | 0.00329213 |
| ENSECAG00000051482 | -2.5263571 | 4.92E-10 | 7.42E-09 |
| ENSECAG00000016226 | -2.5274845 | 2.14E-07 | 1.75E-06 |
| ENSECAG00000024374 | -2.527526 | 4.97E-05 | 0.00022645 |
| ENSECAG00000017927 | -2.5284782 | 3.81E-06 | 2.29E-05 |
| ENSECAG00000011649 | -2.5323503 | 0.00085998 | 0.00276944 |
| ENSECAG00000045269 | -2.533037 | 0.00029627 | 0.00108924 |
| ENSECAG00000053587 | -2.5335182 | 4.20E-06 | 2.49E-05 |
| ENSECAG00000056707 | -2.5364812 | 0.00048641 | 0.00167556 |
| ENSECAG00000015845 | -2.5371199 | 7.80E-08 | 7.09E-07 |
| ENSECAG00000029381 | -2.538011 | 2.92E-32 | 1.28E-29 |
| ENSECAG00000000794 | -2.5387105 | 2.68E-11 | 5.17E-10 |
| ENSECAG00000017990 | -2.5401094 | 1.42E-10 | 2.38E-09 |
| ENSECAG00000052244 | -2.5407974 | 0.01735051 | 0.03744949 |
| ENSECAG00000019912 | -2.5414788 | 3.87E-06 | 2.32E-05 |
| ENSECAG00000021663 | -2.5420778 | 8.06E-09 | 9.36E-08 |
| ENSECAG00000006656 | -2.5428063 | 0.00290832 | 0.00803329 |
| ENSECAG00000016784 | -2.5430705 | 0.02111015 | 0.04431029 |
| ENSECAG00000010452 | -2.5437174 | 0.00055853 | 0.00189247 |
| ENSECAG00000046208 | -2.5440518 | 2.55E-08 | 2.63E-07 |
| ENSECAG00000021580 | -2.5451817 | 0.00856527 | 0.02049685 |
| ENSECAG00000010023 | -2.545417 | 2.05E-08 | 2.16E-07 |
| ENSECAG00000015153 | -2.5455246 | 3.02E-11 | 5.77E-10 |
| ENSECAG00000014410 | -2.5467455 | 9.96E-06 | 5.39E-05 |
| ENSECAG00000018690 | -2.5473198 | 0.00438685 | 0.01149625 |
| ENSECAG00000056772 | -2.5474846 | 0.00137508 | 0.00418459 |
| ENSECAG00000048364 | -2.547855 | 0.00297621 | 0.00819146 |
| ENSECAG00000010585 | -2.5479927 | 1.18E-05 | 6.27E-05 |
| ENSECAG00000056420 | -2.5487103 | 0.00397862 | 0.01057024 |
| ENSECAG00000014884 | -2.5488624 | 2.50E-05 | 0.00012219 |
| ENSECAG00000054759 | -2.5492093 | 2.25E-07 | 1.84E-06 |
| ENSECAG00000058996 | -2.5503704 | 0.00028075 | 0.00103821 |
| ENSECAG00000026369 | -2.5514454 | 0.00105212 | 0.00330206 |
| ENSECAG00000032511 | -2.5531387 | 0.00431527 | 0.01133527 |
| ENSECAG00000059170 | -2.5545426 | 5.67E-07 | 4.19E-06 |
| ENSECAG00000001466 | -2.5545723 | 6.90E-16 | 2.89E-14 |
| ENSECAG00000014103 | -2.5551508 | 0.00202548 | 0.00587887 |
| ENSECAG00000021420 | -2.5551961 | 0.00337793 | 0.00916615 |
| ENSECAG00000021628 | -2.5565727 | 1.41E-07 | 1.21E-06 |
| ENSECAG00000011515 | -2.5599445 | 0.00083873 | 0.00271115 |
| ENSECAG00000014489 | -2.5599509 | 9.01E-27 | 2.01E-24 |
| ENSECAG00000041942 | -2.5602485 | 5.15E-13 | 1.34E-11 |
| ENSECAG00000047093 | -2.5604505 | 1.26E-05 | 6.62E-05 |
| ENSECAG00000016019 | -2.5619321 | 1.33E-18 | 8.69E-17 |
| ENSECAG00000008591 | -2.5619627 | 2.08E-06 | 1.34E-05 |
| ENSECAG00000040351 | -2.5620168 | 4.13E-10 | 6.31E-09 |
| ENSECAG00000056466 | -2.5640578 | 0.00312046 | 0.00852547 |
| ENSECAG00000004768 | -2.5644588 | 1.33E-09 | 1.84E-08 |
| ENSECAG00000050525 | -2.5649784 | 5.46E-08 | 5.15E-07 |
| ENSECAG00000031561 | -2.565719 | 0.00303202 | 0.00832148 |
| ENSECAG00000009263 | -2.566458 | 7.27E-13 | 1.84E-11 |
| ENSECAG00000013267 | -2.5675275 | 1.68E-06 | 1.11E-05 |
| ENSECAG00000057976 | -2.5678045 | 1.34E-09 | 1.85E-08 |
| ENSECAG00000000488 | -2.5683185 | 0.00048794 | 0.00167902 |
| ENSECAG00000058159 | -2.5684584 | 0.00083422 | 0.00269894 |
| ENSECAG00000044483 | -2.5696511 | 0.00023242 | 0.00087984 |
| ENSECAG00000051991 | -2.570491 | 0.00023102 | 0.00087496 |
| ENSECAG00000045496 | -2.5720485 | 0.02361192 | 0.04866106 |
| ENSECAG00000023157 | -2.5736222 | 7.87E-08 | 7.14E-07 |
| ENSECAG00000020470 | -2.57367 | 0.00110894 | 0.00345611 |
| ENSECAG00000021978 | -2.5743323 | 1.39E-07 | 1.19E-06 |
| ENSECAG00000016786 | -2.5769873 | 1.66E-10 | 2.76E-09 |
| ENSECAG00000019665 | -2.5782471 | 7.43E-14 | 2.24E-12 |
| ENSECAG00000015421 | -2.5796917 | 0.00405169 | 0.01072484 |
| ENSECAG00000018308 | -2.5809785 | 1.57E-07 | 1.33E-06 |
| ENSECAG00000048747 | -2.5818013 | 0.00126005 | 0.00386825 |
| ENSECAG00000022647 | -2.5819432 | 3.04E-16 | 1.34E-14 |
| ENSECAG00000041432 | -2.5829002 | 0.00122701 | 0.00377949 |
| ENSECAG00000020345 | -2.5834547 | 2.81E-12 | 6.34E-11 |
| ENSECAG00000029987 | -2.5840412 | 0.00012971 | 0.00052711 |
| ENSECAG00000049256 | -2.5842765 | 0.00034334 | 0.00123985 |
| ENSECAG00000060266 | -2.5846313 | 0.0001219 | 0.00049998 |
| ENSECAG00000011264 | -2.5856619 | 7.77E-05 | 0.00033754 |
| ENSECAG00000060362 | -2.5856739 | 1.73E-08 | 1.86E-07 |
| ENSECAG00000058901 | -2.586349 | 0.00497551 | 0.01283273 |
| ENSECAG00000057884 | -2.5877542 | 0.00264372 | 0.00740309 |
| ENSECAG00000033654 | -2.5919059 | 0.01170863 | 0.02674994 |
| ENSECAG00000044351 | -2.5939151 | 3.15E-05 | 0.00015066 |
| ENSECAG00000048004 | -2.5940552 | 0.01622253 | 0.03537359 |
| ENSECAG00000042946 | -2.5946553 | 0.00104853 | 0.00329388 |
| ENSECAG00000060334 | -2.595205 | 0.0014772 | 0.00444992 |
| ENSECAG00000033909 | -2.5981455 | 2.46E-05 | 0.00012046 |
| ENSECAG00000053576 | -2.5986963 | 0.00060092 | 0.00201879 |
| ENSECAG00000051793 | -2.5989943 | 7.81E-05 | 0.00033892 |
| ENSECAG00000018987 | -2.6002111 | 4.61E-08 | 4.43E-07 |
| ENSECAG00000057448 | -2.600696 | 1.41E-05 | 7.37E-05 |
| ENSECAG00000000843 | -2.6007291 | 2.89E-25 | 5.12E-23 |
| ENSECAG00000047473 | -2.6013653 | 0.00569572 | 0.01441408 |
| ENSECAG00000053802 | -2.6030262 | 0.01567927 | 0.03437012 |
| ENSECAG00000044223 | -2.6030896 | 6.11E-06 | 3.48E-05 |
| ENSECAG00000020849 | -2.6046793 | 0.00019988 | 0.00077115 |
| ENSECAG00000049542 | -2.6053008 | 0.00578822 | 0.01462171 |
| ENSECAG00000013978 | -2.6072869 | 1.12E-25 | 2.15E-23 |
| ENSECAG00000021347 | -2.6136611 | 0.00142375 | 0.00431134 |
| ENSECAG00000004961 | -2.6155503 | 0.0005121 | 0.00175244 |
| ENSECAG00000020216 | -2.6156645 | 0.00237289 | 0.00674612 |
| ENSECAG00000035401 | -2.6156859 | 3.14E-08 | 3.14E-07 |
| ENSECAG00000054472 | -2.6157768 | 0.01177393 | 0.02687987 |
| ENSECAG00000020883 | -2.6163919 | 0.00550296 | 0.01400401 |
| ENSECAG00000034161 | -2.6164227 | 4.71E-05 | 0.0002156 |
| ENSECAG00000012771 | -2.6178738 | 3.82E-08 | 3.74E-07 |
| ENSECAG00000043846 | -2.6187963 | 0.01374425 | 0.03067302 |
| ENSECAG00000009164 | -2.6196571 | 1.56E-05 | 8.04E-05 |
| ENSECAG00000014976 | -2.6216587 | 7.32E-10 | 1.06E-08 |
| ENSECAG00000049672 | -2.6219343 | 1.97E-11 | 3.87E-10 |
| ENSECAG00000010486 | -2.622332 | 1.04E-05 | 5.57E-05 |
| ENSECAG00000057024 | -2.6227943 | 0.00010658 | 0.00044568 |
| ENSECAG00000017957 | -2.6230744 | 0.00117546 | 0.00363908 |
| ENSECAG00000014801 | -2.624581 | 1.20E-21 | 1.23E-19 |
| ENSECAG00000016718 | -2.6245843 | 0.00024776 | 0.00093048 |
| ENSECAG00000024613 | -2.6290328 | 2.56E-12 | 5.83E-11 |
| ENSECAG00000015721 | -2.6298538 | 0.00010906 | 0.00045451 |
| ENSECAG00000014027 | -2.631356 | 1.54E-06 | 1.03E-05 |
| ENSECAG00000000264 | -2.631754 | 4.97E-06 | 2.89E-05 |
| ENSECAG00000056008 | -2.6331074 | 0.01598536 | 0.0349502 |
| ENSECAG00000015446 | -2.633714 | 1.87E-05 | 9.46E-05 |
| ENSECAG00000003193 | -2.6342581 | 1.74E-08 | 1.87E-07 |
| ENSECAG00000023604 | -2.635008 | 0.00189578 | 0.0055423 |
| ENSECAG00000030943 | -2.6356158 | 0.00389119 | 0.01037372 |
| ENSECAG00000026978 | -2.6372245 | 1.21E-10 | 2.07E-09 |
| ENSECAG00000002714 | -2.6372505 | 0.00012989 | 0.00052772 |
| ENSECAG00000056556 | -2.6388106 | 0.01621608 | 0.03536643 |
| ENSECAG00000045297 | -2.6392003 | 0.00018878 | 0.00073086 |
| ENSECAG00000023274 | -2.6413344 | 3.21E-24 | 4.85E-22 |
| ENSECAG00000021520 | -2.6417821 | 5.69E-11 | 1.04E-09 |
| ENSECAG00000057761 | -2.6420713 | 0.00898722 | 0.02134202 |
| ENSECAG00000022186 | -2.6422919 | 9.54E-07 | 6.67E-06 |
| ENSECAG00000047974 | -2.6424732 | 0.00070102 | 0.00230875 |
| ENSECAG00000050479 | -2.642555 | 2.91E-05 | 0.00014003 |
| ENSECAG00000052151 | -2.6428637 | 0.00084566 | 0.00273001 |
| ENSECAG00000012533 | -2.645016 | 0.00404643 | 0.01071219 |
| ENSECAG00000056615 | -2.6457742 | 0.00223778 | 0.00641423 |
| ENSECAG00000056180 | -2.6460601 | 0.00146547 | 0.00442193 |
| ENSECAG00000000771 | -2.6473803 | 1.90E-08 | 2.03E-07 |
| ENSECAG00000024403 | -2.6508814 | 0.02156102 | 0.04509149 |
| ENSECAG00000050004 | -2.6536202 | 2.60E-13 | 7.12E-12 |
| ENSECAG00000016717 | -2.6569265 | 0.00042224 | 0.00148307 |
| ENSECAG00000021586 | -2.659107 | 4.65E-09 | 5.73E-08 |
| ENSECAG00000037885 | -2.659667 | 3.20E-06 | 1.96E-05 |
| ENSECAG00000060179 | -2.6617557 | 5.64E-05 | 0.00025309 |
| ENSECAG00000048786 | -2.6643787 | 5.60E-07 | 4.15E-06 |
| ENSECAG00000016406 | -2.6644239 | 0.00595332 | 0.01497613 |
| ENSECAG00000054720 | -2.6645006 | 1.04E-10 | 1.81E-09 |
| ENSECAG00000022902 | -2.6648669 | 3.17E-06 | 1.94E-05 |
| ENSECAG00000035754 | -2.6650596 | 0.00143543 | 0.00434047 |
| ENSECAG00000038185 | -2.6657234 | 0.00020001 | 0.00077152 |
| ENSECAG00000052763 | -2.6667578 | 1.30E-05 | 6.79E-05 |
| ENSECAG00000024045 | -2.6667723 | 4.68E-27 | 1.08E-24 |
| ENSECAG00000021411 | -2.6676124 | 5.12E-08 | 4.86E-07 |
| ENSECAG00000031885 | -2.6676504 | 0.00084033 | 0.00271593 |
| ENSECAG00000020082 | -2.6704712 | 4.87E-06 | 2.84E-05 |
| ENSECAG00000056758 | -2.670725 | 9.82E-06 | 5.32E-05 |
| ENSECAG00000007840 | -2.6717234 | 0.02005543 | 0.04239911 |
| ENSECAG00000011792 | -2.6744445 | 9.98E-07 | 6.95E-06 |
| ENSECAG00000005701 | -2.6756266 | 8.52E-19 | 5.81E-17 |
| ENSECAG00000020410 | -2.6777846 | 1.16E-06 | 7.97E-06 |
| ENSECAG00000022986 | -2.6791136 | 3.58E-07 | 2.78E-06 |
| ENSECAG00000056063 | -2.6829785 | 0.00069673 | 0.00229715 |
| ENSECAG00000055401 | -2.6839615 | 7.66E-05 | 0.00033327 |
| ENSECAG00000052843 | -2.6846543 | 0.00959588 | 0.02256679 |
| ENSECAG00000005082 | -2.6863043 | 8.26E-20 | 6.64E-18 |
| ENSECAG00000017699 | -2.687457 | 6.37E-26 | 1.27E-23 |
| ENSECAG00000017611 | -2.6878641 | 0.00049633 | 0.00170263 |
| ENSECAG00000044513 | -2.6879678 | 0.00062961 | 0.00210462 |
| ENSECAG00000050385 | -2.6907606 | 0.00440566 | 0.01154089 |
| ENSECAG00000011744 | -2.691322 | 1.62E-15 | 6.48E-14 |
| ENSECAG00000016327 | -2.6928598 | 0.00016575 | 0.00065368 |
| ENSECAG00000050716 | -2.692926 | 1.45E-05 | 7.51E-05 |
| ENSECAG00000041350 | -2.6933022 | 0.00208868 | 0.00603642 |
| ENSECAG00000055621 | -2.6940324 | 0.01046518 | 0.02431177 |
| ENSECAG00000029767 | -2.6967105 | 5.06E-07 | 3.80E-06 |
| ENSECAG00000011718 | -2.6980158 | 0.0001124 | 0.00046695 |
| ENSECAG00000022425 | -2.7001763 | 0.01550239 | 0.03404919 |
| ENSECAG00000043800 | -2.7014771 | 0.00023633 | 0.00089329 |
| ENSECAG00000024701 | -2.7024634 | 7.98E-09 | 9.28E-08 |
| ENSECAG00000048815 | -2.7025569 | 0.00043727 | 0.00152763 |
| ENSECAG00000023335 | -2.7025639 | 8.93E-06 | 4.88E-05 |
| ENSECAG00000013315 | -2.7040166 | 0.00026989 | 0.00100399 |
| ENSECAG00000000185 | -2.7102143 | 2.14E-07 | 1.75E-06 |
| ENSECAG00000025270 | -2.7102768 | 1.24E-06 | 8.46E-06 |
| ENSECAG00000023528 | -2.7122776 | 3.39E-07 | 2.65E-06 |
| ENSECAG00000052290 | -2.7129963 | 0.0046468 | 0.01209802 |
| ENSECAG00000021729 | -2.7133328 | 8.02E-05 | 0.0003468 |
| ENSECAG00000051648 | -2.7133799 | 1.17E-05 | 6.21E-05 |
| ENSECAG00000055164 | -2.7136462 | 0.00081282 | 0.00263579 |
| ENSECAG00000056301 | -2.7190908 | 2.86E-12 | 6.43E-11 |
| ENSECAG00000015174 | -2.7193111 | 0.00275405 | 0.00766304 |
| ENSECAG00000046430 | -2.7208994 | 0.00035229 | 0.00126665 |
| ENSECAG00000016951 | -2.7218514 | 2.37E-06 | 1.51E-05 |
| ENSECAG00000057958 | -2.722576 | 0.00044857 | 0.00156182 |
| ENSECAG00000043272 | -2.7240066 | 1.28E-08 | 1.41E-07 |
| ENSECAG00000014109 | -2.7253267 | 0.02103632 | 0.04418853 |
| ENSECAG00000032497 | -2.7256213 | 9.16E-10 | 1.31E-08 |
| ENSECAG00000059387 | -2.7266328 | 0.00311465 | 0.00851215 |
| ENSECAG00000049052 | -2.7267597 | 0.00064837 | 0.00215602 |
| ENSECAG00000054633 | -2.7295585 | 0.01581334 | 0.03462664 |
| ENSECAG00000036783 | -2.7296387 | 2.17E-13 | 6.02E-12 |
| ENSECAG00000024951 | -2.7309503 | 0.00016947 | 0.00066649 |
| ENSECAG00000017507 | -2.7319719 | 8.42E-06 | 4.62E-05 |
| ENSECAG00000000616 | -2.7378797 | 5.65E-05 | 0.00025372 |
| ENSECAG00000011594 | -2.7380314 | 1.64E-07 | 1.38E-06 |
| ENSECAG00000013719 | -2.7383634 | 0.01022277 | 0.02381299 |
| ENSECAG00000017191 | -2.7394088 | 1.55E-07 | 1.32E-06 |
| ENSECAG00000047827 | -2.7396969 | 1.63E-08 | 1.76E-07 |
| ENSECAG00000019511 | -2.7399184 | 5.81E-06 | 3.32E-05 |
| ENSECAG00000056501 | -2.7415253 | 0.00087878 | 0.00282348 |
| ENSECAG00000058543 | -2.7425203 | 2.65E-05 | 0.00012873 |
| ENSECAG00000016041 | -2.7431907 | 8.40E-36 | 5.87E-33 |
| ENSECAG00000050422 | -2.7432733 | 7.45E-07 | 5.34E-06 |
| ENSECAG00000044656 | -2.7439546 | 1.57E-05 | 8.08E-05 |
| ENSECAG00000012027 | -2.7444841 | 8.32E-09 | 9.62E-08 |
| ENSECAG00000059237 | -2.7476459 | 1.46E-12 | 3.52E-11 |
| ENSECAG00000012524 | -2.7479295 | 8.14E-09 | 9.44E-08 |
| ENSECAG00000024589 | -2.7485751 | 4.75E-06 | 2.77E-05 |
| ENSECAG00000003355 | -2.7492249 | 1.57E-24 | 2.47E-22 |
| ENSECAG00000048345 | -2.7514184 | 2.98E-07 | 2.36E-06 |
| ENSECAG00000019818 | -2.7527447 | 3.74E-09 | 4.70E-08 |
| ENSECAG00000045949 | -2.7528201 | 1.50E-11 | 3.04E-10 |
| ENSECAG00000046463 | -2.7540056 | 2.34E-08 | 2.43E-07 |
| ENSECAG00000000207 | -2.7542006 | 6.55E-09 | 7.81E-08 |
| ENSECAG00000015526 | -2.7545224 | 0.01004023 | 0.02344645 |
| ENSECAG00000041411 | -2.7546216 | 2.52E-07 | 2.02E-06 |
| ENSECAG00000021639 | -2.7571908 | 0.00061522 | 0.00206083 |
| ENSECAG00000060287 | -2.7574707 | 0.00551792 | 0.01403567 |
| ENSECAG00000051024 | -2.7588525 | 2.36E-05 | 0.00011656 |
| ENSECAG00000045754 | -2.7604149 | 0.01187893 | 0.02708637 |
| ENSECAG00000060082 | -2.7620608 | 1.41E-09 | 1.94E-08 |
| ENSECAG00000041634 | -2.762067 | 0.00866898 | 0.02070732 |
| ENSECAG00000012510 | -2.7625362 | 3.81E-15 | 1.44E-13 |
| ENSECAG00000010225 | -2.763457 | 7.30E-10 | 1.06E-08 |
| ENSECAG00000041639 | -2.7634731 | 0.00278489 | 0.00772867 |
| ENSECAG00000007956 | -2.76393 | 0.00074478 | 0.00243664 |
| ENSECAG00000056126 | -2.7640223 | 0.00091041 | 0.00291044 |
| ENSECAG00000002188 | -2.7646162 | 3.02E-05 | 0.0001448 |
| ENSECAG00000017862 | -2.7649639 | 8.27E-19 | 5.67E-17 |
| ENSECAG00000047581 | -2.7672166 | 1.64E-15 | 6.57E-14 |
| ENSECAG00000049081 | -2.7673858 | 9.64E-07 | 6.74E-06 |
| ENSECAG00000014484 | -2.7674801 | 3.05E-08 | 3.07E-07 |
| ENSECAG00000053917 | -2.7681367 | 0.00375682 | 0.01005508 |
| ENSECAG00000031932 | -2.7686483 | 2.23E-06 | 1.42E-05 |
| ENSECAG00000018039 | -2.7695907 | 1.34E-07 | 1.16E-06 |
| ENSECAG00000042902 | -2.7696199 | 7.71E-11 | 1.37E-09 |
| ENSECAG00000053144 | -2.7701483 | 1.88E-05 | 9.51E-05 |
| ENSECAG00000013341 | -2.770176 | 1.24E-16 | 5.83E-15 |
| ENSECAG00000009450 | -2.7704532 | 3.95E-09 | 4.95E-08 |
| ENSECAG00000055630 | -2.7710409 | 0.01168996 | 0.02672094 |
| ENSECAG00000020709 | -2.7715759 | 0.00350898 | 0.00947116 |
| ENSECAG00000024821 | -2.7718012 | 4.05E-05 | 0.00018841 |
| ENSECAG00000024288 | -2.7732891 | 5.99E-06 | 3.42E-05 |
| ENSECAG00000053566 | -2.7736046 | 6.65E-05 | 0.00029342 |
| ENSECAG00000007080 | -2.7737076 | 0.00032231 | 0.001173 |
| ENSECAG00000010555 | -2.7738493 | 8.94E-13 | 2.23E-11 |
| ENSECAG00000029964 | -2.7738506 | 6.19E-23 | 7.43E-21 |
| ENSECAG00000023321 | -2.7740304 | 8.23E-08 | 7.44E-07 |
| ENSECAG00000023091 | -2.7742744 | 7.41E-06 | 4.13E-05 |
| ENSECAG00000051194 | -2.7749478 | 2.54E-05 | 0.00012403 |
| ENSECAG00000024872 | -2.7756806 | 1.50E-06 | 1.00E-05 |
| ENSECAG00000047272 | -2.7766513 | 6.43E-15 | 2.31E-13 |
| ENSECAG00000014143 | -2.7773603 | 0.00016955 | 0.00066668 |
| ENSECAG00000019278 | -2.7774114 | 5.89E-08 | 5.50E-07 |
| ENSECAG00000017897 | -2.777747 | 1.25E-06 | 8.53E-06 |
| ENSECAG00000028354 | -2.7778945 | 1.43E-07 | 1.23E-06 |
| ENSECAG00000058880 | -2.7794867 | 0.01177053 | 0.02687486 |
| ENSECAG00000013155 | -2.7803757 | 1.16E-06 | 7.97E-06 |
| ENSECAG00000000278 | -2.7816456 | 0.00158056 | 0.0047175 |
| ENSECAG00000010312 | -2.7817986 | 3.34E-12 | 7.46E-11 |
| ENSECAG00000010022 | -2.7829851 | 2.87E-06 | 1.78E-05 |
| ENSECAG00000008190 | -2.7834157 | 0.00031776 | 0.00115909 |
| ENSECAG00000000132 | -2.7845579 | 6.01E-14 | 1.85E-12 |
| ENSECAG00000052854 | -2.7856076 | 0.00146573 | 0.00442193 |
| ENSECAG00000059581 | -2.7864078 | 8.04E-05 | 0.00034783 |
| ENSECAG00000034994 | -2.7867217 | 0.01599816 | 0.03497309 |
| ENSECAG00000020486 | -2.7875124 | 0.00111577 | 0.00347257 |
| ENSECAG00000045956 | -2.7885083 | 0.00283746 | 0.00785503 |
| ENSECAG00000032533 | -2.7885517 | 0.00603134 | 0.01515192 |
| ENSECAG00000015918 | -2.788563 | 2.27E-05 | 0.00011246 |
| ENSECAG00000050025 | -2.7907024 | 0.00515707 | 0.01324293 |
| ENSECAG00000054950 | -2.7908554 | 0.00032749 | 0.00118955 |
| ENSECAG00000050480 | -2.7921166 | 3.77E-07 | 2.91E-06 |
| ENSECAG00000021683 | -2.7935138 | 6.27E-05 | 0.00027902 |
| ENSECAG00000010528 | -2.7935615 | 1.78E-06 | 1.16E-05 |
| ENSECAG00000050116 | -2.7946038 | 4.37E-05 | 0.00020198 |
| ENSECAG00000021169 | -2.7946488 | 5.83E-08 | 5.45E-07 |
| ENSECAG00000047793 | -2.7947909 | 4.11E-07 | 3.14E-06 |
| ENSECAG00000044995 | -2.7954729 | 1.32E-16 | 6.19E-15 |
| ENSECAG00000050555 | -2.7966743 | 0.00024031 | 0.00090541 |
| ENSECAG00000023982 | -2.7993818 | 0.00580299 | 0.01465403 |
| ENSECAG00000019231 | -2.8002321 | 1.63E-07 | 1.38E-06 |
| ENSECAG00000004028 | -2.8008936 | 0.0007101 | 0.00233488 |
| ENSECAG00000024600 | -2.8010517 | 0.00106269 | 0.00332963 |
| ENSECAG00000006764 | -2.8019135 | 0.0030425 | 0.00834717 |
| ENSECAG00000007985 | -2.8021961 | 7.62E-06 | 4.24E-05 |
| ENSECAG00000047499 | -2.8023406 | 0.00097969 | 0.00310307 |
| ENSECAG00000014485 | -2.804468 | 0.00013339 | 0.0005396 |
| ENSECAG00000045800 | -2.8057958 | 4.49E-06 | 2.64E-05 |
| ENSECAG00000047114 | -2.8059902 | 6.06E-10 | 9.03E-09 |
| ENSECAG00000059929 | -2.8067956 | 4.49E-08 | 4.33E-07 |
| ENSECAG00000045123 | -2.8068433 | 2.59E-06 | 1.63E-05 |
| ENSECAG00000056832 | -2.8107734 | 4.48E-05 | 0.00020614 |
| ENSECAG00000055826 | -2.8116272 | 0.00919874 | 0.02174955 |
| ENSECAG00000056052 | -2.8117799 | 5.26E-05 | 0.00023783 |
| ENSECAG00000028632 | -2.8118432 | 0.00754705 | 0.01835108 |
| ENSECAG00000007606 | -2.815474 | 3.40E-05 | 0.00016119 |
| ENSECAG00000013422 | -2.8155571 | 9.69E-19 | 6.50E-17 |
| ENSECAG00000005226 | -2.8158497 | 8.16E-05 | 0.00035194 |
| ENSECAG00000003757 | -2.8201968 | 0.00011419 | 0.00047278 |
| ENSECAG00000007664 | -2.8211321 | 0.00174156 | 0.00514502 |
| ENSECAG00000019853 | -2.8217852 | 6.57E-13 | 1.68E-11 |
| ENSECAG00000052078 | -2.8235382 | 3.52E-05 | 0.00016626 |
| ENSECAG00000007460 | -2.8238535 | 4.63E-06 | 2.71E-05 |
| ENSECAG00000006980 | -2.82415 | 1.69E-10 | 2.80E-09 |
| ENSECAG00000035856 | -2.8243479 | 0.00026257 | 0.000981 |
| ENSECAG00000016966 | -2.8251644 | 9.98E-10 | 1.41E-08 |
| ENSECAG00000053257 | -2.8251776 | 0.00013677 | 0.00055158 |
| ENSECAG00000031726 | -2.825463 | 8.04E-09 | 9.35E-08 |
| ENSECAG00000009334 | -2.8259239 | 0.00013907 | 0.00055985 |
| ENSECAG00000058354 | -2.8271706 | 3.57E-05 | 0.00016837 |
| ENSECAG00000007127 | -2.8304077 | 3.22E-05 | 0.00015331 |
| ENSECAG00000009262 | -2.8322681 | 5.22E-06 | 3.02E-05 |
| ENSECAG00000019108 | -2.832859 | 1.48E-10 | 2.48E-09 |
| ENSECAG00000007366 | -2.8349072 | 1.58E-10 | 2.63E-09 |
| ENSECAG00000048167 | -2.8358085 | 1.66E-05 | 8.47E-05 |
| ENSECAG00000050129 | -2.836701 | 2.06E-08 | 2.17E-07 |
| ENSECAG00000026287 | -2.8369334 | 6.16E-08 | 5.74E-07 |
| ENSECAG00000050738 | -2.8387137 | 0.00613967 | 0.01538181 |
| ENSECAG00000011615 | -2.8398463 | 1.43E-07 | 1.23E-06 |
| ENSECAG00000011720 | -2.8400485 | 5.51E-12 | 1.19E-10 |
| ENSECAG00000058839 | -2.8410623 | 0.00452554 | 0.01181536 |
| ENSECAG00000036893 | -2.8428605 | 0.01268175 | 0.02859023 |
| ENSECAG00000050897 | -2.8449459 | 2.81E-17 | 1.50E-15 |
| ENSECAG00000021087 | -2.8453376 | 5.58E-07 | 4.14E-06 |
| ENSECAG00000050554 | -2.8456097 | 0.00018574 | 0.00072144 |
| ENSECAG00000036028 | -2.8458233 | 0.00024325 | 0.00091556 |
| ENSECAG00000049715 | -2.8480172 | 4.27E-05 | 0.00019774 |
| ENSECAG00000019156 | -2.8481179 | 5.13E-05 | 0.00023272 |
| ENSECAG00000008794 | -2.8489571 | 1.57E-08 | 1.70E-07 |
| ENSECAG00000007595 | -2.8496091 | 0.00028587 | 0.00105605 |
| ENSECAG00000038504 | -2.8513298 | 2.74E-15 | 1.06E-13 |
| ENSECAG00000032026 | -2.8518189 | 0.00019146 | 0.00074059 |
| ENSECAG00000045744 | -2.8542799 | 4.45E-18 | 2.70E-16 |
| ENSECAG00000031615 | -2.8547029 | 0.00638478 | 0.01592169 |
| ENSECAG00000018259 | -2.8602688 | 3.55E-05 | 0.00016703 |
| ENSECAG00000045111 | -2.8606644 | 0.00086431 | 0.00278218 |
| ENSECAG00000050688 | -2.8630777 | 9.79E-06 | 5.30E-05 |
| ENSECAG00000018004 | -2.8631183 | 1.23E-05 | 6.50E-05 |
| ENSECAG00000035745 | -2.8637916 | 1.18E-13 | 3.39E-12 |
| ENSECAG00000054461 | -2.8642046 | 2.63E-05 | 0.0001279 |
| ENSECAG00000002601 | -2.8643162 | 1.80E-10 | 2.97E-09 |
| ENSECAG00000018863 | -2.8671717 | 3.22E-17 | 1.70E-15 |
| ENSECAG00000022367 | -2.8690244 | 0.00090778 | 0.0029037 |
| ENSECAG00000054682 | -2.8699053 | 0.00010957 | 0.00045654 |
| ENSECAG00000009075 | -2.870654 | 3.87E-10 | 5.96E-09 |
| ENSECAG00000047465 | -2.8719605 | 1.95E-07 | 1.62E-06 |
| ENSECAG00000060154 | -2.8726961 | 0.00213842 | 0.00616343 |
| ENSECAG00000013616 | -2.8729902 | 1.22E-06 | 8.33E-06 |
| ENSECAG00000056941 | -2.8739548 | 8.59E-05 | 0.00036854 |
| ENSECAG00000046953 | -2.8757848 | 2.10E-08 | 2.21E-07 |
| ENSECAG00000010794 | -2.8761891 | 0.00590112 | 0.01487158 |
| ENSECAG00000010997 | -2.8775378 | 0.0024874 | 0.00701546 |
| ENSECAG00000025167 | -2.877659 | 3.15E-24 | 4.79E-22 |
| ENSECAG00000010048 | -2.8780157 | 6.85E-07 | 4.96E-06 |
| ENSECAG00000031616 | -2.87847 | 4.94E-15 | 1.82E-13 |
| ENSECAG00000020555 | -2.8793353 | 1.94E-08 | 2.06E-07 |
| ENSECAG00000014444 | -2.8836551 | 4.17E-16 | 1.79E-14 |
| ENSECAG00000055209 | -2.8836947 | 0.00167298 | 0.00496406 |
| ENSECAG00000031377 | -2.8866846 | 5.11E-07 | 3.83E-06 |
| ENSECAG00000000288 | -2.8893869 | 5.97E-07 | 4.38E-06 |
| ENSECAG00000046089 | -2.8897024 | 0.01064161 | 0.02465245 |
| ENSECAG00000041545 | -2.8901603 | 1.06E-08 | 1.19E-07 |
| ENSECAG00000007828 | -2.8943305 | 9.01E-06 | 4.92E-05 |
| ENSECAG00000003602 | -2.8966616 | 1.91E-12 | 4.49E-11 |
| ENSECAG00000040402 | -2.8969089 | 0.00184099 | 0.00540215 |
| ENSECAG00000059744 | -2.8977566 | 3.02E-05 | 0.00014501 |
| ENSECAG00000019335 | -2.8982063 | 5.81E-07 | 4.28E-06 |
| ENSECAG00000000627 | -2.8991114 | 2.83E-08 | 2.87E-07 |
| ENSECAG00000023129 | -2.9001484 | 0.00018925 | 0.00073254 |
| ENSECAG00000051918 | -2.9022159 | 0.01298038 | 0.02919572 |
| ENSECAG00000048338 | -2.9027483 | 6.79E-07 | 4.92E-06 |
| ENSECAG00000010860 | -2.9030438 | 3.93E-05 | 0.00018363 |
| ENSECAG00000014596 | -2.9055761 | 1.68E-07 | 1.41E-06 |
| ENSECAG00000055385 | -2.9061316 | 0.00743258 | 0.01812401 |
| ENSECAG00000048623 | -2.907751 | 4.18E-06 | 2.48E-05 |
| ENSECAG00000037788 | -2.9100266 | 2.53E-05 | 0.00012375 |
| ENSECAG00000035223 | -2.910421 | 0.00030804 | 0.00112753 |
| ENSECAG00000018663 | -2.9110391 | 7.14E-08 | 6.54E-07 |
| ENSECAG00000017939 | -2.9123175 | 6.80E-09 | 8.06E-08 |
| ENSECAG00000010612 | -2.9127254 | 3.99E-05 | 0.00018597 |
| ENSECAG00000056720 | -2.9143827 | 0.00036698 | 0.0013104 |
| ENSECAG00000058202 | -2.9150018 | 2.48E-06 | 1.56E-05 |
| ENSECAG00000044317 | -2.9159625 | 1.21E-09 | 1.69E-08 |
| ENSECAG00000039610 | -2.9162867 | 7.69E-05 | 0.00033438 |
| ENSECAG00000018853 | -2.9164814 | 1.88E-11 | 3.72E-10 |
| ENSECAG00000056827 | -2.9179944 | 3.22E-10 | 5.06E-09 |
| ENSECAG00000050282 | -2.919159 | 0.00051207 | 0.00175244 |
| ENSECAG00000045901 | -2.9195427 | 1.63E-05 | 8.36E-05 |
| ENSECAG00000010525 | -2.9206861 | 0.000349 | 0.00125685 |
| ENSECAG00000002710 | -2.9227206 | 0.0007726 | 0.00251855 |
| ENSECAG00000025446 | -2.9242235 | 3.91E-10 | 6.01E-09 |
| ENSECAG00000047396 | -2.9246128 | 5.13E-06 | 2.97E-05 |
| ENSECAG00000044425 | -2.9254814 | 0.00353358 | 0.00952603 |
| ENSECAG00000043290 | -2.9255898 | 0.00971836 | 0.02280922 |
| ENSECAG00000013092 | -2.9265032 | 1.24E-10 | 2.12E-09 |
| ENSECAG00000048832 | -2.9295452 | 0.0039858 | 0.01058573 |
| ENSECAG00000051197 | -2.9300099 | 3.12E-09 | 4.00E-08 |
| ENSECAG00000028239 | -2.9332665 | 0.01309154 | 0.02942797 |
| ENSECAG00000011313 | -2.93344 | 3.48E-08 | 3.45E-07 |
| ENSECAG00000053331 | -2.93367 | 1.56E-05 | 8.04E-05 |
| ENSECAG00000039439 | -2.936577 | 8.47E-07 | 5.99E-06 |
| ENSECAG00000051159 | -2.9377384 | 7.83E-05 | 0.00033957 |
| ENSECAG00000041015 | -2.9377975 | 0.00379591 | 0.01015084 |
| ENSECAG00000020763 | -2.9390759 | 3.27E-07 | 2.56E-06 |
| ENSECAG00000022214 | -2.9393654 | 2.95E-05 | 0.00014211 |
| ENSECAG00000044689 | -2.9393834 | 4.69E-05 | 0.00021496 |
| ENSECAG00000021591 | -2.9397765 | 5.55E-21 | 4.98E-19 |
| ENSECAG00000019741 | -2.9405154 | 5.43E-15 | 1.98E-13 |
| ENSECAG00000054757 | -2.9418719 | 0.00058192 | 0.00196164 |
| ENSECAG00000047513 | -2.9420485 | 5.90E-29 | 1.65E-26 |
| ENSECAG00000025002 | -2.9442433 | 1.39E-11 | 2.82E-10 |
| ENSECAG00000049194 | -2.9456544 | 1.84E-06 | 1.20E-05 |
| ENSECAG00000012909 | -2.9478302 | 1.84E-07 | 1.54E-06 |
| ENSECAG00000059126 | -2.9479008 | 0.00949156 | 0.0223591 |
| ENSECAG00000044759 | -2.9483432 | 2.37E-05 | 0.00011673 |
| ENSECAG00000058567 | -2.9488797 | 0.00220912 | 0.00634349 |
| ENSECAG00000044563 | -2.9508065 | 4.05E-06 | 2.41E-05 |
| ENSECAG00000009918 | -2.9546069 | 2.98E-10 | 4.70E-09 |
| ENSECAG00000055590 | -2.9546639 | 0.00201895 | 0.00586372 |
| ENSECAG00000025981 | -2.9554269 | 0.01321202 | 0.02965704 |
| ENSECAG00000054474 | -2.9555264 | 8.40E-05 | 0.000361 |
| ENSECAG00000020903 | -2.9563022 | 2.15E-08 | 2.26E-07 |
| ENSECAG00000041203 | -2.9568735 | 8.74E-05 | 0.00037419 |
| ENSECAG00000008703 | -2.9573663 | 1.93E-05 | 9.72E-05 |
| ENSECAG00000021074 | -2.9576336 | 2.60E-08 | 2.67E-07 |
| ENSECAG00000044054 | -2.9581251 | 0.00039502 | 0.00139665 |
| ENSECAG00000041624 | -2.9586075 | 0.00692617 | 0.01706043 |
| ENSECAG00000031222 | -2.9590214 | 1.06E-05 | 5.70E-05 |
| ENSECAG00000019905 | -2.9617527 | 8.07E-09 | 9.37E-08 |
| ENSECAG00000005150 | -2.9625124 | 2.69E-05 | 0.00013075 |
| ENSECAG00000051376 | -2.9633464 | 3.68E-08 | 3.62E-07 |
| ENSECAG00000026894 | -2.967002 | 2.38E-06 | 1.51E-05 |
| ENSECAG00000058470 | -2.9692024 | 6.91E-08 | 6.36E-07 |
| ENSECAG00000021703 | -2.9696921 | 2.06E-07 | 1.70E-06 |
| ENSECAG00000059255 | -2.9700903 | 1.80E-06 | 1.18E-05 |
| ENSECAG00000024867 | -2.9707486 | 9.79E-07 | 6.83E-06 |
| ENSECAG00000007132 | -2.9715335 | 2.58E-15 | 9.97E-14 |
| ENSECAG00000020777 | -2.9737823 | 1.12E-08 | 1.25E-07 |
| ENSECAG00000010714 | -2.9752743 | 0.00027305 | 0.00101405 |
| ENSECAG00000019573 | -2.9797141 | 8.54E-05 | 0.00036651 |
| ENSECAG00000023491 | -2.9801541 | 5.39E-05 | 0.00024304 |
| ENSECAG00000000099 | -2.9805476 | 1.08E-23 | 1.47E-21 |
| ENSECAG00000058985 | -2.9812821 | 0.00027057 | 0.00100603 |
| ENSECAG00000046861 | -2.9815617 | 1.01E-05 | 5.45E-05 |
| ENSECAG00000013428 | -2.9823682 | 7.97E-29 | 2.20E-26 |
| ENSECAG00000019485 | -2.983778 | 4.66E-12 | 1.01E-10 |
| ENSECAG00000056398 | -2.9864854 | 3.20E-08 | 3.20E-07 |
| ENSECAG00000059778 | -2.9875516 | 1.39E-23 | 1.85E-21 |
| ENSECAG00000014790 | -2.9886573 | 5.39E-05 | 0.000243 |
| ENSECAG00000014656 | -2.9888544 | 0.00612927 | 0.01536339 |
| ENSECAG00000028842 | -2.9892242 | 3.80E-09 | 4.77E-08 |
| ENSECAG00000015761 | -2.9898649 | 1.17E-05 | 6.23E-05 |
| ENSECAG00000043980 | -2.9948689 | 0.00258684 | 0.00726751 |
| ENSECAG00000021590 | -2.9950746 | 3.09E-06 | 1.90E-05 |
| ENSECAG00000001834 | -2.9962236 | 1.46E-12 | 3.52E-11 |
| ENSECAG00000046354 | -2.9977295 | 0.01473052 | 0.0325619 |
| ENSECAG00000023801 | -2.9981498 | 2.42E-21 | 2.32E-19 |
| ENSECAG00000047711 | -2.998791 | 0.00643199 | 0.01601619 |
| ENSECAG00000048288 | -2.99946 | 0.00101307 | 0.00320064 |
| ENSECAG00000059543 | -2.9999389 | 0.00735209 | 0.01796106 |
| ENSECAG00000025402 | -3.0010677 | 5.89E-07 | 4.34E-06 |
| ENSECAG00000000612 | -3.0017265 | 2.23E-06 | 1.43E-05 |
| ENSECAG00000030119 | -3.0017567 | 0.00230437 | 0.00657895 |
| ENSECAG00000056294 | -3.0018828 | 0.00493753 | 0.01275244 |
| ENSECAG00000009734 | -3.0019458 | 0.00013416 | 0.00054233 |
| ENSECAG00000054102 | -3.002722 | 0.00057639 | 0.00194651 |
| ENSECAG00000033324 | -3.0031516 | 0.00052105 | 0.00178033 |
| ENSECAG00000023426 | -3.0041253 | 3.56E-08 | 3.52E-07 |
| ENSECAG00000002111 | -3.0059678 | 2.24E-25 | 4.04E-23 |
| ENSECAG00000022333 | -3.0065469 | 7.72E-18 | 4.51E-16 |
| ENSECAG00000033927 | -3.0093431 | 0.00157966 | 0.00471597 |
| ENSECAG00000043491 | -3.0094755 | 1.61E-05 | 8.26E-05 |
| ENSECAG00000046550 | -3.0097524 | 0.00017439 | 0.00068308 |
| ENSECAG00000021702 | -3.0099743 | 5.07E-14 | 1.58E-12 |
| ENSECAG00000057764 | -3.0111701 | 0.00486893 | 0.01259899 |
| ENSECAG00000049842 | -3.0112709 | 0.0001902 | 0.00073595 |
| ENSECAG00000053501 | -3.0119178 | 2.57E-06 | 1.62E-05 |
| ENSECAG00000048197 | -3.0134099 | 7.02E-05 | 0.00030823 |
| ENSECAG00000033591 | -3.0146026 | 3.38E-09 | 4.29E-08 |
| ENSECAG00000010886 | -3.0146791 | 2.97E-06 | 1.84E-05 |
| ENSECAG00000035414 | -3.0155579 | 1.15E-05 | 6.09E-05 |
| ENSECAG00000058426 | -3.0179835 | 2.21E-05 | 0.0001095 |
| ENSECAG00000049388 | -3.0198961 | 0.00011088 | 0.00046155 |
| ENSECAG00000051626 | -3.019993 | 1.90E-08 | 2.03E-07 |
| ENSECAG00000011123 | -3.0215136 | 0.00027219 | 0.00101132 |
| ENSECAG00000024630 | -3.0218234 | 2.85E-06 | 1.77E-05 |
| ENSECAG00000013811 | -3.0242344 | 6.04E-10 | 9.00E-09 |
| ENSECAG00000044524 | -3.02468 | 0.00013004 | 0.00052823 |
| ENSECAG00000008463 | -3.0247084 | 0.00024504 | 0.00092153 |
| ENSECAG00000000174 | -3.0254009 | 7.20E-14 | 2.18E-12 |
| ENSECAG00000024692 | -3.0278073 | 0.00020488 | 0.00078732 |
| ENSECAG00000010643 | -3.0282617 | 9.94E-09 | 1.12E-07 |
| ENSECAG00000049660 | -3.0311595 | 0.00123584 | 0.00380222 |
| ENSECAG00000055616 | -3.0314653 | 9.39E-05 | 0.00039814 |
| ENSECAG00000026876 | -3.0329244 | 1.00E-07 | 8.88E-07 |
| ENSECAG00000037094 | -3.0347256 | 0.00018609 | 0.00072233 |
| ENSECAG00000058540 | -3.0348211 | 3.72E-07 | 2.88E-06 |
| ENSECAG00000055377 | -3.0354684 | 9.17E-07 | 6.44E-06 |
| ENSECAG00000060252 | -3.037026 | 5.41E-06 | 3.12E-05 |
| ENSECAG00000045564 | -3.0404136 | 3.92E-06 | 2.35E-05 |
| ENSECAG00000054444 | -3.0406499 | 7.91E-07 | 5.63E-06 |
| ENSECAG00000050163 | -3.0425314 | 0.00017385 | 0.00068157 |
| ENSECAG00000048314 | -3.042793 | 2.01E-08 | 2.13E-07 |
| ENSECAG00000049792 | -3.0432443 | 4.51E-05 | 0.00020752 |
| ENSECAG00000054620 | -3.0454043 | 0.00045214 | 0.00157303 |
| ENSECAG00000054448 | -3.0467847 | 2.70E-08 | 2.75E-07 |
| ENSECAG00000003990 | -3.0486991 | 1.08E-08 | 1.22E-07 |
| ENSECAG00000014137 | -3.0490548 | 1.19E-12 | 2.91E-11 |
| ENSECAG00000044617 | -3.0491892 | 4.61E-09 | 5.69E-08 |
| ENSECAG00000059192 | -3.0500116 | 0.00343059 | 0.00929099 |
| ENSECAG00000046189 | -3.0507386 | 1.06E-05 | 5.67E-05 |
| ENSECAG00000048135 | -3.0519024 | 1.62E-06 | 1.07E-05 |
| ENSECAG00000024803 | -3.0524794 | 3.12E-06 | 1.92E-05 |
| ENSECAG00000007040 | -3.0528863 | 2.94E-07 | 2.33E-06 |
| ENSECAG00000046360 | -3.0546669 | 0.01682692 | 0.0364782 |
| ENSECAG00000025306 | -3.0549119 | 1.15E-07 | 1.01E-06 |
| ENSECAG00000007335 | -3.0567967 | 0.00020404 | 0.00078477 |
| ENSECAG00000060302 | -3.0568503 | 0.0001859 | 0.00072181 |
| ENSECAG00000038904 | -3.0568609 | 3.83E-07 | 2.95E-06 |
| ENSECAG00000058925 | -3.0572736 | 0.01105152 | 0.02546476 |
| ENSECAG00000015909 | -3.0609022 | 2.79E-06 | 1.74E-05 |
| ENSECAG00000014099 | -3.0611906 | 0.00066528 | 0.00220633 |
| ENSECAG00000035133 | -3.0624397 | 3.19E-07 | 2.51E-06 |
| ENSECAG00000022733 | -3.0630754 | 0.00054911 | 0.00186417 |
| ENSECAG00000014219 | -3.0636922 | 1.33E-11 | 2.70E-10 |
| ENSECAG00000016397 | -3.0640941 | 0.00501284 | 0.0129141 |
| ENSECAG00000017403 | -3.0645019 | 2.48E-05 | 0.00012156 |
| ENSECAG00000008479 | -3.0648244 | 5.59E-08 | 5.26E-07 |
| ENSECAG00000006567 | -3.0667227 | 1.15E-08 | 1.28E-07 |
| ENSECAG00000015732 | -3.0672124 | 0.00151577 | 0.00455321 |
| ENSECAG00000010533 | -3.067234 | 3.30E-07 | 2.58E-06 |
| ENSECAG00000018409 | -3.0715236 | 4.11E-05 | 0.00019089 |
| ENSECAG00000057326 | -3.0732622 | 7.66E-13 | 1.93E-11 |
| ENSECAG00000059433 | -3.0737415 | 3.92E-06 | 2.34E-05 |
| ENSECAG00000036634 | -3.0748328 | 3.36E-10 | 5.23E-09 |
| ENSECAG00000024305 | -3.0750412 | 2.97E-07 | 2.36E-06 |
| ENSECAG00000022144 | -3.0764912 | 2.93E-05 | 0.00014122 |
| ENSECAG00000045315 | -3.0768669 | 3.34E-07 | 2.61E-06 |
| ENSECAG00000054397 | -3.0809691 | 0.01132115 | 0.02600291 |
| ENSECAG00000006482 | -3.084194 | 4.61E-05 | 0.00021187 |
| ENSECAG00000057784 | -3.0845967 | 5.55E-07 | 4.13E-06 |
| ENSECAG00000050003 | -3.085171 | 1.68E-07 | 1.42E-06 |
| ENSECAG00000015199 | -3.0881709 | 9.35E-08 | 8.33E-07 |
| ENSECAG00000053607 | -3.0895536 | 7.92E-06 | 4.38E-05 |
| ENSECAG00000023276 | -3.0904424 | 0.00069424 | 0.00229083 |
| ENSECAG00000017481 | -3.0916386 | 0.00058698 | 0.00197688 |
| ENSECAG00000012762 | -3.0918619 | 6.74E-07 | 4.88E-06 |
| ENSECAG00000014658 | -3.0938134 | 6.66E-06 | 3.76E-05 |
| ENSECAG00000053851 | -3.0939891 | 8.25E-09 | 9.56E-08 |
| ENSECAG00000009238 | -3.0960189 | 0.00101334 | 0.00320102 |
| ENSECAG00000016623 | -3.0967679 | 1.05E-10 | 1.83E-09 |
| ENSECAG00000035160 | -3.0972311 | 1.80E-07 | 1.51E-06 |
| ENSECAG00000024330 | -3.0973672 | 0.0050056 | 0.01289693 |
| ENSECAG00000054988 | -3.097594 | 0.00058184 | 0.00196164 |
| ENSECAG00000013590 | -3.0993253 | 2.83E-11 | 5.45E-10 |
| ENSECAG00000010833 | -3.0993606 | 3.11E-08 | 3.12E-07 |
| ENSECAG00000053286 | -3.1042137 | 1.12E-05 | 5.98E-05 |
| ENSECAG00000011771 | -3.1047734 | 9.13E-08 | 8.16E-07 |
| ENSECAG00000046518 | -3.1051515 | 9.65E-18 | 5.57E-16 |
| ENSECAG00000031668 | -3.1056045 | 1.46E-15 | 5.89E-14 |
| ENSECAG00000055835 | -3.1092664 | 0.00168612 | 0.00499909 |
| ENSECAG00000015254 | -3.1095423 | 2.22E-06 | 1.42E-05 |
| ENSECAG00000015046 | -3.110851 | 3.81E-06 | 2.29E-05 |
| ENSECAG00000047234 | -3.1112032 | 0.00535289 | 0.01367817 |
| ENSECAG00000039992 | -3.1123845 | 2.11E-06 | 1.36E-05 |
| ENSECAG00000051756 | -3.1139391 | 6.07E-07 | 4.45E-06 |
| ENSECAG00000045200 | -3.1152075 | 4.90E-23 | 5.98E-21 |
| ENSECAG00000024535 | -3.1158645 | 1.81E-09 | 2.47E-08 |
| ENSECAG00000007138 | -3.1163159 | 5.40E-15 | 1.97E-13 |
| ENSECAG00000056252 | -3.1198102 | 0.0010374 | 0.00326459 |
| ENSECAG00000059633 | -3.1199991 | 0.00846637 | 0.02029059 |
| ENSECAG00000052834 | -3.1206689 | 4.53E-08 | 4.36E-07 |
| ENSECAG00000010767 | -3.121291 | 8.43E-08 | 7.60E-07 |
| ENSECAG00000009839 | -3.1227832 | 6.22E-06 | 3.53E-05 |
| ENSECAG00000058015 | -3.1243337 | 8.41E-06 | 4.62E-05 |
| ENSECAG00000007414 | -3.1258719 | 0.00018262 | 0.00071117 |
| ENSECAG00000055325 | -3.1272236 | 0.00011797 | 0.00048636 |
| ENSECAG00000046598 | -3.1278375 | 1.54E-07 | 1.31E-06 |
| ENSECAG00000023423 | -3.1283102 | 1.47E-08 | 1.60E-07 |
| ENSECAG00000016321 | -3.1309388 | 7.45E-05 | 0.00032508 |
| ENSECAG00000015710 | -3.132533 | 4.71E-06 | 2.75E-05 |
| ENSECAG00000015798 | -3.132834 | 0.00012752 | 0.00051914 |
| ENSECAG00000013584 | -3.1341515 | 4.58E-06 | 2.68E-05 |
| ENSECAG00000011498 | -3.1351278 | 2.07E-09 | 2.78E-08 |
| ENSECAG00000046998 | -3.1383775 | 2.78E-07 | 2.22E-06 |
| ENSECAG00000039545 | -3.1385651 | 4.53E-07 | 3.44E-06 |
| ENSECAG00000021803 | -3.1388621 | 7.71E-10 | 1.12E-08 |
| ENSECAG00000058396 | -3.1390494 | 2.19E-12 | 5.08E-11 |
| ENSECAG00000048758 | -3.1394404 | 2.59E-08 | 2.66E-07 |
| ENSECAG00000049752 | -3.1424902 | 0.00021753 | 0.00082958 |
| ENSECAG00000024861 | -3.1444324 | 1.37E-06 | 9.24E-06 |
| ENSECAG00000053071 | -3.1470503 | 1.09E-08 | 1.23E-07 |
| ENSECAG00000009208 | -3.1485574 | 9.24E-05 | 0.0003929 |
| ENSECAG00000055153 | -3.152308 | 3.84E-06 | 2.30E-05 |
| ENSECAG00000053850 | -3.1532602 | 0.00124969 | 0.00384121 |
| ENSECAG00000009873 | -3.1574941 | 3.94E-17 | 2.02E-15 |
| ENSECAG00000059409 | -3.1583868 | 0.00522801 | 0.01339431 |
| ENSECAG00000000330 | -3.1601625 | 1.50E-05 | 7.75E-05 |
| ENSECAG00000044459 | -3.1603374 | 0.02133936 | 0.04470743 |
| ENSECAG00000004381 | -3.1612187 | 5.18E-06 | 3.00E-05 |
| ENSECAG00000030856 | -3.1615992 | 1.37E-08 | 1.50E-07 |
| ENSECAG00000048772 | -3.1618376 | 2.09E-06 | 1.34E-05 |
| ENSECAG00000053306 | -3.1632705 | 2.14E-11 | 4.18E-10 |
| ENSECAG00000058826 | -3.1652412 | 0.00015048 | 0.0006007 |
| ENSECAG00000010275 | -3.1679755 | 1.44E-05 | 7.48E-05 |
| ENSECAG00000032733 | -3.1681282 | 3.07E-17 | 1.63E-15 |
| ENSECAG00000054197 | -3.1686858 | 1.73E-08 | 1.86E-07 |
| ENSECAG00000044918 | -3.1687481 | 2.00E-07 | 1.66E-06 |
| ENSECAG00000002780 | -3.169462 | 9.67E-06 | 5.25E-05 |
| ENSECAG00000023783 | -3.1704368 | 1.06E-05 | 5.70E-05 |
| ENSECAG00000046347 | -3.1722185 | 0.00263066 | 0.00737391 |
| ENSECAG00000013822 | -3.1739554 | 1.36E-12 | 3.30E-11 |
| ENSECAG00000008749 | -3.1747682 | 2.99E-17 | 1.59E-15 |
| ENSECAG00000013594 | -3.1752198 | 0.023275 | 0.04809091 |
| ENSECAG00000016072 | -3.1804162 | 6.19E-06 | 3.51E-05 |
| ENSECAG00000043029 | -3.1807257 | 4.34E-11 | 8.09E-10 |
| ENSECAG00000021078 | -3.1816122 | 8.27E-07 | 5.86E-06 |
| ENSECAG00000055453 | -3.1816326 | 2.87E-05 | 0.00013869 |
| ENSECAG00000013354 | -3.1836819 | 0.00132287 | 0.00404443 |
| ENSECAG00000030969 | -3.1851208 | 3.29E-05 | 0.00015632 |
| ENSECAG00000036242 | -3.1871432 | 4.65E-06 | 2.72E-05 |
| ENSECAG00000049856 | -3.1887285 | 4.40E-10 | 6.66E-09 |
| ENSECAG00000054570 | -3.1913857 | 0.00155148 | 0.00464114 |
| ENSECAG00000042459 | -3.1918686 | 0.00069744 | 0.00229879 |
| ENSECAG00000000779 | -3.1949137 | 7.92E-06 | 4.38E-05 |
| ENSECAG00000040540 | -3.1949236 | 2.26E-12 | 5.21E-11 |
| ENSECAG00000006002 | -3.1974443 | 3.88E-11 | 7.27E-10 |
| ENSECAG00000034602 | -3.2078476 | 1.46E-23 | 1.93E-21 |
| ENSECAG00000044848 | -3.2096375 | 0.00230359 | 0.00657895 |
| ENSECAG00000053969 | -3.2106842 | 0.00051143 | 0.00175067 |
| ENSECAG00000038379 | -3.212845 | 0.00011667 | 0.00048135 |
| ENSECAG00000059289 | -3.212889 | 9.72E-14 | 2.85E-12 |
| ENSECAG00000026278 | -3.214446 | 0.00025846 | 0.00096744 |
| ENSECAG00000048296 | -3.2161375 | 1.10E-06 | 7.61E-06 |
| ENSECAG00000055218 | -3.2174308 | 2.64E-13 | 7.23E-12 |
| ENSECAG00000023047 | -3.2182596 | 5.00E-05 | 0.00022738 |
| ENSECAG00000027670 | -3.2190307 | 7.09E-05 | 0.000311 |
| ENSECAG00000045720 | -3.2191195 | 8.53E-06 | 4.68E-05 |
| ENSECAG00000060259 | -3.2191979 | 7.01E-06 | 3.93E-05 |
| ENSECAG00000021645 | -3.221705 | 4.23E-09 | 5.26E-08 |
| ENSECAG00000057611 | -3.2219317 | 5.72E-07 | 4.23E-06 |
| ENSECAG00000057944 | -3.2222425 | 1.55E-06 | 1.03E-05 |
| ENSECAG00000012641 | -3.2236947 | 0.00025583 | 0.00095839 |
| ENSECAG00000046538 | -3.2241812 | 2.85E-06 | 1.77E-05 |
| ENSECAG00000000875 | -3.2248528 | 2.86E-09 | 3.71E-08 |
| ENSECAG00000053010 | -3.2311143 | 1.15E-05 | 6.14E-05 |
| ENSECAG00000023022 | -3.231417 | 6.37E-08 | 5.91E-07 |
| ENSECAG00000044462 | -3.2327466 | 6.39E-07 | 4.66E-06 |
| ENSECAG00000015462 | -3.2332673 | 0.00033848 | 0.00122429 |
| ENSECAG00000046626 | -3.2340341 | 7.29E-05 | 0.00031901 |
| ENSECAG00000019680 | -3.2341465 | 1.28E-07 | 1.11E-06 |
| ENSECAG00000014588 | -3.2356321 | 7.72E-15 | 2.74E-13 |
| ENSECAG00000019273 | -3.2399098 | 5.75E-06 | 3.30E-05 |
| ENSECAG00000047625 | -3.2420275 | 0.01873464 | 0.04001189 |
| ENSECAG00000051407 | -3.2435303 | 1.80E-05 | 9.10E-05 |
| ENSECAG00000036299 | -3.2455216 | 0.00335767 | 0.00912115 |
| ENSECAG00000035748 | -3.2455666 | 0.00224624 | 0.00643519 |
| ENSECAG00000052276 | -3.246845 | 3.18E-06 | 1.95E-05 |
| ENSECAG00000050920 | -3.2474928 | 0.00010819 | 0.00045131 |
| ENSECAG00000022069 | -3.2483125 | 5.13E-09 | 6.26E-08 |
| ENSECAG00000024808 | -3.2502807 | 8.17E-07 | 5.79E-06 |
| ENSECAG00000012114 | -3.2526281 | 5.76E-08 | 5.40E-07 |
| ENSECAG00000051322 | -3.2538087 | 1.52E-14 | 5.13E-13 |
| ENSECAG00000020026 | -3.2547848 | 4.02E-08 | 3.93E-07 |
| ENSECAG00000030807 | -3.2567606 | 0.00013394 | 0.00054161 |
| ENSECAG00000049589 | -3.2571858 | 0.0002916 | 0.00107403 |
| ENSECAG00000059194 | -3.2578404 | 3.63E-12 | 8.04E-11 |
| ENSECAG00000043433 | -3.2588461 | 0.01022736 | 0.0238212 |
| ENSECAG00000045235 | -3.2589102 | 3.09E-08 | 3.10E-07 |
| ENSECAG00000057507 | -3.2621504 | 6.81E-13 | 1.74E-11 |
| ENSECAG00000055539 | -3.2643772 | 9.62E-19 | 6.48E-17 |
| ENSECAG00000021578 | -3.2644145 | 1.99E-09 | 2.68E-08 |
| ENSECAG00000056069 | -3.2646046 | 3.36E-14 | 1.08E-12 |
| ENSECAG00000059405 | -3.2675446 | 0.00283014 | 0.00783865 |
| ENSECAG00000014515 | -3.2688295 | 1.99E-09 | 2.68E-08 |
| ENSECAG00000027995 | -3.2698346 | 9.67E-07 | 6.75E-06 |
| ENSECAG00000019992 | -3.2718584 | 9.49E-17 | 4.56E-15 |
| ENSECAG00000015399 | -3.2720639 | 2.09E-05 | 0.00010457 |
| ENSECAG00000000973 | -3.2730649 | 2.02E-06 | 1.30E-05 |
| ENSECAG00000017896 | -3.2735874 | 4.59E-15 | 1.70E-13 |
| ENSECAG00000033590 | -3.2738754 | 4.59E-06 | 2.69E-05 |
| ENSECAG00000011684 | -3.2770686 | 0.0001002 | 0.00042199 |
| ENSECAG00000046267 | -3.2771419 | 0.00024215 | 0.00091186 |
| ENSECAG00000051076 | -3.2774414 | 8.69E-07 | 6.13E-06 |
| ENSECAG00000009538 | -3.2787386 | 2.39E-05 | 0.00011743 |
| ENSECAG00000059973 | -3.2790385 | 8.99E-05 | 0.00038339 |
| ENSECAG00000014954 | -3.2815441 | 0.00176263 | 0.00519833 |
| ENSECAG00000044330 | -3.2823016 | 2.93E-06 | 1.82E-05 |
| ENSECAG00000059144 | -3.2829182 | 3.72E-05 | 0.00017457 |
| ENSECAG00000002658 | -3.2835226 | 1.62E-07 | 1.37E-06 |
| ENSECAG00000025166 | -3.2845104 | 3.58E-21 | 3.36E-19 |
| ENSECAG00000013022 | -3.2849352 | 0.00015743 | 0.00062498 |
| ENSECAG00000043529 | -3.2867803 | 3.68E-05 | 0.00017293 |
| ENSECAG00000013967 | -3.2873619 | 0.00094432 | 0.0030068 |
| ENSECAG00000037936 | -3.2878775 | 5.19E-09 | 6.32E-08 |
| ENSECAG00000024542 | -3.28852 | 3.12E-07 | 2.46E-06 |
| ENSECAG00000052050 | -3.2927717 | 1.99E-05 | 0.00010011 |
| ENSECAG00000050252 | -3.2959659 | 0.00246372 | 0.00696361 |
| ENSECAG00000048419 | -3.2961228 | 0.00287132 | 0.00793993 |
| ENSECAG00000013832 | -3.2980566 | 1.10E-24 | 1.76E-22 |
| ENSECAG00000020191 | -3.3005562 | 8.64E-05 | 0.00037039 |
| ENSECAG00000045420 | -3.3007951 | 0.00945872 | 0.02228879 |
| ENSECAG00000015626 | -3.3060759 | 8.89E-05 | 0.00037992 |
| ENSECAG00000024538 | -3.3062456 | 2.13E-14 | 7.00E-13 |
| ENSECAG00000019575 | -3.3067613 | 0.00069982 | 0.00230551 |
| ENSECAG00000055194 | -3.3085974 | 1.00E-06 | 6.98E-06 |
| ENSECAG00000035535 | -3.3096756 | 0.00052735 | 0.00179994 |
| ENSECAG00000040574 | -3.3114541 | 9.13E-09 | 1.04E-07 |
| ENSECAG00000049743 | -3.3124094 | 4.59E-09 | 5.67E-08 |
| ENSECAG00000049473 | -3.314101 | 0.0003546 | 0.00127375 |
| ENSECAG00000009922 | -3.3179457 | 3.24E-10 | 5.08E-09 |
| ENSECAG00000053866 | -3.3180349 | 0.00498953 | 0.01286443 |
| ENSECAG00000048665 | -3.3212826 | 7.21E-05 | 0.0003157 |
| ENSECAG00000048609 | -3.3236456 | 3.39E-09 | 4.30E-08 |
| ENSECAG00000014927 | -3.3242014 | 0.00014938 | 0.00059702 |
| ENSECAG00000024827 | -3.3242352 | 1.10E-13 | 3.16E-12 |
| ENSECAG00000025148 | -3.3280023 | 1.87E-08 | 2.00E-07 |
| ENSECAG00000044420 | -3.3308356 | 2.97E-08 | 3.00E-07 |
| ENSECAG00000058002 | -3.3312684 | 0.00206349 | 0.00597134 |
| ENSECAG00000035970 | -3.3324825 | 0.00182448 | 0.00535907 |
| ENSECAG00000059806 | -3.3337975 | 0.00946209 | 0.02229437 |
| ENSECAG00000058688 | -3.3382633 | 0.00036713 | 0.00131075 |
| ENSECAG00000058803 | -3.3391924 | 7.51E-06 | 4.18E-05 |
| ENSECAG00000042447 | -3.3392333 | 0.00778683 | 0.01886636 |
| ENSECAG00000027719 | -3.3396424 | 4.33E-11 | 8.07E-10 |
| ENSECAG00000029001 | -3.340447 | 0.00109065 | 0.00340576 |
| ENSECAG00000001004 | -3.3417614 | 1.37E-10 | 2.31E-09 |
| ENSECAG00000038147 | -3.3446578 | 0.0009449 | 0.00300821 |
| ENSECAG00000047159 | -3.3452661 | 1.57E-11 | 3.16E-10 |
| ENSECAG00000016163 | -3.3461801 | 0.00087043 | 0.00279988 |
| ENSECAG00000051554 | -3.3462296 | 0.00298217 | 0.00820557 |
| ENSECAG00000011866 | -3.3476797 | 0.00225731 | 0.0064636 |
| ENSECAG00000023231 | -3.3507398 | 5.99E-09 | 7.18E-08 |
| ENSECAG00000058743 | -3.3550477 | 0.00032991 | 0.00119602 |
| ENSECAG00000058483 | -3.3566068 | 3.57E-11 | 6.75E-10 |
| ENSECAG00000018680 | -3.356904 | 0.00355549 | 0.00957703 |
| ENSECAG00000024915 | -3.3573096 | 4.97E-07 | 3.74E-06 |
| ENSECAG00000018053 | -3.3580895 | 0.00088855 | 0.00285076 |
| ENSECAG00000043869 | -3.3583956 | 9.19E-05 | 0.00039127 |
| ENSECAG00000012203 | -3.3585274 | 4.90E-05 | 0.00022349 |
| ENSECAG00000053767 | -3.3599089 | 9.33E-11 | 1.64E-09 |
| ENSECAG00000044122 | -3.3613132 | 0.00090014 | 0.00288298 |
| ENSECAG00000020165 | -3.36432 | 0.00027326 | 0.00101467 |
| ENSECAG00000021944 | -3.3644688 | 9.68E-07 | 6.76E-06 |
| ENSECAG00000006012 | -3.3660572 | 1.07E-05 | 5.72E-05 |
| ENSECAG00000014604 | -3.3709351 | 8.43E-05 | 0.00036237 |
| ENSECAG00000044495 | -3.372202 | 0.001695 | 0.00502208 |
| ENSECAG00000028980 | -3.3742245 | 1.31E-12 | 3.17E-11 |
| ENSECAG00000000272 | -3.3763017 | 0.00068488 | 0.00226514 |
| ENSECAG00000049198 | -3.3788366 | 0.00025947 | 0.00097103 |
| ENSECAG00000026977 | -3.3790263 | 1.53E-06 | 1.02E-05 |
| ENSECAG00000002332 | -3.3805395 | 1.43E-06 | 9.58E-06 |
| ENSECAG00000000417 | -3.3849542 | 1.26E-11 | 2.58E-10 |
| ENSECAG00000049257 | -3.3882485 | 0.00130763 | 0.00400332 |
| ENSECAG00000008621 | -3.3897366 | 1.03E-06 | 7.14E-06 |
| ENSECAG00000005738 | -3.3918894 | 6.73E-07 | 4.88E-06 |
| ENSECAG00000049561 | -3.3946193 | 9.20E-06 | 5.01E-05 |
| ENSECAG00000057612 | -3.3952494 | 0.00030385 | 0.00111363 |
| ENSECAG00000021800 | -3.398877 | 1.86E-05 | 9.40E-05 |
| ENSECAG00000030307 | -3.3988884 | 6.33E-08 | 5.88E-07 |
| ENSECAG00000008810 | -3.4029135 | 2.20E-08 | 2.30E-07 |
| ENSECAG00000052198 | -3.4042146 | 1.08E-06 | 7.47E-06 |
| ENSECAG00000017843 | -3.4044864 | 8.17E-11 | 1.45E-09 |
| ENSECAG00000027210 | -3.4048228 | 3.10E-13 | 8.41E-12 |
| ENSECAG00000050225 | -3.4078151 | 0.016318 | 0.03555749 |
| ENSECAG00000022689 | -3.4090622 | 2.51E-07 | 2.02E-06 |
| ENSECAG00000022928 | -3.4105121 | 1.03E-09 | 1.45E-08 |
| ENSECAG00000059914 | -3.4142191 | 1.19E-10 | 2.04E-09 |
| ENSECAG00000004708 | -3.4146564 | 1.23E-05 | 6.51E-05 |
| ENSECAG00000038087 | -3.4160468 | 0.00855415 | 0.02047682 |
| ENSECAG00000017495 | -3.4164225 | 4.39E-11 | 8.17E-10 |
| ENSECAG00000013613 | -3.4185243 | 4.84E-14 | 1.52E-12 |
| ENSECAG00000008983 | -3.4201076 | 1.09E-18 | 7.26E-17 |
| ENSECAG00000043211 | -3.4207361 | 0.00013104 | 0.00053161 |
| ENSECAG00000009123 | -3.4224219 | 0.00024712 | 0.00092823 |
| ENSECAG00000046571 | -3.4259916 | 1.35E-06 | 9.14E-06 |
| ENSECAG00000043995 | -3.4265782 | 0.00252567 | 0.0071171 |
| ENSECAG00000043493 | -3.4273205 | 3.05E-08 | 3.07E-07 |
| ENSECAG00000011375 | -3.4320936 | 4.55E-05 | 0.00020926 |
| ENSECAG00000053578 | -3.4336237 | 1.13E-07 | 9.94E-07 |
| ENSECAG00000024969 | -3.4343778 | 0.00175731 | 0.00518539 |
| ENSECAG00000045575 | -3.4344475 | 9.96E-08 | 8.82E-07 |
| ENSECAG00000020613 | -3.4367772 | 2.88E-09 | 3.73E-08 |
| ENSECAG00000028608 | -3.4408443 | 4.55E-06 | 2.67E-05 |
| ENSECAG00000053211 | -3.4410185 | 8.47E-07 | 5.99E-06 |
| ENSECAG00000047222 | -3.4415809 | 2.64E-05 | 0.00012855 |
| ENSECAG00000023443 | -3.4437706 | 4.21E-06 | 2.49E-05 |
| ENSECAG00000023550 | -3.4449139 | 4.00E-06 | 2.38E-05 |
| ENSECAG00000029340 | -3.4500812 | 0.00192113 | 0.00561024 |
| ENSECAG00000036190 | -3.4502439 | 4.33E-06 | 2.56E-05 |
| ENSECAG00000022329 | -3.4508512 | 1.26E-13 | 3.60E-12 |
| ENSECAG00000052724 | -3.4532223 | 0.0023768 | 0.00675468 |
| ENSECAG00000037686 | -3.4541667 | 0.00076819 | 0.00250453 |
| ENSECAG00000034443 | -3.4554696 | 0.00396561 | 0.01054571 |
| ENSECAG00000019084 | -3.4554992 | 1.57E-07 | 1.33E-06 |
| ENSECAG00000049487 | -3.4559815 | 0.00015599 | 0.0006198 |
| ENSECAG00000011289 | -3.4585389 | 0.00012202 | 0.00050039 |
| ENSECAG00000014930 | -3.4609265 | 0.00066007 | 0.00219101 |
| ENSECAG00000022594 | -3.46134 | 4.89E-10 | 7.38E-09 |
| ENSECAG00000014454 | -3.4628087 | 0.00045733 | 0.00158911 |
| ENSECAG00000016177 | -3.4634661 | 3.96E-07 | 3.04E-06 |
| ENSECAG00000019422 | -3.4682442 | 7.26E-07 | 5.22E-06 |
| ENSECAG00000013249 | -3.4691291 | 0.00049136 | 0.0016887 |
| ENSECAG00000020713 | -3.469593 | 7.79E-05 | 0.00033845 |
| ENSECAG00000046499 | -3.4707427 | 0.00263343 | 0.00738075 |
| ENSECAG00000010741 | -3.4722771 | 9.99E-08 | 8.84E-07 |
| ENSECAG00000009548 | -3.4725434 | 5.13E-05 | 0.00023265 |
| ENSECAG00000021775 | -3.4733526 | 1.33E-06 | 9.00E-06 |
| ENSECAG00000024570 | -3.4745644 | 5.21E-05 | 0.00023591 |
| ENSECAG00000051798 | -3.4748447 | 0.00014818 | 0.0005932 |
| ENSECAG00000043793 | -3.4759127 | 1.60E-05 | 8.21E-05 |
| ENSECAG00000006590 | -3.4809947 | 9.51E-12 | 1.98E-10 |
| ENSECAG00000056892 | -3.4820294 | 6.86E-06 | 3.86E-05 |
| ENSECAG00000008500 | -3.4839817 | 0.0001254 | 0.00051199 |
| ENSECAG00000024922 | -3.4868781 | 1.80E-06 | 1.18E-05 |
| ENSECAG00000050336 | -3.48905 | 3.80E-07 | 2.93E-06 |
| ENSECAG00000018724 | -3.490881 | 6.93E-06 | 3.89E-05 |
| ENSECAG00000013207 | -3.4911479 | 8.06E-13 | 2.02E-11 |
| ENSECAG00000000972 | -3.4925772 | 1.52E-23 | 1.99E-21 |
| ENSECAG00000054874 | -3.4949094 | 0.00070183 | 0.00231077 |
| ENSECAG00000009139 | -3.4963574 | 1.42E-05 | 7.40E-05 |
| ENSECAG00000038698 | -3.500267 | 5.06E-14 | 1.58E-12 |
| ENSECAG00000009064 | -3.5010845 | 0.00013301 | 0.0005384 |
| ENSECAG00000045278 | -3.5024845 | 8.16E-07 | 5.78E-06 |
| ENSECAG00000037587 | -3.5032588 | 0.00018793 | 0.00072806 |
| ENSECAG00000030609 | -3.5052228 | 1.52E-07 | 1.30E-06 |
| ENSECAG00000039156 | -3.507445 | 0.00026419 | 0.00098608 |
| ENSECAG00000047054 | -3.508756 | 0.01577074 | 0.03454352 |
| ENSECAG00000050106 | -3.5108556 | 9.49E-05 | 0.00040156 |
| ENSECAG00000054202 | -3.5118328 | 3.80E-10 | 5.86E-09 |
| ENSECAG00000031586 | -3.5122532 | 0.00277723 | 0.00771411 |
| ENSECAG00000057997 | -3.5131176 | 1.32E-05 | 6.93E-05 |
| ENSECAG00000017316 | -3.515893 | 6.83E-06 | 3.84E-05 |
| ENSECAG00000046965 | -3.5171771 | 9.41E-05 | 0.00039896 |
| ENSECAG00000053837 | -3.5173088 | 0.00258617 | 0.00726743 |
| ENSECAG00000012819 | -3.5194033 | 0.00146639 | 0.00442331 |
| ENSECAG00000024814 | -3.5201339 | 0.00026753 | 0.00099672 |
| ENSECAG00000058897 | -3.5211074 | 5.02E-07 | 3.77E-06 |
| ENSECAG00000049679 | -3.5220417 | 0.00010501 | 0.00044018 |
| ENSECAG00000051134 | -3.5225784 | 8.22E-06 | 4.53E-05 |
| ENSECAG00000056263 | -3.5228008 | 4.97E-07 | 3.74E-06 |
| ENSECAG00000053096 | -3.5244549 | 2.42E-05 | 0.00011898 |
| ENSECAG00000033446 | -3.5261125 | 2.60E-06 | 1.63E-05 |
| ENSECAG00000046822 | -3.5281014 | 0.00542166 | 0.01382732 |
| ENSECAG00000058652 | -3.5283509 | 7.83E-07 | 5.58E-06 |
| ENSECAG00000015294 | -3.5286779 | 5.36E-06 | 3.09E-05 |
| ENSECAG00000016380 | -3.529698 | 4.62E-05 | 0.00021195 |
| ENSECAG00000051020 | -3.5308464 | 3.88E-08 | 3.80E-07 |
| ENSECAG00000017015 | -3.5372418 | 9.40E-23 | 1.08E-20 |
| ENSECAG00000048445 | -3.5381383 | 0.00043007 | 0.0015056 |
| ENSECAG00000044421 | -3.5389244 | 0.00058929 | 0.00198376 |
| ENSECAG00000047533 | -3.541198 | 0.00227891 | 0.00651877 |
| ENSECAG00000037415 | -3.5465274 | 1.18E-08 | 1.32E-07 |
| ENSECAG00000038089 | -3.551052 | 3.58E-07 | 2.78E-06 |
| ENSECAG00000056801 | -3.5511294 | 3.28E-15 | 1.25E-13 |
| ENSECAG00000048406 | -3.5547496 | 0.01345469 | 0.03009071 |
| ENSECAG00000049903 | -3.560637 | 6.88E-06 | 3.86E-05 |
| ENSECAG00000006873 | -3.5607075 | 2.32E-08 | 2.41E-07 |
| ENSECAG00000015328 | -3.5611414 | 1.69E-06 | 1.11E-05 |
| ENSECAG00000026322 | -3.5622415 | 2.36E-07 | 1.92E-06 |
| ENSECAG00000048621 | -3.5624668 | 0.0015564 | 0.00465463 |
| ENSECAG00000048121 | -3.5661464 | 5.09E-09 | 6.22E-08 |
| ENSECAG00000009451 | -3.5666189 | 2.42E-06 | 1.53E-05 |
| ENSECAG00000049500 | -3.5666657 | 0.00034621 | 0.0012482 |
| ENSECAG00000047618 | -3.5683472 | 1.50E-05 | 7.74E-05 |
| ENSECAG00000051676 | -3.5703943 | 1.02E-15 | 4.19E-14 |
| ENSECAG00000005252 | -3.573554 | 6.93E-10 | 1.02E-08 |
| ENSECAG00000054360 | -3.5765899 | 0.00133913 | 0.00408634 |
| ENSECAG00000039739 | -3.5770019 | 1.24E-08 | 1.38E-07 |
| ENSECAG00000035068 | -3.5775211 | 6.46E-08 | 5.99E-07 |
| ENSECAG00000048836 | -3.5782795 | 0.0001008 | 0.00042434 |
| ENSECAG00000030145 | -3.5795309 | 0.01613666 | 0.03522989 |
| ENSECAG00000037915 | -3.5841978 | 4.47E-05 | 0.00020593 |
| ENSECAG00000029988 | -3.5863641 | 0.01072338 | 0.02480586 |
| ENSECAG00000057098 | -3.5916195 | 1.28E-05 | 6.71E-05 |
| ENSECAG00000007401 | -3.5955899 | 6.83E-05 | 0.0003007 |
| ENSECAG00000024462 | -3.596483 | 4.18E-07 | 3.18E-06 |
| ENSECAG00000016691 | -3.5994279 | 4.06E-25 | 6.92E-23 |
| ENSECAG00000029901 | -3.6015089 | 0.00077535 | 0.00252603 |
| ENSECAG00000024147 | -3.6015745 | 1.50E-07 | 1.28E-06 |
| ENSECAG00000058171 | -3.6018216 | 7.40E-05 | 0.00032338 |
| ENSECAG00000022404 | -3.6038054 | 2.40E-05 | 0.00011776 |
| ENSECAG00000055023 | -3.6049847 | 6.59E-05 | 0.00029098 |
| ENSECAG00000013888 | -3.6061619 | 4.65E-12 | 1.01E-10 |
| ENSECAG00000051722 | -3.6072693 | 2.31E-06 | 1.47E-05 |
| ENSECAG00000039476 | -3.6203028 | 0.00018739 | 0.0007266 |
| ENSECAG00000045256 | -3.6208299 | 2.34E-17 | 1.27E-15 |
| ENSECAG00000021717 | -3.6225641 | 8.10E-09 | 9.40E-08 |
| ENSECAG00000029958 | -3.6227202 | 1.78E-05 | 9.03E-05 |
| ENSECAG00000022683 | -3.622984 | 2.29E-09 | 3.05E-08 |
| ENSECAG00000011415 | -3.623621 | 0.00053663 | 0.00182658 |
| ENSECAG00000035498 | -3.6296927 | 1.37E-08 | 1.50E-07 |
| ENSECAG00000046667 | -3.6324934 | 0.00261193 | 0.00732875 |
| ENSECAG00000059732 | -3.6347414 | 0.00544302 | 0.01387676 |
| ENSECAG00000045556 | -3.6368746 | 0.00012245 | 0.00050169 |
| ENSECAG00000050291 | -3.6377676 | 0.00109659 | 0.00342191 |
| ENSECAG00000056573 | -3.641015 | 4.95E-05 | 0.00022551 |
| ENSECAG00000040130 | -3.6430005 | 2.01E-11 | 3.94E-10 |
| ENSECAG00000014487 | -3.6438817 | 8.60E-12 | 1.80E-10 |
| ENSECAG00000015893 | -3.6447474 | 1.09E-16 | 5.21E-15 |
| ENSECAG00000021316 | -3.6454838 | 3.04E-10 | 4.78E-09 |
| ENSECAG00000021128 | -3.6503005 | 4.80E-06 | 2.80E-05 |
| ENSECAG00000013596 | -3.6507128 | 0.0014363 | 0.00434193 |
| ENSECAG00000023736 | -3.6551386 | 1.46E-08 | 1.59E-07 |
| ENSECAG00000024837 | -3.6559181 | 9.72E-07 | 6.79E-06 |
| ENSECAG00000020271 | -3.6559488 | 9.97E-09 | 1.13E-07 |
| ENSECAG00000028918 | -3.6560352 | 0.00333594 | 0.00906651 |
| ENSECAG00000002256 | -3.6567645 | 0.00014555 | 0.00058359 |
| ENSECAG00000047765 | -3.6580472 | 2.69E-06 | 1.68E-05 |
| ENSECAG00000016576 | -3.6588984 | 1.86E-15 | 7.33E-14 |
| ENSECAG00000018390 | -3.666586 | 0.01418272 | 0.03149741 |
| ENSECAG00000055328 | -3.6678225 | 0.00042807 | 0.00150002 |
| ENSECAG00000005966 | -3.6693866 | 3.88E-06 | 2.32E-05 |
| ENSECAG00000015100 | -3.6714766 | 2.57E-05 | 0.00012548 |
| ENSECAG00000049876 | -3.6730804 | 1.24E-05 | 6.56E-05 |
| ENSECAG00000050023 | -3.6737057 | 4.70E-05 | 0.00021529 |
| ENSECAG00000009483 | -3.6744631 | 6.52E-10 | 9.64E-09 |
| ENSECAG00000010935 | -3.6766714 | 1.92E-06 | 1.25E-05 |
| ENSECAG00000039620 | -3.6823746 | 0.00011959 | 0.00049208 |
| ENSECAG00000052562 | -3.6848924 | 7.31E-07 | 5.24E-06 |
| ENSECAG00000057787 | -3.685724 | 1.89E-07 | 1.58E-06 |
| ENSECAG00000054843 | -3.6878231 | 2.02E-09 | 2.72E-08 |
| ENSECAG00000026967 | -3.6910194 | 2.46E-06 | 1.56E-05 |
| ENSECAG00000012751 | -3.6926372 | 5.67E-16 | 2.39E-14 |
| ENSECAG00000047476 | -3.6949194 | 5.09E-08 | 4.83E-07 |
| ENSECAG00000040211 | -3.6978652 | 2.91E-09 | 3.77E-08 |
| ENSECAG00000015233 | -3.6985122 | 2.71E-13 | 7.41E-12 |
| ENSECAG00000000918 | -3.7028735 | 8.88E-27 | 2.00E-24 |
| ENSECAG00000050172 | -3.7035208 | 0.00983222 | 0.02303774 |
| ENSECAG00000023008 | -3.7079824 | 8.79E-08 | 7.89E-07 |
| ENSECAG00000050668 | -3.7101838 | 5.36E-17 | 2.70E-15 |
| ENSECAG00000017274 | -3.7105877 | 0.00127967 | 0.00392311 |
| ENSECAG00000047819 | -3.7135503 | 4.65E-05 | 0.00021318 |
| ENSECAG00000034093 | -3.7139537 | 3.06E-07 | 2.42E-06 |
| ENSECAG00000007839 | -3.7186154 | 1.29E-07 | 1.12E-06 |
| ENSECAG00000057831 | -3.7210476 | 9.16E-05 | 0.00039002 |
| ENSECAG00000036138 | -3.7277273 | 1.35E-11 | 2.74E-10 |
| ENSECAG00000044909 | -3.7302104 | 7.37E-09 | 8.66E-08 |
| ENSECAG00000003704 | -3.7336761 | 4.17E-20 | 3.49E-18 |
| ENSECAG00000009736 | -3.7357547 | 4.39E-08 | 4.24E-07 |
| ENSECAG00000051466 | -3.7388096 | 9.00E-08 | 8.06E-07 |
| ENSECAG00000041946 | -3.741799 | 0.00023725 | 0.000896 |
| ENSECAG00000051233 | -3.7431156 | 0.00277575 | 0.00771095 |
| ENSECAG00000045717 | -3.7497919 | 0.00011844 | 0.00048804 |
| ENSECAG00000013598 | -3.7589361 | 6.95E-07 | 5.03E-06 |
| ENSECAG00000018944 | -3.7630064 | 0.0112831 | 0.02592085 |
| ENSECAG00000057543 | -3.7630446 | 0.00094542 | 0.00300903 |
| ENSECAG00000012849 | -3.7631534 | 8.18E-05 | 0.00035279 |
| ENSECAG00000052652 | -3.7651061 | 0.021163 | 0.0443962 |
| ENSECAG00000000233 | -3.7665768 | 1.08E-06 | 7.48E-06 |
| ENSECAG00000021901 | -3.7684688 | 4.99E-06 | 2.90E-05 |
| ENSECAG00000024377 | -3.7693187 | 2.70E-10 | 4.30E-09 |
| ENSECAG00000023797 | -3.7708163 | 1.76E-20 | 1.52E-18 |
| ENSECAG00000048035 | -3.7718723 | 0.00698542 | 0.01718742 |
| ENSECAG00000051029 | -3.7749672 | 3.16E-05 | 0.00015068 |
| ENSECAG00000000681 | -3.7771212 | 0.01395007 | 0.03107799 |
| ENSECAG00000054418 | -3.7820949 | 7.88E-06 | 4.37E-05 |
| ENSECAG00000006548 | -3.7868209 | 1.73E-12 | 4.09E-11 |
| ENSECAG00000046588 | -3.7944948 | 0.00066708 | 0.00221133 |
| ENSECAG00000050842 | -3.7963804 | 0.00043435 | 0.00151846 |
| ENSECAG00000015932 | -3.7984748 | 1.26E-05 | 6.62E-05 |
| ENSECAG00000056379 | -3.7994023 | 2.33E-08 | 2.43E-07 |
| ENSECAG00000051319 | -3.8004533 | 6.78E-14 | 2.07E-12 |
| ENSECAG00000015204 | -3.8017832 | 0.00039385 | 0.00139366 |
| ENSECAG00000022616 | -3.806066 | 0.00268862 | 0.00750717 |
| ENSECAG00000011949 | -3.8111292 | 3.15E-06 | 1.93E-05 |
| ENSECAG00000003417 | -3.8129053 | 7.92E-06 | 4.38E-05 |
| ENSECAG00000045160 | -3.8136917 | 1.54E-06 | 1.03E-05 |
| ENSECAG00000004482 | -3.8159957 | 0.00142372 | 0.00431134 |
| ENSECAG00000012551 | -3.8160519 | 2.61E-06 | 1.64E-05 |
| ENSECAG00000050447 | -3.8191625 | 5.55E-11 | 1.01E-09 |
| ENSECAG00000028706 | -3.8230623 | 1.04E-05 | 5.59E-05 |
| ENSECAG00000010276 | -3.8298131 | 6.64E-05 | 0.00029284 |
| ENSECAG00000028110 | -3.8299235 | 0.0008881 | 0.00285002 |
| ENSECAG00000014646 | -3.8357478 | 1.28E-10 | 2.17E-09 |
| ENSECAG00000055271 | -3.8427488 | 2.78E-08 | 2.82E-07 |
| ENSECAG00000047394 | -3.8430148 | 4.35E-08 | 4.21E-07 |
| ENSECAG00000017214 | -3.8465962 | 7.36E-13 | 1.86E-11 |
| ENSECAG00000006620 | -3.8482794 | 0.01797373 | 0.03863048 |
| ENSECAG00000037404 | -3.8515548 | 2.55E-09 | 3.34E-08 |
| ENSECAG00000048869 | -3.8529261 | 1.11E-05 | 5.90E-05 |
| ENSECAG00000008947 | -3.8580497 | 3.93E-06 | 2.35E-05 |
| ENSECAG00000027842 | -3.8615458 | 3.59E-07 | 2.79E-06 |
| ENSECAG00000029770 | -3.8723083 | 2.09E-15 | 8.18E-14 |
| ENSECAG00000006795 | -3.872621 | 3.14E-28 | 8.25E-26 |
| ENSECAG00000015784 | -3.8786884 | 5.80E-06 | 3.32E-05 |
| ENSECAG00000034646 | -3.8825358 | 8.61E-07 | 6.07E-06 |
| ENSECAG00000044582 | -3.8828525 | 0.00027973 | 0.00103507 |
| ENSECAG00000034035 | -3.8917006 | 0.00012542 | 0.000512 |
| ENSECAG00000001540 | -3.9035585 | 0.00556273 | 0.01413358 |
| ENSECAG00000000527 | -3.9046129 | 0.00262541 | 0.00736287 |
| ENSECAG00000031280 | -3.9054135 | 2.61E-06 | 1.64E-05 |
| ENSECAG00000033081 | -3.9104082 | 3.41E-06 | 2.07E-05 |
| ENSECAG00000046925 | -3.9113832 | 1.80E-06 | 1.18E-05 |
| ENSECAG00000000862 | -3.9120861 | 6.59E-14 | 2.01E-12 |
| ENSECAG00000059215 | -3.9122442 | 1.04E-06 | 7.19E-06 |
| ENSECAG00000027949 | -3.9141959 | 2.86E-06 | 1.77E-05 |
| ENSECAG00000058006 | -3.9148995 | 6.16E-12 | 1.32E-10 |
| ENSECAG00000010614 | -3.916266 | 8.09E-06 | 4.46E-05 |
| ENSECAG00000007939 | -3.9204569 | 8.05E-23 | 9.37E-21 |
| ENSECAG00000055374 | -3.9254744 | 3.12E-06 | 1.92E-05 |
| ENSECAG00000058689 | -3.9292271 | 5.09E-08 | 4.83E-07 |
| ENSECAG00000036379 | -3.9298094 | 3.60E-25 | 6.29E-23 |
| ENSECAG00000035724 | -3.9335244 | 1.12E-06 | 7.72E-06 |
| ENSECAG00000023206 | -3.9408591 | 4.00E-05 | 0.00018651 |
| ENSECAG00000006027 | -3.9431762 | 2.15E-16 | 9.78E-15 |
| ENSECAG00000033314 | -3.9449598 | 0.00020746 | 0.00079557 |
| ENSECAG00000059419 | -3.9491609 | 5.87E-11 | 1.06E-09 |
| ENSECAG00000056631 | -3.9519274 | 0.00133998 | 0.00408803 |
| ENSECAG00000010587 | -3.9566772 | 8.10E-31 | 3.07E-28 |
| ENSECAG00000031648 | -3.9590096 | 4.24E-13 | 1.13E-11 |
| ENSECAG00000012775 | -3.9640666 | 3.58E-05 | 0.0001687 |
| ENSECAG00000055670 | -3.9758363 | 1.69E-10 | 2.79E-09 |
| ENSECAG00000057278 | -3.9796376 | 5.61E-07 | 4.16E-06 |
| ENSECAG00000044284 | -3.9837967 | 7.78E-07 | 5.54E-06 |
| ENSECAG00000043452 | -3.9846846 | 0.01845855 | 0.03952423 |
| ENSECAG00000055588 | -3.9951705 | 1.45E-08 | 1.58E-07 |
| ENSECAG00000047388 | -3.9966835 | 0.00400265 | 0.01061639 |
| ENSECAG00000051666 | -3.9970498 | 3.17E-05 | 0.00015138 |
| ENSECAG00000038799 | -4.0003853 | 3.80E-05 | 0.00017781 |
| ENSECAG00000019808 | -4.0045291 | 0.00077334 | 0.00252019 |
| ENSECAG00000046564 | -4.0061881 | 2.76E-10 | 4.38E-09 |
| ENSECAG00000011573 | -4.008312 | 2.30E-09 | 3.05E-08 |
| ENSECAG00000052961 | -4.0127933 | 2.62E-06 | 1.64E-05 |
| ENSECAG00000039617 | -4.0143429 | 1.63E-14 | 5.50E-13 |
| ENSECAG00000037444 | -4.0319789 | 0.00235312 | 0.00669588 |
| ENSECAG00000011575 | -4.0330488 | 7.74E-17 | 3.77E-15 |
| ENSECAG00000013831 | -4.0332306 | 0.00032462 | 0.00118084 |
| ENSECAG00000017764 | -4.0347338 | 1.54E-05 | 7.92E-05 |
| ENSECAG00000008203 | -4.0363844 | 5.18E-09 | 6.30E-08 |
| ENSECAG00000052911 | -4.0401317 | 0.00010379 | 0.00043562 |
| ENSECAG00000038788 | -4.0404072 | 4.99E-08 | 4.75E-07 |
| ENSECAG00000043861 | -4.0420598 | 1.29E-06 | 8.73E-06 |
| ENSECAG00000045553 | -4.0464298 | 1.40E-07 | 1.21E-06 |
| ENSECAG00000034050 | -4.0510805 | 0.0051036 | 0.01311918 |
| ENSECAG00000044556 | -4.0516524 | 9.34E-13 | 2.32E-11 |
| ENSECAG00000057027 | -4.0536509 | 8.78E-08 | 7.89E-07 |
| ENSECAG00000029039 | -4.054771 | 0.00010018 | 0.00042199 |
| ENSECAG00000011961 | -4.05997 | 1.19E-08 | 1.32E-07 |
| ENSECAG00000000769 | -4.0608358 | 3.33E-05 | 0.00015829 |
| ENSECAG00000012395 | -4.0635777 | 3.70E-08 | 3.64E-07 |
| ENSECAG00000046781 | -4.064572 | 1.38E-12 | 3.33E-11 |
| ENSECAG00000049359 | -4.0709561 | 6.16E-06 | 3.50E-05 |
| ENSECAG00000020422 | -4.0739132 | 0.00181218 | 0.00532689 |
| ENSECAG00000046632 | -4.0746852 | 1.69E-10 | 2.79E-09 |
| ENSECAG00000057041 | -4.076129 | 6.57E-05 | 0.00029039 |
| ENSECAG00000033098 | -4.076338 | 0.01168365 | 0.02671199 |
| ENSECAG00000012656 | -4.0829888 | 2.75E-07 | 2.20E-06 |
| ENSECAG00000006143 | -4.0878198 | 1.03E-13 | 2.99E-12 |
| ENSECAG00000050892 | -4.0918024 | 0.00015808 | 0.00062733 |
| ENSECAG00000051751 | -4.0936141 | 0.00764578 | 0.01856892 |
| ENSECAG00000016639 | -4.0943223 | 0.00105337 | 0.00330505 |
| ENSECAG00000034457 | -4.099543 | 2.92E-11 | 5.61E-10 |
| ENSECAG00000030850 | -4.1046049 | 1.30E-17 | 7.36E-16 |
| ENSECAG00000016572 | -4.1065341 | 2.98E-08 | 3.01E-07 |
| ENSECAG00000046673 | -4.1171683 | 0.0016269 | 0.00483718 |
| ENSECAG00000035069 | -4.1231134 | 0.00240634 | 0.00682214 |
| ENSECAG00000052178 | -4.126927 | 0.00141592 | 0.00428988 |
| ENSECAG00000017600 | -4.1278639 | 3.22E-06 | 1.97E-05 |
| ENSECAG00000010427 | -4.1284697 | 0.00082422 | 0.00266891 |
| ENSECAG00000008473 | -4.1290957 | 0.00277288 | 0.00770491 |
| ENSECAG00000057809 | -4.1328869 | 1.72E-06 | 1.13E-05 |
| ENSECAG00000043819 | -4.1329089 | 4.51E-11 | 8.38E-10 |
| ENSECAG00000019320 | -4.1371247 | 0.00024073 | 0.00090684 |
| ENSECAG00000000649 | -4.143882 | 1.32E-19 | 1.03E-17 |
| ENSECAG00000053284 | -4.1450901 | 4.60E-07 | 3.49E-06 |
| ENSECAG00000033216 | -4.1487046 | 0.00021334 | 0.00081577 |
| ENSECAG00000022132 | -4.150435 | 0.00021774 | 0.00083015 |
| ENSECAG00000024477 | -4.1536166 | 2.48E-08 | 2.56E-07 |
| ENSECAG00000047135 | -4.1576327 | 1.60E-05 | 8.23E-05 |
| ENSECAG00000024183 | -4.1592044 | 7.43E-08 | 6.79E-07 |
| ENSECAG00000058422 | -4.1651165 | 6.73E-07 | 4.88E-06 |
| ENSECAG00000003439 | -4.1653219 | 2.68E-08 | 2.74E-07 |
| ENSECAG00000057791 | -4.1665418 | 0.00102685 | 0.00323841 |
| ENSECAG00000022739 | -4.1713471 | 5.56E-07 | 4.13E-06 |
| ENSECAG00000046915 | -4.1762275 | 1.20E-09 | 1.68E-08 |
| ENSECAG00000015669 | -4.1840157 | 2.30E-12 | 5.31E-11 |
| ENSECAG00000058476 | -4.184587 | 2.62E-06 | 1.64E-05 |
| ENSECAG00000047738 | -4.203204 | 6.10E-20 | 5.01E-18 |
| ENSECAG00000049196 | -4.2056662 | 2.27E-09 | 3.03E-08 |
| ENSECAG00000051909 | -4.2060167 | 0.00075652 | 0.00247116 |
| ENSECAG00000059356 | -4.2070573 | 3.17E-16 | 1.40E-14 |
| ENSECAG00000041013 | -4.2081768 | 0.00296223 | 0.00816304 |
| ENSECAG00000046326 | -4.2089232 | 3.70E-08 | 3.64E-07 |
| ENSECAG00000030004 | -4.2158895 | 1.56E-05 | 8.04E-05 |
| ENSECAG00000029135 | -4.2179567 | 2.72E-05 | 0.00013199 |
| ENSECAG00000032823 | -4.2349331 | 1.22E-06 | 8.32E-06 |
| ENSECAG00000024893 | -4.2359375 | 1.96E-07 | 1.62E-06 |
| ENSECAG00000058083 | -4.2392889 | 1.99E-05 | 9.97E-05 |
| ENSECAG00000050806 | -4.2510246 | 2.73E-06 | 1.70E-05 |
| ENSECAG00000015092 | -4.2527413 | 0.00032871 | 0.0011929 |
| ENSECAG00000006816 | -4.2691303 | 0.00127081 | 0.00389807 |
| ENSECAG00000033181 | -4.2702924 | 6.93E-06 | 3.89E-05 |
| ENSECAG00000058745 | -4.2716514 | 2.71E-06 | 1.69E-05 |
| ENSECAG00000022234 | -4.2717882 | 1.12E-05 | 5.96E-05 |
| ENSECAG00000015072 | -4.2726517 | 0.00107105 | 0.0033511 |
| ENSECAG00000010056 | -4.2751642 | 5.88E-14 | 1.81E-12 |
| ENSECAG00000050413 | -4.2783358 | 0.00038539 | 0.00136697 |
| ENSECAG00000046530 | -4.279614 | 8.43E-11 | 1.49E-09 |
| ENSECAG00000050546 | -4.2842022 | 4.90E-07 | 3.69E-06 |
| ENSECAG00000026855 | -4.2854061 | 7.43E-09 | 8.72E-08 |
| ENSECAG00000052034 | -4.2864903 | 0.00090703 | 0.00290254 |
| ENSECAG00000007354 | -4.2971895 | 8.21E-05 | 0.00035381 |
| ENSECAG00000046146 | -4.3021934 | 3.19E-09 | 4.08E-08 |
| ENSECAG00000055078 | -4.3105671 | 0.00348279 | 0.00940614 |
| ENSECAG00000046403 | -4.3126675 | 0.00017865 | 0.00069744 |
| ENSECAG00000012284 | -4.3148232 | 8.62E-06 | 4.72E-05 |
| ENSECAG00000015255 | -4.3184171 | 1.12E-05 | 5.97E-05 |
| ENSECAG00000020439 | -4.3204384 | 0.00163553 | 0.00486133 |
| ENSECAG00000016679 | -4.3297626 | 5.61E-13 | 1.46E-11 |
| ENSECAG00000050567 | -4.333724 | 3.41E-05 | 0.00016172 |
| ENSECAG00000016114 | -4.3342342 | 3.93E-15 | 1.47E-13 |
| ENSECAG00000047019 | -4.3343668 | 6.65E-07 | 4.83E-06 |
| ENSECAG00000029689 | -4.3347452 | 3.71E-11 | 7.00E-10 |
| ENSECAG00000043355 | -4.3395777 | 4.19E-06 | 2.48E-05 |
| ENSECAG00000054788 | -4.3398706 | 8.39E-06 | 4.62E-05 |
| ENSECAG00000014778 | -4.3479491 | 4.68E-07 | 3.54E-06 |
| ENSECAG00000050669 | -4.3492394 | 9.56E-05 | 0.00040403 |
| ENSECAG00000006269 | -4.3536583 | 7.43E-13 | 1.88E-11 |
| ENSECAG00000045612 | -4.3544848 | 1.73E-09 | 2.36E-08 |
| ENSECAG00000043870 | -4.3631544 | 6.09E-08 | 5.68E-07 |
| ENSECAG00000057209 | -4.3882268 | 0.0007986 | 0.00259376 |
| ENSECAG00000000408 | -4.3915995 | 0.00016161 | 0.0006393 |
| ENSECAG00000005999 | -4.396252 | 4.51E-06 | 2.65E-05 |
| ENSECAG00000053471 | -4.397663 | 1.18E-25 | 2.26E-23 |
| ENSECAG00000060032 | -4.4039298 | 5.50E-07 | 4.10E-06 |
| ENSECAG00000026067 | -4.4041798 | 1.78E-15 | 7.04E-14 |
| ENSECAG00000006510 | -4.405356 | 8.73E-09 | 1.00E-07 |
| ENSECAG00000053970 | -4.4063684 | 2.41E-11 | 4.67E-10 |
| ENSECAG00000051385 | -4.4107698 | 2.30E-10 | 3.72E-09 |
| ENSECAG00000045894 | -4.4148189 | 0.00094272 | 0.00300298 |
| ENSECAG00000055451 | -4.4185105 | 0.0004705 | 0.00162826 |
| ENSECAG00000025897 | -4.4227802 | 1.45E-06 | 9.73E-06 |
| ENSECAG00000021063 | -4.4282532 | 1.06E-11 | 2.18E-10 |
| ENSECAG00000054415 | -4.4302184 | 3.16E-06 | 1.94E-05 |
| ENSECAG00000010460 | -4.4330855 | 0.00053487 | 0.00182171 |
| ENSECAG00000006102 | -4.4338802 | 3.48E-05 | 0.00016455 |
| ENSECAG00000010979 | -4.4375055 | 5.56E-07 | 4.13E-06 |
| ENSECAG00000054608 | -4.438194 | 0.00010125 | 0.0004261 |
| ENSECAG00000007161 | -4.4400553 | 1.97E-05 | 9.92E-05 |
| ENSECAG00000006970 | -4.4425301 | 0.00017096 | 0.00067104 |
| ENSECAG00000018282 | -4.4472851 | 0.00012255 | 0.00050204 |
| ENSECAG00000009405 | -4.4515971 | 3.80E-14 | 1.21E-12 |
| ENSECAG00000057201 | -4.4566252 | 2.12E-05 | 0.00010547 |
| ENSECAG00000033315 | -4.4592151 | 0.00046735 | 0.00161862 |
| ENSECAG00000052907 | -4.4597901 | 3.90E-05 | 0.0001823 |
| ENSECAG00000051956 | -4.4601474 | 4.85E-13 | 1.27E-11 |
| ENSECAG00000009466 | -4.4636072 | 3.00E-07 | 2.37E-06 |
| ENSECAG00000048997 | -4.4687427 | 0.0049467 | 0.01277317 |
| ENSECAG00000035840 | -4.4699726 | 0.00084657 | 0.00273255 |
| ENSECAG00000011748 | -4.4902235 | 2.22E-25 | 4.04E-23 |
| ENSECAG00000006499 | -4.4932641 | 1.75E-14 | 5.84E-13 |
| ENSECAG00000036062 | -4.4982548 | 7.50E-09 | 8.80E-08 |
| ENSECAG00000029359 | -4.5022051 | 2.47E-06 | 1.56E-05 |
| ENSECAG00000051844 | -4.5034519 | 0.00022413 | 0.00085264 |
| ENSECAG00000010887 | -4.5059402 | 5.09E-14 | 1.59E-12 |
| ENSECAG00000047312 | -4.5159129 | 0.00046865 | 0.00162264 |
| ENSECAG00000021577 | -4.5177796 | 6.07E-15 | 2.20E-13 |
| ENSECAG00000011028 | -4.5206249 | 2.95E-09 | 3.80E-08 |
| ENSECAG00000051996 | -4.5247537 | 2.23E-05 | 0.00011024 |
| ENSECAG00000051152 | -4.5253426 | 1.84E-09 | 2.50E-08 |
| ENSECAG00000052678 | -4.527078 | 6.91E-06 | 3.88E-05 |
| ENSECAG00000013430 | -4.5474589 | 2.49E-07 | 2.01E-06 |
| ENSECAG00000051490 | -4.5548207 | 0.00018708 | 0.00072553 |
| ENSECAG00000040082 | -4.561357 | 1.84E-09 | 2.50E-08 |
| ENSECAG00000000714 | -4.5652563 | 6.61E-06 | 3.73E-05 |
| ENSECAG00000006257 | -4.566235 | 6.15E-13 | 1.59E-11 |
| ENSECAG00000049807 | -4.5677299 | 3.08E-08 | 3.09E-07 |
| ENSECAG00000052813 | -4.5723296 | 1.51E-05 | 7.80E-05 |
| ENSECAG00000046720 | -4.5782171 | 1.19E-07 | 1.04E-06 |
| ENSECAG00000045399 | -4.5791329 | 0.02310752 | 0.04781118 |
| ENSECAG00000000087 | -4.600358 | 5.03E-05 | 0.0002289 |
| ENSECAG00000012063 | -4.6104459 | 0.00069449 | 0.00229133 |
| ENSECAG00000023997 | -4.6171159 | 5.23E-17 | 2.64E-15 |
| ENSECAG00000023439 | -4.6276071 | 2.58E-06 | 1.62E-05 |
| ENSECAG00000004805 | -4.6367206 | 3.59E-14 | 1.14E-12 |
| ENSECAG00000054437 | -4.641418 | 0.00014734 | 0.00059004 |
| ENSECAG00000043059 | -4.6417064 | 7.65E-09 | 8.94E-08 |
| ENSECAG00000041919 | -4.6459811 | 1.70E-08 | 1.83E-07 |
| ENSECAG00000036742 | -4.6540862 | 0.00069677 | 0.00229715 |
| ENSECAG00000058659 | -4.6669803 | 7.44E-06 | 4.15E-05 |
| ENSECAG00000055222 | -4.695132 | 1.32E-06 | 8.97E-06 |
| ENSECAG00000060324 | -4.6953374 | 0.00015643 | 0.00062111 |
| ENSECAG00000034320 | -4.6962013 | 4.48E-05 | 0.00020644 |
| ENSECAG00000014951 | -4.6966435 | 1.42E-07 | 1.22E-06 |
| ENSECAG00000029384 | -4.6966497 | 0.00022067 | 0.00084063 |
| ENSECAG00000049934 | -4.6973677 | 6.29E-05 | 0.00027971 |
| ENSECAG00000045919 | -4.6997624 | 1.13E-10 | 1.96E-09 |
| ENSECAG00000010498 | -4.7051037 | 5.03E-05 | 0.00022878 |
| ENSECAG00000036496 | -4.7160032 | 4.02E-06 | 2.39E-05 |
| ENSECAG00000045846 | -4.7272872 | 0.00230435 | 0.00657895 |
| ENSECAG00000048920 | -4.7439412 | 0.00196381 | 0.00572291 |
| ENSECAG00000028394 | -4.7530954 | 0.00076243 | 0.00248863 |
| ENSECAG00000036987 | -4.7574821 | 2.87E-05 | 0.00013828 |
| ENSECAG00000048692 | -4.7782309 | 1.02E-07 | 9.05E-07 |
| ENSECAG00000013941 | -4.7874545 | 1.88E-08 | 2.01E-07 |
| ENSECAG00000045145 | -4.8029383 | 7.45E-08 | 6.80E-07 |
| ENSECAG00000011682 | -4.8267138 | 1.61E-05 | 8.26E-05 |
| ENSECAG00000000719 | -4.8345455 | 2.00E-10 | 3.26E-09 |
| ENSECAG00000051885 | -4.8457667 | 0.00454484 | 0.01185882 |
| ENSECAG00000034392 | -4.8548536 | 2.21E-05 | 0.00010978 |
| ENSECAG00000040341 | -4.8656838 | 4.29E-05 | 0.00019876 |
| ENSECAG00000035776 | -4.8875323 | 0.00013735 | 0.00055373 |
| ENSECAG00000023678 | -4.8919709 | 4.03E-09 | 5.04E-08 |
| ENSECAG00000051836 | -4.9207922 | 2.94E-08 | 2.97E-07 |
| ENSECAG00000011971 | -4.9309127 | 2.27E-09 | 3.03E-08 |
| ENSECAG00000044993 | -4.937595 | 6.60E-09 | 7.85E-08 |
| ENSECAG00000011561 | -4.9435559 | 9.28E-08 | 8.28E-07 |
| ENSECAG00000045432 | -4.9706695 | 1.39E-14 | 4.72E-13 |
| ENSECAG00000040431 | -4.9720292 | 5.16E-09 | 6.29E-08 |
| ENSECAG00000060274 | -4.9819758 | 7.96E-05 | 0.00034479 |
| ENSECAG00000036200 | -4.9840153 | 1.26E-16 | 5.91E-15 |
| ENSECAG00000049193 | -4.9998155 | 2.64E-09 | 3.45E-08 |
| ENSECAG00000042866 | -5.0004422 | 5.33E-09 | 6.46E-08 |
| ENSECAG00000007171 | -5.0210036 | 1.27E-10 | 2.16E-09 |
| ENSECAG00000000336 | -5.021642 | 3.03E-10 | 4.77E-09 |
| ENSECAG00000007982 | -5.0219586 | 0.00076557 | 0.00249707 |
| ENSECAG00000007246 | -5.0440498 | 2.06E-05 | 0.00010301 |
| ENSECAG00000001890 | -5.0452558 | 1.49E-07 | 1.28E-06 |
| ENSECAG00000054211 | -5.0477443 | 1.50E-07 | 1.28E-06 |
| ENSECAG00000022713 | -5.0565159 | 8.26E-05 | 0.00035588 |
| ENSECAG00000037073 | -5.0631625 | 5.09E-05 | 0.00023135 |
| ENSECAG00000039036 | -5.0652956 | 2.69E-06 | 1.68E-05 |
| ENSECAG00000043069 | -5.0785679 | 0.00011607 | 0.00047934 |
| ENSECAG00000015117 | -5.0909182 | 0.00031376 | 0.00114545 |
| ENSECAG00000016275 | -5.1010662 | 1.82E-07 | 1.53E-06 |
| ENSECAG00000048309 | -5.1225401 | 5.05E-09 | 6.18E-08 |
| ENSECAG00000010879 | -5.1314249 | 5.25E-08 | 4.98E-07 |
| ENSECAG00000034517 | -5.1360258 | 4.61E-06 | 2.70E-05 |
| ENSECAG00000048081 | -5.1462161 | 8.58E-05 | 0.00036806 |
| ENSECAG00000039616 | -5.150938 | 7.65E-07 | 5.47E-06 |
| ENSECAG00000060056 | -5.1531062 | 8.14E-06 | 4.49E-05 |
| ENSECAG00000044983 | -5.1538641 | 0.00093698 | 0.00298641 |
| ENSECAG00000039858 | -5.1573948 | 4.41E-05 | 0.00020359 |
| ENSECAG00000035661 | -5.1830328 | 2.04E-06 | 1.31E-05 |
| ENSECAG00000049655 | -5.193942 | 3.81E-08 | 3.74E-07 |
| ENSECAG00000014337 | -5.1984572 | 1.65E-13 | 4.68E-12 |
| ENSECAG00000045076 | -5.215626 | 3.59E-15 | 1.36E-13 |
| ENSECAG00000047284 | -5.2236505 | 1.67E-15 | 6.66E-14 |
| ENSECAG00000045582 | -5.2541153 | 1.53E-07 | 1.31E-06 |
| ENSECAG00000001503 | -5.2571723 | 4.78E-10 | 7.23E-09 |
| ENSECAG00000024445 | -5.2593945 | 6.19E-20 | 5.07E-18 |
| ENSECAG00000048059 | -5.2801823 | 8.41E-07 | 5.95E-06 |
| ENSECAG00000055784 | -5.299116 | 1.19E-06 | 8.18E-06 |
| ENSECAG00000018484 | -5.3152659 | 1.92E-06 | 1.25E-05 |
| ENSECAG00000051879 | -5.3194149 | 0.00065399 | 0.00217277 |
| ENSECAG00000013296 | -5.3242379 | 9.45E-05 | 0.00040035 |
| ENSECAG00000054222 | -5.3493565 | 1.19E-05 | 6.28E-05 |
| ENSECAG00000009662 | -5.3650272 | 1.15E-14 | 3.95E-13 |
| ENSECAG00000058527 | -5.3663368 | 0.00199921 | 0.00581699 |
| ENSECAG00000044498 | -5.3735941 | 1.21E-07 | 1.06E-06 |
| ENSECAG00000031657 | -5.3791879 | 2.37E-10 | 3.82E-09 |
| ENSECAG00000049937 | -5.3947827 | 2.92E-11 | 5.61E-10 |
| ENSECAG00000013767 | -5.4020817 | 3.43E-05 | 0.00016232 |
| ENSECAG00000015178 | -5.4384147 | 1.94E-05 | 9.74E-05 |
| ENSECAG00000050938 | -5.4384864 | 4.87E-10 | 7.36E-09 |
| ENSECAG00000050449 | -5.4593219 | 0.00024511 | 0.00092162 |
| ENSECAG00000049656 | -5.4668511 | 3.41E-06 | 2.07E-05 |
| ENSECAG00000049646 | -5.4758948 | 6.29E-10 | 9.33E-09 |
| ENSECAG00000048880 | -5.4971796 | 3.08E-05 | 0.00014757 |
| ENSECAG00000054306 | -5.5263512 | 4.96E-07 | 3.73E-06 |
| ENSECAG00000035079 | -5.5347894 | 7.56E-06 | 4.20E-05 |
| ENSECAG00000057616 | -5.5388563 | 2.32E-07 | 1.89E-06 |
| ENSECAG00000030012 | -5.5440869 | 2.43E-10 | 3.90E-09 |
| ENSECAG00000046727 | -5.5574544 | 8.87E-10 | 1.27E-08 |
| ENSECAG00000045648 | -5.5716745 | 2.37E-05 | 0.00011685 |
| ENSECAG00000001908 | -5.5767854 | 6.14E-11 | 1.11E-09 |
| ENSECAG00000049360 | -5.6122387 | 0.00012023 | 0.00049451 |
| ENSECAG00000016151 | -5.6191848 | 1.38E-15 | 5.58E-14 |
| ENSECAG00000052559 | -5.623273 | 0.00011065 | 0.00046077 |
| ENSECAG00000002951 | -5.6301924 | 1.64E-05 | 8.39E-05 |
| ENSECAG00000042559 | -5.6366486 | 9.77E-06 | 5.30E-05 |
| ENSECAG00000048813 | -5.6380076 | 3.70E-07 | 2.86E-06 |
| ENSECAG00000053564 | -5.6403446 | 3.69E-10 | 5.70E-09 |
| ENSECAG00000043273 | -5.654256 | 0.00071881 | 0.0023597 |
| ENSECAG00000035263 | -5.6564339 | 3.63E-09 | 4.58E-08 |
| ENSECAG00000020100 | -5.6586139 | 2.01E-13 | 5.60E-12 |
| ENSECAG00000056584 | -5.678669 | 1.27E-06 | 8.65E-06 |
| ENSECAG00000048751 | -5.6895657 | 2.70E-09 | 3.52E-08 |
| ENSECAG00000029396 | -5.718295 | 2.00E-05 | 0.00010045 |
| ENSECAG00000043910 | -5.7275209 | 4.72E-07 | 3.57E-06 |
| ENSECAG00000036932 | -5.740868 | 5.48E-08 | 5.17E-07 |
| ENSECAG00000051092 | -5.7417693 | 3.44E-05 | 0.00016263 |
| ENSECAG00000051794 | -5.7639331 | 0.0001849 | 0.0007187 |
| ENSECAG00000008818 | -5.786768 | 6.13E-11 | 1.11E-09 |
| ENSECAG00000010000 | -5.7880979 | 2.88E-15 | 1.11E-13 |
| ENSECAG00000044231 | -5.789782 | 5.94E-05 | 0.00026531 |
| ENSECAG00000056425 | -5.7911311 | 9.49E-05 | 0.00040156 |
| ENSECAG00000052349 | -5.8016289 | 1.34E-05 | 7.02E-05 |
| ENSECAG00000047917 | -5.8035389 | 2.84E-10 | 4.48E-09 |
| ENSECAG00000047984 | -5.8270781 | 0.00036195 | 0.00129595 |
| ENSECAG00000049300 | -5.8629817 | 2.39E-08 | 2.48E-07 |
| ENSECAG00000035272 | -5.8817136 | 1.52E-06 | 1.01E-05 |
| ENSECAG00000055127 | -5.8877513 | 1.92E-08 | 2.04E-07 |
| ENSECAG00000041688 | -5.8951502 | 4.02E-10 | 6.16E-09 |
| ENSECAG00000060097 | -5.9090606 | 0.01048384 | 0.02435258 |
| ENSECAG00000024521 | -5.93226 | 9.18E-08 | 8.20E-07 |
| ENSECAG00000049803 | -5.9576417 | 1.27E-11 | 2.59E-10 |
| ENSECAG00000051539 | -5.9825562 | 1.60E-11 | 3.22E-10 |
| ENSECAG00000001002 | -6.0707007 | 3.37E-15 | 1.28E-13 |
| ENSECAG00000043555 | -6.089481 | 1.50E-08 | 1.62E-07 |
| ENSECAG00000058842 | -6.0901445 | 5.00E-15 | 1.84E-13 |
| ENSECAG00000015877 | -6.1133985 | 7.59E-08 | 6.92E-07 |
| ENSECAG00000008543 | -6.1378705 | 1.32E-09 | 1.83E-08 |
| ENSECAG00000053613 | -6.1424019 | 4.17E-11 | 7.79E-10 |
| ENSECAG00000052443 | -6.156057 | 1.48E-06 | 9.91E-06 |
| ENSECAG00000049237 | -6.1770541 | 8.53E-12 | 1.79E-10 |
| ENSECAG00000034958 | -6.1813095 | 1.21E-09 | 1.69E-08 |
| ENSECAG00000022198 | -6.1921989 | 2.21E-08 | 2.31E-07 |
| ENSECAG00000040822 | -6.2097907 | 0.00030486 | 0.00111644 |
| ENSECAG00000010697 | -6.2136205 | 3.16E-06 | 1.94E-05 |
| ENSECAG00000001081 | -6.2285837 | 1.65E-06 | 1.09E-05 |
| ENSECAG00000012049 | -6.2392951 | 9.75E-08 | 8.66E-07 |
| ENSECAG00000048531 | -6.2729323 | 1.09E-09 | 1.54E-08 |
| ENSECAG00000059966 | -6.3315742 | 5.28E-06 | 3.05E-05 |
| ENSECAG00000024936 | -6.3777946 | 9.78E-09 | 1.11E-07 |
| ENSECAG00000004201 | -6.4061779 | 2.77E-06 | 1.73E-05 |
| ENSECAG00000060113 | -6.4445447 | 1.76E-09 | 2.40E-08 |
| ENSECAG00000053836 | -6.4462372 | 3.33E-09 | 4.23E-08 |
| ENSECAG00000052248 | -6.4662921 | 2.48E-06 | 1.56E-05 |
| ENSECAG00000034235 | -6.4894142 | 6.53E-08 | 6.05E-07 |
| ENSECAG00000047459 | -6.5372854 | 2.90E-09 | 3.75E-08 |
| ENSECAG00000050619 | -6.7100054 | 0.00040898 | 0.00143991 |
| ENSECAG00000002228 | -6.8228303 | 7.08E-10 | 1.04E-08 |
| ENSECAG00000046284 | -6.8599111 | 1.66E-15 | 6.62E-14 |
| ENSECAG00000028903 | -6.9901021 | 2.49E-12 | 5.70E-11 |
| ENSECAG00000058138 | -7.0445323 | 7.47E-11 | 1.33E-09 |
| ENSECAG00000036708 | -7.0565595 | 4.55E-13 | 1.20E-11 |
| ENSECAG00000059472 | -7.0690253 | 1.93E-18 | 1.23E-16 |
| ENSECAG00000060202 | -7.2326224 | 4.12E-05 | 0.00019159 |
| ENSECAG00000010565 | -7.4930935 | 3.35E-19 | 2.45E-17 |
| ENSECAG00000044821 | -7.521163 | 1.16E-16 | 5.50E-15 |
| ENSECAG00000015736 | -7.5958989 | 7.30E-10 | 1.06E-08 |
| ENSECAG00000022852 | -7.7556143 | 5.62E-06 | 3.22E-05 |
| ENSECAG00000012299 | -7.7864294 | 4.89E-11 | 9.04E-10 |
| ENSECAG00000000182 | -8.0015094 | 1.41E-12 | 3.40E-11 |
| ENSECAG00000020648 | -8.4321626 | 3.81E-18 | 2.33E-16 |
| ENSECAG00000002542 | -8.5173182 | 2.03E-14 | 6.72E-13 |
| ENSECAG00000050616 | -8.6654874 | 3.42E-12 | 7.63E-11 |
| ENSECAG00000029574 | -9.6828414 | 1.55E-20 | 1.34E-18 |
| ENSECAG00000028328 | -22.247708 | 6.86E-14 | 2.09E-12 |

Supplementary Table S6. Term Category retrieved with Gene Ontology (GO) enrichment analyses in the category “Molecular function ” for genes found to be up-regulated in the sarcoid group compared to the control group

| **Descrizione** | **FDR** | **Geni background** | **Geni**  **UP-regolati** |
| --- | --- | --- | --- |
| Signaling receptor binding | 1.75E-19 | 1421 | 54 |
| Cytokine activity | 1.91E-14 | 228 | 23 |
| Glycosaminoglycan binding | 2.85E-10 | 218 | 19 |
| Peptidase regulator activity | 8.68E-10 | 201 | 18 |
| Collagen binding | 3.98E-09 | 62 | 12 |
| Growth factor activity | 6.81E-09 | 140 | 15 |
| Growth factor binding | 2.14E-06 | 117 | 12 |
| Metallopeptidase activity | 4.03E-05 | 193 | 13 |
| Cytokine receptor binding | 0.000179 | 222 | 13 |
| Platelet-derived growth factor binding | 0.000256 | 8 | 5 |
| Carboxypeptidase activity | 0.0014 | 44 | 6 |
| Insulin-like growth factor I binding | 0.0021 | 10 | 4 |
| Protein-lysine 6-oxidase activity | 0.00315 | 5 | 4 |
| Chemoattractant activity | 0.0059 | 33 | 5 |
| Integrin binding | 0.0092 | 100 | 7 |
| Chemokine activity | 0.00968 | 41 | 6 |
| ECM-receptor interaction | 5.46E-06 | 101 | 11 |
| AGE-RAGE signaling pathway in diabetic complications | 0.00111 | 105 | 9 |

Supplementaary Table S7. Term Category retrieved with Gene Ontology (GO) enrichment analyses in the category “Molecular function ” for genes found to be down-regulated in the sarcoid group compared to the control group

| **Descrizione** | **FDR** | **Geni background** | **Geni**  **UP-regolati** |
| --- | --- | --- | --- |
| Channel activity | 7.39E-10 | 459 | 88 |
| Metal ion transmembrane transporter activity | 6.01E-08 | 417 | 76 |
| Calcium ion binding | 3.11E-05 | 636 | 91 |
| Sodium ion transmembrane transporter activity | 0.00136 | 148 | 33 |
| Ligand-gated ion channel activity | 0.0019 | 129 | 28 |
| Active ion transmembrane transporter activity | 0.0069 | 219 | 37 |
| Arachidonic acid metabolism | 0.00648 | 143 | 31 |
| Neuroactive ligand-receptor interaction | 0.00648 | 397 | 60 |

Supplementary table S8. DEGs of DE miRNA-DEG couples (up-regulated miRNAs - down-regulated targets) selected for KEGG pathways enrichment analysis.

| Binding site | DOWN-regulated Target | miRNA hits | UP-regulated miRNAs |
| --- | --- | --- | --- |
| 3-prime UTR | ABCC11 | 10 | miR-1197;miR-216b-5p;miR-382-5p;miR-431-3p;miR-485-5p;miR-487a-3p;miR-493-3p;miR-615-3p;miR-758-3p;miR-92b-3p |
|  | ABHD2 | 12 | miR-1193;miR-1197;miR-134-5p;miR-216b-5p;miR-329-3p;miR-370-3p;miR-377-3p;miR-379-5p;miR-381-3p;miR-487b-3p;miR-493-3p;miR-758-3p |
|  | ABLIM1 | 12 | miR-1185-5p;miR-379-5p;miR-411-5p;miR-431-3p;miR-432-5p;miR-433-3p;miR-485-3p;miR-485-5p;miR-487a-5p;miR-494-3p;miR-541-5p;miR-542-3p |
|  | ABLIM2 | 10 | miR-127-3p;miR-329-3p;miR-411-5p;miR-412-3p;miR-485-3p;miR-485-5p;miR-487a-3p;miR-493-3p;miR-496;miR-615-3p |
|  | ACOX1 | 15 | miR-1185-5p;miR-1197;miR-127-3p;miR-369-3p;miR-376c-3p;miR-381-3p;miR-432-5p;miR-433-3p;miR-487a-3p;miR-487a-5p;miR-495-3p;miR-539-5p;miR-543;miR-758-3p;miR-92b-3p |
|  | ADD2 | 18 | miR-1185-5p;miR-127-3p;miR-134-5p;miR-329-3p;miR-337-5p;miR-376a-3p;miR-376b-3p;miR-382-5p;miR-409-3p;miR-412-3p;miR-431-3p;miR-433-3p;miR-485-5p;miR-487a-3p;miR-487a-5p;miR-487b-3p;miR-496;miR-615-3p |
|  | AFF2 | 21 | miR-1185-5p;miR-1193;miR-127-3p;miR-136-5p;miR-370-3p;miR-381-3p;miR-382-5p;miR-409-3p;miR-412-3p;miR-431-3p;miR-432-5p;miR-485-3p;miR-485-5p;miR-487a-3p;miR-487a-5p;miR-493-3p;miR-539-5p;miR-541-5p;miR-542-3p;miR-615-3p;miR-758-3p |
|  | AGPAT3 | 10 | miR-134-5p;miR-154-5p;miR-299-3p;miR-337-5p;miR-412-3p;miR-431-3p;miR-485-5p;miR-503-5p;miR-543;miR-615-3p |
|  | AJUBA | 10 | miR-127-3p;miR-216b-5p;miR-432-5p;miR-487a-3p;miR-487a-5p;miR-487b-3p;miR-503-5p;miR-541-5p;miR-542-3p;miR-92b-3p |
|  | APOL6 | 12 | miR-1193;miR-134-5p;miR-382-5p;miR-409-3p;miR-424-5p;miR-432-5p;miR-450b-5p;miR-485-3p;miR-485-5p;miR-503-5p;miR-541-5p;miR-758-3p |
|  | AR | 11 | miR-127-3p;miR-299-3p;miR-337-5p;miR-370-3p;miR-376b-3p;miR-432-5p;miR-485-5p;miR-487b-3p;miR-493-3p;miR-503-5p;miR-541-5p |
|  | ARHGAP44 | 12 | miR-134-5p;miR-136-5p;miR-154-5p;miR-370-3p;miR-381-3p;miR-382-5p;miR-412-3p;miR-485-3p;miR-493-3p;miR-496;miR-758-3p;miR-92b-3p |
|  | ARHGEF37 | 12 | miR-134-5p;miR-323a-3p;miR-337-5p;miR-381-3p;miR-424-5p;miR-433-3p;miR-485-5p;miR-487a-5p;miR-543;miR-615-3p;miR-758-3p;miR-92b-3p |
|  | ATP8A2 | 10 | miR-127-3p;miR-329-3p;miR-381-3p;miR-409-3p;miR-487b-3p;miR-493-3p;miR-496;miR-539-5p;miR-615-3p;miR-758-3p |
|  | B3GALT5 | 17 | miR-1193;miR-1197;miR-134-5p;miR-299-3p;miR-370-3p;miR-377-3p;miR-379-5p;miR-431-3p;miR-432-5p;miR-485-3p;miR-485-5p;miR-487a-3p;miR-487a-5p;miR-503-5p;miR-541-5p;miR-615-3p;miR-758-3p |
|  | B4GALNT2 | 14 | miR-1185-5p;miR-1197;miR-216b-5p;miR-329-3p;miR-337-5p;miR-382-5p;miR-412-3p;miR-433-3p;miR-485-3p;miR-485-5p;miR-487a-5p;miR-503-5p;miR-758-3p;miR-92b-3p |
|  | BACH2 | 11 | miR-134-5p;miR-218-5p;miR-337-5p;miR-376c-3p;miR-381-3p;miR-382-5p;miR-412-3p;miR-487b-3p;miR-503-5p;miR-544a;miR-758-3p |
|  | BAIAP2 | 10 | miR-134-5p;miR-299-3p;miR-329-3p;miR-337-5p;miR-409-3p;miR-433-3p;miR-485-5p;miR-503-5p;miR-615-3p;miR-758-3p |
|  | BCAS1 | 12 | miR-127-3p;miR-134-5p;miR-299-3p;miR-329-3p;miR-382-5p;miR-412-3p;miR-433-3p;miR-485-3p;miR-485-5p;miR-539-5p;miR-615-3p;miR-92b-3p |
|  | BCL7A | 13 | miR-1193;miR-127-3p;miR-299-3p;miR-370-3p;miR-379-5p;miR-409-3p;miR-410-3p;miR-424-5p;miR-431-3p;miR-485-5p;miR-487a-5p;miR-493-3p;miR-541-5p |
|  | C10orf105 | 10 | miR-1193;miR-127-3p;miR-136-5p;miR-216b-5p;miR-432-5p;miR-485-5p;miR-493-3p;miR-503-5p;miR-615-3p;miR-92b-3p |
|  | C10orf67 | 13 | miR-1185-5p;miR-1193;miR-216b-5p;miR-337-5p;miR-370-3p;miR-382-5p;miR-411-5p;miR-431-3p;miR-433-3p;miR-485-5p;miR-487a-5p;miR-493-3p;miR-615-3p |
|  | C1orf116 | 12 | miR-299-3p;miR-382-5p;miR-485-3p;miR-487a-3p;miR-493-3p;miR-496;miR-539-5p;miR-541-5p;miR-542-3p;miR-544a;miR-615-3p;miR-92b-3p |
|  | C2CD2 | 11 | miR-1193;miR-134-5p;miR-376b-3p;miR-380-3p;miR-412-3p;miR-432-5p;miR-433-3p;miR-485-5p;miR-541-5p;miR-615-3p;miR-758-3p |
|  | C2orf88 | 11 | miR-1193;miR-134-5p;miR-299-3p;miR-381-3p;miR-409-3p;miR-432-5p;miR-433-3p;miR-485-5p;miR-494-3p;miR-503-5p;miR-541-5p |
|  | CACNA1D | 16 | miR-1197;miR-134-5p;miR-154-5p;miR-329-3p;miR-337-5p;miR-382-5p;miR-409-3p;miR-412-3p;miR-432-5p;miR-433-3p;miR-485-3p;miR-487a-3p;miR-487b-3p;miR-494-3p;miR-541-5p;miR-92b-3p |
|  | CACNA1E | 10 | miR-136-5p;miR-329-3p;miR-370-3p;miR-381-3p;miR-431-3p;miR-432-5p;miR-487a-3p;miR-541-5p;miR-542-3p;miR-758-3p |
|  | CACNB2 | 10 | miR-136-5p;miR-154-5p;miR-329-3p;miR-370-3p;miR-379-5p;miR-412-3p;miR-432-5p;miR-487a-5p;miR-503-5p;miR-543 |
|  | CACNB4 | 13 | miR-1185-5p;miR-154-5p;miR-218-5p;miR-337-5p;miR-377-3p;miR-379-5p;miR-381-3p;miR-409-3p;miR-433-3p;miR-487a-5p;miR-493-3p;miR-495-3p;miR-503-5p |
|  | CACNG5 | 15 | miR-1197;miR-127-3p;miR-136-5p;miR-376b-3p;miR-382-5p;miR-409-3p;miR-431-3p;miR-487a-3p;miR-487a-5p;miR-493-3p;miR-496;miR-503-5p;miR-541-5p;miR-615-3p;miR-92b-3p |
|  | CADM3 | 11 | miR-216b-5p;miR-329-3p;miR-381-3p;miR-382-5p;miR-424-5p;miR-432-5p;miR-433-3p;miR-485-3p;miR-487a-5p;miR-655-3p;miR-92b-3p |
|  | CAMK2B | 13 | miR-329-3p;miR-337-5p;miR-382-5p;miR-409-3p;miR-411-5p;miR-424-5p;miR-485-3p;miR-485-5p;miR-487a-3p;miR-493-3p;miR-541-5p;miR-758-3p;miR-92b-3p |
|  | CC2D2B | 10 | miR-134-5p;miR-154-5p;miR-216b-5p;miR-329-3p;miR-381-3p;miR-412-3p;miR-432-5p;miR-485-3p;miR-543;miR-544a |
|  | CCDC141 | 13 | miR-1193;miR-134-5p;miR-299-3p;miR-329-3p;miR-370-3p;miR-379-5p;miR-431-3p;miR-432-5p;miR-485-5p;miR-493-3p;miR-503-5p;miR-758-3p;miR-889-3p |
|  | CCL28 | 13 | miR-127-3p;miR-154-5p;miR-216b-5p;miR-377-3p;miR-409-3p;miR-412-3p;miR-431-3p;miR-485-5p;miR-493-3p;miR-539-5p;miR-541-5p;miR-615-3p;miR-758-3p |
|  | CCSER1 | 10 | miR-370-3p;miR-382-5p;miR-424-5p;miR-432-5p;miR-485-5p;miR-487a-3p;miR-493-3p;miR-496;miR-539-5p;miR-92b-3p |
|  | CDH6 | 10 | miR-127-3p;miR-299-3p;miR-381-3p;miR-382-5p;miR-409-3p;miR-431-3p;miR-432-5p;miR-485-5p;miR-493-3p;miR-494-3p |
|  | CDH8 | 12 | miR-134-5p;miR-370-3p;miR-381-3p;miR-409-3p;miR-412-3p;miR-485-3p;miR-485-5p;miR-487a-5p;miR-493-3p;miR-541-5p;miR-543;miR-92b-3p |
|  | CHST15 | 13 | miR-1197;miR-127-3p;miR-134-5p;miR-154-5p;miR-216b-5p;miR-299-3p;miR-370-3p;miR-376c-3p;miR-412-3p;miR-431-3p;miR-432-5p;miR-503-5p;miR-758-3p |
|  | CHST9 | 20 | miR-1193;miR-127-3p;miR-134-5p;miR-216b-5p;miR-299-3p;miR-323a-3p;miR-329-3p;miR-370-3p;miR-379-5p;miR-381-3p;miR-432-5p;miR-433-3p;miR-485-5p;miR-487a-5p;miR-494-3p;miR-503-5p;miR-541-5p;miR-615-3p;miR-758-3p;miR-92b-3p |
|  | CLIC5 | 13 | miR-1193;miR-134-5p;miR-329-3p;miR-412-3p;miR-431-3p;miR-432-5p;miR-485-5p;miR-487a-3p;miR-487a-5p;miR-503-5p;miR-541-5p;miR-615-3p;miR-92b-3p |
|  | CLMN | 11 | miR-1197;miR-127-3p;miR-329-3p;miR-337-5p;miR-370-3p;miR-381-3p;miR-409-3p;miR-485-5p;miR-493-3p;miR-503-5p;miR-92b-3p |
|  | CLSTN2 | 10 | miR-127-3p;miR-134-5p;miR-154-5p;miR-216b-5p;miR-381-3p;miR-410-3p;miR-432-5p;miR-487a-3p;miR-487b-3p;miR-493-3p |
|  | CORO2A | 10 | miR-1197;miR-127-3p;miR-134-5p;miR-323a-3p;miR-381-3p;miR-431-3p;miR-432-5p;miR-433-3p;miR-487a-5p;miR-503-5p |
|  | CREG2 | 10 | miR-216b-5p;miR-329-3p;miR-337-5p;miR-409-3p;miR-412-3p;miR-431-3p;miR-485-3p;miR-496;miR-503-5p;miR-615-3p |
|  | CTNNA3 | 15 | miR-1185-5p;miR-1193;miR-1197;miR-216b-5p;miR-299-3p;miR-323a-3p;miR-369-3p;miR-370-3p;miR-412-3p;miR-432-5p;miR-485-3p;miR-485-5p;miR-493-3p;miR-541-5p;miR-92b-3p |
|  | DAPK2 | 13 | miR-1185-5p;miR-134-5p;miR-216b-5p;miR-370-3p;miR-379-5p;miR-382-5p;miR-432-5p;miR-485-3p;miR-485-5p;miR-487a-5p;miR-496;miR-503-5p;miR-541-5p |
|  | DCLK3 | 10 | miR-154-5p;miR-216b-5p;miR-370-3p;miR-381-3p;miR-382-5p;miR-424-5p;miR-432-5p;miR-487a-3p;miR-493-3p;miR-544a |
|  | DGKI | 19 | miR-136-5p;miR-299-3p;miR-329-3p;miR-337-5p;miR-376a-3p;miR-376b-3p;miR-376c-3p;miR-377-3p;miR-381-3p;miR-409-3p;miR-412-3p;miR-432-5p;miR-485-3p;miR-485-5p;miR-493-3p;miR-539-5p;miR-541-5p;miR-542-3p;miR-758-3p |
|  | DPP6 | 12 | miR-1193;miR-216b-5p;miR-299-3p;miR-323a-3p;miR-337-5p;miR-411-5p;miR-485-5p;miR-487a-3p;miR-487a-5p;miR-494-3p;miR-503-5p;miR-615-3p |
|  | DTNA | 18 | miR-1185-5p;miR-1193;miR-134-5p;miR-376c-3p;miR-377-3p;miR-379-5p;miR-382-5p;miR-412-3p;miR-431-3p;miR-433-3p;miR-485-3p;miR-487b-3p;miR-493-3p;miR-494-3p;miR-503-5p;miR-539-5p;miR-541-5p;miR-92b-3p |
|  | ELAPOR1 | 15 | miR-1197;miR-134-5p;miR-377-3p;miR-381-3p;miR-382-5p;miR-409-3p;miR-412-3p;miR-432-5p;miR-485-3p;miR-485-5p;miR-487a-3p;miR-487b-3p;miR-493-3p;miR-541-5p;miR-92b-3p |
|  | ELMOD1 | 11 | miR-134-5p;miR-216b-5p;miR-370-3p;miR-412-3p;miR-432-5p;miR-485-5p;miR-487a-3p;miR-487b-3p;miR-503-5p;miR-542-3p;miR-758-3p |
|  | ELOVL6 | 11 | miR-1197;miR-154-5p;miR-382-5p;miR-409-3p;miR-412-3p;miR-487a-5p;miR-493-3p;miR-503-5p;miR-541-5p;miR-615-3p;miR-92b-3p |
|  | EMCN | 10 | miR-218-5p;miR-337-5p;miR-370-3p;miR-381-3p;miR-382-5p;miR-409-3p;miR-424-5p;miR-487a-5p;miR-493-3p;miR-543 |
|  | EMP2 | 11 | miR-127-3p;miR-299-3p;miR-382-5p;miR-412-3p;miR-424-5p;miR-433-3p;miR-485-3p;miR-485-5p;miR-496;miR-758-3p;miR-92b-3p |
|  | EPB41L5 | 16 | miR-1197;miR-127-3p;miR-134-5p;miR-154-5p;miR-337-5p;miR-376a-3p;miR-380-3p;miR-381-3p;miR-382-5p;miR-412-3p;miR-485-5p;miR-487a-5p;miR-503-5p;miR-615-3p;miR-758-3p;miR-92b-3p |
|  | ERBB4 | 12 | miR-216b-5p;miR-218-5p;miR-370-3p;miR-411-5p;miR-412-3p;miR-431-3p;miR-433-3p;miR-485-3p;miR-487a-5p;miR-495-3p;miR-496;miR-503-5p |
|  | ERG | 17 | miR-1193;miR-127-3p;miR-154-5p;miR-216b-5p;miR-370-3p;miR-381-3p;miR-382-5p;miR-409-3p;miR-412-3p;miR-424-5p;miR-431-3p;miR-432-5p;miR-485-5p;miR-487a-5p;miR-487b-3p;miR-615-3p;miR-758-3p |
|  | FAM107B | 13 | miR-1193;miR-127-3p;miR-134-5p;miR-216b-5p;miR-370-3p;miR-381-3p;miR-409-3p;miR-410-3p;miR-412-3p;miR-431-3p;miR-487a-3p;miR-487b-3p;miR-541-5p |
|  | FAM167A | 10 | miR-1193;miR-127-3p;miR-134-5p;miR-218-5p;miR-329-3p;miR-370-3p;miR-379-5p;miR-431-3p;miR-503-5p;miR-615-3p |
|  | FAM83B | 11 | miR-1193;miR-1197;miR-218-5p;miR-299-3p;miR-409-3p;miR-411-5p;miR-431-3p;miR-485-5p;miR-493-3p;miR-495-3p;miR-541-5p |
|  | FBXL22 | 11 | miR-134-5p;miR-323a-3p;miR-329-3p;miR-370-3p;miR-412-3p;miR-432-5p;miR-450a-5p;miR-485-3p;miR-487a-5p;miR-543;miR-615-3p |
|  | FGF1 | 19 | miR-1193;miR-134-5p;miR-154-5p;miR-216b-5p;miR-323a-3p;miR-370-3p;miR-379-5p;miR-381-3p;miR-411-5p;miR-424-5p;miR-431-3p;miR-432-5p;miR-433-3p;miR-485-5p;miR-487a-5p;miR-493-3p;miR-503-5p;miR-758-3p;miR-92b-3p |
|  | FHIP1A | 12 | miR-1185-5p;miR-1197;miR-127-3p;miR-329-3p;miR-376c-3p;miR-431-3p;miR-432-5p;miR-485-5p;miR-487a-3p;miR-487b-3p;miR-503-5p;miR-543 |
|  | FRK | 16 | miR-1197;miR-134-5p;miR-154-5p;miR-216b-5p;miR-218-5p;miR-370-3p;miR-379-5p;miR-409-3p;miR-432-5p;miR-433-3p;miR-485-5p;miR-493-3p;miR-503-5p;miR-543;miR-544a;miR-655-3p |
|  | FRMD1 | 17 | miR-1193;miR-1197;miR-127-3p;miR-134-5p;miR-154-5p;miR-379-5p;miR-381-3p;miR-409-3p;miR-432-5p;miR-433-3p;miR-485-3p;miR-485-5p;miR-487a-3p;miR-487a-5p;miR-541-5p;miR-615-3p;miR-758-3p |
|  | FRMD3 | 11 | miR-127-3p;miR-134-5p;miR-154-5p;miR-380-3p;miR-381-3p;miR-431-3p;miR-432-5p;miR-433-3p;miR-485-5p;miR-615-3p;miR-758-3p |
|  | FZD3 | 12 | miR-1197;miR-329-3p;miR-337-5p;miR-381-3p;miR-409-3p;miR-432-5p;miR-485-5p;miR-493-3p;miR-495-3p;miR-541-5p;miR-615-3p;miR-758-3p |
|  | GABRA2 | 19 | miR-1193;miR-1197;miR-127-3p;miR-134-5p;miR-299-3p;miR-323a-3p;miR-370-3p;miR-380-3p;miR-432-5p;miR-450a-5p;miR-485-3p;miR-485-5p;miR-487a-3p;miR-487b-3p;miR-493-3p;miR-541-5p;miR-543;miR-758-3p;miR-92b-3p |
|  | GABRA4 | 11 | miR-1185-5p;miR-127-3p;miR-134-5p;miR-329-3p;miR-370-3p;miR-376a-3p;miR-431-3p;miR-432-5p;miR-485-3p;miR-758-3p;miR-92b-3p |
|  | GABRP | 10 | miR-1193;miR-127-3p;miR-154-5p;miR-299-3p;miR-409-3p;miR-410-3p;miR-412-3p;miR-485-5p;miR-503-5p;miR-543 |
|  | GAN | 11 | miR-1185-5p;miR-1197;miR-127-3p;miR-299-3p;miR-337-5p;miR-370-3p;miR-380-3p;miR-409-3p;miR-424-5p;miR-432-5p;miR-487b-3p |
|  | GARIN1A | 10 | miR-127-3p;miR-299-3p;miR-329-3p;miR-376c-3p;miR-382-5p;miR-432-5p;miR-433-3p;miR-539-5p;miR-615-3p;miR-758-3p |
|  | GCNT2 | 10 | miR-127-3p;miR-299-3p;miR-376c-3p;miR-379-5p;miR-412-3p;miR-433-3p;miR-487a-5p;miR-541-5p;miR-758-3p;miR-92b-3p |
|  | GCSAML | 10 | miR-127-3p;miR-134-5p;miR-216b-5p;miR-329-3p;miR-381-3p;miR-412-3p;miR-431-3p;miR-432-5p;miR-485-5p;miR-541-5p |
|  | GFAP | 10 | miR-1185-5p;miR-1193;miR-1197;miR-337-5p;miR-409-3p;miR-485-3p;miR-485-5p;miR-487a-5p;miR-487b-3p;miR-503-5p |
|  | GFRA2 | 14 | miR-1185-5p;miR-1193;miR-1197;miR-216b-5p;miR-379-5p;miR-381-3p;miR-433-3p;miR-485-3p;miR-485-5p;miR-487a-3p;miR-487b-3p;miR-493-3p;miR-503-5p;miR-539-5p |
|  | GLP1R | 10 | miR-127-3p;miR-136-5p;miR-299-3p;miR-431-3p;miR-433-3p;miR-485-3p;miR-487a-5p;miR-503-5p;miR-539-5p;miR-758-3p |
|  | GPRIN2 | 16 | miR-1185-5p;miR-1193;miR-1197;miR-134-5p;miR-154-5p;miR-216b-5p;miR-370-3p;miR-381-3p;miR-412-3p;miR-432-5p;miR-433-3p;miR-485-3p;miR-485-5p;miR-487a-5p;miR-487b-3p;miR-496 |
|  | GREM1 | 14 | miR-136-5p;miR-216b-5p;miR-323a-3p;miR-329-3p;miR-370-3p;miR-381-3p;miR-382-5p;miR-431-3p;miR-433-3p;miR-485-3p;miR-487a-5p;miR-493-3p;miR-496;miR-544a |
|  | GRIK3 | 11 | miR-216b-5p;miR-299-3p;miR-329-3p;miR-337-5p;miR-370-3p;miR-381-3p;miR-485-3p;miR-485-5p;miR-493-3p;miR-503-5p;miR-758-3p |
|  | GRIN2B | 26 | miR-1197;miR-127-3p;miR-154-5p;miR-216b-5p;miR-299-3p;miR-329-3p;miR-337-5p;miR-370-3p;miR-379-5p;miR-381-3p;miR-382-5p;miR-409-3p;miR-412-3p;miR-431-3p;miR-432-5p;miR-433-3p;miR-485-3p;miR-485-5p;miR-487a-5p;miR-493-3p;miR-496;miR-539-5p;miR-541-5p;miR-655-3p;miR-758-3p;miR-92b-3p |
|  | HMGCS1 | 11 | miR-1193;miR-216b-5p;miR-337-5p;miR-380-3p;miR-381-3p;miR-412-3p;miR-431-3p;miR-432-5p;miR-496;miR-503-5p;miR-615-3p |
|  | HRH4 | 13 | miR-127-3p;miR-134-5p;miR-337-5p;miR-382-5p;miR-412-3p;miR-431-3p;miR-485-3p;miR-487a-5p;miR-487b-3p;miR-493-3p;miR-494-3p;miR-541-5p;miR-92b-3p |
|  | IGF2 | 13 | miR-1193;miR-1197;miR-299-3p;miR-329-3p;miR-370-3p;miR-381-3p;miR-409-3p;miR-431-3p;miR-432-5p;miR-485-3p;miR-485-5p;miR-487a-5p;miR-92b-3p |
|  | IGSF9B | 12 | miR-134-5p;miR-154-5p;miR-218-5p;miR-323a-3p;miR-376a-3p;miR-379-5p;miR-381-3p;miR-424-5p;miR-487a-5p;miR-503-5p;miR-543;miR-92b-3p |
|  | IL31RA | 12 | miR-1193;miR-337-5p;miR-382-5p;miR-410-3p;miR-424-5p;miR-432-5p;miR-485-3p;miR-485-5p;miR-487a-3p;miR-487a-5p;miR-487b-3p;miR-493-3p |
|  | ILDR2 | 15 | miR-1185-5p;miR-1193;miR-134-5p;miR-216b-5p;miR-329-3p;miR-376a-3p;miR-376c-3p;miR-381-3p;miR-412-3p;miR-433-3p;miR-485-3p;miR-485-5p;miR-496;miR-503-5p;miR-539-5p |
|  | KCND3 | 14 | miR-127-3p;miR-154-5p;miR-299-3p;miR-329-3p;miR-337-5p;miR-370-3p;miR-409-3p;miR-432-5p;miR-433-3p;miR-485-3p;miR-487a-5p;miR-493-3p;miR-503-5p;miR-615-3p |
|  | KCNK10 | 17 | miR-134-5p;miR-154-5p;miR-216b-5p;miR-337-5p;miR-376a-3p;miR-381-3p;miR-412-3p;miR-431-3p;miR-432-5p;miR-485-5p;miR-487a-5p;miR-493-3p;miR-494-3p;miR-496;miR-615-3p;miR-655-3p;miR-758-3p |
|  | KCNK3 | 12 | miR-1193;miR-127-3p;miR-154-5p;miR-323a-3p;miR-329-3p;miR-412-3p;miR-485-3p;miR-487a-5p;miR-493-3p;miR-615-3p;miR-758-3p;miR-92b-3p |
|  | KCNMA1 | 21 | miR-1185-5p;miR-1193;miR-134-5p;miR-154-5p;miR-216b-5p;miR-299-3p;miR-323a-3p;miR-370-3p;miR-381-3p;miR-382-5p;miR-409-3p;miR-412-3p;miR-431-3p;miR-432-5p;miR-485-3p;miR-487a-3p;miR-487a-5p;miR-539-5p;miR-544a;miR-758-3p;miR-92b-3p |
|  | KCNN3 | 14 | miR-127-3p;miR-323a-3p;miR-329-3p;miR-337-5p;miR-370-3p;miR-381-3p;miR-409-3p;miR-432-5p;miR-450a-5p;miR-485-3p;miR-503-5p;miR-541-5p;miR-544a;miR-758-3p |
|  | KLHL3 | 14 | miR-1193;miR-1197;miR-134-5p;miR-154-5p;miR-376c-3p;miR-381-3p;miR-431-3p;miR-485-3p;miR-487a-3p;miR-487b-3p;miR-495-3p;miR-496;miR-543;miR-615-3p |
|  | KRT40 | 11 | miR-1185-5p;miR-127-3p;miR-216b-5p;miR-299-3p;miR-370-3p;miR-379-5p;miR-382-5p;miR-409-3p;miR-412-3p;miR-493-3p;miR-758-3p |
|  | LANCL3 | 11 | miR-127-3p;miR-216b-5p;miR-299-3p;miR-370-3p;miR-381-3p;miR-382-5p;miR-433-3p;miR-450a-5p;miR-487b-3p;miR-496;miR-615-3p |
|  | LDB3 | 12 | miR-1193;miR-127-3p;miR-134-5p;miR-154-5p;miR-329-3p;miR-376b-3p;miR-377-3p;miR-485-5p;miR-496;miR-503-5p;miR-541-5p;miR-543 |
|  | LEP | 10 | miR-127-3p;miR-134-5p;miR-216b-5p;miR-329-3p;miR-370-3p;miR-487a-5p;miR-493-3p;miR-503-5p;miR-758-3p;miR-92b-3p |
|  | LGSN | 10 | miR-1185-5p;miR-127-3p;miR-337-5p;miR-370-3p;miR-380-3p;miR-432-5p;miR-433-3p;miR-485-5p;miR-487a-5p;miR-493-3p |
|  | LIPH | 12 | miR-1193;miR-370-3p;miR-376b-3p;miR-424-5p;miR-431-3p;miR-432-5p;miR-433-3p;miR-485-3p;miR-485-5p;miR-541-5p;miR-758-3p;miR-92b-3p |
|  | LMCD1 | 13 | miR-1193;miR-1197;miR-134-5p;miR-329-3p;miR-370-3p;miR-409-3p;miR-412-3p;miR-431-3p;miR-432-5p;miR-485-3p;miR-485-5p;miR-487a-3p;miR-541-5p |
|  | LMO3 | 10 | miR-1185-5p;miR-1193;miR-134-5p;miR-154-5p;miR-337-5p;miR-424-5p;miR-431-3p;miR-432-5p;miR-485-3p;miR-487a-5p |
|  | LMX1B | 13 | miR-1197;miR-127-3p;miR-136-5p;miR-299-3p;miR-329-3p;miR-409-3p;miR-412-3p;miR-431-3p;miR-432-5p;miR-485-5p;miR-493-3p;miR-541-5p;miR-615-3p |
|  | LRRC2 | 11 | miR-134-5p;miR-154-5p;miR-216b-5p;miR-329-3p;miR-369-3p;miR-370-3p;miR-412-3p;miR-432-5p;miR-487a-5p;miR-495-3p;miR-496 |
|  | LSAMP | 11 | miR-134-5p;miR-136-5p;miR-370-3p;miR-409-3p;miR-412-3p;miR-431-3p;miR-432-5p;miR-487a-5p;miR-493-3p;miR-503-5p;miR-539-5p |
|  | LYPD6 | 11 | miR-1193;miR-1197;miR-134-5p;miR-376b-3p;miR-381-3p;miR-432-5p;miR-485-5p;miR-541-5p;miR-544a;miR-758-3p;miR-92b-3p |
|  | MACC1 | 15 | miR-127-3p;miR-134-5p;miR-136-5p;miR-323a-3p;miR-329-3p;miR-337-5p;miR-370-3p;miR-382-5p;miR-409-3p;miR-411-5p;miR-412-3p;miR-485-5p;miR-487a-5p;miR-494-3p;miR-655-3p |
|  | MAP3K9 | 15 | miR-1197;miR-127-3p;miR-134-5p;miR-299-3p;miR-323a-3p;miR-370-3p;miR-410-3p;miR-432-5p;miR-485-3p;miR-493-3p;miR-541-5p;miR-543;miR-615-3p;miR-655-3p;miR-92b-3p |
|  | MAP4K2 | 11 | miR-1197;miR-134-5p;miR-136-5p;miR-218-5p;miR-329-3p;miR-409-3p;miR-412-3p;miR-433-3p;miR-494-3p;miR-503-5p;miR-92b-3p |
|  | MAPT | 10 | miR-134-5p;miR-154-5p;miR-216b-5p;miR-431-3p;miR-432-5p;miR-433-3p;miR-485-5p;miR-487a-5p;miR-503-5p;miR-541-5p |
|  | MARVELD3 | 11 | miR-1193;miR-127-3p;miR-337-5p;miR-370-3p;miR-377-3p;miR-412-3p;miR-431-3p;miR-432-5p;miR-485-5p;miR-493-3p;miR-495-3p |
|  | MCTP2 | 10 | miR-127-3p;miR-134-5p;miR-136-5p;miR-412-3p;miR-431-3p;miR-450b-5p;miR-485-5p;miR-494-3p;miR-503-5p;miR-758-3p |
|  | MEGF9 | 13 | miR-134-5p;miR-154-5p;miR-329-3p;miR-370-3p;miR-412-3p;miR-432-5p;miR-433-3p;miR-485-5p;miR-493-3p;miR-496;miR-541-5p;miR-543;miR-615-3p |
|  | MFAP3L | 13 | miR-1197;miR-134-5p;miR-216b-5p;miR-218-5p;miR-299-3p;miR-323a-3p;miR-381-3p;miR-432-5p;miR-487b-3p;miR-493-3p;miR-503-5p;miR-544a;miR-615-3p |
|  | MGAT4C | 18 | miR-1197;miR-127-3p;miR-154-5p;miR-299-3p;miR-329-3p;miR-376a-3p;miR-376b-3p;miR-382-5p;miR-431-3p;miR-432-5p;miR-485-5p;miR-487a-3p;miR-487a-5p;miR-493-3p;miR-503-5p;miR-541-5p;miR-758-3p;miR-92b-3p |
|  | MTA3 | 10 | miR-134-5p;miR-216b-5p;miR-370-3p;miR-409-3p;miR-412-3p;miR-485-3p;miR-487a-5p;miR-493-3p;miR-503-5p;miR-543 |
|  | NCMAP | 11 | miR-127-3p;miR-134-5p;miR-216b-5p;miR-329-3p;miR-370-3p;miR-381-3p;miR-409-3p;miR-485-3p;miR-485-5p;miR-503-5p;miR-92b-3p |
|  | NEBL | 13 | miR-1193;miR-127-3p;miR-134-5p;miR-337-5p;miR-370-3p;miR-381-3p;miR-409-3p;miR-424-5p;miR-432-5p;miR-485-3p;miR-485-5p;miR-503-5p;miR-92b-3p |
|  | NECTIN1 | 10 | miR-127-3p;miR-134-5p;miR-381-3p;miR-412-3p;miR-431-3p;miR-433-3p;miR-487a-5p;miR-541-5p;miR-542-3p;miR-92b-3p |
|  | NEDD4L | 11 | miR-1197;miR-127-3p;miR-134-5p;miR-216b-5p;miR-381-3p;miR-424-5p;miR-431-3p;miR-493-3p;miR-494-3p;miR-503-5p;miR-543 |
|  | NEGR1 | 12 | miR-134-5p;miR-136-5p;miR-154-5p;miR-299-3p;miR-433-3p;miR-450b-5p;miR-485-3p;miR-485-5p;miR-487a-5p;miR-487b-3p;miR-496;miR-503-5p |
|  | NFASC | 11 | miR-127-3p;miR-154-5p;miR-377-3p;miR-410-3p;miR-431-3p;miR-432-5p;miR-450b-5p;miR-485-5p;miR-487a-5p;miR-543;miR-92b-3p |
|  | NFIB | 19 | miR-127-3p;miR-329-3p;miR-376b-3p;miR-377-3p;miR-381-3p;miR-382-5p;miR-409-3p;miR-410-3p;miR-412-3p;miR-431-3p;miR-432-5p;miR-433-3p;miR-485-5p;miR-487a-5p;miR-487b-3p;miR-493-3p;miR-539-5p;miR-615-3p;miR-758-3p |
|  | NKD1 | 12 | miR-1185-5p;miR-329-3p;miR-370-3p;miR-379-5p;miR-381-3p;miR-382-5p;miR-412-3p;miR-450b-5p;miR-485-5p;miR-493-3p;miR-541-5p;miR-758-3p |
|  | NOS1 | 14 | miR-136-5p;miR-154-5p;miR-323a-3p;miR-329-3p;miR-337-5p;miR-409-3p;miR-412-3p;miR-432-5p;miR-433-3p;miR-450a-5p;miR-487b-3p;miR-493-3p;miR-495-3p;miR-758-3p |
|  | NPFFR1 | 10 | miR-329-3p;miR-381-3p;miR-412-3p;miR-450a-5p;miR-485-3p;miR-495-3p;miR-541-5p;miR-543;miR-655-3p;miR-92b-3p |
|  | NPR3 | 13 | miR-1193;miR-127-3p;miR-134-5p;miR-136-5p;miR-154-5p;miR-412-3p;miR-431-3p;miR-485-5p;miR-487b-3p;miR-495-3p;miR-543;miR-544a;miR-615-3p |
|  | NRXN1 | 12 | miR-134-5p;miR-136-5p;miR-154-5p;miR-299-3p;miR-323a-3p;miR-450a-5p;miR-485-3p;miR-487a-3p;miR-487a-5p;miR-541-5p;miR-542-3p;miR-758-3p |
|  | NRXN3 | 13 | miR-136-5p;miR-154-5p;miR-382-5p;miR-409-3p;miR-412-3p;miR-431-3p;miR-432-5p;miR-433-3p;miR-485-3p;miR-485-5p;miR-541-5p;miR-544a;miR-615-3p |
|  | NT5C1A | 16 | miR-1193;miR-1197;miR-134-5p;miR-136-5p;miR-377-3p;miR-379-5p;miR-382-5p;miR-412-3p;miR-431-3p;miR-485-3p;miR-485-5p;miR-487a-5p;miR-487b-3p;miR-503-5p;miR-541-5p;miR-758-3p |
|  | NTNG1 | 17 | miR-1185-5p;miR-1193;miR-127-3p;miR-216b-5p;miR-299-3p;miR-382-5p;miR-410-3p;miR-412-3p;miR-432-5p;miR-485-5p;miR-487a-5p;miR-487b-3p;miR-493-3p;miR-543;miR-615-3p;miR-889-3p;miR-92b-3p |
|  | NTRK2 | 25 | miR-1193;miR-1197;miR-127-3p;miR-134-5p;miR-136-5p;miR-216b-5p;miR-218-5p;miR-323a-3p;miR-370-3p;miR-377-3p;miR-379-5p;miR-381-3p;miR-382-5p;miR-412-3p;miR-424-5p;miR-432-5p;miR-433-3p;miR-485-3p;miR-485-5p;miR-487b-3p;miR-493-3p;miR-503-5p;miR-539-5p;miR-615-3p;miR-758-3p |
|  | NTRK3 | 23 | miR-1197;miR-127-3p;miR-134-5p;miR-154-5p;miR-216b-5p;miR-329-3p;miR-337-5p;miR-370-3p;miR-381-3p;miR-382-5p;miR-424-5p;miR-431-3p;miR-432-5p;miR-433-3p;miR-485-3p;miR-487b-3p;miR-493-3p;miR-495-3p;miR-503-5p;miR-539-5p;miR-541-5p;miR-542-3p;miR-92b-3p |
|  | OPRD1 | 11 | miR-1193;miR-1197;miR-127-3p;miR-216b-5p;miR-329-3p;miR-370-3p;miR-382-5p;miR-431-3p;miR-541-5p;miR-543;miR-615-3p |
|  | OSBPL10 | 14 | miR-134-5p;miR-216b-5p;miR-370-3p;miR-381-3p;miR-409-3p;miR-431-3p;miR-433-3p;miR-485-3p;miR-487b-3p;miR-493-3p;miR-494-3p;miR-615-3p;miR-758-3p;miR-92b-3p |
|  | PLA2G4F | 10 | miR-1193;miR-216b-5p;miR-299-3p;miR-409-3p;miR-412-3p;miR-432-5p;miR-485-5p;miR-487a-5p;miR-615-3p;miR-92b-3p |
|  | PLCE1 | 10 | miR-1185-5p;miR-127-3p;miR-136-5p;miR-154-5p;miR-216b-5p;miR-370-3p;miR-376a-3p;miR-495-3p;miR-758-3p;miR-92b-3p |
|  | PLD5 | 12 | miR-127-3p;miR-329-3p;miR-409-3p;miR-431-3p;miR-432-5p;miR-433-3p;miR-485-5p;miR-487a-5p;miR-493-3p;miR-494-3p;miR-615-3p;miR-92b-3p |
|  | PLEKHA7 | 12 | miR-218-5p;miR-370-3p;miR-381-3p;miR-382-5p;miR-412-3p;miR-432-5p;miR-485-5p;miR-493-3p;miR-544a;miR-615-3p;miR-758-3p;miR-92b-3p |
|  | PLEKHS1 | 10 | miR-1193;miR-127-3p;miR-154-5p;miR-370-3p;miR-381-3p;miR-412-3p;miR-431-3p;miR-485-5p;miR-493-3p;miR-541-5p |
|  | PLXNA4 | 13 | miR-1185-5p;miR-1193;miR-1197;miR-134-5p;miR-380-3p;miR-412-3p;miR-424-5p;miR-432-5p;miR-485-3p;miR-487a-5p;miR-541-5p;miR-615-3p;miR-92b-3p |
|  | PPARA | 10 | miR-1193;miR-134-5p;miR-337-5p;miR-370-3p;miR-380-3p;miR-381-3p;miR-409-3p;miR-487a-5p;miR-496;miR-758-3p |
|  | PRIMA1 | 11 | miR-1185-5p;miR-1193;miR-329-3p;miR-409-3p;miR-412-3p;miR-432-5p;miR-487a-5p;miR-487b-3p;miR-503-5p;miR-541-5p;miR-543 |
|  | PRLR | 18 | miR-1193;miR-1197;miR-127-3p;miR-216b-5p;miR-218-5p;miR-323a-3p;miR-337-5p;miR-370-3p;miR-380-3p;miR-381-3p;miR-409-3p;miR-412-3p;miR-431-3p;miR-432-5p;miR-433-3p;miR-485-3p;miR-487a-5p;miR-503-5p |
|  | PRUNE2 | 10 | miR-1193;miR-134-5p;miR-136-5p;miR-370-3p;miR-379-5p;miR-431-3p;miR-432-5p;miR-485-3p;miR-485-5p;miR-495-3p |
|  | PRXL2A | 12 | miR-134-5p;miR-136-5p;miR-329-3p;miR-370-3p;miR-409-3p;miR-412-3p;miR-487a-5p;miR-487b-3p;miR-493-3p;miR-494-3p;miR-758-3p;miR-92b-3p |
|  | PXMP4 | 18 | miR-127-3p;miR-134-5p;miR-136-5p;miR-154-5p;miR-216b-5p;miR-218-5p;miR-299-3p;miR-329-3p;miR-370-3p;miR-379-5p;miR-431-3p;miR-432-5p;miR-433-3p;miR-485-3p;miR-485-5p;miR-503-5p;miR-542-3p;miR-615-3p |
|  | RAB11FIP4 | 10 | miR-1197;miR-323a-3p;miR-370-3p;miR-382-5p;miR-409-3p;miR-412-3p;miR-485-3p;miR-487a-5p;miR-615-3p;miR-92b-3p |
[truncated: 96,273 more chars]
